# The carbon footprint of products used in five common surgical operations: identifying contributing products and processes

**DOI:** 10.1177/01410768231166135

**Published:** 2023-04-13

**Authors:** Chantelle Rizan, Robert Lillywhite, Malcom Reed, Mahmood F Bhutta

**Affiliations:** 1 Brighton and Sussex Medical School, Royal Sussex County Hospital, Brighton, BN25BE, UK; 2 School of Life Sciences, University of Warwick, Coventry, CV4 7AL, UK; 3 ENT Department, University Hospitals Sussex NHS Foundation Trust, Brighton, BN2 5BE, UK

**Keywords:** Environmental issues, general surgery, orthopaedic and trauma surgery, otolaryngology, surgery

## Abstract

**Objectives:**

Mitigating carbon footprint of products used in resource-intensive areas such as surgical operating rooms will be important in achieving net zero carbon healthcare. The aim of this study was to evaluate the carbon footprint of products used within five common operations, and to identify the biggest contributors (hotspots).

**Design:**

A predominantly process-based carbon footprint analysis was conducted for products used in the five highest volume surgical operations performed in the National Health System in England.

**Setting:**

The carbon footprint inventory was based on direct observation of 6–10 operations/type, conducted across three sites within one NHS Foundation Trust in England.

**Participants:**

Patients undergoing primary elective carpal tunnel decompression, inguinal hernia repair, knee arthroplasty, laparoscopic cholecystectomy, tonsillectomy (March 2019 – January 2020).

**Main outcome measures:**

We determined the carbon footprint of the products used in each of the five operations, alongside greatest contributors through analysis of individual products and of underpinning processes.

**Results:**

The mean average carbon footprint of products used for carpal tunnel decompression was 12.0 kg CO_2_e (carbon dioxide equivalents); 11.7 kg CO_2_e for inguinal hernia repair; 85.5 kg CO_2_e for knee arthroplasty; 20.3 kg CO_2_e for laparoscopic cholecystectomy; and 7.5 kg CO_2_e for tonsillectomy. Across the five operations, 23% of product types were responsible for ≥80% of the operation carbon footprint. Products with greatest carbon contribution for each operation type were the single-use hand drape (carpal tunnel decompression), single-use surgical gown (inguinal hernia repair), bone cement mix (knee arthroplasty), single-use clip applier (laparoscopic cholecystectomy) and single-use table drape (tonsillectomy). Mean average contribution from production of single-use items was 54%, decontamination of reusables 20%, waste disposal of single-use items 8%, production of packaging for single-use items 6% and linen laundering 6%.

**Conclusions:**

Change in practice and policy should be targeted towards those products making greatest contribution, and should include reducing single-use items and switching to reusables, alongside optimising processes for decontamination and waste disposal, modelled to reduce carbon footprint of these operations by 23%–42%.

## Introduction

The healthcare sector is responsible for an estimated 4.4% of global greenhouse gas emissions.^1^ Medical equipment accounts for 10% of the National Health Service (NHS) carbon footprint in England,^2^ and mitigating this, particularly for products used in resource-intensive areas, such as surgical operating rooms, will be an important step in meeting environmental sustainability goals, such as commitments by 18 countries to reach net zero carbon healthcare.^3^

Two systematic reviews of the carbon footprint of surgical operations found the three major contributors to be: energy consumption; anaesthesia; and single-use products (with smaller contributions from reusable products, water usage and capital goods).^4,5^ The majority (90%–99%) of operating room energy consumption relates to heating, ventilation and air conditioning (HVAC), reduceable through operating room design (including installation of occupancy sensors or set-back systems after hours, reduced air flow turnover and newer buildings with improved energy efficiency) and use of renewable energy sources.^6^ The environmental impact of general anaesthesia can be reduced through use of inhalational agents with low global warming potential, gas scavenging systems and preferencing regional or total intravenous anaesthesia.^7^ Reducing single-use equipment in the operating room is also an important strategy,^8^ but to date has received little analysis.

A ‘carbon footprint’ is an estimate of direct and indirect greenhouse gases associated with a given product or process, with non-carbon greenhouse gases equated to carbon dioxide equivalents (CO_2_e) based on their global warming potential, allowing summation. Studies have previously estimated the carbon footprint of products used in surgery relative to other components of whole operations (summarised in systematic reviews),^4,5^ associating products with up to three-quarters of the carbon footprint of a cataract operation.^9^ Few previous studies^9–11^ have reported the carbon footprint of individual surgical products, although a carbon footprint of a hysterectomy operation identified production of cotton (used for laparotomy pads and operating towels), spun bound polypropylene (gowns, drapes and instrument tray wrap), and paper and cardboard (packaging) as large contributors.^11^ Studies focusing on individual surgical products include those evaluating surgical textiles,^12^ surgical scissors^13^ and sharps containers,^14^ finding lower carbon footprint for reusable compared with single-use equivalents. Strategy towards reducing greenhouse gas emissions associated with the operating room includes reduction, reuse, repair and recycling of products.^15^

Choice of products for an operation is largely determined by surgeons, and many seem willing to mitigate carbon footprint.^16^ Ours is the first study to systematically evaluate the carbon footprint of products used in common operations, and to identify the biggest contributors (hotspots) to target for change in practice and policy.

## Methods

A carbon footprint was conducted in accordance with the Greenhouse Gas Accounting Sector Guidance for Pharmaceutical Products and Medical Devices,^17^ which builds upon the Greenhouse Gas Protocol Product Life Cycle Accounting and Reporting Standard.^18^ A process-based approach using activity data was used for the majority of products, but where unfeasible (due to lack of relevant emission factors), an environmentally extended input–output (EEIO) model was used based on financial data.^18^

### Scope and boundary setting

We selected for analysis the five highest volume surgical procedures performed in the NHS in England using the 2017–2018 Hospital Episode Statistics database,^19^ and included only operations typically performed by surgical specialties recognised by the Royal College of Surgeons of England,^20^ and excluded diagnostic procedures, or those commonly performed outside the operating room. These were total knee arthroplasty (80,627 performed in 2017–2018 in England), cholecystectomy (73,069), inguinal hernia repair (64,650), carpal tunnel decompression (47,023) and tonsillectomy (46,131).^19^ The functional unit was defined as one of each of these operations.

The system boundary was set to include production of materials for products used within the operating room, and their associated primary packaging (excluding bulk packaging), and encompassed ‘cradle to factory gate’ activity, including raw material extraction, transportation to primary processing site, primary processing and manufacture of materials (Supplementary Figure 1). Emission factors available for a small number of materials (indicated in Supplementary Table 1) included transportation from the factory gate to point of use (equating to the manufacturing site for multi-component items). Where an EEIO model was used, this encompassed all activities from raw material extraction to the point of sale. Due to study scale and lack of available data, we excluded processes in the manufacture of multi-component items (beyond production of constituent materials) and onward distribution to site of use. We included waste disposal, steam sterilisation of reusable instruments and laundering of textiles. Sterilisation of single-use items was excluded as contribution to total carbon footprint of such items has previously been estimated at <1%.^21^ We also excluded operating room capital goods (as these were unlikely to reach the significance threshold),^18^ anaesthetic components (aside from those administered by the surgeon) and operating room electricity (including HVAC) and water consumption.

### Inventory analysis

The carbon footprint inventory was based on direct observation of operating rooms across three sites of University Hospital Sussex NHS Foundation Trust between March 2019 and January 2020. We observed 10 of each type of operation, except for laparoscopic cholecystectomy and inguinal hernia repair, where only six of each were observed (onset of the COVID-19 pandemic curtailed data collection). Emergency and revision cases were excluded. We found that there was little variation between operations, and this was reliably captured by observing at least six operations.

The material composition of items was determined through packaging and manufacturer information where available, or through expert assessment by author RL. We weighed individual material components using Fisherbrand FPRS4202 Precision balance scales (Fisher Scientific, Loughborough, UK). Where it was not possible to deconstruct multi-component items, weight was allocated based on probable ratio. For reusable products, the number of product lifetime uses was estimated through a combination of product manufacturer correspondence, a one-year retrospective audit of decontamination of instruments at the Royal Sussex County Hospital (providing data on individual instrument sets’ number of uses per year) and expert assessment of instrument age and likely lifespan (for example, based on instrument markings, design and manufacturer stamps). Some ‘single-use’ items were used across multiple operations in a day, and typical number of uses was determined through discussion with operating room staff. The choice of waste streams used for disposal of single-use products was directly observed for individual operations (non-specified recycling was processed as domestic waste at the study site and was classified as domestic waste). For reusable products, waste streams used at the end of product life were determined through discussion with the on-site sterile services department and the hospitals’ linen supplier.

### Impact assessment

The carbon footprint of each product used within operations was determined through applying best available emission factors from a variety of sources (Supplementary Table 1). The Inventory of Carbon and Energy (ICE) database (version 3)^22^ was the primary source of emission factors for materials, as this uses average data for materials supplied to the UK. Where materials were not included in the ICE database, we used the UK Government Greenhouse Gas Conversion Factors for Company Reporting database,^23^ and where not included there either, we used the Ecoinvent v3.6 database embedded within SimaPro v9.10,^24^ which includes global average processes. Process-based emission factors were unavailable for the majority of pharmaceuticals and cleaning chemicals (aside from some anaesthetic agents sourced from relevant literature),^25^ and so financial spend emission factors were used from the Small World Consulting Carbon Factors Dataset v5.3,^26^ which was also used for stainless-steel process-based emission factors. Our previous studies (conducted at the same study site) were used to provide emission factors for healthcare waste^27^ and decontamination.^28^ There were no available emission factor data specific to healthcare linen laundering, and so we used activity data from previous studies^29,30^ alongside transportation data specific to the study site (Supplementary Methods 1).

### Hotspot analysis

We determined the mean average carbon footprint of each operation type. To ascertain products or processes to potentially prioritise when mitigating a carbon footprint, we categorised the carbon footprint for products (single-use and reusable) in each operation type on three measures:

1. By product category. We determined the total carbon footprint associated with the use of personal protective equipment, surgical equipment and devices; patient and/or instrument table drapes; cleaning products; and pharmaceuticals (each categorised as single-use or reusable as appropriate).
2. By individual product. We ordered products by individual carbon footprint, and determined those that cumulatively contributed ≥80% to the total (considered to be the 'majority’, in line with the Pareto Principle). ^31^
3. By process. We determined the total carbon footprint associated with the production of products, production of packaging, decontamination, linen laundering and waste disposal.

For the purposes of hotspot analysis, the carbon footprint of decontamination was allocated across instruments within a set according to weight, while the carbon footprint of washing instrument set containers was assigned to the containers themself. Where the carbon footprint of material production was determined using the EEIO method, the majority (90%) was allocated to the product, with 10% to packaging (aligning with the proportional split observed across the dataset). When reporting mean averages across the whole dataset, these were weighted equally for each of the five operation types.

## Results

### Carbon footprint of products used in operations

The mean number of reusable instrument sets, single-use instrument packs, individually wrapped reusable instruments, pharmaceuticals and other items used in each operation type are summarised in Table 1. Supplementary Tables 2–11 show the carbon footprint of reusable and single-use products used across each operation type, Supplementary Tables 12–16 show the number of each product type used and Supplementary Tables 17–21 combine these data to calculate overall carbon footprint of each product.

**Table 1.** Number of products used in each type of operation.

	Mean number of products (range)					
Operation type (number of operations observed)	Reusable instrument set	Single-use instrument pack	Individually wrapped reusable instruments	Other reusable products	Other single-use products	Pharmaceutical items
Carpal tunnel decompression (10)	1 (1–1)	1 (1–1)	–	6.4 (5–8)	46.9 (40–52)	3.7 (3–5)
Inguinal hernia repair (6)	1 (1–1)	–	0.3 (0–1)	9.7 (8–11)	58.2 (47–74)	4.6 (2–8)
Knee arthroplasty (10)	6.2 (5–8)	1 (1–1)	4.2 (2–5)	5.8 (5–7)	113.4 (89–143)	9.5 (8–11)
Laparoscopic cholecystectomy (6)	3 (3–3)	–	1.3 (1–2)	11.5 (10–13)	63.8 (61–71)	6.7 (6–8)
Tonsillectomy (10)	1 (1–1)	–	–	9.2 (7–12)	30.4 (21–35)	3.2 (2–4)

Note: Reusable instrument sets and single-use instrument packs contain multiple items. Individual product details are specified in Supplementary Tables 2–11.

The mean carbon footprint of products used for carpal tunnel decompression was 12.0 kg CO_2_e (range 11.3–12.9 kg CO_2_e); for inguinal hernia repair 11.7 kg CO_2_e (range 10.1–15.0); for knee arthroplasty 85.5 kg CO_2_e (range 72.3–94.9); for laparoscopic cholecystectomy 20.3 kg CO_2_e (range 18.8–22.0); and 7.5 kg CO_2_e for tonsillectomy (range 6.3–8.2).

### Hotspot analysis of products and underlying processes

Table 2 shows the mean average contribution of product categories to total carbon footprint of products used in five operation types (individual operation results are provided in Supplementary Table 22). Across the dataset, around two-thirds of carbon footprint related to single-use products, and one-third to reusable products (Supplementary Figure 2). The greatest contributions were from single-use equipment and medical devices (mean average 24%), and by reusable instrument sets and individually wrapped reusable instruments (24%). This was followed by single-use patient or instrument table drapes (14%), pharmaceuticals (13%), single-use personal protective equipment (PPE) (11%), single-use items associated with cleaning and waste (6%), reusable PPE (4%), reusable patient and instrument table drapes (3%) and non-set reusable equipment (0.1%).

**Table 2.** Carbon footprint of products used in operations by product category.

		Carbon footprint (g CO_2_e)											
		Reusable items					Single-use items						
Operation		Instrument sets and individually wrapped instruments	Personal protective equipment	Patient and/or instrument table drapes	Non-set equipment	Total reusable items	Personal protective equipment	Patient and/or instrument table drapes	Equipment and medical devices	Pharmaceuticals	Cleaning products, waste	Total single-use items	Total for operation
Carpal tunnel decompression	Mean ± sd	1922 ± 0	265 ± 49	N/A	56 ± 38	**2242 ± 39**	2722 ± 400	3881 ± 0	1699 ± 301	795 ± 242	671 ± 36	**9768 ± 399**	**12,011 ± 412**
	Percentage of total	16%	2%		0.46%	**19%**	23%	32%	14%	7%	6%	**81%**	**100%**
	Range	1922–1922	232–371		0–79	**2154–2294**	1890–3330	3881–3881	1388–2245	422–1123	629–756	**9060–10,583**	**11,294–12,863**
Inguinal hernia repair	Mean ± sd	2669 ± 360	560 ± 213	1042 ± 235	10 ± 0.2	**4281 ± 323**	1850 ± 652	666 ± 620	2596 ± 919	1426 ± 1463	861 ± 96	**7398 ± 1631**	**11,679 ± 1840**
	Percentage of total	23%	5%	9%	0.08%	**37**	16%	6%	22%	12%	7%	**63%**	**100%**
	Range	2396–3214	290–796	745–1222	9–10	**3860–4620**	1356–2705	0–1667	1744–3927	145–4070	707–926	**5705–10,343**	**10,128–14,963**
Knee arthroplasty	Mean ± sd	14,808 ± 2162	433 ± 65	N/A	0.09 ± 0.2	**15,242 ± 2133**	5904 ± 1217	12,801 ± 270	22,781 ± 2034	26,807 ± 5912	1919 ± 213	**70,212 ± 7190**	**85,454 ± 7097**
	Percentage of total	17%	1%		0.0001%	**18%**	7%	15%	27%	31%	2%	**82%**	**100%**
	Range	12,031–18,965	387–542		0–0.44	**12,418–19,351**	3915 7410	12,421–13,171	20,774–25,340	15,407–33,607	1671–2320	**55,145–80,120**	**72,247–94,875**
Laparoscopic cholecystectomy	Mean ± sd	6674 ± 109	977 ± 226	1165 ± 53	9 ± 0	**8825 ± 326**	1121 ± 560	740 ± 38	7253 ± 1292	1419 ± 487	932 ± 91	**11,465 ± 1141**	**20,290 ± 1408**
	Percentage of total	33%	5%	6%	0.05%	**43%**	6%	4%	36%	7%	5%	**57%**	**100%**
	Range	6604–6820	659–1263	1098–1222	9–9	**8371–9283**	551–2095	705–775	5646–8655	939–2254	801–1014	**10,190–12,772**	**18,751–22,024**
Tonsillectomy	Mean ± sd	2296 ± 36	712 ± 56	94 ± 197	N/A	**3102 ± 211**	387 ± 59	1060 ± 150	1754 ± 494	460 ± 227	711 ± 50	**4372 ± 758**	**7474 ± 734**
	Percentage of total	31%	10%	1%		**42%**	5%	14%	23%	6%	10%	**58%**	**100%**
	Range	2279–2364	660–808	0–475		**2945–3499**	323–489	755–1131	1159–2268	115–658	663–795	**3392–5129**	**6337–8170**
Percentage contribution of product category to total carbon footprint, mean average across all five operations		24%	4%	3%	0.1%	**32%**	11%	14%	24%	13%	6%	**68%**	**100%**

Mean results across dataset – see Supplementary Table 22 for results by individual operation. ‘Instrument sets and individually wrapped instruments’ carbon footprint includes single-use sterile barrier system and single-use items within the set where relevant. The number of items used are specified in Supplementary Tables 12–16. CO_2_e: carbon dioxide equivalents; sd: standard deviation.

**Figure 1.**
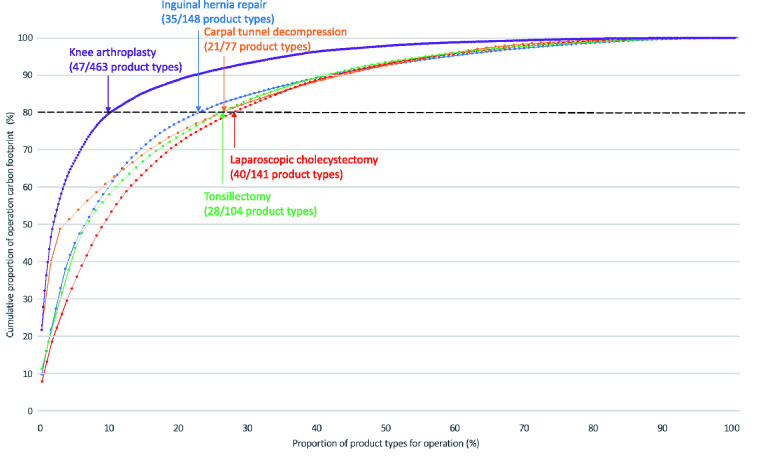
Proportion of products responsible for cumulative carbon footprint for each operation. Cumulative carbon footprint contribution and proportion of product types based on mean across all operations for each operation type. Each data point relates to a single-product type (e.g. suction receptacle). Arrows mark the point at which 80% of carbon footprint reached.

The carbon footprint of individual products used across operations is ranked in Supplementary Tables 23–27. Across the dataset, a small proportion of product types were responsible for ≥80% of carbon footprint of an operation (Figure 1 and Table 3): 21 out of 77 product types (27%) for carpal tunnel decompression; 35/148 (24%) for inguinal hernia repair; 47/463 (10%) for knee arthroplasty; 40/141 (28%) for laparoscopic cholecystectomy; and 28/104 (27%) for tonsillectomy. The mean average across operations was 23% of product types responsible for ≥80% carbon footprint.

**Table 3.** Product types responsible for the majority (≥80%) of the carbon footprint of each operation.

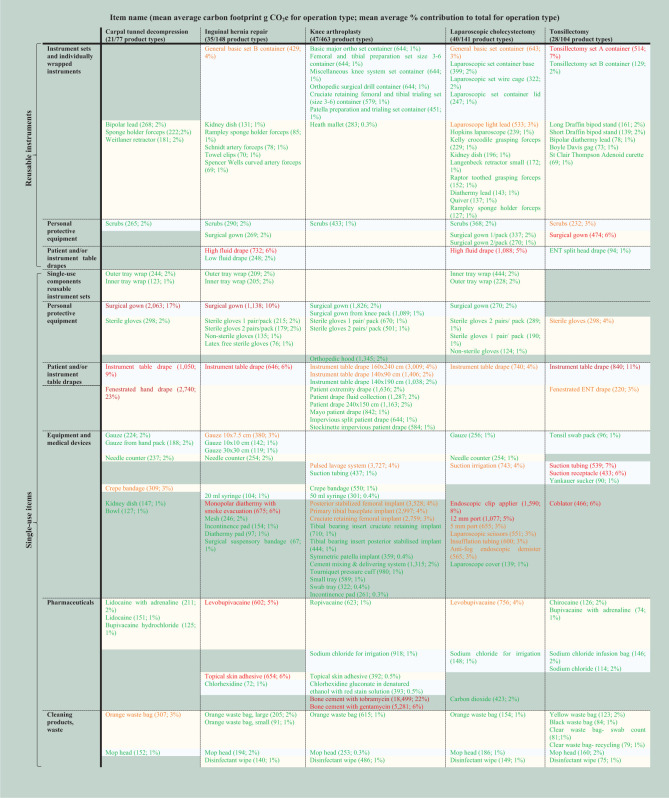					

Products listed are those responsible cumulatively for mean average ≥80% carbon footprint of each operation type. Mean average contribution of each product type across operations is specified in parenthesis (carbon footprint; percentage contribution), and will depend on the carbon footprint of the item, number of items used and number of operations in which item was used. Individual products contributing >10% carbon footprint are indicated in dark red, 5–10% in red, 3–5% orange, <1% green. Items are clustered in yellow or blue (alternating) where items are used across multiple operation types, and are ordered by magnitude of carbon footprint within clusters.

The three highest contributing products for carpal tunnel decompression were single-use fenestrated hand drape (mean average 23%), single-use surgical gowns (17%) and single-use instrument table drape (9%); for inguinal hernia repair: single-use surgical gowns (10%), reusable high fluid drape (6%) and single-use monopolar diathermy with smoke evacuation 6%); for knee arthroplasty: bone cement mix either with tobramycin or gentamicin (22% and 6%, respectively – the latter used for fewer operations) and single-use pulsed lavage system (4%); for laparoscopic cholecystectomy: single-use endoscopic clip applier (8%), reusable high fluid drape (5%) and single-use 12 mm port (5%); for tonsillectomy: single-use instrument table drape (11%), single-use suction tubing (7%) and reusable tonsillectomy set container (7%).

Table 4 shows the mean average contribution of processes to carbon footprint of the five operation types (individual operation results are provided in Supplementary Table 28), with highest contributions from the production of single-use products (54%), sterilisation (20%) and waste disposal of single-use products (8%), followed by production of packaging for single-use products (6%) and linen laundering (6%). There were small contributions from the production of single-use packaging for reusables (3%), production of reusables (2%) and waste disposal of reusables (1%).

**Table 4.** Carbon footprint of processes across life cycle of products used in all five operations.

Operation		Carbon footprint (g CO_2_e)								
		Reusable items					Single-use items			
		Production all reusables	Production single-use packaging for reusables	Decontamination	Linen laundering	Waste disposal all reusables	Production single-use items	Production packaging for single-use items	Waste disposal all single-use items	Total for operation
Carpal tunnel decompression	Mean ± sd	99 ± 25	309 ± 2	1531 ± 0	221 ± 41	82 ± 7	8041 ± 341	462 ± 40	1266 ± 61	**12,011 ± 412**
	Percentage of total	1%	3%	13%	2%	1%	67%	4%	11%	**100%**
	Range	51–119	305–310	1531–1531	194–310	73–87	7567–8805	385–515	1108–1324	**11,294–12,863**
Inguinal hernia repair	Mean ± sd	272 ± 81	572 ± 562	1995 ± 311	1174 ± 247	156 ± 118	5756 ± 1521	698 ± 73	1056 ± 279	**11,679 ± 1840**
	Percentage of total	2%	5%	17%	10%	1%	49%	6%	9%	**100%**
	Range	171–357	120–1293	1531–2299	881–1457	58–312	4492–8709	569–767	728–1442	**10,128–14,963**
Knee arthroplasty	Mean ± sd	539 ± 92	285 ± 50	13,965 ± 2029	362 ± 55	90 ± 15	59,115 ± 6490	6134 ± 695	4963 ± 168	**85,454 ± 7097**
	Percentage of total	1%	0.33%	16%	0.42%	0.11%	69%	7%	6%	**100%**
	Range	425–687	191–348	11,351–17,959	323–453	65–112	45,874–68,193	4608–6875	4663–5202	**72,247–94,875**
Laparoscopic cholecystectomy	Mean ± sd	458 ± 38	895 ± 78	5579 ± 75	1586 ± 150	224 ± 19	8859 ± 1032	1539 ± 120	1152 ± 326	**20,290 ± 1408**
	Percentage of total	2%	4%	27%	8%	1%	44%	8%	6%	**100%**
	Range	398–506	792–1007	5530–5676	1351–1777	199–246	7777–10,259	1338–1664	764–1597	**18,751–22,024**
Tonsillectomy	Mean ± sd	217 ± 19	125 ± 31	2154 ± 0	561 ± 127	26 ± 10	3245 ± 460	324 ± 143	823 ± 192	**7474 ± 734**
	Percentage of total	3%	2%	29%	8%	0.35%	43%	4%	11%	**100**
	Range	199–249	110–184	2154–2154	455–790	21–51	2508–3686	154–463	482–986	**6337–8170**
Percentage contribution of process category to total carbon footprint, mean average across all five operations		2%	3%	20%	6%	1%	54%	6%	8%	**100%**

Mean results across dataset – see Supplementary Table 28 for results by individual operation. CO_2_e: carbon dioxide equivalents; sd: standard deviation.

## Discussion

This is the first study to evaluate the carbon footprint of products used in common surgical operations. The mean average carbon footprint of products used for tonsillectomy was 7.5 kg CO_2_e, 11.7 kg CO_2_e for inguinal hernia repair, 12.0 kg CO_2_e for carpal tunnel decompression, 20.3 kg CO_2_e for laparoscopic cholecystectomy and 85.5 kg CO_2_e for knee arthroplasty. Previous systematic reviews^4,5^ of studies evaluating carbon footprint of single surgical operations found that this ranged from 6 kg CO_2_e (cataract surgery in India)^9^ to 814 kg CO_2_e (robotic hysterectomy, USA)^11^; however, the extent to which findings of studies can be compared is limited due to differences in methodological approaches, system boundaries, sources of emission factors, assumptions and allocation methods. We are aware of only one prior study evaluating an operation included in our analysis, which estimated carbon footprint of carpal tunnel decompression at 83 kg CO_2_e.^32^ However, that estimate included theatre energy consumption, decontamination and waste (and excluded product production), and the majority of carbon footprint related to decontamination (mean 61 kg CO_2_e/operation,^32^ compared with our own finding of 1.5 kg CO_2_e/operation). Here, one sterilisation cycle was estimated to use 864 kW for 1.2 h (1037 kWh),^32^ without a clear reason for the large discrepancy with our published data indicating a washer/disinfector uses 12.03 kWh/cycle and a steam steriliser uses 50.55 kWh/cycle.^28^

The choice of products used to perform a surgical operation is largely influenced by surgeons, and this study found, across observed operations, 54% of the total carbon footprint of products related to the production of single-use products. This was found to be major carbon hotspot in previous studies evaluating the carbon footprint of operations, including cataract surgery in France^33^ and New Zealand^34^ (production including manufacture and distribution of single-use products responsible for 73% and 77% of the total for operation, respectively), cardiac surgery in France (86%)^35^ coronary artery bypass graft in the United States (80%)^36^ and hysterectomy in the United States (60%),^11^ although the absolute proportions cited should be considered with caution, as different studies included different components of the operating theatre within their system boundary.

Across the five operations assessed, we found that relatively few products (mean average 23%) were associated with ≥80% of the carbon footprint of products used, aligning with the Pareto Principle (whereby 80% of an effect is associated with 20% inputs).^31^ We also found that production and disposal of single-use items, and associated packaging, were responsible for over two-thirds (Table 4, mean summed average 69%) of carbon footprint of products used across operations. Strategies to mitigate carbon footprint of common operations should include eliminating or finding low carbon alternatives for products with biggest contribution.

Strategies to eliminate products include avoiding non-sterile gloves, where they could be replaced with hand-washing, not opening gauze swab packs unless required, and asking suppliers to remove rarely used items from single-use pre-prepared packs. Different surgical approaches can also confer reductions, for example five of the tonsillectomies in this study were performed using steel dissection with diathermy and/or ties for haemostasis, and five used a single-use coblation^TM^ wand, associated with 0.93 kg CO_2_e/wand, opened in addition to the standard tonsillectomy set. We appreciate that there may be clinical reasons to prefer one technique over another, but are not aware of reasons why a coblation^TM^ wand cannot be manufactured for multiple use.

A review of 28 carbon footprint studies of products used in the operating theatre found this was lower for all reusable products when compared to single-use equivalents^5^ (aside from a study comparing a large number of reusable instruments used for spinal fusion to a heavily consolidated single-use set^37^ or a study from Australia where coal-based energy sources were modelled).^21^ Another review of healthcare products demonstrated that switching from single-use to reusable equivalents was associated with 61% mean average reductions in carbon footprint for protective equipment, 53% for invasive equipment and 36% for non-invasive equipment.^38^ A number of single-use high carbon products have reusable alternatives. For example, single-use gowns, patient drapes and instrument table drapes were high carbon contributors in this study; yet, a review found that reusable surgical textiles held significant reductions (200–300%) in carbon footprint,^12^ and there is no evidence that reusable textiles are clinically inferior.^8^ Single-use laparoscopic clip appliers, scissors and ports were responsible for 19% of the carbon footprint of laparoscopic cholecystectomy, but use of hybrid (predominantly reusable) equivalents could reduce their carbon footprint fourfold.^39^ There are also opportunities to switch many products in single-use pre-prepared packs for reusable equivalents. For example, we observed that the single-use hand-set (used for carpal tunnel decompression) contained a single-use kidney dish, bowl, light cover, patient drape and table drape, all of which could potentially be reusable, and the set also contained a sponge that was not used by any of the surgeons locally.

Such strategies should be supported by optimising approaches related to all stages of the life cycle of products. This includes manufacture: for example, titanium femoral knee implants manufactured using electron beam melting were found to have a fourfold lower carbon footprint compared with conventional manufacturing using milling.^40^ In the current study, decontamination of reusable instruments was responsible for 21% of carbon footprint, and we previously reported that this could be optimised through processing instruments in sets rather than individually, maximal loading of decontamination machines, using low-carbon energy sources and recycling sterile barrier systems.^28^ Once a reusable item reaches the end of its apparent lifespan, there may be further opportunities for repair, which have been modelled to reduce the carbon footprint of reusable scissors by 20%.^41^ Waste constituted 8% of carbon, and in our setting, we found infectious waste (569 kg CO_2_e generated per tonne disposed) or clinical waste (1,074 kg CO_2_e/tonne)^27^ streams were predominantly used, even though non-infectious offensive waste streams would have been appropriate,^42^ and could halve or quarter carbon footprint of waste (240 kg CO_2_e/tonne).^27^ Further strategies to reduce the carbon footprint of surgical waste may include ensuring appropriate bins are available in theatres, improving waste segregation (industry may facilitate this through labelling and reducing the number of different materials), including supporting recycling and recyclability through manufacturer and supplier labelling and prudent use of materials.^43^ Approaches to optimise the carbon footprint of healthcare laundering (responsible for 6%) have not previously been evaluated, and is an important area for future research.

Table 5 models the impact of combining some of these strategies for the operations observed in this study, identifying and modelling areas for reduction of products used, opting for reusable equivalent products (where these are already available in the market), and optimising decontamination and waste. We estimate that these strategies combined could lead to a theoretical 32% carbon reductions across the five operations (38% carpal tunnel decompression, 28% inguinal hernia repair, 23% knee arthroplasty, 31% laparoscopic cholecystectomy, 42% tonsillectomy), which, if realised across England,^19^ would save over 2600 tonnes CO_2_e per year. We are aware that this may be difficult to achieve in practice, because for example 100% recycling or optimal loading of decontamination machines cannot always be achieved, but exemplifies the scale of change that may be possible. We are also cognisant that innovation may allow this figure to be surpassed, for example through more reusable products becoming available. It is likely that such strategies will also be associated with financial savings. For example, switching from single-use to reusable laryngoscopes was found to save 59%–87% cost for blades and 77%–95% for handles,^44^ and switching from single-use laparoscopic equipment to hybrid was associated with halving of costs.^39^ Furthermore, some recycling do not charge for waste collection, reducing disposal costs.^45^

**Table 5.** Example strategies for mitigating the carbon footprint of operations.

	*Reduction in carbon footprint (g CO_2_e per operation) relative to mean average (% reduction)* Strategy for mitigation, reductions				
Operation *(Baseline mean carbon footprint g CO_2_e)*	Reduce/rationalise	Switch single-use products to reusable equivalents	Optimise decontamination	Optimise waste	Total
Carpal tunnel decompression(12,011)	*41 (0.3%)*• Rationalise non-sterile gloves (modelled on lowest observed number)• Remove unused foam cube from pre-prepared set	*2539 (21%)*• Bowl• Kidney dish• Light handle• Nail scrubbing brush• Surgical face mask• Patient drape• Surgical gown• Surgical hat• Table drape	*643 (5%)*• Optimise loading of decontamination machines• Switch reusable containers to tray wrap	*1349 (11%)*• Maximal recycling	*4571 (38%)*
Inguinal hernia repair(11,679)	*47 (0.4%)*• Rationalise non-sterile gloves (modelled on lowest observed number)	*793 (7%)*• Absorbent towel pack• Diathermy tip• Kidney dish• Light handle• Patient drape• Nail scrubbing brush• Surgical face mask• Surgical gown• Surgical hat• Table drape	*1177 (10%)*• Optimise loading of decontamination machines• Switch reusable containers to tray wrap• Where two general basic sets observed, opting for lower carbon option• Integrating individually wrapped instruments into sets○ Roberts artery forceps○ Collingwood Stewart hernia forceps	*1212 (10%)*• Maximal recycling	*3229 (28%)*
Knee arthroplasty(85,454)	*230 (3%)*• Rationalise non-sterile gloves (modelled on lowest observed number)• Avoid skin stapler	*8343 (10%)*• Adhesive operative towel• Bowls• Kidney dish• Light cover• Monopolar diathermy• Monopolar diathermy• Nail scrubbing brush• Orthopaedic hood• Patient drapes• Suction receptacle• Suction tip• Surgical face mask• Surgical gown• Surgical hat• Swab trays • Table drapes• Tourniquet pressure cuff• Tray (small) • Yankauer sucker	*6438 (8%)*• Optimise loading of decontamination machines• Switch reusable containers to tray wrap• Integrating individually wrapped instruments into sets○ Bipolar diathermy○ Blunt Hohman bone elevator○ Diathermy extras○ Diathermy lead○ Lanes tissue forceps○ Light cover○ Light handle○ Non-toothed lamina spreaders○ Semb bone holding forceps	*5053 (6%)*• Maximal recycling	*20,064 (23%)*
Laparoscopic cholecystectomy(20,290)	*64 (0.3%)*• Rationalise non-sterile gloves (modelled on lowest observed number)	*2418 (12%)*• Absorbent towel pack• Diathermy pad lead• Endoscopic clip applier^a^• Laparoscopic scissors^a^• Light handle• Nail scrubbing brush• Ports^a^• Suction receptacle• Surgical face mask• Surgical gown• Surgical hat• Table drape	*2527 (12%)*• Optimise loading of decontamination machines• Switch reusable containers to tray wrap• Integrating individually wrapped instruments into sets○ Diathermy lead○ Laparoscopic grasping forceps○ Quiver and clip	*1375 (7%)*• Maximal recycling	*6384 (31%)*
Tonsillectomy(7474)	*485 (6%)*• Rationalise non-sterile gloves (modelled on lowest observed number)• Avoid Coblation^TM^ wand	*887 (12%)*• Fenestrated ENT drape• Gallipot• Kidney dish• Nail scrubbing brush• Suction tip• Surgical face mask• Surgical hat• Table drape• Suction receptacle• Yankauer sucker	*921 (12%)*• Where two tonsillectomy sets observed, opting for lower carbon option• Optimise loading of decontamination machines• Switch reusable containers to tray wrap	*849 (11%)*• Maximal recycling	*3143 (42%)*

Reductions in carbon footprint (relative to mean average across observed operations for given type), modelling impact of switching from single-use to reusable products using mean average reductions derived in previous systematic review (61% reduction for protective equipment, 53% for invasive equipment and 36% for non-invasive equipment),^38^ impact of optimising decontamination based on previous study by the authors,^28^ and assuming all waste was recycled, with GHG emissions associated with recycling process assigned to the production of the recycled goods, in line with the recycled content method.^18^

^a^Predominantly reusable.

A hybrid approach was used in this study, using a process-based approach wherever possible, and an EEIO model applied where there was a lack of available emission factors (for a small number of pharmaceuticals and chemicals). Combining such methods resulted in the system boundaries differing between included products (Supplementary Figure 1), limiting the extent to which products derived through different approaches can be compared. The impact on results will be small for most operations evaluated, as the components modelled using an EEIO approach had a small mean average contribution (2% of total carbon footprint results for carpal tunnel decompression, 8% inguinal hernia repair, 4% laparoscopic cholecystectomy, 4% tonsillectomy). However, for knee arthroplasty, 31% of the carbon footprint of all products was determined through the EEIO method, of which bone cement was responsible for 91% of this, estimated at 13 kg CO_2_e per packet of bone cement, of which one or two packets were used per operation (in two and eight cases, respectively). Given this large contribution, we decided to model this using a process-based approach based on component materials with available emission factors (Supplementary Table 29, but factors were not available for the components N,n-dimethyl-para- toluidine, hydroquinone, gentamicin sulphate, dibenzoyl peroxide and tobramycin sulphate). This reduced the carbon footprint of bone cement with gentamicin and tobramycin by 93%–95% to 683 g CO_2_e–947 g CO_2_e per packet respectively. The large discrepancy in results derived through EEIO and process-based approaches for bone cement highlights the need for the academic community to develop emission factors for pharmaceutical products.

### Limitations

This study is limited by the validity of emission factors in databases reporting national or global average emissions of materials and inputs, and differences in system boundaries between these sources. The impact of this was limited through using a consistent database within a given study, and only using alternative sources where a given material or process was not available in the primary database. Use of such databases was a pragmatic decision as it was not feasible to obtain primary data upstream of the hospital study site. Use of an EEIO model to determine carbon footprint of certain pharmaceuticals and chemicals is a further limitation, with impact discussed. A previous review of national health system carbon footprints highlighted the challenge posed by lack of available data relating to healthcare products, alongside a lack of relevant emission factors in particular for pharmaceutical products.^46^ Our findings should be used with caution to compare individual products, and instead dedicated full life cycle assessments should be conducted of specific products, including detailed primary data across the product life cycle and evaluating a range of environmental impacts.

Our findings may not be generalisable to other contexts, for example to UK surgeons practising alternative approaches or techniques in surgery, and to other countries such as the USA where single-use products are widely used in surgery, or to low-income countries where reuse of products is the norm. In common with other carbon footprinting studies, the number of operations observed was relatively small and undertaken at a single site, and the carbon footprint of operations will vary by setting, patient variables and surgical or anaesthetic technique.

We recognise that to instigate change towards reduced and reuse of equipment in the operating room will require leadership and behaviour change from surgical teams and their representative bodies, alongside changes in infrastructure and funding to ensure that reusable equipment is the financially and logistically preferable option. It will also need a more refined approach to estimating risk of infection from reuse of equipment which in many cases is based upon hypothetical rather than real-world risk.^8^

## Conclusion

We found that production of single-use items, decontamination of reusable instruments and waste disposal were the largest contributors to the carbon footprint of products used across five common operations. Relatively few products (23%) were responsible for ≥80% of the carbon footprint, and so efforts should be targeted in particular towards these products, through eliminating single-use items or switching to reusables where feasible, alongside optimising associated decontamination processes and waste segregation and recycling, which could reduce the carbon footprint by one-third.

## Supplemental Material

sj-pdf-1-jrs-10.1177_01410768231166135 - Supplemental material for The carbon footprint of products used in five common surgical operations: identifying contributing products and processes Click here for additional data file. Supplemental material, sj-pdf-1-jrs-10.1177_01410768231166135 for The carbon footprint of products used in five common surgical operations: identifying contributing products and processes by Chantelle Rizan, Robert Lillywhite, Malcom Reed and Mahmood F Bhutta in Journal of the Royal Society of Medicine

## Declarations

### Guarantor

CR.

### Provenance

Not commissioned; peer reviewed by Qing-Wei Chin and Julie Morris.

### ORCID iDs

Chantelle Rizan https://orcid.org/0000-0002-9518-2948

## References

[1] Karliner J Slotterback S Boyd R Ashby B Steele K. Health care's climate footprint, Climate-smart health care series green paper number one. Health Care without Harm, September 2019. See https://noharm-global.org/sites/default/files/documents-files/5961/HealthCaresClimateFootprint_092319.pdf (last checked 9 August 2022).

[2] Tennison I Roschnik S Ashby B Boyd R Hamilton I Oreszczyn T , et al. Health care's response to climate change: a carbon footprint assessment of the NHS in England. Lancet Planeta Health 2021; 5: e84–e92. 10.1016/S2542-5196(20)30271-0 PMC7887664 33581070

[3] World Health Organization. COP26 Health Programme Country Commitments. World Health Organization, 2021. See www.who.int/initiatives/cop26-health-programme/country-commitments (last checked 9 August 2022).

[4] Rizan C Steinbach I Nicholson R Lillywhite R Reed M Bhutta M. The carbon footprint of operating theatres: a systematic review. Ann Surg 2020; 272: 986–995. 32516230 10.1097/SLA.0000000000003951

[5] Drew J Christie SD Tyedmers P Smith-Forrester J Rainham D. Operating in a climate crisis: a state-of-the-science review of life cycle assessment within surgical and anesthetic care. Environ Health Perspect 2021; 129: 76001. 34251875 10.1289/EHP8666 PMC8274692

[6] MacNeill AJ Lillywhite R Brown CJ. The impact of surgery on global climate: a carbon footprinting study of operating theatres in three health systems. Lancet Planet Health 2017; 1: e381–e388. 29851650 10.1016/S2542-5196(17)30162-6

[7] White SM Shelton CL Gelb AW Lawson C McGain F Muret J , et al. Principles of environmentally-sustainable anaesthesia: a global consensus statement from the World Federation of Societies of Anaesthesiologists. Anaesthesia 2022; 77: 201–212. 34724710 10.1111/anae.15598 PMC9298028

[8] Bhutta MF. Our over-reliance on single-use equipment in the operating theatre is misguided, irrational and harming our planet. Ann R Coll Surg Engl 2021; 103: 709–712. 34719955 10.1308/rcsann.2021.0297 PMC10335235

[9] Thiel CL Schehlein E Ravilla T Ravindran RD Robin AL Saeedi OJ , et al. Cataract surgery and environmental sustainability: waste and lifecycle assessment of phacoemulsification at a private healthcare facility. J Cataract Refract Surg 2017; 43: 1391–1398. 29223227 10.1016/j.jcrs.2017.08.017 PMC5728421

[10] Campion N Thiel CL DeBlois J Woods NC Landis AE Bilec MM. Life cycle assessment perspectives on delivering an infant in the US. Sci Total Environ 2012; 425: 191–198. 22482785 10.1016/j.scitotenv.2012.03.006 PMC3563327

[11] Thiel CL Eckelman M Guido R Huddleston M Landis AE Sherman J , et al. Environmental impacts of surgical procedures: life cycle assessment of hysterectomy in the United States. Environ Sci Technol 2015; 49: 1779–1786. 25517602 10.1021/es504719g PMC4319686

[12] Overcash M. A comparison of reusable and disposable perioperative textiles: sustainability state-of-the-art 2012. Anesth Analg 2012; 114: 1055–1066. 22492184 10.1213/ANE.0b013e31824d9cc3

[13] Ibbotson S Dettmer T Kara S Herrmann C. Eco-efficiency of disposable and reusable surgical instruments – a scissors case. Int J Life Cycle Assess 2013; 18: 1137–1148.

[14] McPherson B Sharip M Grimmond T. The impact on life cycle carbon footprint of converting from disposable to reusable sharps containers in a large US hospital geographically distant from manufacturing and processing facilities. PeerJ 2019; 7: e6204. 30809428 10.7717/peerj.6204 PMC6388662

[15] Rizan C Bhutta MF. Strategy for net-zero carbon surgery. Br J Surg 2021; 108: 737–739. 33963828 10.1093/bjs/znab130

[16] Harris H Bhutta MF Rizan C. A survey of UK and Irish surgeons' attitudes, behaviours and barriers to change for environmental sustainability. Ann R Coll Surg Engl 2021; 103: 725–729. 34719956 10.1308/rcsann.2021.0271 PMC10335270

[17] Penny T Fisher K Collins M Allison C. Greenhouse Gas Accounting Sector Guidance for Pharmaceutical Products and Medical Devices. London, UK: Environmental Resources Management, 2012.

[18] Bhatia P Cummis C Brown A Draucker L Rich D Lahd H . Greenhouse Gas Protocol, Product Life Cycle Accounting and Reporting Standard. USA: Institute of World Resources, 2011.

[19] NHS Digital. Hospital Admitted Patient Care Activity, 2017–18. UK: NHS Digital, September 2018. See https://digital.nhs.uk/data-and-information/publications/statistical/hospital-admitted-patient-care-activity/2017-18 (last checked 9 August 2022).

[20] Royal College of Surgeons of England. Surgical Specialties. London, UK: Royal College of Surgeons of England, 2022. See www.rcseng.ac.uk/careers-in-surgery/trainees/foundation-and-core-trainees/surgical-specialties/ (last checked 9 August 2022).

[21] McGain F McAlister S McGavin A Story D. A life cycle assessment of reusable and single-use central venous catheter insertion kits. Anesth Analg 2012; 114: 1073–1080. 22492185 10.1213/ANE.0b013e31824e9b69

[22] Jones C Hammond G. Inventory of Carbon and Energy v3.0* [database]*. Bath, UK: Circular Ecology, University of Bath, 2019.

[23] *Department for Environment, Food and Rural Affairs/Department for Business* *, Energy & Industrial Strategy. UK Government GHG Conversion Factors for Company Reporting* [database]. London, UK: Department for Environment, Food and Rural Affairs/Department for Business, Energy & Industrial Strategy, 2021.

[24] PRé Sustainability. SimaPro Version 9.10, Ecoinvent (version 3.6) [database]. Amersfort, Netherlands: PRé Sustainability, 2019.

[25] Parvatker AG Tunceroglu H Sherman JD Coish P Anastas P Zimmerman JB Eckelman MJ. Cradle-to-gate greenhouse gas emissions for twenty anesthetic active pharmaceutical ingredients based on process scale-up and process design calculations. ACS Sustain Chem Eng 2019; 7: 6580–6591.

[26] Small World Consulting. Carbon Factors Dataset version 5.3 [database]. Lancaster, UK: Small World Consulting, Lancaster University, 2020.

[27] Rizan C Bhutta MF Reed M Lillywhite R. The carbon footprint of waste streams in a UK hospital. J Clean Product 2021; 286: 125446.

[28] Rizan C Lillywhite R Reed M Bhutta M. Minimising carbon footprint and financial costs of steam sterilization and packaging reusable surgical instruments. Br J Surg 2022; 109: 200–210. 34849606 10.1093/bjs/znab406 PMC10364739

[29] Vozzola E Overcash M Griffing E. Environmental considerations in the selection of isolation gowns: a life cycle assessment of reusable and disposable alternatives. Am J Infect Control 2018; 46: 881–886. 29655666 10.1016/j.ajic.2018.02.002

[30] Carre A. Life Cycle Assessment Comparing Laundered Surgical Gowns with Polypropylene Based Disposable Gowns. Melbourne, Australia: RMIT University, 2008.

[31] Koch R. The 80/20 Principle. Finland: Nicholas Brealey Publishing, 2007.

[32] Zhang D Dyer GSM Blazar P Earp BE. The environmental impact of open versus endoscopic carpal tunnel release. J Hand Surg Am 2022; 48: 46–52. 35123818 10.1016/j.jhsa.2021.12.003

[33] Ferrero A Thouvenin R Hoogewoud F Marcireau I Offret O Louison P , et al. The carbon footprint of cataract surgery in a French university hospital. J Fr Ophtalmol 2022; 45: 57–64. 34823888 10.1016/j.jfo.2021.08.004

[34] Latta M Shaw C Gale J. The carbon footprint of cataract surgery in Wellington. N Z Med J 2021; 134: 13–21. 34531593

[35] Grinberg D Buzzi R Pozzi M Schweizer R Capsal JF Thinot B , et al. Eco-audit of conventional heart surgery procedures. Eur J Cardiothorac Surg 2021; 60: 1325–1331. 34411226 10.1093/ejcts/ezab320

[36] Hubert J Gonzalez-Ciccarelli LF Wang AW Toledo E Ferrufino R Smalls K , et al. Carbon emissions during elective coronary artery bypass surgery, a single center experience. J Clin Anesth 2022; 80: 110850. 35525051 10.1016/j.jclinane.2022.110850

[37] Leiden A Cerdas F Noriega D Beyerlein J Herrmann C. Life cycle assessment of a disposable and a reusable surgery instrument set for spinal fusion surgeries. Resour Conserv Recycl 2020; 156: 104704.

[38] Keil M Viere T Helms K Rogowski W. The impact of switching from single-use to reusable healthcare products: a transparency checklist and systematic review of life-cycle assessments. Eur J Public Health 2023; 33: 56–63. 36433787 10.1093/eurpub/ckac174 PMC9898010

[39] Rizan C Bhutta MF. Environmental impact and life cycle financial cost of hybrid (reusable/single-use) instruments versus single-use equivalents in laparoscopic cholecystectomy. Surg Endosc 2022; 36: 4067–4078. 34559257 10.1007/s00464-021-08728-z PMC9085686

[40] Lyons R Newell A Ghadimi P Papakostas N. Environmental impacts of conventional and additive manufacturing for the production of Ti-6Al-4V knee implant: a life cycle approach. Int J Adv Manuf Technol 2021; 112: 787–801.

[41] Rizan C Brophy T Lillywhite R Reed M Bhutta M. Life cycle assessment and life cycle cost of repairing surgical scissors. Int J Life Cycle Assess 2022; 27: 780–795.

[42] Department of Health. Health Technical Memorandum (HTM) 01-01 on the management and decontamination of surgical instruments (medical devices) used in acute care. London, UK: Department of Health, March 2013. See https://assets.publishing.service.gov.uk/government/uploads/system/uploads/attachment_data/file/536144/HTM0101PartA.pdf (last checked 9 August 2022).

[43] Shoham MA Baker NM Peterson ME Fox P. The environmental impact of surgery: a systematic review. Surgery 2022; 172: 897–905. 35788282 10.1016/j.surg.2022.04.010

[44] Sherman JD Raibley LA Eckelman MJ. Life cycle assessment and costing methods for device procurement: comparing reusable and single-use disposable laryngoscopes. Anesth Analg 2018; 127: 434–443. 29324492 10.1213/ANE.0000000000002683

[45] McGain F Clark M Williams T Wardlaw T. Recycling plastics from the operating suite. Anaesth Intensive Care 2008; 36: 913–914. 19115664

[46] Booth A. Carbon footprint modelling of national health systems: opportunities, challenges and recommendations. Int J Health Plann Manage 2022; 37: 1885–1893. 35212060 10.1002/hpm.3447 PMC9541808

