# Supplementary material for: The carbon footprint of products used in five common surgical operations: identifying contributing products and processes
Source: J R Soc Med. 2023 Apr 13;116(6):199–213. doi: 10.1177/01410768231166135 (PMC10331364; doi:10.1177/01410768231166135)
Supplement: sj-pdf-1-jrs-10.1177_01410768231166135 - Supplemental material for The carbon footprint of products used in five common surgical operations: identifying contributing products and processes [file sj-pdf-1-jrs-10.1177_01410768231166135.pdf]

Supplementary methods 1: Linen laundering emission factor 2

Supplementary figure 1: System boundary. 4
Supplementary figure 2: Carbon footprint of operations, broken down by underpinning life cycle processes 5

Supplementary Table 1: Emissions factors 6
Supplementary Table 2: Carbon footprint of reusable instrument set used for carpal tunnel decompression 8
Supplementary Table 3: Carbon footprint of reusable (non-set) and single-use items used for carpal tunnel decompression 10
Supplementary Table 4: Carbon footprint of reusable instrument sets used for inguinal hernia repair 18
Supplementary Table 5: Carbon footprint of reusable (non-set) and single-use items used for inguinal hernia repair 24
Supplementary Table 6: Carbon footprint of reusable instrument sets used for knee arthroplasty 36
Supplementary Table 7: Carbon footprint of reusable (non-set) and single-use items used for knee arthroplasty 48
Supplementary Table 8: Carbon footprint of reusable instrument sets used for laparoscopic cholecystectomy 69
Supplementary Table 9: Carbon footprint of reusable (non-set) and single-use items used for laparoscopic cholecystectomy 75
Supplementary Table 10: Carbon footprint of reusable instrument sets used for tonsillectomy 88
Supplementary Table 11: Carbon footprint of reusable (non-set) and single-use items used for tonsillectomy 92
Supplementary Table 12: Items and waste streams used for carpal tunnel decompression operation 1-10 99
Supplementary Table 13: Items and waste streams used for inguinal hernia repair operation 1-6 101
Supplementary Table 14: Items and waste streams used for knee arthroplasty 1-10 103
Supplementary Table 15: Items and waste streams used for laparoscopic cholecystectomy operation 1-6 109
Supplementary Table 16: Items and waste streams used for tonsillectomy operation 1-10 112
Supplementary Table 17: Carbon footprint of carpal tunnel decompression operations impact assessment 114
Supplementary Table 18: Carbon footprint of inguinal hernia repair operations impact assessment 118
Supplementary Table 19: Carbon footprint of knee arthroplasty operations impact assessment 122
Supplementary Table 20: Carbon footprint of laparoscopic cholecystectomy operations impact assessment 131
Supplementary Table 21: Carbon footprint of tonsillectomy operations impact assessment 136
Supplementary Table 22: Carbon footprint of products used in operations by product category. 140
Supplementary Table 23: Mean average carbon footprint of products used for carpal tunnel decompression 142
Supplementary Table 24: Mean average carbon footprint of products used for inguinal hernia repair 144
Supplementary Table 25: Mean average carbon footprint of products used for knee arthroplasty 148
Supplementary Table 26: Mean average carbon footprint of products used for laparoscopic cholecystectomy 158
Supplementary Table 27: Mean average carbon footprint of products used for tonsillectomy 162
Supplementary table 28 Product types responsible for majority (≥80%) of carbon footprint of each operation. 165
Supplementary Table 29: Carbon footprint of processes across life cycle of products used in all five operations 168
Supplementary table 30: Carbon footprint of bone cement using process-based approach 170

Supplementary methods 1: Linen laundering emission factor

This supplementary information estimates the carbon footprint of laundering one kg of reusable surgical linens such as the reusable surgical gowns and drapes used within the study site operating theatres. These were made from microfibre polyester (according to manufacturer information). The energy and water requirements of healthcare laundry were determined using data reported within a Life Cycle Assessment study by Vozzola et al. which examined reusable isolation gowns made from woven polyester.(1) This was based on a 2016 survey by the International Association of Healthcare Textile Managers, which surveyed nineteen healthcare laundry companies (thirteen in the USA and six in Canada), and these are likely to use similar processes to UK sites. Unlike other LCAs identified in a literature review of reusable versus single-use perioperative textiles,(2) the study by Vozzola et al.(1) distinguished between water supplied and water treated (estimating the water lost due to evaporation and incorporation into products). There is a lack of evidence on emission factors relating to detergents, and this component of linen laundering was not included in the study by Vozzola et al.(1) A report published by RMIT University(3) identified that detergents were responsible for 2.5% of the carbon footprint of laundering surgical gowns (including energy and water use, and excluding transport), and an uplift factor was applied to the relevant sub-total here. The carbon footprint of transportation was also included, based on the journey from the principal study site (Royal Sussex County Hospital) to the linen laundering facility (Elis, Camberwell). The carbon footprint was determined using emission factors outlined in Supplementary Information Table 1.

Supplementary Information Table 1: Emission factors used for linen laundering. CV=calorific value, tonne.km= one tonne of transported goods over one km. Note 2019 Department for Environment, Food and Rural Affairs (DEFRA)/ Department for Business, Energy & Industrial Strategy (BEIS) emission factors were used here and have since been updated, but using 2021 emission factors makes <1% difference to the emission factor derived here for linen laundering.

Process/ product		Emission factor		Emission factor unit	Source
		Component	Total		
Natural Gas	Combustion	0.18	0.21	kg CO_2e/ kWh (Gross CV)	DEFRA/BEIS(4)
	Well to tank	0.02			
HGV (diesel, average HGV), average laden		0.11	0.11	kg CO_2e/ tonne.km	DEFRA/BEIS(4)
UK electricity (aggregated)		0.32	0.32	kg CO_2e/ kWh	DEFRA/BEIS(4)
Water supply		0.34	0.34	kg CO_2e/ m^3	DEFRA/BEIS(4)
Water treatment		0.71	0.71	kg CO_2e/ m^3	DEFRA/BEIS(4)

The carbon footprint of linen laundering was estimated to be 0.46 kg CO_2e/ kg dry linen (Supplementary information table 2). This value is similar to that reported by the European

Textile Services Association which estimates that the carbon footprint of hotel linen laundering was 0.43 kg CO_2e/ kg.(5)

Supplementary Information Table 2: Carbon footprint of laundering of reusable healthcare linen. RSCH= Royal Sussex County Hospital

**Input**	**Source**	**Quantity used per kg of dry linen**	**Carbon footprint (kg CO_2e/ kg dry linen)**	
			Component	Total
Natural gas	Vozzola et al.(1)	1.59 kWh	0.331	0.461
Electricity	Vozzola et al.(1)	0.28 kWh	0.089	
Water supply	Vozzola et al.(1)	$0.011m^3$	0.004	
Water treatment	Vozzola et al.(1)	$0.011m^3$	0.008	
Detergent	RMIT(3)	N/A	0.011	
Travel	RSCH to Elis round trip	161km	0.018	

Supplementary figure 1: System boundary.
DEFRA/BEIS= Department of Environment Food and Rural Affairs/ Department for Business, Energy and Industrial Strategy, EEIO= environmentally extended input-output, ICE= Inventory of Carbon and Energy.

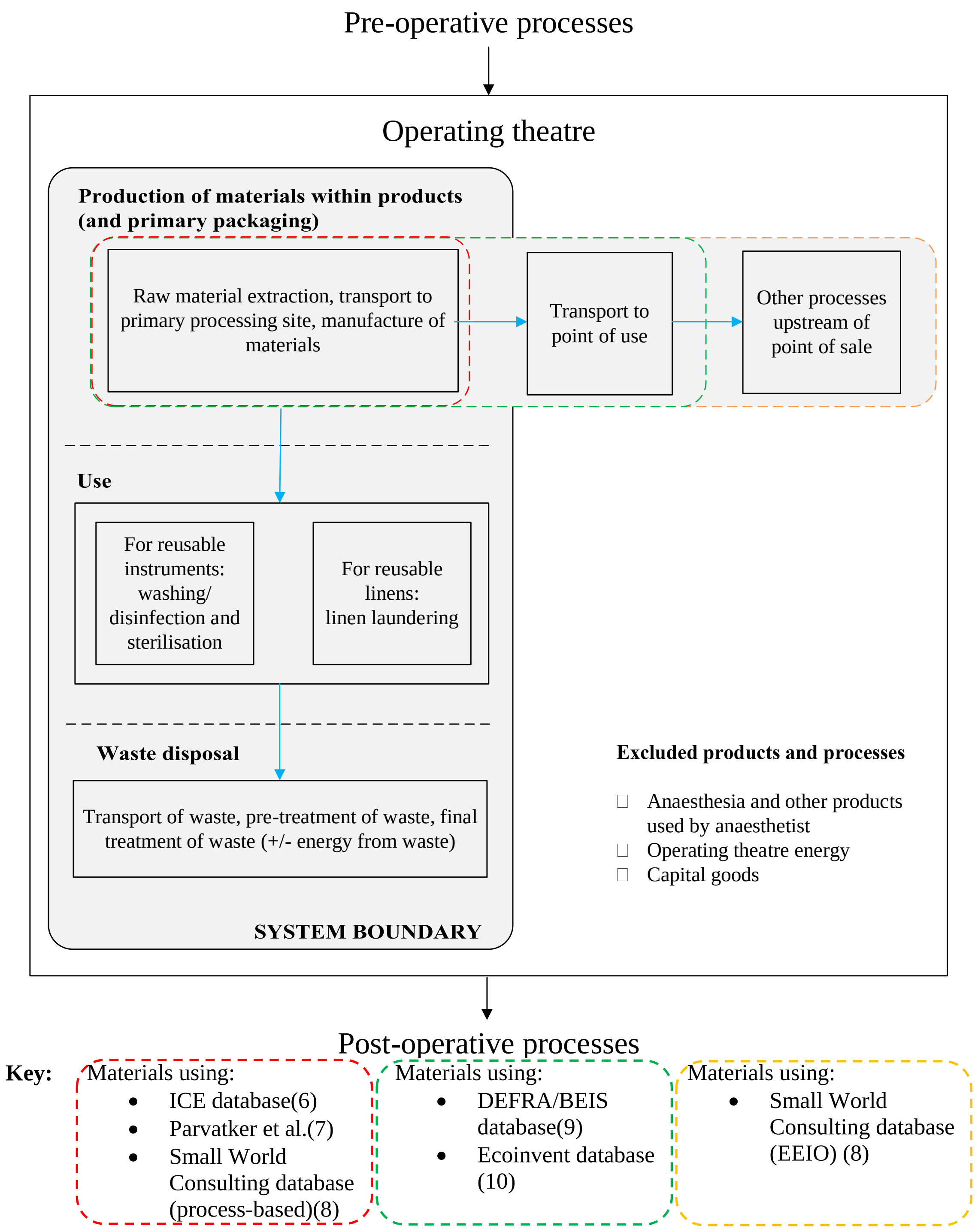

Supplementary figure 2: Carbon footprint of operations, broken down by underpinning life cycle processes

Based on mean average across all operations for each operation type. CO_2e= carbon dioxide equivalents.

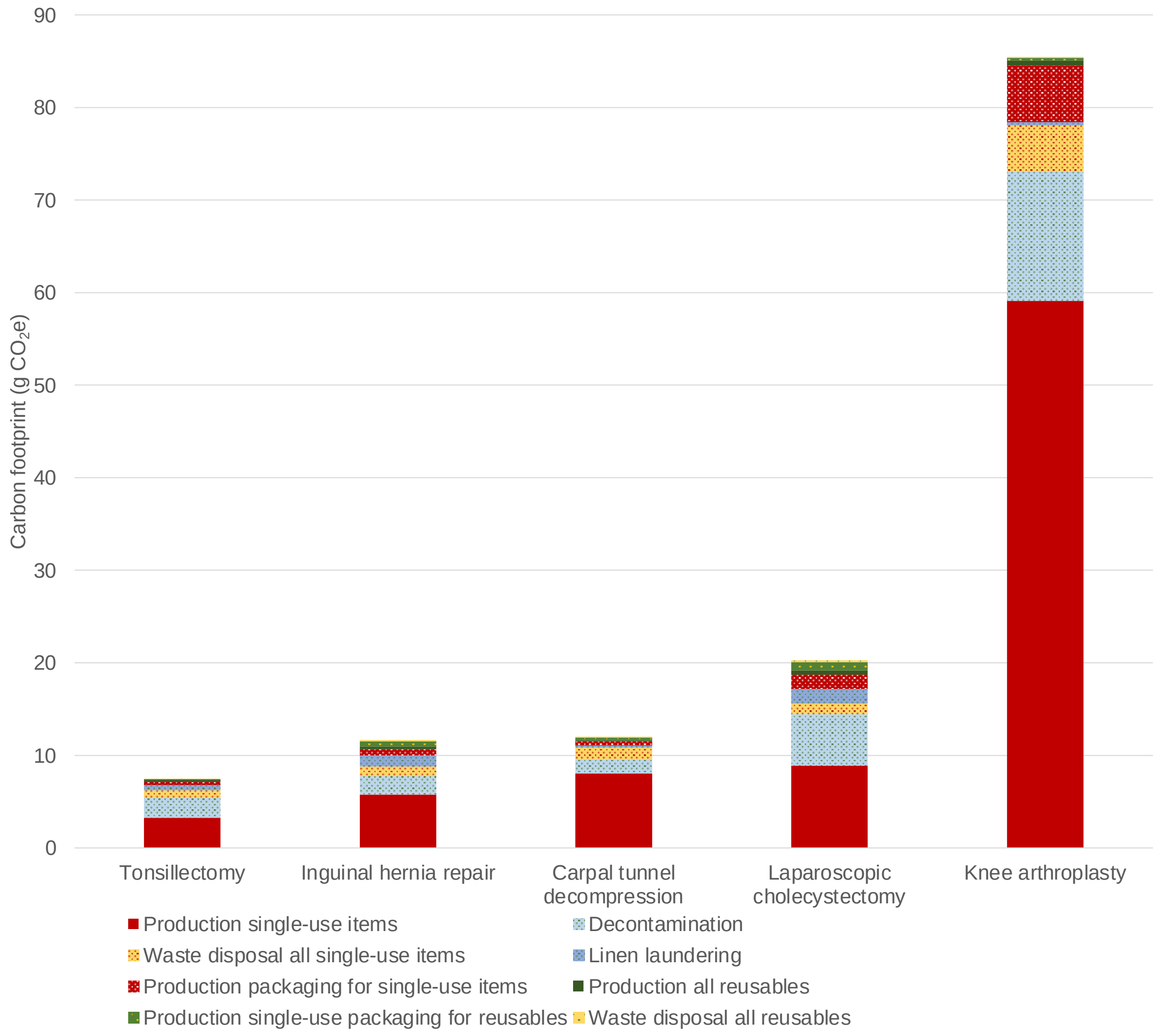

Supplementary Table 1: Emissions factors

CO_2e= carbon dioxide equivalents *Activities include transportation from the site of primary processing to the point of use (equating to the manufacturer of multi-component items). **Where financial spend was used, this encompassed all activities from raw material extraction to the point of sale. All other material/pharmaceutical emission factors constitute ‘cradle to factory gate’ emissions, including raw material extraction, transportation to primary processing site, primary processing and manufacturing through to factory gate.

Material/ process	Emission factor	Emission factor unit	Source
**Material**			
Acrylonitrile Butadiene Styrene (ABS)	3.76	kg CO_2e/ kg	ICE v3(6)
Aluminium cast	6.72	kg CO_2e/ kg	
Aluminium foil	7.47	kg CO_2e/ kg	
Brass	4.80	kg CO_2e/ kg	
Copper	3.81	kg CO_2e/ kg	
Cotton fabric	6.78	kg CO_2e/ kg	
Cotton padding	1.28	kg CO_2e/ kg	
Expanded polystyrene	3.29	kg CO_2e/ kg	
General plastics	3.31	kg CO_2e/ kg	
General polyethylene	2.54	kg CO_2e/ kg	
General purpose polystyrene	3.43	kg CO_2e/ kg	
Glass general	1.44	kg CO_2e/ kg	
Glass reinforced plastic	8.10	kg CO_2e/ kg	
High density polyethylene resin	1.93	kg CO_2e/ kg	
Lithium-ion battery*	6.31	kg CO_2e/ kg	DEFRA/BEIS(9)
Low density polyethylene film	2.60	kg CO_2e/ kg	ICE v3(6)
Low density polyethylene resin	2.08	kg CO_2e/ kg	
Nickel	12.40	kg CO_2e/ kg	
Non-woven polyester*	5.56	kg CO_2e/ kg	Ecoinvent v3.6(10) using 'Non-woven polyester {GLO}| market for textile, non woven polyester| Cut-off,U'
Nylon (polyamide) 6 polymer	9.14	kg CO_2e/ kg	ICE v3(6)
Paper	1.49	kg CO_2e/ kg	
Petroleum slack wax*	1.08	kg CO_2e/ kg	Ecoinvent v3.6(10)
Polycarbonate	7.62	kg CO_2e/ kg	ICE v3(6)
Polyethylene terephthalate*	4.03	kg CO_2e/ kg	DEFRA/BEIS(9)
Polypropylene injection moulding	4.49	kg CO_2e/ kg	ICE v3(6)
Polypropylene oriented film	3.43	kg CO_2e/ kg	
Polyurethane flexible foam	4.84	kg CO_2e/ kg	
Polyurethane rigid foam	4.26	kg CO_2e/ kg	
Polyvinylchloride general	3.10	kg CO_2e/ kg	
Polyvinylchloride injection moulding	3.30	kg CO_2e/ kg	
Rubber	2.85	kg CO_2e/ kg	
Silicone*	3.34	kg CO_2e/ kg	Ecoinvent v3.6(10) using 'Silicone product {RoW}| market for silicone product| Cut-off,U'
Silk*	36.14	kg CO_2e/ kg	Ecoinvent v3.6(10) using 'Yarn, silk {GLO}| market for yarn, silk| Cut-off,U'
Stainless steel	6.15	kg CO_2e/ kg	Small World Consulting v5.3(8)
Titanium	20.60	kg CO_2e/ kg	ICE v3(6)
Zinc	4.18	kg CO_2e/ kg	

**Pharmaceuticals and cleaning chemicals**			
Manufacture of basic pharmaceutical products and pharmaceutical preparations**	0.192	kg CO_2e/ £	Small World Consulting v5.3(8)
Manufacture of cleaning preparations**	0.169	kg CO_2e/ £	
Adrenaline	34	kg CO_2e/ kg	Parvatker et al.(7)
Bupivacaine hydrochloride	23	kg CO_2e/ kg	
Carbon dioxide liquid*	0.0063	kg CO_2e/ L	Ecoinvent v3.6(10) using 'Carbon dioxide, liquid{ROW}| market for| Cut-off,U' (assumes 1kg liquid CO_2e=150 L liquid CO_2e based upon product labelling)
Levobupivacaine	23	kg CO_2e/ kg	Parvatker et al.(7)
Lidocaine	29	kg CO_2e/ kg	
Ropivacaine hydrochloride	36	kg CO_2e/ kg	
**Instrument decontamination and linen laundering**			
Additional washing of standard sized reusable container (sterile barrier system)	0.623	kg CO_2e/ reusable container	Rizan et al. (2022)(11)
Additional washing of small sized reusable container (sterile barrier system)	0.312	kg CO_2e/ reusable container	
Linen laundering	0.461	kg CO_2e/ kg	Supplementary information (linen laundering)
Reusable instrument decontamination (steam sterilisation)- standard sized set	1.531	kg CO_2e/ standard sized set	Rizan et al. (2022)(11)
Reusable instrument decontamination (steam sterilisation)- small sized set	0.996	kg CO_2e/ small sized set	
Reusable instrument steam decontamination- supplementary item	0.145	kg CO_2e/ supplementary item	
**Waste**			
Anatomical waste	1074	kg CO_2e/ tonne	Rizan et al. (2021) (12)
Clinical waste	1074	kg CO_2e/ tonne	
Domestic waste	172	kg CO_2e/ tonne	
Infectious waste	569	kg CO_2e/ tonne	
Non-infectious offensive waste	249	kg CO_2e/ tonne	
Medicinal contaminated sharps waste	1074	kg CO_2e/ tonne	
Recycling (scrap metal, clothing, batteries)	21	kg CO_2e/ tonne	

Supplementary Table 2: Carbon footprint of reusable instrument set used for carpal tunnel decompression

Table reports carbon footprint of minor op set used for carpal tunnel decompression (only one set used across all observed operations) The material composition of items was determined through packaging and manufacturer information where available, and alternatively through expert assessment, taking into account available emission factors. Weight is total for a given item (accounting for multiple items where relevant). ‘Use’ equates to decontamination of instruments, determined per set. CO_2e= carbon dioxide equivalents

Category	Item	Material(s)	Weight (g)	Number of uses	Waste stream A	Carbon footprint per use ($g\ CO_2e$)		
						Production	Use	Waste stream A
**Minor op set**								
**Sterile barrier system**	Identification tag	High density polyethylene resin	11.87	46	Non-infectious offensive waste	0.50	See total	0.0642
	Kit list	Paper	4.85	1	Infectious waste	7.23	N/A	2.7612
	Metal tray (small)	Stainless steel	362.62	1,554	Scrap metal recycling	1.43	See total	0.0050
	Pin mat	Silicone	170.76	1,036	Non-infectious offensive waste	0.55		0.0410
	Tray wrap (inner, 90x90 cm)	Paper	59.5	1	Infectious waste	88.66	N/A	33.87
	Tray wrap (outer, 90x90 cm)	Polypropylene oriented film	61.02	1	Infectious waste	209.30		34.74
**Instruments and set items**	Adson dissecting toothed forceps	Stainless steel	20.20	1,036	Scrap metal recycling	0.12	See total	0.0004
	Bipolar diathermy forceps (small)	Stainless steel	15.26	40	Medicinal contaminated sharps waste	2.34		0.4096
		Nylon (polyamide) 6 polymer	1.70			0.39		0.0457
	Bipolar lead	Polyvinylchloride general	80.96	50	Non-infectious offensive waste	5.02		0.4031
		Copper	9.00			0.69		0.0448
	BP scalpel handle x2	Stainless steel	24.42	518	Scrap metal recycling	0.29		0.0010
	Crile wood needle holder	Stainless steel	33.30	1,036	Scrap metal recycling	0.20		0.0007

	Gillies skin hook x2	Stainless steel	14.15	1,813	Scrap metal recycling	0.05		0.0002
	Kilner cats paw retractor x2	Stainless steel	18.23	1,813	Scrap metal recycling	0.06		0.0002
	McDonald dissector	Stainless steel	23.53	1,036	Scrap metal recycling	0.14		0.0005
	Mosquito artery forceps (curved) x2	Stainless steel	18.69	2,849	Scrap metal recycling	0.04		0.0001
	Sponge holder forceps x2	Stainless steel	76.16	2,331	Scrap metal recycling	0.20		0.0007
	Stevens tenotomy scissor	Stainless steel	20.69	518	Medicinal contaminated sharps waste	0.25		0.0429
		Low density polyethylene resin	0.42			0.002		0.0009
	Suture scissor	Stainless steel	35.20	518	Scrap metal recycling	0.42		0.0014
	Towel clip x2	Stainless steel	30.65	1,554	Scrap metal recycling	0.12		0.0004
	Weitlaner retractor (3x4 teeth)	Stainless steel	62.24	1,295	Scrap metal recycling	0.30		0.0010
	Weitlaner retractor (2x3 teeth)	Stainless steel	41.56	1,295	Scrap metal recycling	0.20		0.0007
					**Total minor op set=1,922.18 g CO_2e**	**318.48**	**1,531.27**	**72.44**

Supplementary Table 3: Carbon footprint of reusable (non-set) and single-use items used for carpal tunnel decompression

Table includes all single-use items used across all observed operations listed here and the carbon footprint per item as listed. Items used for individual operations varied, alongside number of items used. The material composition of items was determined through packaging and manufacturer information where available, and alternatively through expert assessment, taking into account available emission factors. Weight is total for a given item (accounting for multiple items where relevant). 'Use' equates to linen laundering of reusable linens. All waste of given item disposed of using consistent waste stream (A). Carbon footprint per use equates to a single use of an item in a single operation. CO_2e= carbon dioxide equivalents. Ops.= operations

Item	Component	Material(s)	Weight (g), or cost where specified with (£)	Number of uses	Number of ops per use	Waste stream A	Carbon footprint per use (g CO_2e)			
							Production	Use	Waste stream A	Total (waste A)
**Reusable personal protective equipment**										
Reusable scrubs	Product	Cotton fabric	421.17	75	5	Clothing recycling	7.61	38.79	0.02	46.43
**Non-set reusable equipment**										
Reusable tourniquet pressure cuff	Product	Polyvinylchloride general	82.89	6	1	Infectious waste	42.82	N/A	7.86	50.69
		High density polyethylene resin	54.85				17.64	N/A	5.20	22.85
	Packaging	Low density polyethylene film	11.19				4.85	N/A	1.06	5.91
**Single-use patient and instrument table drapes from 'Hand pack' set**										
Patient drape (fenestrated hand drape)	Product	Polypropylene oriented film	676.08	1	1	Infectious waste	2,318.95	N/A	384.90	2,703.86
		Rubber	10.55				30.07	N/A	6.01	36.07
Table drape (instruments)	Product	Polyvinylchloride general	123.17	1	1	Infectious waste	381.83	N/A	70.12	451.95

		Nylon (polyamide) 6 polymer	61.58				562.84	N/A	35.06	597.90
‘Hand pack’ packaging (drape component)	Packaging	Low density polyethylene film	25.09	1	1	Infectious waste	65.24	N/A	14.29	79.53
		Paper	5.70				8.50	N/A	3.25	11.75
**Single-use equipment from ‘Hand pack’ set**										
Bowl	Product	Polypropylene injection moulding	12.58	1	1	Infectious waste	56.46	N/A	7.16	63.62
Foam cube	Product	Polyurethane flexible foam	1.71	1	1	Infectious waste	8.28	N/A	0.97	9.25
Gauze swab	Product	Cotton fabric	25.63	1	1	Infectious waste	173.77	N/A	14.59	188.36
Kidney dish	Product	Polypropylene injection moulding	29.07	1	1	Infectious waste	130.52	N/A	16.55	147.07
Light cover	Product	Low density polyethylene film	4.78	1	1	Infectious waste	12.43	N/A	2.72	15.15
Needle counter	Product	Acrylonitrile Butadiene Styrene	44.50	1	1	Medicinal contaminated sharps waste	167.32	N/A	47.80	215.12
		Polyurethane rigid foam	4.07				17.34	N/A	4.37	21.71
Surgical blade	Product	Stainless steel	0.39	1	1	Medicinal contaminated sharps waste	2.40	N/A	0.42	2.82
	Packaging	Aluminium foil	0.52				3.88	N/A	0.56	4.44
Syringe (20 ml)	Product	High density polyethylene resin	10.66	1	1	Infectious waste	20.57	N/A	6.07	26.64
‘Hand pack’ packaging (single-use equipment component)	Packaging	Low density polyethylene film	3.86	1	1	Infectious waste	10.03	N/A	2.20	12.22
		Paper	0.88				1.31	N/A	0.50	1.80
**Single-use personal protective equipment**										
Gloves (non-sterile, pair)	Product	Rubber	6.45	1	1	Infectious waste	18.38	N/A	3.67	22.05
Sterile gloves (pair)	Product	Rubber	27.31	1	1	Infectious waste	77.83	N/A	15.55	93.38

	Packaging	General polyethylene	5.98	1	1		15.19	N/A	3.40	18.59
		Paper	5.91				8.81	N/A	3.36	12.17
Sterile gloves (two pairs)	Product	Rubber	42.14	1	1	Infectious waste	120.10	N/A	23.99	144.09
	Packaging	Paper	11.61				17.30	N/A	6.61	23.91
		General polyethylene	5.61				14.25	N/A	3.19	17.44
Sterile gloves (two pair, latex free)	Product	General polyethylene	44.68	1	1	Infectious waste	113.49	N/A	25.44	138.92
	Packaging	Paper	11.66				17.37	N/A	6.64	24.01
		General polyethylene	5.71				14.50	N/A	3.25	17.75
Surgical face mask	Product	Polypropylene oriented film	4.36	1	1	Infectious waste	14.95	N/A	2.48	17.44
Surgical face mask with eye protection	Product	Polypropylene oriented film	6.23	1	1	Infectious waste	21.37	N/A	3.55	24.92
		Low density polyethylene film	4.04				10.50	N/A	2.30	12.80
Surgical gown (including hand towels)	Product	Polypropylene oriented film	139.00	1	1	Infectious waste	476.78	N/A	79.14	555.92
		Rubber	7.32				20.85	N/A	4.17	25.02
		Paper	13.83				20.61	N/A	7.87	28.48
	Packaging	Paper	19.30				28.76	N/A	10.99	39.74
		Low density polyethylene film	5.11				13.29	N/A	2.91	16.20
Surgical hat	Product	Polypropylene oriented film	3.54	1	4	Infectious waste	3.04	N/A	0.50	3.54
Visor	Product	Low density polyethylene resin	8.54	1	1	Infectious waste	17.76	N/A	4.86	22.63
		Low density polyethylene film	2.39				6.21	N/A	1.36	7.57
	Packaging	Low density polyethylene film	1.84				4.78	N/A	1.05	5.83

		Paper	1.43				2.13	N/A	0.81	2.94
		Nylon (polyamide) 6 polymer	0.44				4.02	N/A	0.25	4.27
**Single-use equipment and medical devices**										
Crepe bandage (7.5 cm wide)	Product	Cotton fabric	38.69	1	1	Infectious waste	262.32	N/A	22.03	284.34
	Packaging	Paper	7.09				10.56	N/A	4.04	14.60
		Low density polyethylene film	3.14				8.16	N/A	1.79	9.95
Elasticated fabric dressing strip	Product	Rubber	0.65	1	1	Infectious waste	1.85	N/A	0.37	2.22
Gauze (individual piece)	Product	Cotton fabric	5.06	1	1	Infectious waste	34.31	N/A	2.88	37.19
Gauze (sterile pack)	Product	Cotton fabric	25.52	1	1	Infectious waste	173.03	N/A	14.53	187.55
	Packaging	Paper	5.12				7.63	N/A	2.91	10.54
		Low density polyethylene film	1.63				4.24	N/A	0.93	5.17
Incontinence pad	Product	Low density polyethylene film	30.18	1	1	Infectious waste	78.47	N/A	17.18	95.65
Nail scrubbing brush	Product	Low density polyethylene resin	9.01	1	1	Infectious waste	18.74	N/A	5.13	23.87
		Polyurethane flexible foam	2.05				9.92	N/A	1.17	11.09
		Polypropylene injection moulding	0.91				4.09	N/A	0.52	4.60
	Packaging	Low density polyethylene film	1.31				3.41	N/A	0.75	4.15
		Paper	0.60				0.89	N/A	0.34	1.24
Needle (blue)	Product	Stainless steel	0.18	1	1	Medicinal contaminated sharps waste	1.13	N/A	0.20	1.33
		Polypropylene injection moulding	0.04				0.20	N/A	0.05	0.24

	Packaging	High density polyethylene resin	0.40			Infectious waste	0.77	N/A	0.23	1.00
		Low density polyethylene film	0.12				0.31	N/A	0.07	0.38
		Paper	0.11				0.16	N/A	0.06	0.23
Needle (green)	Product	Stainless steel	0.20	1	1	Medicinal contaminated sharps waste	1.23	N/A	0.21	1.44
		Polypropylene injection moulding	0.05				0.22	N/A	0.05	0.28
	Packaging	High density polyethylene resin	0.52			Infectious waste	1.00	N/A	0.30	1.30
		Low density polyethylene film	0.12				0.31	N/A	0.07	0.38
		Paper	0.11				0.16	N/A	0.06	0.23
Needle (red)	Product	Stainless steel	0.41	1	1	Medicinal contaminated sharps waste	2.51	N/A	0.44	2.95
		Polypropylene injection moulding	0.10				0.46	N/A	0.11	0.57
	Packaging	High density polyethylene resin	0.60			Infectious waste	1.16	N/A	0.34	1.50
		Low density polyethylene film	0.12				0.31	N/A	0.07	0.38
		Paper	0.11				0.16	N/A	0.06	0.23
Non-woven dressing	Product	Nylon (polyamide) 6 polymer	0.58	1	1	Infectious waste	5.30	N/A	0.33	5.63
	Packaging	Paper	1.22				1.82	N/A	0.69	2.51
		Low density polyethylene film	0.64				1.66	N/A	0.36	2.03
Skin marker	Product	High density polyethylene resin	6.23	1	1	Infectious waste	12.02	N/A	3.55	15.57
		Paper	0.57				0.85	N/A	0.32	1.17
	Packaging	Paper	1.82				1.36	N/A	0.52	1.87
		Low density polyethylene film	0.91				29.48	N/A	6.46	35.94

Stockinette tubular bandage	Product	Cotton fabric	11.34	1	1	Infectious waste	76.89	N/A	6.46	83.34
Surgical blade	Product	Stainless steel	0.39	1	1	Medicinal contaminated sharps waste	2.40	N/A	0.42	2.82
	Packaging	Aluminium foil	0.52			Infectious waste	3.88	N/A	0.30	4.18
Suture (monofilament, non-absorbable, 4-0)	Product	Stainless steel	0.01	1	1	Medicinal contaminated sharps waste	0.06	N/A	0.01	0.07
		Nylon (polyamide) 6 polymer	0.00				0.00	N/A	0.00	0.00
	Packaging	Paper	2.82			Infectious waste	4.20	N/A	1.61	5.81
		Low density polyethylene film	0.49				1.27	N/A	0.28	1.55
Suture (monofilament, non-absorbable 5-0)	Product	Stainless steel	0.01	1	1	Medicinal contaminated sharps waste	0.06	N/A	0.01	0.07
		Nylon (polyamide) 6 polymer	0.00				0.00	N/A	0.00	0.00
	Packaging	Paper	2.82			Infectious waste	4.20	N/A	1.61	5.81
		Low density polyethylene film	0.49				1.27	N/A	0.28	1.55
Syringe (10 ml)	Product	Polypropylene injection moulding	3.01	1	1	Infectious waste	13.51	N/A	1.71	15.23
		General polyethylene	3.01				7.65	N/A	1.71	9.36
		Rubber	0.25				0.71	N/A	0.14	0.85
	Packaging	Low density polyethylene film	0.45				1.17	N/A	0.26	1.43
		Paper	0.39				0.58	N/A	0.22	0.80
Syringe (20 ml)	Product	Polypropylene injection moulding	7.10	1	1	Infectious waste	31.90	N/A	4.04	35.94
		General polyethylene	7.10				18.04	N/A	4.04	22.09
		Rubber	0.59				1.69	N/A	0.34	2.02

	Packaging	Low density polyethylene film	1.11				2.89	N/A	0.63	3.52
		Paper	0.72				1.07	N/A	0.41	1.48
Tape (clear)	Product	Nylon (polyamide) 6 polymer	0.51	1	1	Infectious waste	4.66	N/A	0.29	4.95
Undercast padding	Product	Cotton padding	17.63	1	1	Infectious waste	22.57	N/A	10.04	32.60
	Packaging	Paper	6.89				10.27	N/A	3.92	14.19
		Low density polyethylene film	1.65				4.29	N/A	0.94	5.23
**Pharmaceuticals**										
Bupivacaine hydrochloride 0.5% (10 ml)	Product	Bupivacaine hydrochloride	10.00	1	1	N/A	230.00	N/A	N/A	230.00
	Packaging	Low density polyethylene film	3.71			Infectious waste	9.65	N/A	2.11	11.76
		Low density polyethylene resin	2.66				5.53	N/A	1.51	7.05
		Paper	0.55				0.82	N/A	0.31	1.13
Chlorhexidine 2% from 500 ml container (100 ml)	Product and packaging	Chlorhexidine 2%	£0.56	1	1	N/A	107.30	N/A	N/A	107.30
Hyaluronidase 1500 I.U. (10 ml)	Product and packaging	Hyaluronidase	£16.19	1	8	Infectious waste	387.75	N/A	N/A	387.75
	Packaging (weight for waste)		1.48				N/A	N/A	0.11	N/A
Levobupivacaine 5mg/10 ml (10 ml)	Product	Levobupivacaine	10.00	1	1	N/A	230.00	N/A	N/A	230.00
	Packaging	Low density polyethylene resin	2.65			Infectious waste	5.51	N/A	1.51	7.02
		Low density polyethylene film	0.46				1.20	N/A	0.26	1.46
		Paper	0.40				0.60	N/A	0.23	0.82
Lidocaine 1% (10 ml)	Product	Lidocaine	10.00	1	1	N/A	290.00	N/A	N/A	290.00

	Packaging	Low density polyethylene resin	4.41	1	1	Infectious waste	9.17	N/A	2.51	11.68
Lidocaine 1% with adrenaline 1:200,000 (20 ml)	Product	Lidocaine	20.00	1	1	N/A	580.00	N/A	N/A	580.00
	Packaging	Low density polyethylene film	33.22			Medicinal contaminated sharps waste	86.37	N/A	35.68	122.05
Sodium Chloride 0.9% from 1 litre bottle (100 ml)	Product and packaging	Sodium chloride	£0.40	1	1	N/A	75.87	N/A	N/A	75.87
**Cleaning products, waste**										
Clear bin bag (linen laundering)	Product	Low density polyethylene film	13.91	1	5	Domestic waste	7.23	N/A	0.48	7.71
		Nylon (polyamide) 6 polymer	2.58				4.72	N/A	0.09	4.80
Disinfectant sachet	Product and packaging	Disinfectant	£0.70	1	5	Infectious waste	26.82	N/A	N/A	26.82
	Packaging (weight for waste)		2.58				N/A	N/A	0.66	N/A
Disinfectant wipe	Product	Non-woven polyester	2.30	1	1	Infectious waste	12.79	N/A	1.31	14.10
Orange waste bag (infectious waste) with cable tie from theatre	Product	Polypropylene oriented film	70.61	1	1	Infectious waste	242.19	N/A	40.20	282.39
		Nylon (polyamide) 6 polymer	2.58				23.58	N/A	1.47	25.05
Orange waste bag (infectious waste) with cable tie from scrub room	Product	Polypropylene oriented film	70.61	1	5	Infectious waste	48.44	N/A	8.04	56.48
		Nylon (polyamide) 6 polymer	2.58				4.72	N/A	0.29	5.01
Mop head	Product	Nylon (polyamide) 6 polymer	70.36	1	5	Infectious waste	128.62	N/A	8.01	136.63
		High density polyethylene resin	30.16				11.64	N/A	3.43	15.08
Red bag (linen laundering) with cable tie	Product	Polypropylene oriented film	29.22	1	5	Domestic waste	20.04	N/A	1.00	21.05
		Nylon (polyamide) 6 polymer	2.58				4.72	N/A	0.09	4.80

Supplementary Table 4: Carbon footprint of reusable instrument sets used for inguinal hernia repair

Table includes all reusable instrument sets and individually wrapped instruments across all observed operations, and the carbon footprint per set. Sets and individually wrapped instruments used for individual operations varied. The material composition of items was determined through packaging and manufacturer information where available, and alternatively through expert assessment, taking into account available emission factors. Weight is total for a given item (accounting for multiple items where relevant). Some items listed as 'bottom tray'/'top tray' where not individually counted by theatre staff. 'Use' equates to decontamination of instruments, determined per set. All waste of given item disposed of using consistent waste stream (A). CO_2e= carbon dioxide equivalents

**Category**	**Item**	**Material(s)**	**Weight (g)**	**Number of uses**	**Waste stream A**	**Carbon footprint per use (g CO_2e)**		
						**Production**	**Use**	**Waste stream A**
			**General basic set (A)**					
**Sterile barrier system**	Identification tag	High density polyethylene resin	12.13	46	Non-infectious offensive waste	0.51	See total	0.0656
	Kit list	Paper	4.85	1	Infectious waste	7.23	N/A	2.7612
	Metal tray (medium)	Stainless steel	1147.39	1,554	Scrap metal recycling	4.54	See total	0.0157
	Tray lining (60x60 cm)	Paper	21.11	1	Infectious waste	31.45	N/A	12.02
	Tray wrap (inner, 150x180 cm)	Polypropylene oriented film	154.12	1	Infectious waste	528.63		87.74
	Tray wrap (outer, 150x180 cm)	Polypropylene oriented film	156.5	1	Infectious waste	536.80		89.10
**Instruments and set items**	Allis tissue forceps x2	Stainless steel	62.30	4,476	Scrap metal recycling	0.09	See total	0.0003
	Babcock forceps x2	Stainless steel	47.18	4,476	Scrap metal recycling	0.06		0.0002
	Baby Mixter forceps	Stainless steel	25.60	4,476	Scrap metal recycling	0.04		0.0001
	BP scalpel handle (no. 3) x2	Stainless steel	51.56	895	Scrap metal recycling	0.35		0.0012
	BP scalpel handle (no. 4)	Stainless steel	27.78	895	Scrap metal recycling	0.19		0.0007
	Crile baby needle holder	Stainless steel	36.64	1,791	Scrap metal recycling	0.13		0.0004

	Czerny retractor x2	Stainless steel	103.32	5,372	Scrap metal recycling	0.12		0.0004
	Debakey plan dissecting forceps x2	Stainless steel	50.08	1,343	Scrap metal recycling	0.23		0.0008
	Diathermy lead	Polyvinylchloride general	86.52	50	Non-infectious offensive waste	5.36		0.4307
		Copper	9.61			0.73		0.0478
	Diathermy quiver	Polypropylene injection moulding	95.15	1,343	Non-infectious offensive waste	0.32		0.0176
	Dunhill artery forceps x5	Stainless steel	111.00	4,476	Scrap metal recycling	0.15		0.0005
	Gallipot x2	Polypropylene injection moulding	8.06	1	Non-infectious offensive waste	36.19	N/A	2.0064
	Gilles toothed dissecting forceps x2	Stainless steel	46.74	1,074	Scrap metal recycling	0.27	See total	0.0009
	Hernia ring	Stainless steel	37.62	2,523	Scrap metal recycling	0.09		0.0003
	Kidney dish (20 cm)	Polypropylene injection moulding	50.22	895	Non-infectious offensive waste	0.25		0.0140
	Lahey cholecystectomy forceps	Stainless steel	45.99	2,523	Scrap metal recycling	0.11		0.0004
	Lanes tissue forceps x2	Stainless steel	93.16	4,476	Scrap metal recycling	0.13		0.0004
	Langenbeck medium retractor x2	Stainless steel	159.40	6,267	Scrap metal recycling	0.16		0.0005
	Langenbeck small retractor x2	Stainless steel	119.12	6,267	Scrap metal recycling	0.12		0.0004
	Mayo curved scissor	Stainless steel	70.08	895	Scrap metal recycling	0.48		0.0017
	Mayo Hegar needle holder	Stainless steel	38.65	1,791	Scrap metal recycling	0.13		0.0005
	Mayo straight scissor	Stainless steel	64.64	1,343	Scrap metal recycling	0.30		0.0010
	McIndoe curved scissor	Stainless steel	35.61	895	Scrap metal recycling	0.24		0.0008
	McIndoe plain diathermy forceps	Stainless steel	38.30	40	Medicinal contaminated sharps waste	5.88		1.0283
		Nylon (polyamide) 6 polymer	4.26			0.97		0.1143

	Morris retractor	Stainless steel	102.06	5,372	Scrap metal recycling	0.12		0.0004
	Mosquito fine artery forceps x5	Stainless steel	102.80	2,686	Scrap metal recycling	0.24		0.0008
	Paper (90x90 cm)	Paper	49.00	1	Infectious waste	73.01	N/A	27.8963
	Pulp tray (no.3)	Paper	12.13	1	Infectious waste	18.07		6.9058
	Spencer Wells curved artery forceps x2	Stainless steel	109.86	4,476	Scrap metal recycling	0.15	See total	0.0005
	Sponge holder forceps x4	Stainless steel	294.92	4,029	Scrap metal recycling	0.45		0.0016
	Towel clip x5	Stainless steel	162.15	895	Scrap metal recycling	1.11		0.0039
	Travers self-retaining retractor	Stainless steel	121.73	1,970	Scrap metal recycling	0.38		0.0013
**Total general basic set (A)= 3,017.22g CO_2e**						**1,255.77**	**1,531.27**	**230.18**
**General basic set (B)**								
**Sterile barrier system**	Basket	Stainless steel	1170.57	116	Scrap metal recycling	62.01	See total	0.2149
	Container	Aluminium cast	2996.54	1,000	Scrap metal recycling	20.14		0.0638
	Filter paper	Paper	3.55	1	Infectious waste	5.29	N/A	2.0211
	Identification tag	High density polyethylene resin	8.21	46	Non-infectious offensive waste	0.34	See total	0.0444
	Kit list	Paper	4.85	1	Infectious waste	7.23	N/A	2.7612
	Tamper proof tags	General plastics	1.78	1	Infectious waste	5.89		1.0134
**Instruments and set items**	Allis tissue forceps x2	Stainless steel	56.92	4,476	Scrap metal recycling	0.08	See total	0.0003
	Babcock tissue forceps x2	Stainless steel	48.50	4,476	Scrap metal recycling	0.07		0.0002
	Bag plain closure (H)	Paper	13.69	1	Infectious waste	20.40	N/A	7.7939
	Bipolar diathermy	Stainless steel	31.98	40		4.91	See total	0.8588

					Medicinal contaminated sharps waste			
		High density polyethylene resin	31.98			1.54		0.8588
	Bonney toothed dissecting forceps	Stainless steel	61.07	4,029	Scrap metal recycling	0.09		0.0003
	BP scalpel handle (no. 3) x2	Stainless steel	49.40	895	Scrap metal recycling	0.34		0.0012
	BP scalpel handle (no. 4) x2	Stainless steel	57.44	895	Scrap metal recycling	0.39		0.0014
	Bulldog clip	Stainless steel	49.77	1,343	Scrap metal recycling	0.23		0.0008
	Czerny retractor x2	Stainless steel	123.94	5,372	Scrap metal recycling	0.14		0.0005
	Debakey dissecting forceps	Stainless steel	21.42	1,343	Scrap metal recycling	0.10		0.0003
	Diathermy lead	Polyvinylchloride general	86.52	50	Non-infectious offensive waste	5.36		0.4307
		Copper	9.61			0.73		0.0478
	Diathermy quiver	Polypropylene injection moulding	96.13	1,343	Non-infectious offensive waste	0.32		0.0178
	Gallipot x3	Polypropylene injection moulding	14.10	1	Infectious waste	63.31	N/A	8.0273
	Gillies toothed dissecting forceps	Stainless steel	24.31	1,074	Scrap metal recycling	0.14	See total	0.0005
	Halstead mosquito curved artery forceps x6	Stainless steel	122.76	2,686	Scrap metal recycling	0.28		0.0010
	Kidney dish (25 cm) x4	Polypropylene injection moulding	395.52	895	Non-infectious offensive waste	1.98		0.1100
	Lanes tissue forceps x2	Stainless steel	84.32	4,476	Scrap metal recycling	0.12		0.0004
	Langenbeck retractor (medium) x2	Stainless steel	105.10	6,267	Scrap metal recycling	0.10		0.0004
	Mayo curved scissors	Stainless steel	70.12	895	Scrap metal recycling	0.48		0.0017
	Mayo Hegar needle holder x2	Stainless steel	58.24	1,791	Scrap metal recycling	0.20		0.0007
	Mayo straight scissors	Stainless steel	44.34	1,343	Scrap metal recycling	0.20		0.0007

	McIndoe dissecting forceps	Stainless steel	20.69	2,238	Scrap metal recycling	0.06		0.0002
	McIndoe plain diathermy forceps	Stainless steel	32.49	40	Medicinal contaminated sharps waste	4.99		0.8725
		Nylon (polyamide) 6 polymer	3.61			0.82		0.0969
	McIndoe scissors	Stainless steel	34.01	895	Scrap metal recycling	0.23		0.0008
	Plain dissecting forceps (5")	Stainless steel	23.09	40	Scrap metal recycling	3.55		0.0123
	Plain dissecting forceps (7")	Stainless steel	37.01	895	Scrap metal recycling	0.25		0.0009
	Rampley sponge holder forceps x4	Stainless steel	256.56	1,343	Scrap metal recycling	1.17		0.0041
	Schnidt artery forceps x6	Stainless steel	238.56	4,029	Scrap metal recycling	0.36		0.0013
	Spencer Wells curved artery forceps x4	Stainless steel	209.52	3,581	Scrap metal recycling	0.36		0.0012
	Spencer Wells straight artery forceps x6	Stainless steel	146.46	4,476	Scrap metal recycling	0.20		0.0007
	Stitch scissor	Stainless steel	32.74	4,476	Scrap metal recycling	0.04		0.0002
	Towel clip x6	Stainless steel	211.86	1,343	Scrap metal recycling	0.97		0.0034
	Travers self retaining retractor	Stainless steel	107.66	895	Scrap metal recycling	0.74		0.0026
	Treves toothed dissecting forceps x6	Stainless steel	141.12	1,970	Scrap metal recycling	0.44		0.0015
**Total general basic set (B)= 2,395.86g CO_2e**						**216.62**	**2,153.96**	**25.27**
**Roberts artery forceps**								
**Sterile barrier system**	Flexible pouch	General polyethylene	11.14	1	Infectious waste	28.30	N/A	6.3421
		Paper	8.06			12.01		4.5887
	Identification tag	High density polyethylene resin	8.21	46	Non-infectious offensive waste	0.34	See total	0.0444

**Instruments and set items**	Roberts artery forceps	Stainless steel	42.46	4,476	Scrap metal recycling	0.06		0.0002
**Total Roberts artery forceps= 197.07 g CO_2e**						**40.71**	**145.38**	**10.98**
**Collingwood Stewart hernia forceps**								
**Sterile barrier system**	Flexible pouch	General polyethylene	11.14	1	Infectious waste	28.30	N/A	6.3421
		Paper	8.06			12.01		4.5887
	Identification tag	High density polyethylene resin	8.21	46	Non-infectious offensive waste	0.34	See total	0.0444
**Instruments and set items**	Collingwood Stewart hernia forceps	Stainless steel	38.57	2,523	Scrap metal recycling	0.09		0.0003
**Total Collingwood Stewart hernia forceps= 197.10 g CO_2e**						**40.74**	**145.38**	**10.98**

Supplementary Table 5: Carbon footprint of reusable (non-set) and single-use items used for inguinal hernia repair
Table includes all single-use items used across all observed operations listed here and the carbon footprint per item as listed. Items used for individual operations varied, alongside number of items used. The material composition of items was determined through packaging and manufacturer information where available, and alternatively through expert assessment, taking into account available emission factors. Weight is total for a given item (accounting for multiple items where relevant). 'Use' equates to linen laundering of reusable linens. Carbon footprint per use equates to a single use of an item in a single operation. CO_2e= carbon dioxide equivalents. Ops.= operations

Item	Component	Material(s)	Weight (g), or cost where specified with (£)	Number of uses	Number of ops per use	Waste stream A	Waste stream B	Carbon footprint per use (g CO_2e)					
								Production	Use	Waste stream A	Waste stream B	Total (waste A)	Total (waste B)
**Reusable patient drapes**													
High fluid drape	Product	Polyethylene terephthalate	1876.3	75	1	Clothing recycling	N/A	100.88	864.09	0.53	N/A	965.51	N/A
	Packaging	Paper	51.53	1		Infectious waste		76.78	N/A	29.34	N/A	106.12	N/A
		Nylon (polyamide) 6 polymer	2.76					25.23	N/A	1.57	N/A	26.80	N/A
Huck towel	Product	Cotton fabric	120.55	75	1	Clothing recycling	N/A	10.90	55.52	0.03	N/A	66.45	N/A
	Packaging	Paper	20.63	1		Infectious waste		30.74	N/A	11.74	N/A	42.48	N/A
		Low density polyethylene film	4.6					11.96	N/A	2.62	N/A	14.58	N/A
Low fluid drape	Product	Polyethylene terephthalate	1171.14	75	1	Clothing recycling	N/A	62.97	539.35	0.33	N/A	602.64	N/A
	Packaging	Paper	71.04	1		Domestic waste		105.85	N/A	12.20	N/A	118.05	N/A
		Nylon (polyamide) 6 polymer	2.56					23.40	N/A	0.44	N/A	23.84	N/A
**Reusable personal protective equipment**													
Reusable surgical gown	Product	Polyethylene terephthalate	303.70	75	1	Clothing recycling	N/A	16.33	139.86	0.09	N/A	156.28	N/A

(including hand towels)		Rubber	15.98					0.61	7.36	0.0045	N/A	7.97	N/A
		Paper	13.41	1		Domestic waste	Clinical waste	19.98	N/A	2.30	14.40	22.28	34.39
	Packaging	Paper	19.90					29.65	N/A	3.42	21.38	33.07	51.03
		General polyethylene	6.42					29.65	N/A	3.42	21.38	33.07	51.03
Reusable surgical hat	Product	Cotton fabric	26.58	75	4	Clothing recycling	N/A	0.60	3.06	0.0019	N/A	3.66	N/A
Reusable scrubs	Product	Cotton fabric	421.17	75	4	Clothing recycling	N/A	9.52	48.49	0.0299	N/A	58.04	N/A
**Non-set reusable equipment**													
Diathermy pad lead	Product	Polyvinylchloride general	121.57	50	1	Non-infectious offensive waste	N/A	7.54	N/A	0.61	N/A	8.14	N/A
		Copper	13.51					1.03	N/A	0.07	N/A	1.10	N/A
Shaver base	Product	High density polyethylene resin	38.15	1,300	1	Infectious waste	Clinical waste	0.06	N/A	0.02	0.03	0.07	0.09
		Aluminium cast	35.43					0.18	N/A	0.02	0.03	0.20	0.21
		Lithium ion battery	35.43			Batteries recycling	N/A	0.17	N/A	0.0006	N/A	0.17	N/A
**Single-use personal protective equipment**													
Gloves (non-sterile, pair)	Product	Rubber	6.45	1	1	Infectious waste	Clinical waste	18.38	N/A	3.67	6.93	22.05	25.31
Sterile gloves (pair)	Product	Rubber	27.31	1	1	Infectious waste	Clinical waste	77.83	N/A	15.55	29.33	93.38	107.17
	Packaging	General polyethylene	5.98			Domestic waste		15.19	N/A	1.03	6.42	16.22	21.61
		Paper	5.91					8.81	N/A	1.02	6.35	9.82	15.15
Sterile gloves (two pairs)	Product	Rubber	42.14	1	1	Infectious waste	N/A	120.10	N/A	23.99	N/A	144.09	N/A
	Packaging	Paper	11.61			Domestic waste		17.30	N/A	1.99	N/A	19.29	N/A

		General polyethylene	5.61					14.25	N/A	0.96	N/A	15.21	N/A
Sterile gloves (one pair, latex free)	Product	General polyethylene	24.66	1	1	Infectious waste	Clinical waste	62.64	N/A	14.04	26.49	76.68	89.12
	Packaging	Paper	6.01			Domestic waste		8.95	N/A	1.03	6.46	9.99	15.41
		General polyethylene	5.83					14.81	N/A	1.00	6.26	15.81	21.07
Sterile under-gloves (pair)	Product	Rubber	18.54	1	1	Infectious waste	Clinical waste	52.84	N/A	10.56	19.91	63.39	72.75
	Packaging	General polyethylene	5.64			Domestic waste		14.33	N/A	0.97	6.06	15.29	20.38
		Paper	5.85					8.72	N/A	1.00	6.28	9.72	15.00
Surgical face mask	Product	Polypropylene oriented film	4.36	1	1	Infectious waste	Clinical waste	14.95	N/A	2.48	4.68	17.44	19.64
Surgical face mask with eye protection	Product	Polypropylene oriented film	6.23	1	1	Infectious waste	Clinical waste	21.37	N/A	3.55	6.69	24.92	28.06
		Low density polyethylene film	4.04					10.50	N/A	2.30	4.34	12.80	14.84
Surgical gown (including hand towels)	Product	Polypropylene oriented film	139.004	1	1	Infectious waste	Clinical waste	476.78	N/A	79.14	149.31	555.92	626.09
		Rubber	7.316					20.85	N/A	4.17	7.86	25.02	28.71
		Paper	13.83			Domestic waste		20.61	N/A	2.38	14.86	22.98	35.46
	Packaging	Paper	25.34					37.76	N/A	4.35	27.22	42.11	64.98
		Low density polyethylene film	5.11					13.29	N/A	0.88	5.49	14.16	18.77
Surgical hat	Product	Polypropylene oriented film	3.54	1	4	Infectious waste	Clinical waste	3.04	N/A	0.50	0.95	3.54	3.99
**Single-use patient drape and instrument table drapes**													
Patient drape (incise drape, with iodine)	Product	Low density polyethylene film	11.65	1	1	Infectious waste	N/A	30.29	N/A	6.63	N/A	36.92	N/A
		Iodine	£0.02					0.00	N/A	0.74	N/A	0.74	N/A
	Packaging	Paper	26.085					38.87	N/A	14.85	N/A	53.72	N/A

		General polyethylene	7.945					20.18	N/A	4.52	N/A	24.70	N/A
Table drape (instruments)	Product	Polyvinylchloride general	122.17	1	1	Infectious waste	N/A	378.73	N/A	69.55	N/A	448.28	N/A
		Polypropylene oriented film	61.09					209.54	N/A	34.78	N/A	244.32	N/A
	Packaging	Paper	12.64					18.83	N/A	7.20	N/A	26.03	N/A
		Polypropylene oriented film	9.05					31.04	N/A	5.15	N/A	36.19	N/A
		Low density polyethylene film	6.49					16.87	N/A	3.69	N/A	20.57	N/A
**Single-use equipment and medical devices**													
Absorbent towel pack	Product	Paper	28.47	1	1	Infectious waste	N/A	42.42	N/A	16.21	N/A	58.63	N/A
	Packaging	Paper	5.00					7.45	N/A	2.85	N/A	10.30	N/A
		Low density polyethylene film	2.16					5.62	N/A	1.23	N/A	6.85	N/A
Diathermy pad	Product	Aluminium foil	5.49	1	1	Infectious waste	Clinical waste	41.01	N/A	3.13	5.90	44.14	46.91
		General polyethylene	5.49					13.94	N/A	3.13	5.90	17.07	19.84
	Packaging	Polyethylene terephthalate	1.84					7.42	N/A	1.05	1.98	8.47	9.40
Diathermy tip	Product	Stainless steel	1.58	1	1	Medicinal contaminated sharps waste	N/A	9.72	N/A	1.70	N/A	11.42	N/A
		High density polyethylene resin	0.53					1.02	N/A	0.57	N/A	1.58	N/A
	Packaging	Low density polyethylene film	0.90			Infectious waste		2.34	N/A	0.51	N/A	2.85	N/A
		Paper	0.59					0.88	N/A	0.34	N/A	1.21	N/A
Gauze (sterile pack, 10x7.5 cm)	Product	Cotton fabric	19.66	1	1	Infectious waste	Clinical waste	133.29	N/A	11.19	21.12	144.49	154.41
	Packaging	Paper	4.73				Domestic waste	7.05	N/A	2.69	0.81	9.74	7.86

		Low density polyethylene film	1.54					4.00	N/A	0.88	0.26	4.88	4.27
Gauze (sterile pack, 30x30 cm)	Product	Cotton fabric	91.90	1	1	Infectious waste	N/A	623.08	N/A	52.32	N/A	675.40	N/A
	Packaging	Paper	12.67					18.88	N/A	7.21	N/A	26.09	N/A
		Low density polyethylene film	3.72					9.67	N/A	2.12	N/A	11.79	N/A
Gauze (sterile pack, 10x10 cm)	Product	Cotton fabric	25.52	1	1	Infectious waste	Clinical waste	173.03	N/A	14.53	27.41	187.55	200.44
	Packaging	Paper	5.12				Domestic waste	7.63	N/A	2.91	0.88	10.54	8.51
		Low density polyethylene film	1.63					4.24	N/A	0.93	0.28	5.17	4.52
Incontinence pad	Product	Cotton padding	15.09	1	1	Infectious waste	Clinical waste	19.32	N/A	8.59	16.21	27.91	35.52
		Low density polyethylene film	15.09					39.23	N/A	8.59	16.21	47.82	55.44
Kidney dish	Product	Paper	18.98	1	1	Clinical waste	N/A	28.28	N/A	20.39	N/A	48.67	N/A
	Packaging	Low density polyethylene film	5.86			Domestic waste		15.24	N/A	1.01	N/A	16.24	N/A
		Paper	4.89					7.29	N/A	0.84	N/A	8.13	N/A
Light handle	Product	Low density polyethylene film	4.78	1	1	Infectious waste	Clinical waste	12.43	N/A	2.72	5.13	15.15	17.56
	Packaging	Low density polyethylene film	3.38				Domestic waste	8.79	N/A	1.92	0.58	10.71	9.37
		Paper	1.93					2.88	N/A	1.10	0.33	3.97	3.21
Mesh	Product	Polypropylene oriented film	1.06	1	1	N/A	N/A	3.64	N/A	N/A	N/A	3.64	3.64
	Packaging	Paper	84.66			Infectious waste	Clinical waste	126.14	N/A	48.20	90.94	174.34	217.08
		Low density polyethylene film	7.17					18.64	N/A	4.08	7.70	22.72	26.34
		Polypropylene oriented film	7.15					24.52	N/A	4.07	7.68	28.60	32.20
Monopolar diathermy	Product	Low density polyethylene resin	78.58	1	1	Infectious waste	Clinical waste	163.45	N/A	44.74	84.41	208.18	247.85

with smoke evacuation system		Polyvinylchloride general	54.07					167.62	N/A	30.78	58.08	198.40	225.70
		High density polyethylene resin	39.62					76.47	N/A	22.56	42.56	99.02	119.02
		Stainless steel	5.36			Medicinal contaminated sharps waste	N/A	32.91	N/A	5.75	N/A	38.67	N/A
		Copper	3.79			Infectious waste	Clinical waste	14.44	N/A	2.16	4.07	16.60	18.51
		Acrylonitrile Butadiene Styrene	1.86					6.99	N/A	1.06	2.00	8.05	8.99
	Packaging	Low density polyethylene film	11.33					29.46	N/A	6.45	12.17	35.91	41.63
		Polypropylene oriented film	8.47					29.05	N/A	4.82	9.10	33.87	38.15
		Paper	1.34					2.00	N/A	0.76	1.44	2.76	3.44
Nail scrubbing brush	Product	Low density polyethylene resin	9.01	1	1	Domestic waste	Clinical waste	18.74	N/A	1.55	9.68	20.29	28.42
		Polyurethane flexible foam	2.05					9.92	N/A	0.35	2.20	10.27	12.12
		Polypropylene injection moulding	0.91					4.09	N/A	0.16	0.98	4.24	5.06
	Packaging	Low density polyethylene film	1.31					3.41	N/A	0.23	1.41	3.63	4.81
		Paper	0.6					0.89	N/A	0.10	0.64	1.00	1.54
Needle (green)	Product	Stainless steel	0.20	1	1	Medicinal contaminated sharps waste	N/A	1.23	N/A	0.21	N/A	1.44	N/A
		Polypropylene injection moulding	0.05					0.22	N/A	0.05	N/A	0.28	N/A
	Packaging	High density polyethylene resin	0.52			Infectious waste	Domestic waste	1.00	N/A	0.30	0.09	1.30	1.09
		Low density polyethylene film	0.12					0.31	N/A	0.07	0.02	0.38	0.33
		Paper	0.11					0.16	N/A	0.06	0.02	0.23	0.18
Needle counter	Product	Acrylonitrile Butadiene Styrene	44.50	1	1		N/A	167.32	N/A	47.80	N/A	215.12	N/A

		Polyurethane rigid foam	4.00			Medicinal contaminated sharps waste		17.04	N/A	4.30	N/A	21.34	N/A
	Packaging	Low density polyethylene film	4.38			Infectious waste	Domestic waste	11.39	N/A	2.49	0.75	13.88	12.14
		Polypropylene oriented film	1.15					3.94	N/A	0.65	0.20	4.60	4.14
Nonwoven dressing (10x20 cm)	Product	Cotton padding	2.21	1	1	Infectious waste	Clinical waste	2.83	N/A	1.26	2.38	4.09	5.21
		Low density polyethylene film	0.12					0.30	N/A	0.07	0.13	0.37	0.43
	Packaging	Paper	4.17			Domestic waste	N/A	6.21	N/A	0.72	N/A	6.93	N/A
		Polypropylene oriented film	2.04					7.00	N/A	0.35	N/A	7.35	N/A
		Low density polyethylene film	1.71					4.45	N/A	0.29	N/A	4.74	N/A
Nonwoven dressing (10x30 cm)	Product	Cotton padding	10.67	1	1	Clinical waste	N/A	13.66	N/A	11.46	N/A	25.12	N/A
		Low density polyethylene film	0.56					1.46	N/A	0.60	N/A	2.06	N/A
	Packaging	Paper	3.22			Domestic waste		4.80	N/A	0.55	N/A	5.35	N/A
		Polypropylene oriented film	2.9					9.95	N/A	0.50	N/A	10.45	N/A
		Low density polyethylene film	2.53					6.58	N/A	0.43	N/A	7.01	N/A
Pre-operative adhesive glove	Product	Low density polyethylene film	2.60	1	1	Infectious waste	Clinical waste	6.76	N/A	1.48	2.79	8.24	9.55
	Packaging	Paper	1.73					2.58	N/A	0.98	1.86	3.56	4.44
Reinforced skin closure strip	Product	General polyethylene	0.66	1	1	Clinical waste	N/A	1.68	N/A	0.71	N/A	2.39	N/A
	Packaging	Paper	1.49					2.22	N/A	1.60	N/A	3.82	N/A
		Low density polyethylene film	0.74					1.92	N/A	0.79	N/A	2.72	N/A
Shaver head	Product	Stainless steel	2.9	1	1		N/A	17.82	N/A	3.11	N/A	20.94	N/A

		Acrylonitrile Butadiene Styrene	2.76			Medicinal contaminated sharps waste		10.36	N/A	2.96	N/A	13.32	N/A
		Low density polyethylene resin	2.76					5.73	N/A	2.96	N/A	8.69	N/A
	Packaging	Polyethylene terephthalate	0.77			Infectious waste	Clinical waste	3.10	N/A	0.44	0.83	3.54	3.93
		Paper	0.23					0.34	N/A	0.13	0.25	0.47	0.59
Specimen pot (40 ml 4% formaldehyde)	Product	4% Formaldehyde	£0.20	1	1	Clinical waste	N/A	42.97	N/A	N/A	N/A	42.97	N/A
	(Product weight-for waste)		40.00					0.00	N/A	42.97	N/A	42.97	N/A
	Product	Polypropylene injection moulding	6.53					29.32	N/A	7.01	N/A	36.33	N/A
	Product	High density polyethylene resin	5.19					10.02	N/A	5.57	N/A	15.59	N/A
Surgical blade (10)	Product	Stainless steel	0.61	1	1	Medicinal contaminated sharps waste	N/A	3.75	N/A	0.66	N/A	4.40	N/A
	Packaging	Aluminium foil	0.46			Infectious waste	Domestic waste	3.44	N/A	0.26	0.08	3.70	3.52
Surgical suspensory bandage	Product	Cotton fabric	23.30	1	1	Domestic waste	N/A	157.97	N/A	4.00	N/A	161.98	N/A
		Stainless steel	2.92					17.94	N/A	0.50	N/A	18.45	N/A
	Packaging	Low density polyethylene film	3.55			Clinical waste		9.23	N/A	3.81	N/A	13.04	N/A
		Paper	3.02					4.50	N/A	3.24	N/A	7.74	N/A
Suture (braided, absorbable, 0)	Product	Stainless steel	0.26	1	1	Medicinal contaminated sharps waste	N/A	1.60	N/A	0.28	N/A	1.88	N/A
		Nylon (polyamide) 6 polymer	0.09					0.82	N/A	0.10	N/A	0.92	N/A
	Packaging	Aluminium foil	1.22			Infectious waste	Domestic waste	9.11	N/A	0.69	0.21	9.81	9.32
		Paper	0.91					1.36	N/A	0.52	0.16	1.87	1.51
		Polypropylene oriented film	0.52					1.78	N/A	0.30	0.09	2.08	1.87

		Low density polyethylene film	0.50					1.30	N/A	0.28	0.09	1.58	1.39
Suture (braided, absorbable, 2-0)	Product	Stainless steel	0.26	1	1	Medicinal contaminated sharps waste	N/A	1.60	N/A	0.28	N/A	1.88	N/A
		Nylon (polyamide) 6 polymer	0.09					0.82	N/A	0.10	N/A	0.92	N/A
	Packaging	Aluminium foil	1.22			Infectious waste	Domestic waste	9.11	N/A	0.69	0.21	9.81	9.32
		Paper	0.91					1.36	N/A	0.52	0.16	1.87	1.51
		Polypropylene oriented film	0.52					1.78	N/A	0.30	0.09	2.08	1.87
		Low density polyethylene film	0.50					1.30	N/A	0.28	0.09	1.58	1.39
Suture (monofilament, absorbable, 3-0)	Product	Stainless steel	0.26	1	1	Medicinal contaminated sharps waste	N/A	1.60	N/A	0.28	N/A	1.88	N/A
		Nylon (polyamide) 6 polymer	0.09					0.82	N/A	0.10	N/A	0.92	N/A
	Packaging	Aluminium foil	1.22			Infectious waste	Domestic waste	9.11	N/A	0.69	0.21	9.81	9.32
		Paper	0.91					1.36	N/A	0.52	0.16	1.87	1.51
		Polypropylene oriented film	0.52					1.78	N/A	0.30	0.09	2.08	1.87
		Low density polyethylene film	0.50					1.30	N/A	0.28	0.09	1.58	1.39
Suture (monofilament, nonabsorbable, 1)	Product	Stainless steel	0.26	1	1	Medicinal contaminated sharps waste	N/A	1.60	N/A	0.28	N/A	1.88	N/A
		Nylon (polyamide) 6 polymer	0.09					0.82	N/A	0.10	N/A	0.92	N/A
	Packaging	Aluminium foil	1.22			Infectious waste	Domestic waste	9.11	N/A	0.69	0.21	9.81	9.32
		Paper	0.91					1.36	N/A	0.52	0.16	1.87	1.51
		Polypropylene oriented film	0.52					1.78	N/A	0.30	0.09	2.08	1.87
		Low density polyethylene film	0.50					1.30	N/A	0.28	0.09	1.58	1.39
Suture (monofilament,	Product	Stainless steel	0.26	1	1		N/A	1.60	N/A	0.28	N/A	1.88	N/A

nonabsorbable, 2-0)		Nylon (polyamide) 6 polymer	0.09			Medicinal contaminated sharps waste		0.82	N/A	0.10	N/A	0.92	N/A
	Packaging	Aluminium foil	1.22			Infectious waste	Domestic waste	9.11	N/A	0.69	0.21	9.81	9.32
		Paper	0.91					1.36	N/A	0.52	0.16	1.87	1.51
		Polypropylene oriented film	0.52					1.78	N/A	0.30	0.09	2.08	1.87
		Low density polyethylene film	0.5					1.30	N/A	0.28	0.09	1.58	1.39
Syringe (20 ml)	Product	Polypropylene injection moulding	7.10	1	1	Infectious waste	Clinical waste	31.90	N/A	4.04	7.63	35.94	39.53
		General polyethylene	7.10					18.04	N/A	4.04	7.63	22.09	25.67
	Product	Rubber	0.59					1.69	N/A	0.34	0.64	2.02	2.32
		Low density polyethylene film	1.11					2.89	N/A	0.63	0.19	3.52	3.08
		Paper	0.72					1.07	N/A	0.41	0.12	1.48	1.20
**Pharmaceuticals**													
Chlorhexidine 1% from 500 ml container (100 ml)	Product and packaging	Chlorhexidine 2%	£0.56	1	1	N/A	N/A	107.30	N/A	N/A	N/A	107.30	N/A
Levobupivacaine 5mg/10 ml (10 ml)	Product	Levobupivacaine	10	1	1	N/A	N/A	230.00	N/A	N/A	N/A	230.00	N/A
	Packaging	Low density polyethylene resin	2.65	1	1	Infectious waste	Clinical waste	5.51	N/A	1.51	2.85	7.02	8.36
		Low density polyethylene film	0.46	1	1			1.20	N/A	0.26	0.49	1.46	1.69
		Paper	0.40	1	1			0.60	N/A	0.23	0.43	0.82	1.03
Povidone iodine 10% from 500 ml	Product and packaging	Povidone iodine	£0.96	1	1	N/A	N/A	183.24	N/A	N/A	N/A	183.24	183.24

container (60 ml)													
Sodium Chloride 0.9% from 1 litre bottle (50 ml)	Product and packaging	Sodium chloride	£0.20	1	1	N/A	N/A	37.94	N/A	N/A	N/A	37.94	37.94
Topical skin adhesive (0.8 g)	Product	Topical skin adhesive (0.8 g)	£20.47	1	1	N/A	N/A	3,922.02	N/A	N/A	N/A	3,922.02	N/A
	(Packaging - weight for waste)		4.38	1	1	Infectious waste		0.00	N/A	2.49	N/A	2.49	N/A
**Cleaning products, waste**													
Black waste bag (domestic waste) with cable tie	Product	Polypropylene oriented film	22.37	1	1	Domestic waste	N/A	76.73	N/A	3.84	N/A	80.57	N/A
		Nylon (polyamide) 6 polymer	2.58					23.58	N/A	0.44	N/A	24.02	N/A
Chlorine tablet	Product and packaging	Chlorine tablet	£0.04	1	1	N/A	N/A	7.01	N/A	0.00	N/A	7.01	N/A
Clear bin bag (for swab count)	Product	Low density polyethylene film	12.48	1	1	Clinical waste	N/A	32.45	N/A	13.41	N/A	45.85	N/A
		Nylon (polyamide) 6 polymer	2.58					23.58	N/A	2.77	N/A	26.35	N/A
Clear bin bag (recycling) with cable tie	Product	Low density polyethylene film	15.01	1	1	Domestic waste	N/A	39.03	N/A	2.58	N/A	41.60	N/A
		Nylon (polyamide) 6 polymer	2.58					23.58	N/A	0.44	N/A	24.02	N/A
Disinfectant sachet	Product	Disinfectant	£0.70	1	4	Infectious waste	N/A	33.53	N/A	N/A	N/A	33.53	N/A
	(Packaging - weight for waste)		5.81					N/A	N/A	0.83	N/A	0.83	N/A
Disinfectant wipe	Product	Non-woven polyester	3.34	1	1	Infectious waste	Clinical waste	18.57	N/A	1.90	3.59	20.47	22.16
Green bag (linen	Product	Low density polyethylene film	16.47	1	4	Domestic waste	N/A	10.71	N/A	0.71	N/A	11.41	N/A

laundering) with cable tie		Nylon (polyamide) 6 polymer	2.58					5.90	N/A	0.11	N/A	6.01	N/A
Mop head	Product	Nylon (polyamide) 6 polymer	70.36	1	4	Infectious waste	Clinical waste	160.77	N/A	10.01	18.89	170.79	179.67
		High density polyethylene resin	30.16					14.55	N/A	4.29	8.10	18.84	22.65
Orange waste bag (infectious waste, large) with cable tie	Product	Polypropylene oriented film	70.61	1	1	Infectious waste	N/A	242.19	N/A	40.20	N/A	282.39	N/A
		Nylon (polyamide) 6 polymer	2.58					23.58	N/A	1.47	N/A	25.05	N/A
Orange waste bag (infectious waste, small) with cable tie	Product	Polypropylene oriented film	27.75	1	1	Infectious waste	N/A	95.18	N/A	15.80	N/A	110.98	N/A
		Nylon (polyamide) 6 polymer	2.58					23.58	N/A	1.47	N/A	25.05	N/A
Red bag (linen laundering) with cable tie	Product	Polypropylene oriented film	29.22	1	4	Domestic waste	N/A	25.06	N/A	1.25	N/A	26.31	N/A
		Nylon (polyamide) 6 polymer	2.58					5.90	N/A	0.11	N/A	6.01	N/A
Yellow waste bag (clinical waste) with cable tie	Product	Polypropylene oriented film	28.15	1	1	Clinical waste	N/A	96.55	N/A	30.24	N/A	126.79	N/A
		Nylon (polyamide) 6 polymer	2.58					23.58	N/A	2.77	N/A	26.35	N/A

Supplementary Table 6: Carbon footprint of reusable instrument sets used for knee arthroplasty

Table includes all reusable instrument sets and individually wrapped instruments across all observed operations, and the carbon footprint per set. Sets and individually wrapped instruments used for individual operations varied. The material composition of items was determined through packaging and manufacturer information where available, and alternatively through expert assessment, taking into account available emission factors. Weight is total for a given item (accounting for multiple items where relevant). Some items listed as 'bottom tray'/'top tray' where not individually identified by theatre or sterile services staff and so were clustered. 'Use' equates to decontamination of instruments, determined per set. All waste of given item disposed of using consistent waste stream (A). CO_2e= carbon dioxide equivalents

Category	Item	Material(s)	Weight (g)	Number of uses	Waste stream A	Carbon footprint per use (g CO_2e)		
						Production	Use	Waste stream A
**Basic major orthopaedic set**								
**Sterile barrier system**	Basket	Stainless steel	955.83	116	Scrap metal recycling	50.63	See total	0.1755
	Container	Aluminium cast	3213.76	1000	Scrap metal recycling	21.60		0.0684
	Filter paper	Paper	3.55	1	Infectious waste	5.29	N/A	2.0211
	Identification tag	High density polyethylene resin	8.21	46	Non-infectious offensive waste	0.34	See total	0.0444
	Kit list	Paper	4.85	1	Infectious waste	7.23	N/A	2.7612
	Pin mat	Silicone	51.04	888	Non-infectious offensive waste	0.19	See total	0.0143
	Tamper proof tags	General plastics	1.78	1	Infectious waste	5.89	N/A	1.0134
**Instruments and set items**	Bone hook	Stainless steel	65.96	2441	Scrap metal recycling	0.17	See total	0.0006
	Bone nibbler x2	Stainless steel	488.51	888	Scrap metal recycling	3.38		0.0117
	Bonney toothed dissecting forceps	Stainless steel	47.40	1775	Scrap metal recycling	0.16		0.0006
	BP scalpel handle (no. 3) x2	Stainless steel	51.86	666	Scrap metal recycling	0.48		0.0017
	BP scalpel handle (no. 3L)	Stainless steel	44.23	666	Scrap metal recycling	0.41		0.0014

	Bristow elevator	Stainless steel	108.95	888	Scrap metal recycling	0.75		0.0026
	Capener gouge (large)	Stainless steel	122.61	666	Medicinal contaminated sharps waste	1.13		0.1979
		Acrylonitrile Butadiene Styrene	30.65			0.17		0.0495
	Capener gouge (small)	Stainless steel	121.84	666	Medicinal contaminated sharps waste	1.12		0.1966
		Acrylonitrile Butadiene Styrene	30.46			0.17		0.0492
	Cement gun	Stainless steel	454.15	888	Non-infectious offensive waste	3.14		0.1274
		Glass reinforced plastic	454.15			4.14		0.1274
	Curette	Stainless steel	85.12	976	Medicinal contaminated sharps waste	0.54		0.0936
		Glass reinforced plastic	85.12			0.71		0.0937
	Curette (double ended)	Stainless steel	46.98	888	Scrap metal recycling	0.33		0.0011
	Galabin ligature carrier	Stainless steel	27.52	1775	Scrap metal recycling	0.10		0.0003
	Gillies toothed dissecting forceps	Stainless steel	30.10	1775	Scrap metal recycling	0.10		0.0004
	Heath mallet	Stainless steel	929.94	1553	Scrap metal recycling	3.68		0.0127
		Rubber	232.49			0.43		0.0032
	Hohmann lever bone x2	Stainless steel	199.16	1109	Scrap metal recycling	1.10		0.0038
	Kocher artery forceps (7") x4	Stainless steel	191.16	1775	Scrap metal recycling	0.66		0.0023
	Langenbeck retractor (large) x2	Stainless steel	233.20	1997	Scrap metal recycling	0.72		0.0025
	Langenbeck retractor (medium) x2	Stainless steel	163.82	1997	Scrap metal recycling	0.50		0.0017
	Mayo needle holder x 2	Stainless steel	99.64	1775	Scrap metal recycling	0.34		0.0012
	Mayo scissor (curved, 6")	Stainless steel	63.35	1109	Scrap metal recycling	0.35		0.0012

	Mayo scissor (straight, 6")	Stainless steel	48.60	976	Scrap metal recycling	0.31		0.0011
	McDonald dissector	Stainless steel	23.88	1775	Scrap metal recycling	0.08		0.0003
	McGoey punch	Stainless steel	270.51	888	Scrap metal recycling	1.87		0.0065
	McIndoe scissor	Stainless steel	42.39	1109	Scrap metal recycling	0.23		0.0008
	Norfolk and Norwich retractor x2	Stainless steel	384.70	1109	Scrap metal recycling	2.13		0.0074
	Osteotome (25 mm)	Stainless steel	127.17	1109	Scrap metal recycling	0.70		0.0024
	Osteotome (8 mm)	Stainless steel	134.27	1109	Scrap metal recycling	0.74		0.0026
	Pin for instruments	Stainless steel	12.74	2663	Scrap metal recycling	0.03		0.0001
	Quiver	Polypropylene injection moulding	96.17	888	Scrap metal recycling	0.49		0.0023
	Scalpel handle (no. 4) x2	Stainless steel	58.72	666	Scrap metal recycling	0.54		0.0019
	Scalpel handle (no. 4 L)	Stainless steel	57.62	666	Scrap metal recycling	0.53		0.0018
	Spencer Wells artery forceps (curved, 7") x2	Stainless steel	102.12	1775	Scrap metal recycling	0.35		0.0012
	Spencer Wells artery forceps (straight, 5") x2	Stainless steel	62.94	1775	Scrap metal recycling	0.22		0.0008
	Sponge holder x3	Stainless steel	232.29	1997	Scrap metal recycling	0.71		0.0025
	Stitch scissor	Stainless steel	31.24	1331	Scrap metal recycling	0.14		0.0005
	Towel clip x3	Stainless steel	88.11	1109	Scrap metal recycling	0.49		0.0017
	Trethowan bone lever x2	Stainless steel	81.48	1109	Scrap metal recycling	0.45		0.0016
	Treves dissecting forceps	Stainless steel	29.11	1775	Scrap metal recycling	0.10		0.0003
	Universal orange scissor	Stainless steel	45.69	444	Medicinal contaminated sharps waste	0.63		0.1106
		Glass reinforced plastic	45.69			0.83		0.1106

**Total basic major orthopaedic set=2,288.88 g CO_2e**						**127.58**	**2,153.96**	**7.34**
**Miscellaneous knee system set**								
**Sterile barrier system**	Container	Aluminium cast	3,099.44	1000	Scrap metal recycling	20.83	See total	0.0660
	Filter paper	Paper	3.55	1	Infectious waste	5.29	N/A	2.0211
	Identification tag	High density polyethylene resin	8.21	46	Non-infectious offensive waste	0.34	See total	0.0444
	Kit list	Paper	4.85	1	Infectious waste	7.23	N/A	2.7612
	Tamper proof tags	General plastics	1.78	1	Infectious waste	5.89		1.0134
	Tray	Stainless steel	2,278.39	1000	Non-infectious offensive waste	14.00	See total	0.5672
		Acrylonitrile Butadiene Styrene	119.92		Non-infectious offensive waste	0.45		0.0299
**Instruments and set items**	Drill bit/ pins x4	Stainless steel	10.59	688	Scrap metal recycling	0.09		0.0003
	Instruments (bottom tray, 12 items)	Stainless steel	1,102.82	688	Non-infectious offensive waste	9.85		0.3991
		High density polyethylene resin	275.71			0.77		0.0998
	Instruments (top tray, 13 items)	Stainless steel	1,320.89	688	Non-infectious offensive waste	11.80		0.4780
		High density polyethylene resin	330.22			0.93		0.1195
	Nails (short, headed) x4	Stainless steel	7.14	688	Scrap metal recycling	0.06		0.0002
	Pins (long, headless) x4	Stainless steel	14.24	688	Scrap metal recycling	0.13		0.0004
**Total miscellaneous set=2,239.23 g CO_2e**						**77.67**	**2,153.96**	**7.60**
**Femoral and tibial preparation set (size 3-6)**								
	Container	Aluminium cast	3,099.44	1000	Scrap metal recycling	20.83	See total	0.0660

**Sterile barrier system**								
	Filter paper	Paper	3.55	1	Infectious waste	5.29	N/A	2.0211
	Identification tag	High density polyethylene resin	8.21	46	Non-infectious offensive waste	0.34	See total	0.0444
	Kit list	Paper	4.85	1	Infectious waste	7.23	N/A	2.7612
	Tamper proof tags	General plastics	1.78	1	Infectious waste	5.89		1.0134
	Tray	Stainless steel	2,996.19	1000	Non-infectious offensive waste	18.41	See total	0.7458
		Acrylonitrile Butadiene Styrene	157.69			0.59		0.0393
**Instruments and set items**	Instruments (bottom tray, 13 items)	Stainless steel	3,679.06	2040	Non-infectious offensive waste	11.08		0.4489
		High density polyethylene resin	164.47			0.16		0.0201
	Instruments (top tray, 17 items)	Stainless steel	1,480.21	2040	Scrap metal recycling	4.46		0.0155
**Total femoral and tibial preparation set (size 3-6)= 2,235.42 g CO_2e**						**74.28**	**2,153.96**	**7.18**
**Cruciate retaining femoral and tibial trialing set (size 3-6)**								
**Sterile barrier system**	Container	Aluminium cast	3,099.44	1000	Scrap metal recycling	20.83	See total	0.0660
	Filter paper	Paper	3.55	1	Infectious waste	5.29	N/A	2.0211
	Identification tag	High density polyethylene resin	8.21	46	Non-infectious offensive waste	0.34	See total	0.0444
	Kit list	Paper	4.85	1	Infectious waste	7.23	N/A	2.7612
	Tamper proof tags	General plastics	1.78	1	Infectious waste	5.89		1.0134
	Tray	Stainless steel	2,457.99	1000	Non-infectious offensive waste	15.10	See total	0.6119
		Acrylonitrile Butadiene Styrene	129.37	1000		0.49		0.0322
**Instruments and set items**	Instruments (bottom tray, 12 items)	Stainless steel	370.56	1126	Scrap metal recycling	2.02		0.0070

	Instruments (top tray, 16 items)	Stainless steel	1,988.55	1126	Scrap metal recycling	10.86		0.0376
		Nylon (polyamide) 6 polymer	61.40		Non-infectious offensive waste	0.50		0.0136
**Total cruciate retaining femoral and tibial trialing set (size 3-6)= 2,229.12 g CO_2e**						**68.55**	**2,153.96**	**6.61**
**Posterior stabilised femoral and tibial trialing set (size 3-6)**								
**Sterile barrier system**	Container	Aluminium cast	3,099.44	1000	Scrap metal recycling	20.83	See total	0.0660
	Filter paper	Paper	3.55	1	Infectious waste	5.29	N/A	2.0211
	Identification tag	High density polyethylene resin	8.21	46	Non-infectious offensive waste	0.34	See total	0.0444
	Kit list	Paper	4.85	1	Infectious waste	7.23	N/A	2.7612
	Tamper proof tags	General plastics	1.78	1	Infectious waste	5.89		1.0134
	Tray	Stainless steel	2,643.49	1000	Non-infectious offensive waste	16.24	See total	0.6580
		Acrylonitrile Butadiene Styrene	139.13			0.52		0.0346
**Instruments and set items**	Instruments (bottom tray, 16 items)	Acrylonitrile Butadiene Styrene	407.84	610	Non-infectious offensive waste	2.51		0.1664
	Instruments (top tray, 16 items)	Stainless steel	2,874.83	610	Scrap metal recycling	28.96		0.1004
		Nylon (polyamide) 6 polymer	69.35		Non-infectious offensive waste	1.04		0.0283
**Total posterior stabilised femoral and tibial trialing set (size 3-6)= 2,249.72 g CO_2e**						**88.86**	**2,153.96**	**6.89**
**Cruciate retaining femoral and tibial preparation and trialing set (size 1,2,7,8)**								
**Sterile barrier system**	Container	Aluminium cast	3,099.44	1,000	Scrap metal recycling	20.83	See total	0.0660
	Filter paper	Paper	3.55	1	Infectious waste	5.29	N/A	2.0211
	Identification tag	High density polyethylene resin	8.21	46	Non-infectious offensive waste	0.34	See total	0.0444

	Kit list	Paper	4.85	1	Infectious waste	7.23	N/A	2.7612
	Tamper proof tags	General plastics	1.78	1	Infectious waste	5.89		1.0134
	Tray	Stainless steel	2,667.63	1,000	Non-infectious offensive waste	16.39	See total	0.6641
		Acrylonitrile Butadiene Styrene	140.40			0.53		0.0349
**Instruments and set items**	Instruments (bottom tray, 18 items)	Stainless steel	1685.78	174	Scrap metal recycling	59.54		0.2063
		Nylon (polyamide) 6 polymer	235.31		Non-infectious offensive waste	12.36		0.3366
	Instruments (top tray, 19 items)	Stainless steel	1812.36	174	Scrap metal recycling	64.01		0.2218
		Nylon (polyamide) 6 polymer	220.42		Non-infectious offensive waste	11.58		0.3153
**Total cruciate retaining femoral and tibial preparation and trialing set (size 1,2,7,8)= 2,365.63 g CO_2e**						**203.98**	**2,153.96**	**7.69**
**Patella preparation and trialing set**								
**Sterile barrier system**	Container	Aluminium cast	3,099.44	1,000	Scrap metal recycling	20.83	See total	0.0660
	Filter paper	Paper	3.55	1	Infectious waste	5.29	N/A	2.0211
	Identification tag	High density polyethylene resin	8.21	46	Non-infectious offensive waste	0.34	See total	0.0444
	Kit list	Paper	4.85	1	Infectious waste	7.23	N/A	2.7612
	Tamper proof tags	General plastics	1.78	1	Infectious waste	5.89		1.0134
	Tray	Stainless steel	2944.47	1,000	Non-infectious offensive waste	18.09	See total	0.7330
		Acrylonitrile Butadiene Styrene	154.97			0.58		0.0386
**Instruments and set items**	Instruments (bottom tray, 13 items)	Stainless steel	49.20	500	Scrap metal recycling	0.60		0.0021
	Instruments (top tray, 5 items)	Stainless steel	1300.49	500	Scrap metal recycling	15.98		0.0554
		Brass	407.50			3.91		0.0174

**Total patella preparation and trialing set= 2,239.47 g CO_2e**						**78.76**	**2,153.96**	**6.75**
**Orthopaedic surgical drill set**								
**Sterile barrier system**	Container	Aluminium cast	3,099.44	1,000	Scrap metal recycling	20.83	See total	0.0660
	Filter paper	Paper	3.55	1	Infectious waste	5.29	N/A	2.0211
	Identification tag	High density polyethylene resin	8.21	46	Non-infectious offensive waste	0.34	See total	0.0444
	Kit list	Paper	4.85	1	Infectious waste	7.23	N/A	2.7612
	Tamper proof tags	General plastics	1.78	1	Infectious waste	5.89		1.0134
	Tray	Stainless steel	1322.95	1,000	Non-infectious offensive waste	8.13	See total	0.3293
**Instruments and set items**	Instruments (13 items)	Stainless steel	3498.15	473	Scrap metal recycling	45.45		0.0282
		Acrylonitrile Butadiene Styrene	320.20		Non-infectious offensive waste	2.55		0.1685
**Total orthopaedic surgical drill set= 2,255.92 g CO_2e**						**95.70**	**2,153.96**	**626**
**Knee navigation set**								
**Sterile barrier system**	Container	Aluminium cast	3,099.44	1,000	Scrap metal recycling	20.83	See total	0.0660
	Filter paper	Paper	3.55	1	Infectious waste	5.29	N/A	2.0211
	Identification tag	High density polyethylene resin	8.21	46	Non-infectious offensive waste	0.34	See total	0.0444
	Kit list	Paper	4.85	1	Infectious waste	7.23	N/A	2.7612
	Pin mat	Silicone	110.25	888	Non-infectious offensive waste	0.41	See total	0.0309
	Tamper proof tags	General plastics	1.78	1	Infectious waste	5.89	N/A	1.0134
	Tray	Stainless steel	3089.65	1000	Scrap metal recycling	18.99	See total	0.0658

**Instruments and set items**	Instruments (13 items)	Stainless steel	901.24	248	Non-infectious offensive waste	22.38		0.9064
		High density polyethylene resin	56.33			0.44		0.0567
		Acrylonitrile Butadiene Styrene	168.98			2.57		0.1700
**Total knee navigation set= 2,245.46 g CO_2e**						**84.36**	**2,153.96**	**7.14**
**Diathermy extras**								
**Sterile barrier system**	Flexible pouch	General polyethylene	15.42	1	Infectious waste	39.17	N/A	8.7788
		Paper	10.46			15.59		5.9550
	Identification tag	High density polyethylene resin	8.21	46	Non-infectious offensive waste	0.34	See total	0.0444
	Kit list	Paper	4.85	1	Infectious waste	7.23	N/A	2.7612
	Tray	Paper	20.41	1	Scrap metal recycling	30.41	See total	0.4346
**Instruments and set items**	Bipolar diathermy forceps	Stainless steel	31.98	40	Medicinal contaminated sharps waste	4.91		0.8588
		High density polyethylene resin	31.98			1.54		0.8588
	Diathermy lead	Polyvinylchloride general	74.62	50	Non-infectious offensive waste	4.63		0.3715
		Copper	8.29			0.63		0.0413
	Monopolar diathermy	Stainless steel	28.91	40	Medicinal contaminated sharps waste	4.44		0.7763
		High Density Polyethylene Resin	28.91			1.39		0.7763
**Total diathermy extras= 277.33 g CO_2e**						**110.28**	**145.38**	**21.66**
**Bipolar diathermy**								
	Flexible pouch	General polyethylene	15.42	1	Infectious waste	39.17	N/A	8.7788

**Sterile barrier system**		Paper	10.46			15.59		5.9550
**Instruments and set items**	Bipolar diathermy forceps	Stainless steel	31.98	40	Medicinal contaminated sharps waste	4.91	See total	0.8588
		High density polyethylene resin	31.98			1.54		0.8588
**Bipolar diathermy = 223.04 g CO_2e**						**61.21**	**145.38**	**16.45**
**Diathermy lead**								
**Sterile barrier system**	Flexible pouch	General polyethylene	15.42	1	Infectious waste	39.17	N/A	8.7788
		Paper	10.46			15.59		5.9550
	Identification tag	High density polyethylene resin	8.21	46	Non-infectious offensive waste	0.34	See total	0.0444
**Instruments and set items**	Diathermy lead	Polyvinylchloride general	74.62	50	Non-infectious offensive waste	4.63		0.3715
		Copper	8.29			0.63		0.0413
**Total diathermy lead= 220.93 g CO_2e**						**60.35**	**145.38**	**15.19**
**Blunt Hohman bone elevator**								
**Sterile barrier system**	Flexible pouch	General polyethylene	11.14	1	Infectious waste	28.30	N/A	6.3421
		Paper	8.06			12.01		4.5887
	Identification tag	High density polyethylene resin	8.21	46	Non-infectious offensive waste	0.34	See total	0.0444
**Instruments and set items**	Blunt Hohman bone elevator	Stainless steel	131.44	375	Scrap metal recycling	2.15		0.0075
**Total blunt Hohman bone elevator= 199.17 g CO_2e**						**42.80**	**145.38**	**10.98**
**Lanes tissue forceps**								

**Sterile barrier system**	Flexible pouch	General polyethylene	11.14	1	Infectious waste	28.30	N/A	6.3421
		Paper	8.06			12.01		4.5887
	Identification tag	High density polyethylene resin	8.21	46	Non-infectious offensive waste	0.34	See total	0.0444
**Instruments and set items**	Lanes tissue forceps	Stainless steel	81.28	300	Scrap metal recycling	1.66		0.0058
**Total Lanes tissue forceps= 198.68 g CO_2e**						**42.31**	**145.38**	**10.98**
**Light cover**								
**Sterile barrier system**	Flexible pouch	General polyethylene	11.14	1	Infectious waste	28.30	N/A	6.3421
		Paper	8.06			12.01		4.5887
	Identification tag	High density polyethylene resin	8.21	46	Non-infectious offensive waste	0.34	See total	0.0444
**Instruments and set items**	Light cover	High density polyethylene resin	183.71	393	Non-infectious offensive waste	0.90		0.2661
**Total light cover= 198.18 g CO_2e**						**41.55**	**145.38**	**11.24**
**Light handle**								
**Sterile barrier system**	Flexible pouch	General polyethylene	11.14	1	Infectious waste	28.30	N/A	6.3421
		Paper	8.06			12.01		4.5887
	Identification tag	High density polyethylene resin	8.21	46	Non-infectious offensive waste	0.34	See total	0.0444
**Instruments and set items**	Light handle	High density polyethylene resin	87.05	188	Non-infectious offensive waste	0.89		0.2636
**Total light handle= 198.17 g CO_2e**						**41.54**	**145.38**	**11.24**
**Non-toothed lamina spreader (large)**								

**Sterile barrier system**	Flexible pouch	General polyethylene	15.42	1	Infectious waste	39.17	N/A	8.7788
		Paper	10.46			15.59		5.9550
	Identification tag	High density polyethylene resin	8.21	46	Non-infectious offensive waste	0.34	See total	0.0444
**Instruments and set items**	Non-toothed lamina spreader (large)	Stainless steel	430.38	308	Scrap metal recycling	8.59		0.0298
**Total non-toothed lamina spreader (large)= 223.88 g CO_2e**						**63.68**	**145.38**	**14.81**
**Non-toothed lamina spreader (small)**								
**Sterile barrier system**	Flexible pouch	General polyethylene	11.14	1	Infectious waste	28.30	N/A	6.3421
		Paper	8.06			12.01		4.5887
	Identification tag	High density polyethylene resin	8.21	46	Non-infectious offensive waste	0.34	See total	0.0444
**Instruments and set items**	Non-toothed lamina spreader (small)	Stainless steel	130.42	225	Scrap metal recycling	3.56		0.0123
**Total non-toothed lamina spreader (small)= 200.58 g CO_2e**						**44.21**	**145.38**	**10.99**
**Semb bone holding forceps (small)**								
**Sterile barrier system**	Flexible pouch	General polyethylene	8.3	1	Infectious waste	21.08	N/A	4.7253
		Paper	5.21			7.76		2.9661
	Identification tag	High density polyethylene resin	8.21	46	Non-infectious offensive waste	0.34	See total	0.0444
**Instruments and set items**	Semb bone holding forceps	Stainless steel	159.08	186	Scrap metal recycling	5.25		0.0182
**Total Semb bone holding forceps = 187.57 g CO_2e**						**34.44**	**145.38**	**7.75**

Supplementary Table 7: Carbon footprint of reusable (non-set) and single-use items used for knee arthroplasty

Table includes all single-use items used across all observed operations listed here and the carbon footprint per item as listed. Items used for individual operations varied, alongside number of items used. The material composition of items was determined through packaging and manufacturer information where available, and alternatively through expert assessment, taking into account available emission factors. Weight is total for a given item (accounting for multiple items where relevant). 'Use' equates to linen laundering of reusable linens. Carbon footprint per use equates to a single use of an item in a single operation. CO_2e= carbon dioxide equivalents. Ops.= operations

Item	Component	Material(s)	Weight (g), or cost where specified with (£)	Number of uses	Number of ops per use	Waste stream A	Waste stream B	Carbon footprint per use (g CO_2e)					
								Production	Use	Waste stream A	Waste stream B	Total (waste A)	Total (waste B)
**Reusable personal protective equipment**													
Reusable scrubs	Product	Cotton fabric	421.17	75	3	Clothing recycling	N/A	12.69	64.65	0.04	N/A	77.39	N/A
**Non-set reusable equipment**													
Shaver base	Product	High density polyethylene resin	38.15	1300	1	Infectious waste	N/A	0.06	N/A	0.02	N/A	0.07	N/A
		Aluminium cast	35.43					0.18	N/A	0.02	N/A	0.20	N/A
		Lithium ion battery	35.43			Batteries recycling		0.17	N/A	0.00	N/A	0.17	N/A
**Single-use personal protective equipment from 'Knee pack' set**													
Gown	Product	Polypropylene oriented film	260.60	1	1	Infectious waste	N/A	893.87	N/A	148.37	N/A	1,042.24	N/A
		Rubber	13.72					39.09	N/A	7.81	N/A	46.90	N/A
Knee pack packaging (equipment component)	Packaging	Paper	14.33	1	1	Infectious waste	N/A	21.35	N/A	8.16	N/A	29.51	N/A
		Polyethylene terephthalate	5.20					20.96	N/A	2.96	N/A	23.92	N/A

		Polypropylene oriented film	0.55					1.87	N/A	0.31	N/A	2.19	N/A
**Single-use patient and instrument table drapes from ‘Knee pack’ set**													
Patient drape (240x150 cm)	Product	Polypropylene oriented film	284.23	1	1	Infectious waste	N/A	974.91	N/A	161.82	N/A	1,136.72	N/A
		Paper	12.95					19.30	N/A	7.37	N/A	26.67	N/A
Patient drape (90 x75 cm)	Product	Polypropylene oriented film	54.79	1	1	Infectious waste	N/A	187.93	N/A	31.19	N/A	219.12	N/A
		Paper	4.63					6.90	N/A	2.64	N/A	9.53	N/A
Patient drape (extremity, 230x325 cm)	Product	Polypropylene oriented film	396.77	1	1	Infectious waste	N/A	1,360.92	N/A	225.89	N/A	1,586.81	N/A
		Rubber	14.33					40.84	N/A	8.16	N/A	49.00	N/A
Patient drape (impervious, split, 152x177 cm)	Product	Polyvinylchloride general	175.56	1	1	Infectious waste	N/A	544.24	N/A	99.95	N/A	644.18	N/A
Patient drape (Mayo cover, 75x144 cm) x2	Product	Polyvinylchloride general	90.01	1	1	Infectious waste	N/A	279.03	N/A	51.24	N/A	330.27	N/A
		Nylon (polyamide) 6 polymer	19.90					500.78	N/A	11.33	N/A	512.11	N/A
Patient drape (pouch fluid collection, 40x35 cm)	Product	Low density polyethylene film	26.10	1	1	Infectious waste	N/A	1,031.60	N/A	225.89	N/A	1,257.49	N/A
	Packaging	Paper	26.10					21.35	N/A	8.16	N/A	29.51	N/A
Patient drape (stockinette, impervious, 30x120 cm)	Product	Polyvinylchloride general	66.93	1	1	Infectious waste	N/A	279.03	N/A	51.24	N/A	330.27	N/A
		Nylon (polyamide) 6 polymer	72.06					238.55	N/A	14.86	N/A	253.41	N/A
Table drape (140x90 cm)	Product	Polyvinylchloride general	119.47	1	1	Infectious waste	N/A	207.48	N/A	38.10	N/A	245.59	N/A
		Nylon (polyamide) 6 polymer	59.73					1,091.9 3	N/A	68.01	N/A	1,159.94	N/A
	Product	Polyvinylchloride general	121.79	1	1	Infectious waste	N/A	377.54	N/A	69.33	N/A	446.87	N/A

Table drape (fan folded, 140x190 cm)		Nylon (polyamide) 6 polymer	60.89					556.53	N/A	34.67	N/A	591.20	N/A
Knee pack packaging (equipment component)	Packaging	Paper	88.32	1	1	Infectious waste	N/A	131.59	N/A	50.28	N/A	181.87	N/A
		Polyethylene terephthalate	32.04					129.20	N/A	18.24	N/A	147.45	N/A
		Polypropylene oriented film	3.37					11.55	N/A	1.92	N/A	13.47	N/A
**Single-use equipment from 'Knee pack' set**													
Bowl (250 ml) x2	Product	Polypropylene injection moulding	13.24	1	1	Infectious waste	N/A	59.45	N/A	7.54	N/A	66.99	N/A
Bowl (500 ml)	Product	Polypropylene injection moulding	18.13	1	1	Infectious waste	N/A	81.40	N/A	10.32	N/A	91.73	N/A
Cast padding bandage x2	Product	Cotton padding	47.56	1	1	Infectious waste	N/A	60.88	N/A	27.08	N/A	87.95	N/A
	Packaging	Paper	48.56					72.35	N/A	27.65	N/A	100.00	N/A
Crepe bandage x2	Product	Cotton fabric	74.84	1	1	Infectious waste	N/A	507.42	N/A	42.61	N/A	550.02	N/A
Diathermy bag	Product	Low density polyethylene film	18.47	1	1	Infectious waste	N/A	48.02	N/A	10.52	N/A	58.54	N/A
	Packaging	Paper	1.92					2.86	N/A	1.09	N/A	3.95	N/A
Diathermy tip cleaner	Product	Polyurethane flexible foam	1.65	1	1	Infectious waste	N/A	7.97	N/A	0.94	N/A	8.91	N/A
		Nylon (polyamide) 6 polymer	0.18					1.65	N/A	0.10	N/A	1.75	N/A
	Packaging	Paper	0.15					0.22	N/A	0.09	N/A	0.31	N/A
Kidney dish x3	Product	Polypropylene oriented film	28.71	1	1	Infectious waste	N/A	98.48	N/A	16.34	N/A	114.82	N/A
Light cover	Product	Low density polyethylene film	4.46	1	1	Infectious waste	N/A	11.60	N/A	2.54	N/A	14.14	N/A
Monopolar diathermy	Product	High density polyethylene resin	24.15	1	1	Medicinal contaminated sharps waste	N/A	46.60	N/A	25.94	N/A	72.54	N/A

		Polyvinylchloride general	34.31			Infectious waste		106.36	N/A	19.53	N/A	125.89	N/A
		Stainless steel	3.39			Medicinal contaminated sharps waste		20.83	N/A	3.64	N/A	24.47	N/A
		Copper	3.81			Infectious waste		14.52	N/A	2.17	N/A	16.69	N/A
	Packaging	Paper	0.27					0.40	N/A	0.15	N/A	0.56	N/A
Needle counter	Product	Acrylonitrile Butadiene Styrene	44.50	1	1	Infectious waste	N/A	167.32	N/A	25.33	N/A	192.65	N/A
		Polyurethane flexible foam	4.00					19.36	N/A	2.28	N/A	21.64	N/A
Skin marker pen	Product	High density polyethylene resin	8.45	1	1	Infectious waste	N/A	16.31	N/A	4.81	N/A	21.12	N/A
		Paper	0.45					0.67	N/A	0.26	N/A	0.93	N/A
Suction tubing	Product	Polyvinylchloride general	116.66	1	1	Infectious waste	N/A	361.65	N/A	66.42	N/A	428.06	N/A
		High density polyethylene resin	3.43					6.62	N/A	1.95	N/A	8.57	N/A
Surgical blade (10) x3	Product	Stainless steel	0.61	1	1	Medicinal contaminated sharps waste	N/A	3.75	N/A	0.66	N/A	4.40	N/A
	Packaging	Aluminium foil	0.46			Infectious waste		3.44	N/A	0.26	N/A	3.70	N/A
Swab gauze (10x7.5 cm) x5	Product	Cotton fabric	4.42	1	1	Infectious waste	N/A	29.97	N/A	2.52	N/A	32.48	N/A
Swab gauze (30x30 cm) x10	Product	Cotton fabric	18.39	1	1	Infectious waste	N/A	124.68	N/A	10.47	N/A	135.15	N/A
Towel dressing (2 in pack)	Product	Paper	21.18	1	1	Infectious waste	N/A	31.56	N/A	12.06	N/A	43.62	N/A
Tray (small)	Product	Polypropylene injection moulding	116.43	1	1	Infectious waste	N/A	522.77	N/A	66.29	N/A	589.06	N/A
Yankauer sucker	Product	Polyvinylchloride injection moulding	16.98	1	1	Infectious waste	N/A	56.03	N/A	9.67	N/A	65.70	N/A
	Packaging	Paper	37.38	1	1		N/A	55.69	N/A	21.28	N/A	76.97	N/A

Knee pack packaging (equipment component)						Infectious waste							
		Polyethylene terephthalate	13.56					54.68	N/A	7.72	N/A	62.40	N/A
		Polypropylene oriented film	1.43					4.89	N/A	0.81	N/A	5.70	N/A
**Single-use personal protective equipment**													
Gloves (non-sterile, pair)	Product	Rubber	6.45	1	1	Infectious waste	N/A	18.38	N/A	3.67	N/A	22.05	N/A
Orthopaedic hood	Product	Polypropylene oriented film	61.68	1	1	Infectious waste	N/A	211.56	N/A	35.12	N/A	246.68	N/A
		Low density polyethylene film	33.69					87.59	N/A	19.18	N/A	106.77	N/A
		Stainless steel	33.69					207.03	N/A	19.18	N/A	226.21	N/A
	Packaging	Low density polyethylene film	18.02				Non-infectious offensive waste	46.85	N/A	10.26	4.49	57.11	51.34
		Polypropylene oriented film	9.42					32.31	N/A	5.36	2.34	37.67	34.66
Sterile gloves (pair)	Product	Rubber	27.31	1	1	Infectious waste	N/A	77.83	N/A	15.55	N/A	93.38	N/A
	Packaging	General polyethylene	5.98				Non-infectious offensive waste	15.19	N/A	3.40	1.49	18.59	16.68
		Paper	5.91					8.81	N/A	3.36	1.47	12.17	10.28
Sterile gloves (two pairs)	Product	Rubber	42.14	1	1	Infectious waste	N/A	120.10	N/A	23.99	N/A	144.09	N/A
	Packaging	Paper	11.61				Non-infectious offensive waste	17.30	N/A	6.61	2.89	23.91	20.19
		General polyethylene	5.61					14.25	N/A	3.19	1.40	17.44	15.65
Sterile gloves (two pair, latex free)	Product	General polyethylene	44.68	1	1	Infectious waste	N/A	113.49	N/A	25.44	N/A	138.92	N/A
	Packaging	Paper	11.66				Non-infectious offensive waste	17.37	N/A	6.64	2.90	24.01	20.28
		General polyethylene	5.71					14.50	N/A	3.25	1.42	17.75	15.92
Surgical face mask	Product	Polypropylene oriented film	4.36	1	1	Infectious waste	N/A	14.95	N/A	2.48	N/A	17.44	N/A

Surgical face mask with eye protection	Product	Polypropylene oriented film	6.23	1	1	Infectious waste	N/A	21.37	N/A	3.55	N/A	24.92	N/A
		Low density polyethylene film	4.04					10.50	N/A	2.30	N/A	12.80	N/A
Surgical gown (including hand towels)	Product	Polypropylene oriented film	139.00	1	1	Infectious waste	N/A	476.78	N/A	79.14	N/A	555.92	N/A
		Rubber	7.32					20.85	N/A	4.17	N/A	25.02	N/A
		Paper	13.83					20.61	N/A	7.87	N/A	28.48	N/A
	Packaging	Low density polyethylene film	5.11	1	1		Non-infectious offensive waste	13.29	N/A	2.91	1.27	16.20	14.56
		Paper	25.34					37.76	N/A	14.43	6.31	52.18	44.06
Surgical hat	Product	Polypropylene oriented film	3.54	1	1	Infectious waste	N/A	12.14	N/A	2.02	N/A	14.16	N/A
Sweat bands for hood x3	Product	Polyurethane flexible foam	3.29	1	1	Infectious waste	N/A	15.92	N/A	1.87	N/A	17.80	N/A
		Polyethylene terephthalate	0.37					1.49	N/A	0.21	N/A	1.70	N/A
**Single-use patient and instrument table drapes**													
Patient drape (adhesive split sheet)	Product	Polypropylene oriented film	231.80	1	1	Infectious waste	N/A	795.07	N/A	131.97	N/A	927.04	N/A
	Packaging	Paper	8.13					12.11	N/A	4.63	N/A	16.74	N/A
		Low density polyethylene film	6.48					16.85	N/A	3.69	N/A	20.54	N/A
		Polypropylene oriented film	6.46					22.16	N/A	3.68	N/A	25.84	N/A
Patient drape (clear U drape)	Product	Low density polyethylene film	115.94	1	1	Infectious waste	N/A	301.44	N/A	66.01	N/A	367.45	N/A
	Packaging	Low density polyethylene film	18.15					47.19	N/A	10.33	N/A	57.52	N/A
		Paper	7.67					11.43	N/A	4.37	N/A	15.79	N/A
Patient drape (incise drape)	Product	Low density polyethylene film	36.52	1	1	Infectious waste	N/A	94.95	N/A	20.79	N/A	115.74	N/A
	Packaging	Paper	71.62					106.71	N/A	40.77	N/A	147.49	N/A

		General polyethylene	8.69					22.07	N/A	4.95	N/A	27.02	N/A
Patient drape (incise drape, with iodine)	Product	Low density polyethylene film	23.30	1	1	Infectious waste	N/A	60.58	N/A	13.27	N/A	73.85	N/A
		Iodine	£0.04					7.91	N/A	1.47	N/A	9.38	N/A
	Packaging	Paper	52.17					77.73	N/A	29.70	N/A	107.43	N/A
		General polyethylene	15.89					40.36	N/A	9.05	N/A	49.41	N/A
Table drape (instruments)	Product	Polyvinylchloride general	375.06	1	1	Infectious waste	N/A	1162.69	N/A	213.53	N/A	1376.21	N/A
	Packaging	Paper	12.67					18.88	N/A	7.21	N/A	26.09	N/A
		Nylon (polyamide) 6 polymer	8.56					78.24	N/A	4.87	N/A	83.11	N/A
		Low density polyethylene film	6.02					15.65	N/A	3.43	N/A	19.08	N/A
**Single-use equipment and medical devices**													
Adhesive operative towel	Product	Nylon (polyamide) 6 polymer	20.41	1	1	Infectious waste	N/A	186.55	N/A	11.62	N/A	198.17	N/A
		Polyvinylchloride general	20.41					63.27	N/A	11.62	N/A	74.89	N/A
	Packaging	Low density polyethylene film	9.25					24.05	N/A	5.27	N/A	29.32	N/A
		Paper	3.36					5.01	N/A	1.91	N/A	6.92	N/A
Batteries for knee navigation set (x3)	Product	Lithium ion battery	9.81	1	1	Batteries recycling	N/A	61.88	N/A	0.21	N/A	62.09	N/A
		Aluminium cast	1.09					7.32	N/A	0.02	N/A	7.35	N/A
	Packaging	Low density polyethylene film	4.19			Infectious waste		10.89	N/A	2.39	N/A	13.28	N/A
		Polypropylene oriented film	3.16					10.84	N/A	1.80	N/A	12.64	N/A
	Product	Cotton padding	10.67	1	1	Infectious waste	N/A	13.66	N/A	6.07	N/A	19.73	N/A

Border dressing (10x30 cm)		Low density polyethylene film	0.56					1.46	N/A	0.32	N/A	1.78	N/A
	Packaging	Paper	3.22					4.80	N/A	1.83	N/A	6.63	N/A
		Polypropylene oriented film	2.90					9.95	N/A	1.65	N/A	11.60	N/A
		Low density polyethylene film	2.53					6.58	N/A	1.44	N/A	8.02	N/A
Border dressing (6x8 cm)	Product	Cotton padding	1.68	1	1	Infectious waste	N/A	2.15	N/A	0.96	N/A	3.11	N/A
		Low density polyethylene film	0.09					0.23	N/A	0.05	N/A	0.28	N/A
	Packaging	Paper	1.07					1.59	N/A	0.61	N/A	2.20	N/A
		Low density polyethylene film	0.86					2.24	N/A	0.49	N/A	2.73	N/A
		Polypropylene oriented film	0.73					2.50	N/A	0.42	N/A	2.92	N/A
Catheter tip syringe	Product	High density polyethylene resin	28.03	1	1	Infectious waste	N/A	54.10	N/A	15.96	N/A	70.06	N/A
	Packaging	Low density polyethylene film	2.16					5.62	N/A	1.23	N/A	6.85	N/A
		Paper	1.07					1.59	N/A	0.61	N/A	2.20	N/A
Cement mixing and delivery system	Product	High density polyethylene resin	214.15	1	1	Infectious waste	N/A	413.31	N/A	121.92	N/A	535.23	N/A
		Polyvinylchloride injection moulding	37.04					122.23	N/A	21.09	N/A	143.32	N/A
		Low density polyethylene resin	24.73					51.44	N/A	14.08	N/A	65.52	N/A
		Glass reinforced plastic	13.10					106.11	N/A	7.46	N/A	113.57	N/A
	Packaging	General purpose polystyrene	98.78					338.82	N/A	56.24	N/A	395.05	N/A
		Polypropylene oriented film	11.82					40.54	N/A	6.73	N/A	47.27	N/A
		Low density polyethylene film	4.74					12.32	N/A	2.70	N/A	15.02	N/A
Cement mixing bowl	Product	Glass reinforced plastic	164.54	1	1	Infectious waste	N/A	1,332.77	N/A	93.67	N/A	1,426.45	N/A

		Polyvinylchloride general	36.69					113.74	N/A	20.89	N/A	134.63	N/A
		High density polyethylene resin	26.19					50.55	N/A	14.91	N/A	65.46	N/A
	Packaging	General purpose polystyrene	81.14					278.31	N/A	46.19	N/A	324.50	N/A
		Low density polyethylene film	18.26					47.48	N/A	10.40	N/A	57.87	N/A
		Polypropylene oriented film	6.76					23.19	N/A	3.85	N/A	27.04	N/A
Crepe bandage (15 cm wide)	Product	Cotton fabric	77.55	1	1	Infectious waste	N/A	525.79	N/A	44.15	N/A	569.94	N/A
	Packaging	Paper	5.66					8.43	N/A	3.22	N/A	11.66	N/A
		Polypropylene oriented film	3.57					12.25	N/A	2.03	N/A	14.28	N/A
		Low density polyethylene film	3.19					8.29	N/A	1.82	N/A	10.11	N/A
Cruciate retaining femoral implant	Product	Titanium	251.99	1	1	N/A	N/A	5,190.99	N/A	0.00	N/A	5,190.99	N/A
	Packaging	Paper	101.71			Domestic waste		151.55	N/A	17.47	N/A	169.02	N/A
		Polyethylene terephthalate	30.83			Infectious waste		124.32	N/A	17.55	N/A	141.87	N/A
		Polypropylene oriented film	2.50					8.58	N/A	1.42	N/A	10.00	N/A
		Low density polyethylene resin	2.41					5.01	N/A	1.37	N/A	6.38	N/A
Diathermy bag	Product	Low density polyethylene film	18.47	1	1	Infectious waste	N/A	48.02	N/A	10.52	N/A	58.54	N/A
	Packaging	Low density polyethylene film	4.03					10.48	N/A	2.29	N/A	12.77	N/A
		Paper	1.92					2.86	N/A	1.09	N/A	3.95	N/A
Diathermy tip	Product	Stainless steel	1.58	1	1	Medicinal contaminated sharps waste	N/A	9.72	N/A	1.70	N/A	11.42	N/A
		High density polyethylene resin	0.53					1.02	N/A	0.57	N/A	1.58	N/A
	Packaging	Low density polyethylene film	0.90			Infectious waste		2.34	N/A	0.51	N/A	2.85	N/A

		Paper	0.59					0.88	N/A	0.34	N/A	1.21	N/A
Diathermy tip cleaner	Product	Aluminium foil	5.49	1	1	Infectious waste	N/A	41.01	N/A	3.13	N/A	44.14	N/A
		General polyethylene	5.49					13.94	N/A	3.13	N/A	17.07	N/A
	Packaging	Polyethylene terephthalate	1.84					7.42	N/A	1.05	N/A	8.47	N/A
Elasticated fabric dressing strip	Product	Rubber	0.65	1	1	Infectious waste	N/A	1.85	N/A	0.37	N/A	2.22	N/A
Gauze (individual piece)	Product	Cotton fabric	5.06	1	1	Infectious waste	N/A	34.31	N/A	2.88	N/A	37.19	N/A
Gauze (sterile pack)	Product	Cotton fabric	25.52	1	1	Infectious waste	N/A	173.03	N/A	14.53	N/A	187.55	N/A
	Packaging	Paper	5.12					7.63	N/A	2.91	N/A	10.54	N/A
		Low density polyethylene resin	1.28					2.66	N/A	0.73	N/A	3.39	N/A
High-vacuum wound drainage	Product	General plastics	124.43	1	1	Infectious waste	N/A	411.86	N/A	70.84	N/A	482.70	N/A
		Polyvinylchloride general	35.26					109.31	N/A	20.07	N/A	129.38	N/A
		Stainless steel	13.87					85.23	N/A	7.90	N/A	93.13	N/A
		High density polyethylene resin	5.14					9.92	N/A	2.93	N/A	12.85	N/A
	Packaging	Low density polyethylene film	7.14					18.56	N/A	4.06	N/A	22.63	N/A
		Paper	2.35					3.50	N/A	1.34	N/A	4.84	N/A
Incontinence pad	Product	Low density polyethylene film	40.01	1	1	Infectious waste	N/A	104.03	N/A	22.78	N/A	126.80	N/A
		Cotton padding	40.01					51.21	N/A	22.78	N/A	73.99	N/A
Intravenous infusion giving set	Product	High density polyethylene resin	13.60	1	1	Infectious waste	N/A	26.25	N/A	7.74	N/A	33.99	N/A
		Low density polyethylene resin	27.49					57.18	N/A	15.65	N/A	72.83	N/A

	Packaging	Low density polyethylene film	2.16					5.62	N/A	1.23	N/A	6.85	N/A
		Paper	1.53					2.28	N/A	0.87	N/A	3.15	N/A
Marker pen and ruler	Product	High density polyethylene resin	6.27	1	1	Infectious waste	N/A	12.10	N/A	3.57	N/A	15.67	N/A
		Paper	0.59					0.88	N/A	0.34	N/A	1.21	N/A
	Packaging	Paper	1.85					2.76	N/A	1.05	N/A	3.81	N/A
		Low density polyethylene film	0.95					2.47	N/A	0.54	N/A	3.01	N/A
Monopolar diathermy	Product	Polyvinylchloride general	34.31	1	1	Medicinal contaminated sharps waste	N/A	46.60	N/A	25.94	N/A	72.54	N/A
		High density polyethylene resin	24.15					11.82	N/A	4.09	N/A	15.91	N/A
		Copper	3.81					14.52	N/A	4.09	N/A	18.62	N/A
		Stainless steel	3.39					20.83	N/A	3.64	N/A	24.47	N/A
	Packaging	Low density polyethylene film	3.61			Infectious waste		5.38	N/A	2.06	N/A	7.43	N/A
		Paper	3.27					6.80	N/A	1.86	N/A	8.66	N/A
Nail scrubbing brush	Product	Low density polyethylene resin	9.01	1	1	Infectious waste	N/A	18.74	N/A	5.13	N/A	23.87	N/A
		Polyurethane flexible foam	2.05					9.92	N/A	1.17	N/A	11.09	N/A
		Polypropylene injection moulding	0.91					4.09	N/A	0.52	N/A	4.60	N/A
	Packaging	Low density polyethylene film	1.31					3.41	N/A	0.75	N/A	4.15	N/A
		Paper	0.60					0.89	N/A	0.34	N/A	1.24	N/A
Needle (green)	Product Packaging	Stainless steel	0.20	1	1	Medicinal contaminated sharps waste	N/A	1.23	N/A	0.21	N/A	1.44	N/A
		Polypropylene injection moulding	0.05					0.22	N/A	0.05	N/A	0.28	N/A
		High density polyethylene resin	0.52			Infectious waste		1.00	N/A	0.30	N/A	1.30	N/A

		Low density polyethylene film	0.12					0.31	N/A	0.07	N/A	0.38	N/A
		Paper	0.11					0.16	N/A	0.06	N/A	0.23	N/A
Needle (red)	Product	Stainless steel	0.41	1	1	Medicinal contaminated sharps waste	N/A	2.51	N/A	0.44	N/A	2.95	N/A
		Polypropylene injection moulding	0.10					0.46	N/A	0.11	N/A	0.57	N/A
	Packaging	High density polyethylene resin	0.60			Infectious waste		1.16	N/A	0.34	N/A	1.50	N/A
		Low density polyethylene film	0.12					0.31	N/A	0.07	N/A	0.38	N/A
		Paper	0.11					0.16	N/A	0.06	N/A	0.23	N/A
Needle (white)	Product	Stainless steel	0.29	1	1	Medicinal contaminated sharps waste	N/A	1.77	N/A	0.31	N/A	2.08	N/A
		Polypropylene injection moulding	0.07					0.32	N/A	0.08	N/A	0.40	N/A
	Packaging	High density polyethylene resin	0.60			Infectious waste		1.16	N/A	0.34	N/A	1.50	N/A
		Low density polyethylene film	0.15					0.39	N/A	0.09	N/A	0.48	N/A
		Paper	0.10					0.15	N/A	0.06	N/A	0.21	N/A
Posterior stabilised femoral implant	Product	Titanium	412.32	1	1	N/A	N/A	8,493.79	N/A	0.00	N/A	8,493.79	N/A
	Packaging	Paper	101.71			Domestic waste		151.55	N/A	17.47	N/A	169.02	N/A
		Polyethylene terephthalate	30.83			Infectious waste		124.32	N/A	17.55	N/A	141.87	N/A
		Polypropylene oriented film	2.50					8.58	N/A	1.42	N/A	10.00	N/A
		Low density polyethylene resin	2.41					5.01	N/A	1.37	N/A	6.38	N/A
Pre-operative adhesive glove	Product	Low density polyethylene film	2.60	1	1	Infectious waste	N/A	6.76	N/A	1.48	N/A	8.24	N/A
	Packaging	Paper	1.73					2.58	N/A	0.98	N/A	3.56	N/A
	Product	Titanium	129.59	1	1	N/A	N/A	2,669.55	N/A	0.00	N/A	2,669.55	N/A

Primary tibial baseplate implant	Packaging	Paper	101.71			Domestic waste		151.55	N/A	17.47	N/A	169.02	N/A
		Polyethylene terephthalate	30.83			Infectious waste		124.32	N/A	17.55	N/A	141.87	N/A
		Polypropylene oriented film	2.50					8.58	N/A	1.42	N/A	10.00	N/A
		Low density polyethylene resin	2.41					5.01	N/A	1.37	N/A	6.38	N/A
Pulsed lavage system	Product	Acrylonitrile Butadiene Styrene	302.68	1	1	Infectious waste		1,138.08	N/A	172.32	N/A	1,310.40	N/A
		Polyvinylchloride general	282.35					875.28	N/A	160.74	N/A	1,036.02	N/A
		Lithium ion battery	124.77			Batteries recycling		787.05	N/A	2.66	N/A	789.71	N/A
		Copper	5.76			Infectious waste		21.95	N/A	3.28	N/A	25.23	N/A
	Packaging	Polyethylene terephthalate	116.06					468.00	N/A	66.07	N/A	534.07	N/A
		Polypropylene oriented film	7.80					26.75	N/A	4.44	N/A	31.19	N/A
Saw blade	Product	Stainless steel	23.82	1	1	Medicinal contaminated sharps waste	N/A	146.37	N/A	25.59	N/A	171.96	N/A
	Packaging	Low density polyethylene film	1.81			Infectious waste		4.71	N/A	1.03	N/A	5.74	N/A
		General polyethylene	1.81					4.60	N/A	1.03	N/A	5.63	N/A
		Polypropylene oriented film	0.80					2.74	N/A	0.46	N/A	3.20	N/A
Self-adherent bandage	Product	Polyurethane flexible foam	57.58	1	1	Infectious waste	N/A	278.69	N/A	32.78	N/A	311.47	N/A
	Packaging	Paper	11.09					16.52	N/A	6.31	N/A	22.84	N/A
		Low density polyethylene film	2.49					6.47	N/A	1.42	N/A	7.89	N/A
Shaver head	Product	Stainless steel	2.90	1	1	Medicinal contaminated sharps waste	N/A	17.82	N/A	3.11	N/A	20.94	N/A
		Acrylonitrile Butadiene Styrene	2.76					10.36	N/A	2.96	N/A	13.32	N/A

		Low density polyethylene resin	2.76					5.73	N/A	2.96	N/A	8.69	N/A
	Packaging	Polyethylene terephthalate	0.77			Infectious waste		3.10	N/A	0.44	N/A	3.54	N/A
		Paper	0.23					0.34	N/A	0.13	N/A	0.47	N/A
Skin stapler	Product	Acrylonitrile Butadiene Styrene	51.50	1	1	Medicinal contaminated sharps waste	N/A	193.63	N/A	55.32	N/A	248.95	N/A
		Aluminium cast	4.58					30.76	N/A	4.92	N/A	35.68	N/A
		Stainless steel	1.14					7.03	N/A	1.23	N/A	8.26	N/A
	Packaging	Polyethylene terephthalate	15.00			Infectious waste	N/A	60.49	N/A	8.54	N/A	69.03	N/A
		Polypropylene oriented film	1.55					5.32	N/A	0.88	N/A	6.20	N/A
Sticky label	Product	Paper	0.40	1	1	Infectious waste	N/A	0.60	N/A	0.23	N/A	0.82	N/A
		Paper	0.25					0.37	N/A	0.14	N/A	0.51	N/A
Suction receptacle	Product	Low density polyethylene resin	72.62	1	1	Infectious waste	N/A	151.04	N/A	41.34	N/A	192.38	N/A
Suction tip	Product	High density polyethylene resin	18.68	1	1	Infectious waste	N/A	36.05	N/A	10.63	N/A	46.69	N/A
	Packaging	Paper	2.19					3.26	N/A	1.25	N/A	4.51	N/A
		Low density polyethylene film	2.17					5.64	N/A	1.24	N/A	6.88	N/A
Suction tubing	Product	Polyvinylchloride injection moulding	121.21	1	1	Infectious waste	N/A	399.99	N/A	69.01	N/A	469.00	N/A
	Packaging	Low density polyethylene film	5.40					14.04	N/A	3.07	N/A	17.11	N/A
		Paper	3.31					4.93	N/A	1.88	N/A	6.82	N/A
Surgical blade (10)	Product	Stainless steel	0.61	1	1	Medicinal contaminated sharps waste	N/A	3.75	N/A	0.66	N/A	4.40	N/A
	Packaging	Aluminium foil	0.46			Infectious waste		3.44	N/A	0.26	N/A	3.70	N/A
	Product	Stainless steel	0.25	1	1		N/A	1.54	N/A	0.27	N/A	1.80	N/A

Suture (braided, absorbable, 2-0)						Medicinal contaminated sharps waste							
		Nylon (polyamide) 6 polymer	0.23					2.10	N/A	0.25	N/A	2.35	N/A
	Packaging	High density polyethylene resin	3.19			Infectious waste		6.16	N/A	1.82	N/A	7.98	N/A
		Aluminium foil	1.25					9.34	N/A	0.71	N/A	10.05	N/A
		Low density polyethylene resin	0.47					0.98	N/A	0.27	N/A	1.25	N/A
		Polypropylene oriented film	0.60					2.06	N/A	0.34	N/A	2.40	N/A
Suture (monofilament, absorbable, 3-0, 643)	Product	Stainless steel	0.03	1	1	Medicinal contaminated sharps waste	N/A	0.18	N/A	0.03	N/A	0.22	N/A
		Nylon (polyamide) 6 polymer	0.05					0.46	N/A	0.05	N/A	0.51	N/A
	Packaging	Aluminium foil	1.33			Infectious waste		9.94	N/A	0.76	N/A	10.69	N/A
		Paper	0.88					1.31	N/A	0.50	N/A	1.81	N/A
		Low density polyethylene resin	0.50					1.04	N/A	0.28	N/A	1.32	N/A
		Polypropylene oriented film	0.61					2.09	N/A	0.35	N/A	2.44	N/A
Suture (monofilament, absorbable 3-0, 696)	Product	Stainless steel	0.03	1	1	Medicinal contaminated sharps waste	N/A	0.18	N/A	0.03	N/A	0.22	N/A
		Nylon (polyamide) 6 polymer	0.05					0.46	N/A	0.05	N/A	0.51	N/A
	Packaging	Aluminium foil	1.33			Infectious waste		9.94	N/A	0.76	N/A	10.69	N/A
		Paper	0.88					1.31	N/A	0.50	N/A	1.81	N/A
		Low density polyethylene resin	0.50					1.04	N/A	0.28	N/A	1.32	N/A
		Polypropylene oriented film	0.61					2.09	N/A	0.35	N/A	2.44	N/A
Suture (braided, absorbable 1-0, 803)	Product	Stainless steel	0.25	1	1	Medicinal contaminated sharps waste	N/A	1.54	N/A	0.27	N/A	1.80	N/A
		Nylon (polyamide) 6 polymer	0.23					2.10	N/A	0.25	N/A	2.35	N/A

	Packaging	High density polyethylene resin	3.19			Infectious waste		6.16	N/A	1.82	N/A	7.98	N/A
		Aluminium foil	1.25					9.34	N/A	0.71	N/A	10.05	N/A
		Low density polyethylene resin	0.47					0.98	N/A	0.27	N/A	1.25	N/A
		Polypropylene oriented film	0.60					2.06	N/A	0.34	N/A	2.40	N/A
Suture (braided, absorbable 1-0, 932)	Product	stainless steel	0.25	1	1	Medicinal contaminated sharps waste	N/A	1.54	N/A	0.27	N/A	1.80	N/A
		Nylon (polyamide) 6 polymer	0.02					0.21	N/A	0.02	N/A	0.23	N/A
	Packaging	High density polyethylene resin	3.19			Infectious waste		6.16	N/A	1.82	N/A	7.98	N/A
		Aluminium foil	1.25					9.34	N/A	0.71	N/A	10.05	N/A
		Low density polyethylene resin	0.47					0.98	N/A	0.27	N/A	1.25	N/A
		Polypropylene oriented film	0.60					2.06	N/A	0.34	N/A	2.40	N/A
Swab tray (large)	Product	Polyethylene terephthalate	58.32	1	1	Infectious waste	N/A	235.17	N/A	33.20	N/A	268.37	N/A
Swab tray (small)	Product	Polyethylene terephthalate	27.90	1	1	Infectious waste	N/A	112.50	N/A	15.88	N/A	128.39	N/A
Symmetric patella implant	Product	Titanium	10.70	1	1	N/A	N/A	220.42	N/A	0.00	N/A	220.42	N/A
	Packaging	Paper	71.41			Domestic waste		106.40	N/A	12.27	N/A	118.67	N/A
		General plastics	33.35			Infectious waste		110.39	N/A	18.99	N/A	129.38	N/A
		Aluminium foil	4.87					36.38	N/A	2.77	N/A	39.15	N/A
		Low density polyethylene resin	1.84					3.83	N/A	1.05	N/A	4.87	N/A
Syringe (20 ml)	Product	Polypropylene injection moulding	7.10	1	1	Infectious waste	N/A	31.90	N/A	4.04	N/A	35.94	N/A
		General polyethylene	7.10					18.04	N/A	4.04	N/A	22.09	N/A
		Rubber	0.59					1.69	N/A	0.34	N/A	2.02	N/A

	Packaging	Low density polyethylene film	1.11					2.89	N/A	0.63	N/A	3.52	N/A
		Paper	0.72					1.07	N/A	0.41	N/A	1.48	N/A
Syringe (50 ml)	Product	Polypropylene injection moulding	15.06	1	1	Infectious waste	N/A	67.63	N/A	8.58	N/A	76.21	N/A
		General polyethylene	15.06					38.26	N/A	8.58	N/A	46.83	N/A
		Rubber	1.26					3.58	N/A	0.71	N/A	4.29	N/A
	Packaging	Low density polyethylene film	2.35					6.11	N/A	1.34	N/A	7.45	N/A
		Paper	1.04					1.55	N/A	0.59	N/A	2.14	N/A
Tibial bearing insert cruciate retaining implant	Product	Titanium	36.33	1	1	N/A	N/A	748.40	N/A	0.00	N/A	748.40	N/A
	Packaging	Paper	91.30			Domestic waste		136.04	N/A	15.68	N/A	151.72	N/A
		General plastics	21.23			Infectious waste		70.27	N/A	12.09	N/A	82.36	N/A
		Aluminium foil	3.22					24.05	N/A	1.83	N/A	25.89	N/A
		Low density polyethylene resin	2.13					4.43	N/A	1.21	N/A	5.64	N/A
Tibial bearing insert posterior stabilised implant	Product	Titanium	40.97	1	1	N/A	N/A	843.98	N/A	0.00	N/A	843.98	N/A
	Packaging	Paper	91.30			Domestic waste		136.04	N/A	15.68	N/A	151.72	N/A
		General plastics	21.23			Infectious waste		70.27	N/A	12.09	N/A	82.36	N/A
		Aluminium foil	3.22					24.05	N/A	1.83	N/A	25.89	N/A
		Low density polyethylene resin	2.13					4.43	N/A	1.21	N/A	5.64	N/A
Tourniquet pressure cuff (leg)	Product	Polyvinylchloride general	165.14	1	1	Infectious waste	N/A	511.92	N/A	94.01	N/A	605.93	N/A
		High density polyethylene resin	136.95					264.30	N/A	77.96	N/A	342.27	N/A
	Packaging	Low density polyethylene film	10.19					26.49	N/A	5.80	N/A	32.30	N/A

Transparent film adhesive dressing	Product	Low density polyethylene film	0.62	1	1	Infectious waste	N/A	1.61	N/A	0.35	N/A	1.96	N/A
	Packaging	Paper	4.11					6.12	N/A	2.34	N/A	8.46	N/A
		Low density polyethylene film	1.45					3.77	N/A	0.83	N/A	4.60	N/A
Tubular support bandage	Product	Cotton fabric	29.22	1	1	Infectious waste	N/A	198.11	N/A	16.64	N/A	214.75	N/A
Wound closure strips	Product	General polyethylene	0.66	1	1	Infectious waste	N/A	1.68	N/A	0.38	N/A	2.05	N/A
	Packaging	Paper	1.49					2.22	N/A	0.85	N/A	3.07	N/A
		Low density polyethylene film	0.74					1.92	N/A	0.42	N/A	2.35	N/A
**Pharmaceuticals**													
Adrenaline 1mg in 1 ml (1 ml)	Product	Adrenaline	1.00	1	1	N/A	N/A	34.00	N/A	N/A	N/A	34.00	N/A
	Packaging	Glass	1.00			Medicinal contaminated sharps waste		1.38	N/A	1.07	N/A	2.45	N/A
Bone cement mix (with gentamicin)	Product and packaging	Bone cement (with gentamicin)	£68.28	1	1	N/A	N/A	13,082.34	N/A	N/A	N/A	13,082.34	N/A
	(Glass packaging-weight for waste)		11.61			Medicinal contaminated sharps waste		N/A	N/A	12.47	N/A	12.47	N/A
	(Non-glass packaging-weight for waste)		189.65			Infectious waste		N/A	N/A	107.97	N/A	107.97	N/A
Bone cement mix (with tobramycin)	Product and packaging	Bone cement (with tobramycin)	£68.60	1	1	N/A	N/A	13,143.65	N/A	N/A	N/A	13,143.65	N/A
	(Glass packaging-weight for waste)		9.30			Medicinal contaminated sharps waste		N/A	N/A	9.99	N/A	9.99	N/A

	(Non-glass packaging- weight for waste)		104.75			Infectious waste		N/A	N/A	59.64	N/A	59.64	N/A
Chlorhexidine 2% from 500 ml bottle (150 ml)	Product	Chlorhexidine 2%	£0.84	1	1	N/A	N/A	160.94	N/A	N/A	N/A	160.94	N/A
Chlorhexidine gluconate in 70% denatured ethanol with 4 ml red stain solution from 200 ml bottle (150 ml)	Product	Chlorhexidine gluconate in 70% denatured ethanol with 4 ml red stain solution	£5.13	1	1	N/A	N/A	982.90	N/A	N/A	N/A	982.90	N/A
Iodinated povidone 10% w/w alcoholic tincture from 500 ml bottle (50 ml)	Product	Iodinated povidone 10% w/w alcoholic tincture	£0.48	1	1	N/A	N/A	92.16	N/A	N/A	N/A	92.16	N/A
Ketorolac tromethamine 30mg in 1 ml (1 ml)	Product	Ketorolac tromethamine 30mg in 1 ml	£1.36	1	1	N/A	N/A	260.57	N/A	N/A	N/A	260.57	N/A
	(Packaging - weight for waste)		1.48			Medicinal contaminated sharps waste		N/A	N/A	1.59	N/A	1.59	N/A
Levobupivacaine 2.5mg/ml (10 ml)	Product	Levobupivacaine	10.00	1	1	N/A	N/A	230.00	N/A	N/A	N/A	230.00	N/A
	Packaging	Low density polyethylene resin	2.65			Infectious waste		5.51	N/A	1.51	N/A	7.02	N/A
		Low density polyethylene film	0.46					1.20	N/A	0.26	N/A	1.46	N/A
		Paper	0.40					0.60	N/A	0.23	N/A	0.82	N/A
Ropivacaine 75mg in 10 ml (10 ml)	Product	Ropivacaine hydrochloride	10.00	1	1	Infectious waste	N/A	360.00	N/A	5.69	N/A	365.69	N/A
	Packaging	Polyethylene terephthalate	2.56					10.32	N/A	1.46	N/A	11.78	N/A

		Polypropylene injection moulding	2.41					10.82	N/A	1.37	N/A	12.19	N/A
Sodium chloride 0.9% (100 ml)	Product	Sodium chloride 0.9%	£0.59	1	1	N/A	N/A	113.04	N/A	N/A	N/A	113.04	N/A
	(Packaging - weight for waste)		5.16			Infectious waste		N/A	N/A	2.94	N/A	2.94	N/A
Sodium chloride 0.9% for irrigation (3 L bag)	Product	Sodium chloride 0.9%	£4.52	1	1	N/A	N/A	866.02	N/A	N/A	N/A	866.02	N/A
	(Packaging - weight for waste)		91.13			Infectious waste		N/A	N/A	51.88	N/A	51.88	N/A
Topical skin adhesive (0.8 g)	Product	Topical skin adhesive (0.8 g)	£20.47	1	1	N/A	N/A	3,922.02	N/A	N/A	N/A	3,922.02	N/A
	(Packaging - weight for waste)		4.38			Infectious waste		N/A	N/A	2.49	N/A	2.49	N/A
**Cleaning products, waste**													
Anatomical waste bin	Product	Paper	81.22	1	1	Anatomical waste	N/A	121.02	N/A	87.24	N/A	208.26	N/A
Clear bin bag (linen laundering)	Product	Low density polyethylene film	13.91	1	3	Domestic waste	N/A	12.06	N/A	0.80	N/A	12.85	N/A
		Nylon (polyamide) 6 polymer	2.58					7.86	N/A	0.15	N/A	8.01	N/A
Clear waste bag (recycling) with cable tie	Product	Low density polyethylene film	15.01	1	1	Domestic waste	N/A	39.03	N/A	2.58	N/A	41.60	N/A
		Nylon (polyamide) 6 polymer	2.58					23.58	N/A	0.44	N/A	24.02	N/A
Disinfectant sachet	Product	Disinfectant	£0.70	1	1	N/A	N/A	134.12	N/A	N/A	N/A	134.12	N/A
	(Packaging - weight for waste)		5.81			Infectious waste		N/A	N/A	3.31	N/A	3.31	N/A
Disinfectant wipe	Product	Non-woven polyester	2.30	1	1	Infectious waste	N/A	12.79	N/A	1.31	N/A	14.10	N/A
Orange waste bag (infectious waste) with	Product	Polypropylene oriented film	70.61	1	1	Infectious waste	N/A	242.19	N/A	40.20	N/A	282.39	N/A
		Nylon (polyamide) 6 polymer	2.58					23.58	N/A	1.47	N/A	25.05	N/A

cable tie from theatre													
Orange waste bag (infectious waste) with cable tie from scrub room	Product	Polypropylene oriented film	70.61	1	3	Infectious waste	N/A	80.73	N/A	13.40	N/A	94.13	N/A
		Nylon (polyamide) 6 polymer	2.58					7.86	N/A	0.49	N/A	8.35	N/A
Mop head	Product	Nylon (polyamide) 6 polymer	70.36	1	3	Infectious waste	N/A	214.36	N/A	13.35	N/A	227.72	N/A
		High density polyethylene resin	30.16					19.40	N/A	5.72	N/A	25.13	N/A
Red bag (linen laundering) with cable tie	Product	Polypropylene oriented film	29.22	1	3	Domestic waste	N/A	33.41	N/A	1.67	N/A	35.08	N/A
		Nylon (polyamide) 6 polymer	2.58					7.86	N/A	0.15	N/A	8.01	N/A
Yellow/ black waste bag (non-infectious offensive waste) with cable tie from scrub room	Product	Polypropylene oriented film	24.93	1	3	Non-infectious offensive waste	N/A	28.50	N/A	2.07	N/A	30.57	N/A
		Nylon (polyamide) 6 polymer	2.58					7.86	N/A	0.21	N/A	8.07	N/A

Supplementary Table 8: Carbon footprint of reusable instrument sets used for laparoscopic cholecystectomy

Table includes all reusable instrument sets and individually wrapped instruments across all observed operations, and the carbon footprint per set. Sets and individually wrapped instruments used for individual operations varied. The material composition of items was determined through packaging and manufacturer information where available, and alternatively through expert assessment, taking into account available emission factors. Weight is total for a given item (accounting for multiple items where relevant). Some items listed as ‘bottom tray’/’top tray’ where not individually counted by theatre staff. ‘Use’ equates to decontamination of instruments, determined per set. All waste of given item disposed of using consistent waste stream (A). CO_2e= carbon dioxide equivalents

Category	Item	Material(s)	Weight (g)	Number of uses	Waste stream A	Carbon footprint per use (g CO_2e)		
						Production	Use	Waste stream A
**General basic set**								
**Sterile barrier system**	Basket	Stainless steel	1170.57	116	Scrap metal recycling	62.01	See total	0.2149
	Container	Aluminium cast	2996.54	1,000	Scrap metal recycling	20.14		0.0638
	Filter paper	Paper	3.55	1	Infectious waste	5.29	N/A	2.0211
	Identification tag	High density polyethylene resin	8.21	46	Non-infectious offensive waste	0.34	See total	0.0444
	Kit list	Paper	4.85	1	Infectious waste	7.23	N/A	2.7612
	Tamper proof tags	General plastics	1.78	1	Infectious waste	5.89		1.0134
**Instruments and set items**	Allis tissue forceps x2	Stainless steel	56.92	4,476	Scrap metal recycling	0.08	See total	0.0003
	Babcock tissue forceps x2	Stainless steel	48.50	4,476	Scrap metal recycling	0.07		0.0002
	Bag plain closure (H)	Paper	13.69	1	Infectious waste	20.40	N/A	7.7939
	Bipolar diathermy	Stainless steel	31.98	40	Medicinal contaminated sharps waste	4.91	See total	0.8588
		High density polyethylene resin	31.98			1.54		0.8588
	Bonney toothed dissecting forceps	Stainless steel	61.07	4,029	Scrap metal recycling	0.09		0.0003

	BP scalpel handle (no. 3) x2	Stainless steel	49.40	895	Scrap metal recycling	0.34		0.0012
	BP scalpel handle (no. 4) x2	Stainless steel	57.44	895	Scrap metal recycling	0.39		0.0014
	Bulldog clip	Stainless steel	49.77	1,343	Scrap metal recycling	0.23		0.0008
	Czerny retractor x2	Stainless steel	123.94	5,372	Scrap metal recycling	0.14		0.0005
	Debakey dissecting forceps	Stainless steel	21.42	1,343	Scrap metal recycling	0.10		0.0003
	Diathermy lead	Polyvinylchloride general	86.52	50	Non-infectious offensive waste	5.36		0.4307
		Copper	9.61			0.73		0.0478
	Diathermy quiver	Polypropylene injection moulding	96.13	1,343	Non-infectious offensive waste	0.32		0.0178
	Gallipot x3	Polypropylene injection moulding	14.10	1	Infectious waste	63.31	N/A	8.0273
	Gillies toothed dissecting forceps	Stainless steel	24.31	1,074	Scrap metal recycling	0.14	See total	0.0005
	Halstead mosquito curved artery forceps x6	Stainless steel	122.76	2,686	Scrap metal recycling	0.28		0.0010
	Kidney dish (25 cm) x4	Polypropylene injection moulding	395.52	895	Non-infectious offensive waste	1.98		0.1100
	Lanes tissue forceps x2	Stainless steel	84.32	4,476	Scrap metal recycling	0.12		0.0004
	Langenbeck retractor (medium) x2	Stainless steel	105.10	6,267	Scrap metal recycling	0.10		0.0004
	Mayo curved scissors	Stainless steel	70.12	895	Scrap metal recycling	0.48		0.0017
	Mayo Hegar needle holder x2	Stainless steel	58.24	1,791	Scrap metal recycling	0.20		0.0007
	Mayo straight scissors	Stainless steel	44.34	1,343	Scrap metal recycling	0.20		0.0007
	McIndoe dissecting forceps	Stainless steel	20.69	2,238	Scrap metal recycling	0.06		0.0002
	McIndoe plain diathermy forceps	Stainless steel	32.49	40	Medicinal contaminated sharps waste	4.99		0.8725
		Nylon (polyamide) 6 polymer	3.61			0.82		0.0969

	McIndoe scissors	Stainless steel	34.01	895	Scrap metal recycling	0.23		0.0008
	Plain dissecting forceps (5")	Stainless steel	23.09	40	Scrap metal recycling	3.55		0.0123
	Plain dissecting forceps (7")	Stainless steel	37.01	895	Scrap metal recycling	0.25		0.0009
	Rampley sponge holder forceps x4	Stainless steel	256.56	1,343	Scrap metal recycling	1.17		0.0041
	Schnidt artery forceps x6	Stainless steel	238.56	4,029	Scrap metal recycling	0.36		0.0013
	Spencer Wells curved artery forceps x4	Stainless steel	209.52	3,581	Scrap metal recycling	0.36		0.0012
	Spencer Wells straight artery forceps x6	Stainless steel	146.46	4,476	Scrap metal recycling	0.20		0.0007
	Stitch scissor	Stainless steel	32.74	4,476	Scrap metal recycling	0.04		0.0002
	Towel clip x6	Stainless steel	211.86	1,343	Scrap metal recycling	0.97		0.0034
	Travers self retaining retractor	Stainless steel	107.66	895	Scrap metal recycling	0.74		0.0026
	Treves toothed dissecting forceps x6	Stainless steel	141.12	1,970	Scrap metal recycling	0.44		0.0015
**Total general basic set (B)= 2,395.86g CO_2e**						**216.62**	**2,153.96**	**25.27**
**General laparoscopic set**								
**Sterile barrier system**	Container base (deep)	Aluminium cast	2,629.88	116	Scrap metal recycling	17.67	See total	0.0560
	Container lid (deep)	General plastic	1,659.99	1,000	Non-infectious offensive waste	5.49		0.4132
	Filter paper	Paper	3.55	1	Infectious waste	5.29	N/A	2.0211
	Identification tag	High density polyethylene resin	12.13	46	Non-infectious offensive waste	0.51	See total	0.0656
	Kit list	Paper	4.85	1	Infectious waste	7.23	N/A	2.7612
	Laparoscopic instrument rack	Stainless steel	937.84	500	Scrap metal recycling	11.53	See total	0.0399
		Rubber	165.50			0.94		0.0070

	Tamper proof tags	General plastics	1.78	1	Infectious waste	5.89	N/A	1.0134
**Instruments and set items**	Desjardin forceps	Stainless steel	33.64	2028.40	Scrap metal recycling	0.10	See total	0.0004
	Diathermy hook	stainless steel	30.64	500.00	Scrap metal recycling	0.38		0.0013
	Diathermy lead	Polyvinylchloride general	94.88	50.00	Non-infectious offensive waste	5.88		0.4724
		Copper	10.54			0.80		0.0525
	Johann grasping forceps	Polypropylene injection moulding	58.51	500.00	Medicinal contaminated sharps waste	0.53		0.1257
		Stainless steel	26.26			0.32		0.0564
	Kelly crocodile grasping forceps x2	Polypropylene injection moulding	117.48	500.00	Medicinal contaminated sharps waste	1.05		0.2524
		Stainless steel	58.74			0.72		0.1262
	Kocher artery forceps x2	Stainless steel	93.42	2353.50	Scrap metal recycling	0.24		0.0008
	Lahey right angle dissecting forceps	Polypropylene injection moulding	52.34	500.00	Medicinal contaminated sharps waste	0.47		0.1124
		Stainless steel	26.17			0.32		0.0562
	Langenbeck retractor (small) x2	Stainless steel	132.86	3549.70	Scrap metal recycling	0.23		0.0008
	Littlewoods tissue forceps x2	Stainless steel	80.96	2353.50	Scrap metal recycling	0.21		0.0007
	Manhes grasping forceps	Polypropylene injection moulding	59.37	500.00	Medicinal contaminated sharps waste	0.53		0.1275
		Stainless steel	29.69			0.36		0.0638
	Maryland dissecting forceps	Polypropylene injection moulding	52.06	500.00	Medicinal contaminated sharps waste	0.47		0.1118
		Stainless steel	26.03			0.32		0.0559
	Pietlyn dissecting forceps	Polypropylene injection moulding	57.89	500.00	Medicinal contaminated sharps waste	0.52		0.1244
		Stainless steel	28.94			0.36		0.0622

	Raptor toothed grasping forceps	Polypropylene injection moulding	77.99	500.00	Medicinal contaminated sharps waste	0.70		0.1675
		Stainless steel	39.00			0.48		0.0838
**Total general laparoscopic set= 2,231.95g CO_2e**						**69.56**	**2,153.96**	**8.43**
**Laparoscope set**								
**Sterile barrier system**	Identification tag	High density polyethylene resin	13.72	46	Non-infectious offensive waste	0.58	See total	0.0742
	Kit list	Paper	4.85	1	Infectious waste	7.23	N/A	2.7612
	Tray wrap (outer, 100x100 cm)	Paper	110.88	1	Infectious waste	165.21		63.13
	Tray wrap (inner, 100x100 cm)	Polypropylene oriented film	111.10	1	Infectious waste	381.07		63.25
	Wire cage with lid	Stainless steel	892.10	500	Scrap metal recycling	10.96	See total	0.0380
**Instruments and set items**	Light lead	Polypropylene injection moulding	208.38	400	Non-infectious offensive waste	2.34		0.1297
		Stainless steel	104.19			1.60		0.0648
		Glass general	104.19			0.38		0.0648
	Hopkins laparoscope (10 mm, 0 degree)	Stainless steel	151.15	500	Non-infectious offensive waste	1.86		0.0753
		Polypropylene injection moulding	26.67			0.24		0.0133
		Glass general	8.89			0.03		0.0044
**Total laparoscope set = 1,778.07 g CO_2e**						**571.49**	**1,076.89**	**129.60**
**Diathermy lead**								
**Sterile barrier system**	Flexible pouch	General polyethylene	11.14	1	Infectious waste	28.30	N/A	6.3421
		Paper	8.06			12.01		4.5887

	Identification tag	High density polyethylene resin	8.21	46	Non-infectious offensive waste	0.34	See total	0.0444
**Instruments and set items**	Diathermy lead	Polyvinylchloride general	94.88	50	Non-infectious offensive waste	5.88		0.4724
		Copper	10.54			0.80		0.0525
**Total diathermy lead= 204.22 g CO_2e**						**47.34**	**145.38**	**11.50**
**Laparoscopic grasping forceps**								
**Sterile barrier system**	Flexible pouch	General polyethylene	15.42	1	Infectious waste	39.17	N/A	8.7788
		Paper	10.46			15.59		5.9550
	Identification tag	High density polyethylene resin	8.21	46	Non-infectious offensive waste	0.34	See total	0.0444
**Instruments and set items**	Laparoscopic grasping forceps	Polypropylene injection moulding	59.37	500	Medicinal contaminated sharps waste	0.53		0.1275
		Stainless steel	29.69			0.36		0.0638
**Total laparoscopic grasping forceps = 216.35 g CO_2e**						**55.99**	**145.38**	**14.97**
**Quiver and clip**								
**Sterile barrier system**	Flexible pouch	General polyethylene	11.14	1	Infectious waste	28.30	N/A	6.3421
		Paper	8.06			12.01		4.5887
	Identification tag	High density polyethylene resin	8.21	46	Non-infectious offensive waste	0.34	See total	0.0444
**Instruments and set items**	Quiver	Polypropylene injection moulding	279.75	1,342.89	Non-infectious offensive waste	0.94		0.0519
	Clip	Stainless steel	18.97	1,342.89	Scrap metal recycling	0.09		0.0003
**Quiver and clip= 198.08 g CO_2e**						**41.67**	**145.38**	**11.03**

Supplementary Table 9: Carbon footprint of reusable (non-set) and single-use items used for laparoscopic cholecystectomy
Table includes all single-use items used across all observed operations listed here and the carbon footprint per item as listed. Items used for individual operations varied, alongside number of items used. The material composition of items was determined through packaging and manufacturer information where available, and alternatively through expert assessment, taking into account available emission factors. Weight is total for a given item (accounting for multiple items where relevant). 'Use' equates to linen laundering of reusable linens. Carbon footprint per use equates to a single use of an item in a single operation. CO_2e= carbon dioxide equivalents. Ops.= operations

Item	Component	Material(s)	Weight (g), or cost where specified with (£), or volume where specified	Number of uses	Number of ops per use	Waste stream A	Waste stream B	Carbon footprint per use (g CO_2e)					
								Production	Use	Waste stream A	Waste stream B	Total (waste A)	Total (waste B)
**Reusable patient drapes**													
High fluid drape	Product	Polyethylene terephthalate	1876.3	75	1	Clothing recycling	N/A	100.88	864.09	0.53	N/A	965.51	N/A
	Packaging	Paper	51.53	1		Infectious waste	Domestic waste	76.78	N/A	29.34	8.85	106.12	85.63
		Nylon (polyamide) 6 polymer	2.76					25.23	N/A	1.57	0.47	26.80	25.70
Huck towel	Product	Cotton fabric	120.55	75	1	Clothing recycling	N/A	10.90	55.52	0.03	N/A	66.45	N/A
	Packaging	Paper	20.63	1		Infectious waste	Domestic waste	30.74	N/A	11.74	3.54	42.48	34.28
		Low density polyethylene film	4.6					11.96	N/A	2.62	0.79	14.58	12.75
**Reusable personal protective equipment**													
Reusable surgical gown (including hand towels)	Product	Polyethylene terephthalate	303.70	75	1	Clothing recycling	N/A	16.33	139.86	0.09	N/A	156.28	N/A
		Rubber	15.98					0.61	7.36	0.0045	N/A	7.97	N/A

		Paper	13.41	1		Domestic waste		19.98	N/A	2.30	N/A	22.28	N/A
	Packaging	Paper	19.90					29.65	N/A	3.42	N/A	33.07	N/A
		General polyethylene	6.42					29.65	N/A	3.42	N/A	33.07	N/A
Reusable surgical gown (including hand towels) double pack	Product	Polyethylene terephthalate	555.48	75	1	Clothing recycling	N/A	29.87	255.82	0.16	N/A	285.84	N/A
		Rubber	29.24					1.11	13.46	0.01	N/A	14.58	N/A
		Paper	24.57	1		Domestic waste		36.61	N/A	4.22	N/A	40.83	N/A
	Packaging	Paper	24.31					36.22	N/A	4.18	N/A	40.40	N/A
		General polyethylene	8.81					22.38	N/A	1.51	N/A	23.89	N/A
Reusable surgical hat	Product	Cotton fabric	26.58	75	4	Clothing recycling	N/A	0.60	3.06	0.0019	N/A	3.66	N/A
Reusable scrubs	Product	Cotton fabric	421.17	75	4	Clothing recycling	N/A	9.52	48.49	0.0299	N/A	58.04	N/A
**Non-set reusable equipment**													
Diathermy pad lead	Product	Polyvinylchloride general	121.57	50	1	Non-infectious offensive waste	N/A	7.54	N/A	0.61	N/A	8.14	N/A
		Copper	13.51					1.03	N/A	0.07	N/A	1.10	N/A
**Single-use personal protective equipment**													
Gloves (non-sterile, pair)	Product	Rubber	6.45	1	1	Infectious waste	Non-infectious offensive waste	18.38	N/A	3.67	1.61	22.05	19.99
Sterile gloves (pair)	Product	Rubber	27.31	1	1	Infectious waste	Non-infectious offensive waste	77.83	N/A	15.55	6.80	93.38	84.63
	Packaging	General polyethylene	5.98			Domestic waste	N/A	15.19	N/A	1.03	N/A	16.22	N/A

		Paper	5.91					8.81	N/A	1.02	N/A	9.82	N/A
Sterile gloves (two pairs)	Product	Rubber	42.14	1	1	Infectious waste	Non-infectious offensive waste	120.10	N/A	23.99	10.49	144.09	130.59
	Packaging	Paper	11.61			Domestic waste	N/A	17.30	N/A	1.99	N/A	19.29	N/A
		General polyethylene	5.61					14.25	N/A	0.96	N/A	15.21	N/A
Sterile gloves (one pair, latex free)	Product	General polyethylene	24.66	1	1	Infectious waste	Non-infectious offensive waste	62.64	N/A	14.04	6.14	76.68	68.78
	Packaging	Paper	6.01			Domestic waste	N/A	8.95	N/A	1.03	N/A	9.99	N/A
		General polyethylene	5.83					14.81	N/A	1.00	N/A	15.81	N/A
Surgical face mask	Product	Polypropylene oriented film	4.36	1	1	Infectious waste	Non-infectious offensive waste	14.95	N/A	2.48	1.09	17.44	16.04
Surgical face mask with eye protection	Product	Polypropylene oriented film	6.23	1	1	Infectious waste	Non-infectious offensive waste	21.37	N/A	3.55	1.55	24.92	22.92
		Low density polyethylene film	4.04					10.50	N/A	2.30	1.01	12.80	11.51
Surgical gown (including hand towels)	Product	Polypropylene oriented film	139.004	1	1	Infectious waste	Non-infectious offensive waste	476.78	N/A	79.14	34.60	555.92	511.39
		Rubber	7.316					20.85	N/A	4.17	1.82	25.02	22.67
		Paper	13.83			Domestic waste	N/A	20.61	N/A	2.38	N/A	22.98	N/A
	Packaging	Paper	25.34					37.76	N/A	4.35	N/A	42.11	N/A
		Low density polyethylene film	5.11					13.29	N/A	0.88	N/A	14.16	N/A
Surgical hat	Product	Polypropylene oriented film	3.54	1	4	Infectious waste	Non-infectious offensive waste	3.04	N/A	0.50	0.22	3.54	3.26

**Single-use instrument table drapes**													
Table drape (instruments)	Product	Polyvinylchloride general	122.17	1	1	Infectious waste	Non-infectious offensive waste	378.73	N/A	69.55	30.41	448.28	409.14
		Polypropylene oriented film	61.09					209.54	N/A	34.78	15.21	244.32	224.75
	Packaging	Paper	12.64				Domestic waste	18.83	N/A	7.20	2.17	26.03	21.00
		Polypropylene oriented film	9.05					31.04	N/A	5.15	1.55	36.19	32.60
		Low density polyethylene film	6.49					16.87	N/A	3.69	1.11	20.57	17.99
**Single-use equipment and medical devices**													
Absorbent towel pack	Product	Paper	28.47	1	1	Infectious waste	N/A	42.42	N/A	16.21	N/A	58.63	N/A
	Packaging	Paper	5					7.45	N/A	2.85	N/A	10.30	N/A
		Low density polyethylene film	2.16					5.62	N/A	1.23	N/A	6.85	N/A
Anti-fog endoscopic demister	Product	Anti-fog	£3.5	1	1	N/A	N/A	670.59	N/A	N/A	N/A	670.59	670.59
	(Product only weight-for waste)		5.47			Infectious waste	Non-infectious offensive waste	N/A	N/A	3.11	1.36	3.11	1.36
	(Packaging weight-for waste)		15.2				Domestic waste	N/A	N/A	8.65	2.61	8.65	2.61
Diathermy pad	Product	Aluminium foil	5.49	1	1	Infectious waste	Non-infectious offensive waste	41.01	N/A	3.13	1.37	44.14	42.38
		General polyethylene	5.49					13.94	N/A	3.13	1.37	17.07	15.31
	Packaging	Polyethylene terephthalate	1.84				Domestic waste	7.42	N/A	1.05	0.32	8.47	7.74
Endoscopic clip applier	Product	Stainless steel	64.13	1	1		N/A	394.08	N/A	68.88	N/A	462.96	N/A

		Polypropylene injection moulding	24.91			Medicinal contaminated sharps waste		111.85	N/A	26.76	N/A	138.60	N/A
		Polycarbonate	19.83					151.10	N/A	21.30	N/A	172.40	N/A
		Polyvinylchloride general	6.71					20.80	N/A	7.21	N/A	28.01	N/A
		Nylon (polyamide) 6 polymer	0.4					3.66	N/A	0.43	N/A	4.09	N/A
		Titanium	0.08					1.65	N/A	0.09	N/A	1.73	N/A
	Packaging	Paper	178.64			Infectious waste	Domestic waste	266.17	N/A	101.70	30.69	367.88	296.86
		Polyethylene terephthalate	97.18					391.87	N/A	55.33	16.69	447.19	408.56
		High density polyethylene resin	9.39					18.12	N/A	5.35	1.61	23.47	19.74
Gauze (sterile pack)	Product	Cotton fabric	19.66	1	1	Infectious waste	Non-infectious offensive waste	133.29	N/A	11.19	4.89	144.49	138.19
	Packaging	Paper	4.73				Domestic waste	7.05	N/A	2.69	0.81	9.74	7.86
		Low density polyethylene film	1.54					4.00	N/A	0.88	0.26	4.88	4.27
Incontinence pad	Product	Cotton padding	15.09	1	1	Infectious waste	Non-infectious offensive waste	19.32	N/A	8.59	3.76	27.91	23.07
		Low density polyethylene film	15.09					39.23	N/A	8.59	3.76	47.82	42.99
Insufflating tubing	Product	Polyvinylchloride general	124.23	1	1	Infectious waste	Non-infectious offensive waste	385.11	N/A	70.73	30.92	455.84	416.04
		Polypropylene injection moulding	20.86					93.66	N/A	11.88	5.19	105.54	98.85
	Packaging	Low density polyethylene film	9.94				Domestic waste	25.84	N/A	5.66	1.71	31.50	27.55
		Paper	6.72					10.01	N/A	3.83	1.15	13.84	11.17
		Polyvinylchloride general	5.60					17.36	N/A	3.19	0.96	20.55	18.32
Laparoscope cover	Product	Low density polyethylene film	19.55	1	1	Infectious waste	Non-infectious	50.83	N/A	11.13	4.87	61.96	55.70

		Polyvinylchloride general	18.69				offensive waste	57.94	N/A	10.64	4.65	68.58	62.59
		Paper	2.06					3.07	N/A	1.17	0.51	4.24	3.58
	Packaging	Paper	2.62				Domestic waste	3.90	N/A	1.49	0.45	5.40	4.35
		Low density polyethylene film	1.97					5.12	N/A	1.12	0.34	6.24	5.46
Laparoscopic scissors	Product	Stainless steel	27.23	1	1	Medicinal contaminated sharps waste	N/A	167.33	N/A	29.25	N/A	196.58	N/A
		Polycarbonate	26.68					203.30	N/A	28.66	N/A	231.96	N/A
		Silicone	5.53					18.47	N/A	5.94	N/A	24.41	N/A
		General plastics	0.54					1.79	N/A	0.58	N/A	2.37	N/A
		Copper	0.3					1.14	N/A	0.32	N/A	1.47	N/A
		Zinc	0.3					1.25	N/A	0.32	N/A	1.58	N/A
		Nickel	0.3					3.72	N/A	0.32	N/A	4.04	N/A
	Packaging	Nylon (polyamide) 6 polymer	14.01			Infectious waste	Domestic waste	128.05	N/A	7.98	2.41	136.03	130.46
		High density polyethylene resin	14.01					27.04	N/A	7.98	2.41	35.02	29.45
		Paper	8.28					12.34	N/A	4.71	1.42	17.05	13.76
		Polypropylene oriented film	4.4					15.09	N/A	2.50	0.76	17.60	15.85
Laparoscopic tissue retrieval system	Product	Nylon (polyamide) 6 polymer	2.37	1	1	Infectious waste	Non-infectious offensive waste	21.66	N/A	1.35	0.59	23.01	22.25
	Packaging	Low density polyethylene film	3.55				Domestic waste	9.23	N/A	2.02	0.61	11.25	9.84
		Paper	3.02					4.50	N/A	1.72	0.52	6.22	5.02
Light handle	Product	Low density polyethylene film	4.78	1	1	Infectious waste	Non-infectious	12.43	N/A	2.72	1.19	15.15	13.62

							offensive waste						
	Packaging	Low density polyethylene film	3.38				Domestic waste	8.79	N/A	1.92	0.58	10.71	9.37
		Paper	1.93					2.88	N/A	1.10	0.33	3.97	3.21
Nail scrubbing brush	Product	Low density polyethylene resin	9.01	1	1	Domestic waste	N/A	18.74	N/A	1.55	N/A	20.29	N/A
		Polyurethane flexible foam	2.05					9.92	N/A	0.35	N/A	10.27	N/A
		Polypropylene injection moulding	0.91					4.09	N/A	0.16	N/A	4.24	N/A
	Packaging	Low density polyethylene film	1.31					3.41	N/A	0.23	N/A	3.63	N/A
		Paper	0.6					0.89	N/A	0.10	N/A	1.00	N/A
Needle (green)	Product	Stainless steel	0.2	1	1	Medicinal contaminated sharps waste	N/A	1.23	N/A	0.21	N/A	1.44	N/A
		Polypropylene injection moulding	0.05					0.22	N/A	0.05	N/A	0.28	N/A
	Packaging	High density polyethylene resin	0.52			Infectious waste	Domestic waste	1.00	N/A	0.30	0.09	1.30	1.09
		Low density polyethylene film	0.12					0.31	N/A	0.07	0.02	0.38	0.33
		Paper	0.11					0.16	N/A	0.06	0.02	0.23	0.18
Needle counter	Product	Acrylonitrile Butadiene Styrene	44.50	1	1	Medicinal contaminated sharps waste	N/A	167.32	N/A	47.80	N/A	215.12	N/A
		Polyurethane rigid foam	4.00					17.04	N/A	4.30	N/A	21.34	N/A
	Packaging	Low density polyethylene film	4.38			Infectious waste	Domestic waste	11.39	N/A	2.49	0.75	13.88	12.14
		Polypropylene oriented film	1.15					3.94	N/A	0.65	0.20	4.60	4.14
Nonwoven dressing (6x7 cm)	Product	Cotton fabric	0.42	1	1	Infectious waste	Non-infectious offensive waste	2.83	N/A	0.24	0.10	3.07	2.94
		Low density polyethylene film	0.02					0.05	N/A	0.01	0.005	0.06	0.06
	Packaging	Paper	1.15				Domestic waste	1.71	N/A	0.65	0.20	2.37	1.91

		Low density polyethylene film	1.06					2.76	N/A	0.60	0.18	3.36	2.94
Port (12 mm)	Product	Polycarbonate	58.84	1	1	Medicinal contaminated sharps waste	N/A	448.36	N/A	63.20	N/A	511.56	N/A
		Polypropylene injection moulding	6.29					28.24	N/A	6.76	N/A	35.00	N/A
		Silicone	3.86					12.89	N/A	4.15	N/A	17.04	N/A
		Stainless steel	1.29					7.93	N/A	1.39	N/A	9.31	N/A
		Low density polyethylene resin	1.6					3.33	N/A	1.72	N/A	5.05	N/A
	Packaging	Nylon (polyamide) 6 polymer	5.09			Infectious waste	Domestic waste	46.52	N/A	2.90	0.87	49.42	47.40
		High density polyethylene resin	8.29					16.00	N/A	4.72	1.42	20.72	17.42
		Low density polyethylene film	0.46					1.20	N/A	0.26	0.08	1.46	1.28
Port (5 mm, dual pack)	Product	Polycarbonate	59.83	1	1	Medicinal contaminated sharps waste	N/A	455.90	N/A	64.27	N/A	520.17	N/A
		Polypropylene injection moulding	7.38					33.14	N/A	7.93	N/A	41.06	N/A
		Silicone	4.36					14.56	N/A	4.68	N/A	19.25	N/A
		Low density polyethylene resin	1.42					2.95	N/A	1.53	N/A	4.48	N/A
	Packaging	Nylon (polyamide) 6 polymer	5.29			Infectious waste	Domestic waste	48.35	N/A	3.01	0.91	51.36	49.26
		High density polyethylene resin	8.54					16.48	N/A	4.86	1.47	21.34	17.95
Pressure saline infusion bag	Product	Rubber	45.52	1	1	Infectious waste	N/A	129.73	N/A	25.92	N/A	155.65	N/A
		Nylon (polyamide) 6 polymer	33.94					310.21	N/A	19.32	N/A	329.53	N/A
		High density polyethylene resin	25.16					42.21	N/A	12.45	N/A	54.66	N/A
		Acrylonitrile Butadiene Styrene	21.87					48.56	N/A	14.32	N/A	62.88	N/A
		Polyvinylchloride general	9.57					29.67	N/A	5.45	N/A	35.12	N/A

		Polypropylene injection moulding	3.63					16.30	N/A	2.07	N/A	18.37	N/A
	Packaging	Low density polyethylene film	7.25					18.85	N/A	4.13	N/A	22.98	N/A
Reinforced skin closure strip	Product	General polyethylene	0.66	1	1	Clinical waste	N/A	1.68	N/A	0.71	N/A	2.39	N/A
	Packaging	Paper	1.49			Infectious waste	Non-infectious offensive waste	2.22	N/A	0.85	0.37	3.07	2.59
		Low density polyethylene film	0.74					1.92	N/A	0.42	0.18	2.35	2.11
Specimen pot (40 ml 4% formaldehyde)	Product	4% Formaldehyde	£0.20	1	1	Clinical waste	N/A	37.63	N/A	N/A	N/A	37.63	N/A
	(Product weight-for waste)		40					N/A	N/A	42.97	N/A	42.97	N/A
	Product	Polypropylene injection moulding	6.53			Infectious waste	Non-infectious offensive waste	29.32	N/A	3.72	1.63	33.04	30.95
		High density polyethylene resin	5.19					10.02	N/A	2.95	1.29	12.97	11.31
Specimen pot (not pre-filled)	Product	Polypropylene injection moulding	28.77	1	1	Infectious waste	Non-infectious offensive waste	129.18	N/A	16.38	7.16	145.56	136.34
Suction irrigation	Product	Polyvinylchloride general	283.76	1	1	Infectious waste	Non-infectious offensive waste	879.66	N/A	161.55	70.64	1,041.20	950.29
		Polypropylene injection moulding	39.67					76.56	N/A	22.58	9.88	99.15	86.44
		High density polyethylene resin	25.96					174.45	N/A	14.78	6.46	189.23	180.91
		Aluminium cast	14.5					97.44	N/A	8.26	3.61	105.70	101.05
	Packaging	Paper	28.30				Domestic waste	73.58	N/A	16.11	4.86	89.69	78.44
		Low density polyethylene film	20.67					39.89	N/A	11.77	3.55	51.66	43.44
Suction receptacle	Product	High density polyethylene resin	72.62	1	1	Infectious waste	Non-infectious offensive waste	140.15	N/A	41.34	18.08	181.49	158.22
		Low density polyethylene resin	72.62					151.04	N/A	41.34	18.08	192.38	169.12

Surgical blade (11)	Product	Stainless steel	0.46	1	1	Medicinal contaminated sharps waste	N/A	2.83	N/A	0.49	N/A	3.32	N/A
	Packaging	Aluminium foil	0.51			Infectious waste	Domestic waste	3.81	N/A	0.29	0.09	4.10	3.90
Surgical blade (15)	Product	Stainless steel	0.39	1	1	Medicinal contaminated sharps waste	N/A	2.40	N/A	0.42	N/A	2.82	N/A
	Packaging	Aluminium foil	0.52			Infectious waste	Domestic waste	3.88	N/A	0.30	0.09	4.18	3.97
Suture (braided, absorbable, 3-0)	Product	Stainless steel	0.408	1	1	Medicinal contaminated sharps waste	N/A	2.51	N/A	0.44	N/A	2.95	N/A
		Nylon (polyamide) 6 polymer	0.102					0.93	N/A	0.11	N/A	1.04	N/A
	Packaging	High density polyethylene resin	0.6			Infectious waste	Domestic waste	1.16	N/A	0.34	0.10	1.50	1.26
		Low density polyethylene film	0.12					0.31	N/A	0.07	0.02	0.38	0.33
		Paper	0.11					0.16	N/A	0.06	0.02	0.23	0.18
Suture (monofilament, absorbable, 3-0)	Product	Stainless steel	0.26	1	1	Medicinal contaminated sharps waste	N/A	1.60	N/A	0.28	N/A	1.88	N/A
		Nylon (polyamide) 6 polymer	0.09					0.82	N/A	0.10	N/A	0.92	N/A
	Packaging	Aluminium foil	1.22			Infectious waste	Domestic waste	9.11	N/A	0.69	0.21	9.81	9.32
		Paper	0.91					1.36	N/A	0.52	0.16	1.87	1.51
		Polypropylene oriented film	0.52					1.78	N/A	0.30	0.09	2.08	1.87
		Low density polyethylene film	0.5					1.30	N/A	0.28	0.09	1.58	1.39
Suture (monofilament, absorbable, 1)	Product	Stainless steel	0.26	1	1	Medicinal contaminated sharps waste	N/A	1.60	N/A	0.28	N/A	1.88	N/A
		Nylon (polyamide) 6 polymer	0.09					0.82	N/A	0.10	N/A	0.92	N/A
	Packaging	Aluminium foil	1.22			Infectious waste	Domestic waste	9.11	N/A	0.69	0.21	9.81	9.32
		Paper	0.91					1.36	N/A	0.52	0.16	1.87	1.51

		Polypropylene oriented film	0.52					1.78	N/A	0.30	0.09	2.08	1.87
		Low density polyethylene film	0.5					1.30	N/A	0.28	0.09	1.58	1.39
Syringe (10 ml)	Product	Polypropylene injection moulding	3.01	1	1	Infectious waste	Non-infectious offensive waste	13.51	N/A	1.71	0.75	15.23	14.26
		General polyethylene	3.01					7.65	N/A	1.71	0.75	9.36	8.39
		Rubber	0.25					0.71	N/A	0.14	0.06	0.85	0.77
	Packaging	Low density polyethylene film	0.45				Domestic waste	1.17	N/A	0.26	0.08	1.43	1.25
		Paper	0.39					0.58	N/A	0.22	0.07	0.80	0.65
Syringe (20 ml)	Product	Polypropylene injection moulding	7.10	1	1	Infectious waste	Non-infectious offensive waste	31.90	N/A	4.04	1.77	35.94	33.67
		General polyethylene	7.10					18.04	N/A	4.04	1.77	22.09	19.81
		Rubber	0.59					1.69	N/A	0.34	0.15	2.02	1.83
	Packaging	Low density polyethylene film	1.11				Domestic waste	2.89	N/A	0.63	0.19	3.52	3.08
		Paper	0.72					1.07	N/A	0.41	0.12	1.48	1.20
Tonsil swab pack	Product	Cotton fabric	5.81	1	1	Infectious waste	Non-infectious offensive waste	39.39	N/A	3.31	1.45	42.70	40.84
	Packaging	Paper	1.92				Domestic waste	2.86	N/A	1.09	0.33	3.95	3.19
		Low density polyethylene film	0.96					2.50	N/A	0.55	0.16	3.04	2.66
**Pharmaceuticals**													
Carbon dioxide (from 3kg cylinder containing 450 L)	Product and packaging	Carbon dioxide liquid	1 litre	1	1	N/A	N/A	6.00	N/A	N/A	N/A	6.00	N/A

Chlorhexidine 1% from 500 ml container (50 ml)	Product and packaging	Chlorhexidine 2%	£0.28	1	1	N/A	N/A	53.65	N/A	N/A	N/A	53.65	N/A
Levobupivacaine 2.5mg/10 ml (10 ml)	Product	Levobupivacaine	10.00	1	1	N/A	N/A	230.00	N/A	N/A	N/A	230.00	N/A
	Packaging	Low density polyethylene resin	2.65			Infectious waste	Domestic waste	5.51	N/A	1.51	0.46	7.02	5.97
		Low density polyethylene film	0.46					1.20	N/A	0.26	0.08	1.46	1.28
		Paper	0.40					0.60	N/A	0.23	0.07	0.82	0.66
Sodium Chloride 0.9% for irrigation (1 L bag)	Product and packaging	Sodium chloride	£1.50	1	1	N/A	N/A	287.40	N/A	0.00	N/A	287.40	287.40
	(Packaging -weight for waste)		30.03			Infectious waste	Domestic waste	0.00	N/A	17.10	5.16	17.10	5.16
Sodium Chloride 0.9% from 1 litre bottle (50 ml)	Product and packaging	Sodium chloride	£0.20	1	1	N/A	N/A	37.94	N/A	N/A	N/A	37.94	N/A
**Cleaning products, waste**													
Black waste bag (domestic waste) with cable tie	Product	Polypropylene oriented film	22.37	1	1	Domestic waste	N/A	76.73	N/A	3.84	N/A	80.57	N/A
		Nylon (polyamide) 6 polymer	2.58					23.58	N/A	0.44	N/A	24.02	N/A
Clear bin bag (for diathermy quiver)	Product	Low density polyethylene film	12.48	1	1	Non-infectious offensive waste	N/A	32.45	N/A	3.11	N/A	35.55	N/A
		Nylon (polyamide) 6 polymer	2.58					23.58	N/A	0.64	N/A	24.22	N/A
Clear bin bag (recycling) with cable tie	Product	Low density polyethylene film	15.01	1	1	Domestic waste	N/A	39.03	N/A	2.58	N/A	41.60	N/A
		Nylon (polyamide) 6 polymer	2.58					23.58	N/A	0.44	N/A	24.02	N/A
Disinfectant sachet	Product	Disinfectant	£0.70	1	4	Infectious waste	N/A	33.53	N/A	N/A	N/A	33.53	N/A

	(Packaging - weight for waste)		5.81					N/A	N/A	0.83	N/A	0.83	N/A
Disinfectant wipe	Product	Non-woven polyester	3.34	1	1	Infectious waste	Clinical waste	18.57	N/A	1.90	3.59	20.47	22.16
Green bag (linen laundering) with cable tie	Product	Low density polyethylene film	16.47	1	4	Domestic waste	N/A	10.71	N/A	0.71	N/A	11.41	N/A
		Nylon (polyamide) 6 polymer	2.58					5.90	N/A	0.11	N/A	6.01	N/A
Mop head	Product	Nylon (polyamide) 6 polymer	70.36	1	4	Infectious waste	Clinical waste	160.77	N/A	10.01	18.89	170.79	179.67
		High density polyethylene resin	30.16					14.55	N/A	4.29	8.10	18.84	22.65
Orange waste bag (infectious waste, large) with cable tie	Product	Polypropylene oriented film	70.61	1	1	Infectious waste	N/A	242.19	N/A	40.20	N/A	282.39	N/A
		Nylon (polyamide) 6 polymer	2.58					23.58	N/A	1.47	N/A	25.05	N/A
Orange waste bag (infectious waste, small) with cable tie	Product	Polypropylene oriented film	27.75	1	1	Infectious waste	N/A	95.18	N/A	15.80	N/A	110.98	N/A
		Nylon (polyamide) 6 polymer	2.58					23.58	N/A	1.47	N/A	25.05	N/A
Red bag (linen laundering) with cable tie	Product	Polypropylene oriented film	29.22	1	4	Domestic waste	N/A	25.06	N/A	1.25	N/A	26.31	N/A
		Nylon (polyamide) 6 polymer	2.58					5.90	N/A	0.11	N/A	6.01	N/A
Yellow/ black waste bag (non-infectious offensive waste, large) with cable tie	Product	Polypropylene oriented film	24.93	1	1	Non-infectious offensive waste	N/A	85.51	N/A	6.21	N/A	91.72	N/A
		Nylon (polyamide) 6 polymer	2.58					23.58	N/A	0.64	N/A	24.22	N/A
Yellow/ black waste bag (non-infectious offensive waste, small) with cable tie	Product	Polypropylene oriented film	17.16	1	1	Non-infectious offensive waste	N/A	44.62	N/A	4.27	N/A	48.89	N/A
		Nylon (polyamide) 6 polymer	2.58					23.58	N/A	0.64	N/A	24.22	N/A

Supplementary Table 10: Carbon footprint of reusable instrument sets used for tonsillectomy

Table includes all reusable instrument sets used across all observed operations, and the carbon footprint per set. Sets used for individual operations varied. The material composition of items was determined through packaging and manufacturer information where available, and alternatively through expert assessment, taking into account available emission factors. Weight is total for a given item (accounting for multiple items where relevant). 'Use' equates to decontamination of instruments, determined per set. All waste of given item disposed of using consistent waste stream (A). CO_2e= carbon dioxide equivalents

Category	Item	Material(s)	Weight (g)	Number of uses	Waste stream A	Carbon footprint per use (g CO_2e)		
						Production	Use	Waste stream A
**Tonsillectomy set A**								
**Sterile barrier system**	Basket	Stainless steel	1053.09	116	Scrap metal recycling	55.79	See total	0.1933
	Container	Aluminium cast	2996.54	1,000	Scrap metal recycling	20.14		0.0638
	Filter paper	Paper	3.55	1	Infectious waste	5.29	N/A	2.0211
	Identification tag	High density polyethylene resin	8.21	46	Non-infectious offensive waste	0.34	See total	0.0444
	Kit list	Paper	4.85	1	Infectious waste	7.23	N/A	2.7612
	Tamper proof tags	General plastics	1.78	1	Infectious waste	5.89		1.0134
**Instruments and set items**	Adenoid St Clair Thompson curette	Stainless steel	90.00	2,000	Scrap metal recycling	0.28	See total	0.0010
	Beckman curette	Stainless steel	60.20	2,000	Scrap metal recycling	0.18		0.0006
	Bipolar diathermy lead	Rubber	85.94	50	Non-infectious offensive waste	4.90		0.4279
		Copper	9.55			0.73		0.0475
	Bipolar diathermy forceps (8")	Stainless steel	30.46	40	Medicinal contaminated sharps waste	4.68		0.8180
		Nylon (polyamide) 6 polymer	3.38			0.77		0.0909

	Birkett straight tonsil forceps	Stainless steel	42.26	1,348	Scrap metal recycling	0.19		0.0007
	Boyle Davis gag	Stainless steel	94.72	1,540	Scrap metal recycling	0.38		0.0013
	Boyle Davis tongue plate (64 mm)	Stainless steel	41.74	1,540	Scrap metal recycling	0.17		0.0006
	Boyle Davis tongue plate (74 mm)	Stainless steel	67.86	1,540	Scrap metal recycling	0.27		0.0009
	Boyle Davis tongue plate (89 mm)	Stainless steel	76.12	1,540	Scrap metal recycling	0.30		0.0011
	Boyle Davis tongue plate (99 mm)	Stainless steel	78.95	1,540	Scrap metal recycling	0.32		0.0011
	Bulldog clip	Stainless steel	48.76	385	Scrap metal recycling	0.78		0.0027
	Dennis Browne forceps	Stainless steel	79.55	1,540	Scrap metal recycling	0.32		0.0011
	Dissecting forceps (long, toothed)	Stainless steel	30.22	2,118	Scrap metal recycling	0.09		0.0003
	Draffin bipod stand (long) x2	Stainless steel	209.38	2,310	Scrap metal recycling	0.56		0.0019
	Draffin bipod stand (short) x2	Stainless steel	180.27	2,310	Scrap metal recycling	0.48		0.0017
	Gag guard	Low density polyethylene resin	1.98	1	Clinical waste	4.12	N/A	2.1268
	Gwyne Evans tonsil dissector	Stainless steel	31.70	2,695	Scrap metal recycling	0.07	See total	0.0003
	Hurd dissector/ pillar retractor	Stainless steel	56.31	2,695	Scrap metal recycling	0.13		0.0004
	Kidney dish (20 cm)	Polypropylene injection moulding	51.29	385	Non-infectious offensive waste	0.60		0.0332
	Luc forceps (large)	Stainless steel	59.24	1,540	Scrap metal recycling	0.24		0.0008
	Metzenbaum curved scissors	Stainless steel	41.74	809	Scrap metal recycling	0.32		0.0011
	Negus curved artery forceps (large)	Stainless steel	44.32	2,118	Scrap metal recycling	0.13		0.0004
	Negus knot pusher	Stainless steel	46.43	3,465	Scrap metal recycling	0.08		0.0003
	Wilson tonsil artery forceps	Stainless steel	34.29	2,118	Scrap metal recycling	0.10		0.0003

**Total tonsillectomy set A=2,279.47 g CO_2e**						**115.84**	**2,153.96**	**9.66**
**Tonsillectomy set B**								
**Sterile Barrier System**	Basket	Stainless steel	1245.17	116	Scrap metal recycling	65.96	See total	0.2286
	Container	Aluminium cast	2996.54	1000	Scrap metal recycling	20.14		0.0638
	Filter paper	Paper	3.55	1	Infectious waste	5.29	N/A	2.0211
	Identification tag	High density polyethylene resin	8.21	46	Non-infectious offensive waste	0.34	See total	0.0444
	Kit list	Paper	4.85	1	Infectious waste	7.23	N/A	2.7612
	Tamper proof tags	General plastics	1.78	1	Infectious waste	5.89		1.0134
**Instruments and set items**	Bag plain closure (H)	Paper	13.69	1	Infectious waste	20.40		7.7939
	Bipolar diathermy lead	Rubber	85.94	50	Non-infectious offensive waste	4.90	See total	0.4279
		Copper	9.55			0.73		0.0475
	Bipolar forceps (8")	Stainless steel	30.46	40	Medicinal contaminated sharps waste	4.68		0.8180
		Nylon (polyamide) 6 polymer	3.38	40	Medicinal contaminated sharps waste	0.77		0.0909
	Birketts straight tonsil forceps	Stainless steel	42.26	1348	Scrap metal recycling	0.19		0.0007
	Boyle Davis gag (adult)	Stainless steel	107.36	1540	Scrap metal recycling	0.43		0.0015
	Boyle Davis gag (paediatric)	Stainless steel	94.77	1540	Scrap metal recycling	0.38		0.0013
	Bulldog clip	Stainless steel	48.76	385	Scrap metal recycling	0.78		0.0027
	Diathermy quiver	Polypropylene injection moulding	96.09	770	Non-infectious offensive waste	0.56		0.0311
	Doughty tongue plate (3.5")	Stainless steel	68.72	1540	Scrap metal recycling	0.27		0.0010

	Doughty tongue plate (4")	Stainless steel	82.85	1540	Scrap metal recycling	0.33		0.0011
	Draffin bipod stand x 2	Stainless steel	192.50	2310	Scrap metal recycling	0.51		0.0018
	Gag guard	Low density polyethylene resin	3.05	1	Infectious waste	6.34	N/A	1.7364
	Gallipot (60 ml) x2	Polypropylene injection moulding	8.40	1	Infectious waste	37.72		4.7822
	Gwyne Evans tonsil dissector	Stainless steel	31.70	2695	Scrap metal recycling	0.07	See total	0.0003
	Kidney dish (25 cm) x2	Polypropylene injection moulding	98.88	385	Non-infectious offensive waste	1.15		0.0639
	Luc forceps (large)	Stainless steel	59.24	1540	Scrap metal recycling	0.24		0.0008
	Masson needle holder (10")	Stainless steel	78.95	1925	Scrap metal recycling	0.25		0.0009
	Mcindoe curved scissor (7")	Stainless steel	39.44	809	Scrap metal recycling	0.30		0.0010
	Mollison pillar retractor	Stainless steel	36.74	2118	Scrap metal recycling	0.11		0.0004
	Negus curved artery forceps (large)	Stainless steel	44.32	2118	Scrap metal recycling	0.13		0.0004
	Negus knot pusher	Stainless steel	46.43	3465	Scrap metal recycling	0.08		0.0003
	Small receiver x2	Polypropylene injection moulding	102.58	385	Non-infectious offensive waste	1.20		0.0663
	Towel clip (ball/socket)	Stainless steel	62.00	1348	Scrap metal recycling	0.28		0.0010
	Treves plain dissecting forceps (5")	Stainless steel	22.67	1925	Scrap metal recycling	0.07		0.0003
	Waugh toothed dissecting forceps (8")	Stainless steel	39.42	2118	Scrap metal recycling	0.11		0.0004
	Wilson tonsil artery forceps	Stainless steel	34.29	2118	Scrap metal recycling	0.10		0.0003
	Woods ENT scissors	Stainless steel	55.02	1540	Scrap metal recycling	0.22		0.0008
	Yankauer sucker	Stainless steel	63.92	2503	Scrap metal recycling	0.16		0.0005
**Total tonsillectomy set B=2,364.29 g CO_2e**						**188.32**	**2,153.96**	**22.01**

Supplementary Table 11: Carbon footprint of reusable (non-set) and single-use items used for tonsillectomy

Table includes all single-use items used across all observed operations listed here and the carbon footprint per item as listed. Items used for individual operations varied, alongside number of items used. The material composition of items was determined through packaging and manufacturer information where available, and alternatively through expert assessment, taking into account available emission factors. Weight is total for a given item (accounting for multiple items where relevant). 'Use' equates to linen laundering of reusable linens. Carbon footprint per use equates to a single use of an item in a single operation. CO_2e= carbon dioxide equivalents. Ops.= operations

Item	Component	Material(s)	Weight (g), or cost where specified with (£)	Number of uses	Number of ops per use	Waste stream A	Waste stream B	Carbon footprint per use (g CO_2e)					
								Production	Use	Waste stream A	Waste stream B	Total (waste A)	Total (waste B)
**Reusable patient drapes**													
Reusable ENT split head drape (two 42"x42")	Product	Polyethylene terephthalate	738.99	75	1	Clothing recycling	N/A	39.73	340.33	0.21	N/A	380.27	N/A
	Packaging	Paper	35.70	1	1	Domestic waste	Infectious waste	53.19	N/A	6.13	20.3245	59.33	73.52
		Nylon (polyamide) 6 polymer	2.20					20.11	N/A	0.38	1.2525	20.49	21.36
**Reusable personal protective equipment**													
Reusable surgical gown (including hand towels)	Product	Polyethylene terephthalate	303.70	75	1	Clothing recycling	N/A	16.33	139.86	0.09	N/A	156.28	N/A
		Rubber	15.98					0.61	7.36	0.005	N/A	7.97	N/A
		Paper	13.41	1	1	Domestic waste		19.98	N/A	2.30	N/A	22.28	N/A
	Packaging	General polyethylene	6.42					16.31	N/A	1.10	N/A	17.41	N/A
		Paper	19.90					29.65	N/A	3.42	N/A	33.07	N/A
Reusable surgical hat	Product	Cotton fabric	26.58	75	5	Clothing recycling	N/A	0.48	2.45	0.002	N/A	2.93	N/A
Reusable scrubs	Product	Cotton fabric	421.17	75	5	Clothing recycling	N/A	7.61	38.79	0.02	N/A	46.43	N/A

**Single-use personal protective equipment**													
Gloves (non-sterile, one pair)	Product	Rubber	6.45	1	1	Clinical waste	Infectious waste	18.38	N/A	6.93	3.67	25.31	22.05
Sterile gloves (one pair)	Product	Rubber	27.31	1	1	Clinical waste	Infectious waste	77.83	N/A	29.33	15.55	107.17	93.38
	Packaging	General polyethylene	5.98			Domestic waste	N/A	15.19	N/A	1.03	N/A	16.22	N/A
		Paper	5.91					8.81	N/A	1.02	N/A	9.82	N/A
Surgical face mask	Product	Polypropylene oriented film	4.36	1	1	Clinical waste	Infectious waste	14.95	N/A	4.68	2.48	19.64	17.44
Surgical face mask with eye protection	Product	Polypropylene oriented film	6.23	1	1	Clinical waste	Infectious waste	21.37	N/A	6.69	3.55	28.06	24.92
		Low density polyethylene film	4.04					10.50	N/A	4.34	2.30	14.84	12.80
Surgical hat	Product	Polypropylene oriented film	3.54	1	5	Clinical waste	Infectious waste	2.43	N/A	0.76	0.40	3.19	2.83
**Single-use patient or table drapes**													
Patient drape (fenestrated ENT drape)	Product	Low density polyethylene film	17.98	1	1	Clinical waste	N/A	46.75	N/A	19.31	N/A	66.06	N/A
		Nylon (polyamide) 6 polymer	17.98					164.34	N/A	19.31	N/A	183.65	N/A
	Packaging	Low density polyethylene film	9.03			Domestic waste	N/A	23.48	N/A	1.55	N/A	25.03	N/A
Table drape (instruments)	Product	Polyvinylchloride general	122.17	1	1	Clinical waste	N/A	378.73	N/A	131.23	69.55	509.95	448.28
		Polypropylene oriented film	61.09					209.54	N/A	65.62	34.78	275.16	244.32
	Packaging	Low density polyethylene film	6.49			Domestic waste	N/A	16.87	N/A	1.11	3.69	17.99	20.57
		Paper	12.64					18.83	N/A	2.17	7.20	21.00	26.03
		Polypropylene oriented film	9.05					31.04	N/A	1.55	5.15	32.60	36.19

**Single-use equipment and medical devices**													
Coblation™ wand	Product	Low density polyethylene resin	74.55	1	1	Clinical waste	N/A	155.06	N/A	80.08	N/A	235.14	N/A
		Polyvinylchloride general	37.27					115.54	N/A	40.03	N/A	155.57	N/A
		Polycarbonate	17.47			Medicinal contaminated sharps waste	N/A	133.12	N/A	18.77	N/A	151.89	N/A
		Stainless steel	17.47					107.35	N/A	18.77	N/A	126.12	N/A
	Packaging	Paper	56.74			Domestic waste	N/A	84.54	N/A	9.75	N/A	94.29	N/A
		General polyethylene	53.83					136.73	N/A	9.25	N/A	145.98	N/A
		Polypropylene oriented film	6.58					22.57	N/A	1.13	N/A	23.70	N/A
Kidney dish	Product	Paper	18.98	1	1	Clinical waste	N/A	28.28	N/A	20.39	N/A	48.67	N/A
	Packaging	Low density polyethylene film	5.86			Domestic waste	N/A	15.24	N/A	1.01	N/A	16.24	N/A
		Paper	4.89					7.29	N/A	0.84	N/A	8.13	N/A
Filter needle	Product	Stainless steel	0.40	1	1	Medicinal contaminated sharps waste	N/A	2.46	N/A	0.43	N/A	2.89	N/A
		Polypropylene injection moulding	0.10					0.45	N/A	0.11	N/A	0.56	N/A
	Packaging	High density polyethylene resin	0.59			Domestic waste	N/A	1.14	N/A	0.10	N/A	1.24	N/A
		Low density polyethylene film	0.23					0.60	N/A	0.04	N/A	0.64	N/A
		Paper	0.11					0.16	N/A	0.02	N/A	0.18	N/A
Gallipot	Product	Polypropylene injection moulding	6.40	1	1	Clinical waste	N/A	28.74	N/A	6.87	N/A	35.61	N/A
	Packaging	Paper	1.60			Domestic waste	N/A	2.38	N/A	0.27	N/A	2.66	N/A
		Low density polyethylene film	1.45					3.77	N/A	0.25	N/A	4.02	N/A
Incontinence pad	Product	Cotton padding	15.09	1	1	Infectious waste	N/A	19.32	N/A	8.59	N/A	27.91	N/A

		Low density polyethylene film	15.09					39.23	N/A	8.59	N/A	47.82	N/A
Braided silk tonsil ties	Product	Silk	1.15	1	1	Clinical waste	Infectious waste	41.56	N/A	1.24	0.65	42.80	42.22
	Packaging	Paper	2.01			Domestic waste	N/A	2.99	N/A	0.35	N/A	3.34	N/A
		Low density polyethylene film	0.58					1.51	N/A	0.10	N/A	1.61	N/A
Gauze (non-sterile)	Product	Cotton fabric	1.99	1	1	Clinical waste	N/A	13.49	N/A	2.14	N/A	15.63	N/A
Nail scrubbing brush	Product	Low density polyethylene resin	9.01	1	1	Domestic waste	Infectious waste	18.74	N/A	1.55	5.13	20.29	23.87
		Polyurethane flexible foam	2.05					9.92	N/A	0.35	1.17	10.27	11.09
		Polypropylene injection moulding	0.91			Domestic waste	N/A	4.09	N/A	0.16	N/A	4.24	N/A
	Packaging	Low density polyethylene film	1.31					3.41	N/A	0.23	N/A	3.63	N/A
		Paper	0.60					0.89	N/A	0.10	N/A	1.00	N/A
Suction tip	Product	Polyvinylchloride injection moulding	2.87	1	1	Clinical waste	Infectious waste	9.47	N/A	3.08	1.63	12.55	11.10
	Packaging	Low density polyethylene film	1.49			Domestic waste	Infectious waste	3.87	N/A	0.26	0.85	4.13	4.72
		Paper	1.20					1.79	N/A	0.21	0.68	1.99	2.47
Suction receptacle	Product	High density polyethylene resin	72.62	1	1	Clinical waste	Infectious waste	140.16	N/A	78.00	41.34	218.16	181.50
		Low density polyethylene resin	72.62					151.05	N/A	78.00	41.34	229.05	192.39
Suction tubing	Product	Polyvinylchloride injection moulding	121.21	1	1	Clinical waste	Infectious waste	399.99	N/A	130.20	69.01	530.19	469.00
	Packaging	Low density polyethylene film	3.08			Domestic waste	Infectious waste	8.01	N/A	0.53	1.75	8.54	9.76
		Paper	3.31					4.93	N/A	0.57	1.88	5.50	6.82
		Low density polyethylene film	2.32					6.03	N/A	0.40	1.32	6.43	7.35
Syringe (10 ml)	Product	Polypropylene injection moulding	3.01	1	1	Clinical waste	N/A	13.51	N/A	3.23	N/A	16.75	N/A

		General polyethylene	3.01					7.65	N/A	3.23	N/A	10.88	N/A
		Rubber	0.25					0.71	N/A	0.27	N/A	0.98	N/A
	Packaging	Low density polyethylene film	0.45			Domestic waste	N/A	1.17	N/A	0.08	N/A	1.25	N/A
		Paper	0.39					0.58	N/A	0.07	N/A	0.65	N/A
Tonsil swab pack	Product	Cotton fabric	5.81	1	1	Clinical waste	Infectious waste	39.39	N/A	6.24	3.31	45.63	42.70
	Packaging	Paper	1.92			Domestic waste	Infectious waste	2.86	N/A	0.33	1.09	3.19	3.95
		Low density polyethylene film	0.96					2.50	N/A	0.16	0.55	2.66	3.04
Yankauer sucker	Product	Polyvinylchloride injection moulding	18.68	1	1	Clinical waste	Infectious waste	61.64	N/A	20.06	10.63	81.71	72.28
	Packaging	Paper	2.19			Domestic waste	Infectious waste	3.26	N/A	0.38	1.25	3.64	4.51
		Low density polyethylene film	2.17					5.64	N/A	0.37	1.24	6.01	6.88
Specimen pot (40 ml 4% formaldehyde)	Product	4% Formaldehyde	£0.20	1	1	Clinical waste	N/A	37.63	N/A	N/A	N/A	37.63	N/A
	Product (weight-for waste)		40					N/A	N/A	42.97	N/A	42.97	N/A
	Packaging	Polypropylene injection moulding	6.53					29.32	N/A	7.01	N/A	36.33	N/A
		High density polyethylene resin	6.53					33.89	N/A	7.01	N/A	40.90	N/A
**Pharmaceuticals**													
Bupivacaine 0.5% with 1:200,000 adrenaline (10 ml)	Product	Bupivacaine hydrochloride	10.00	1	1	N/A	N/A	230.00	N/A	N/A	N/A	230.00	N/A
	Packaging	Glass general	6.38			Medicinal contaminated sharps waste		9.19	N/A	6.85	N/A	16.04	N/A
	Product	Bupivacaine hydrochloride	10.00	1	1	N/A	N/A	230.00	N/A	N/A	N/A	230.00	N/A

Chirocaine 2.5mg/ml (10 ml)	Packaging	High density polyethylene resin	0.55	1	1	Domestic waste		1.06	N/A	0.09	N/A	1.16	N/A
		Low density polyethylene resin	4.70					9.78	N/A	0.81	N/A	10.58	N/A
		Low density polyethylene resin	3.16			Clinical waste		6.57	N/A	3.39	N/A	9.97	N/A
Sodium chloride 0.9% from 1 litre bottle (150 ml)	Product and packaging	Sodium Chloride 0.9% from 1 litre bottle	£0.59	1	1	N/A	N/A	113.81	N/A	N/A	N/A	113.81	N/A
Sodium chloride 0.9%, intravenous infusion bag (500 ml)	Product and packaging	Sodium chloride 0.9% intravenous infusion bag (500 ml)	£1.38	1	1	Clinical waste	N/A	264.41	N/A	27.04	N/A	264.41	N/A
	Packaging (weight for waste)		25.17					N/A	N/A	27.04	N/A	27.04	N/A
Yellow soft paraffin BP 100% from 15g tube	Product	Petroleum slack wax	0.79	1	1	N/A	N/A	0.85	N/A	N/A	N/A	0.85	N/A
**Cleaning products, waste**													
Black waste bag (domestic waste) with cable tie	Product	Polypropylene oriented film	22.37	1	1	Domestic waste	N/A	76.73	N/A	3.84	N/A	80.57	N/A
		Nylon (polyamide) 6 polymer	2.58					23.58	N/A	0.44	N/A	24.02	N/A
Clear waste bag (for swab count)	Product	Low density polyethylene film	22.78	1	1	Clinical waste	Infectious waste	59.23	N/A	24.47	12.97	83.70	72.20
Clear waste bag (recycling) with cable tie	Product	Low density polyethylene film	15.01	1	1	Domestic waste	N/A	39.03	N/A	2.58	N/A	41.60	N/A
		Nylon (polyamide) 6 polymer	2.58					23.58	N/A	0.44	N/A	24.02	N/A
Disinfectant sachet	Product	Disinfectant	£0.70	1	5	Domestic waste	N/A	26.82	N/A	N/A	N/A	26.82	N/A
	Packaging (weight for waste)		5.81					N/A	N/A	0.20	N/A	0.20	N/A

Disinfectant wipe	Product	Non-woven polyester	2.30	1	1	Clinical waste	Infectious waste	12.79	N/A	2.47	1.31	15.26	14.10
Green bag (linen laundering) with cable tie	Product	Low density polyethylene film	29.22	1	5	Domestic waste	N/A	15.19	N/A	1.00	N/A	16.20	N/A
		Nylon (polyamide) 6 polymer	2.58					4.72	N/A	0.09	N/A	4.80	N/A
Mop head	Product	Nylon (polyamide) 6 polymer	70.36	1	5	Clinical waste	Infectious waste	128.62	N/A	15.12	8.01	143.73	136.63
		High density polyethylene resin	30.16					11.64	N/A	6.48	3.43	18.12	15.08
Orange waste bag (infectious waste) with cable tie	Product	Polypropylene oriented film	70.61	1	1	Infectious waste	N/A	242.19	N/A	40.20	N/A	282.39	N/A
		Nylon (polyamide) 6 polymer	2.58					23.58	N/A	1.47	N/A	25.05	N/A
Yellow waste bag (clinical waste) with cable tie	Product	Polypropylene oriented film	28.15	1	1	Clinical waste	N/A	96.55	N/A	30.24	N/A	126.79	N/A
		Nylon (polyamide) 6 polymer	2.58					23.58	N/A	2.77	N/A	26.35	N/A

Supplementary Table 12: Items and waste streams used for carpal tunnel decompression operation 1-10

Operation number C1=carpal tunnel decompression one etc. All waste of given item disposed of using consistent waste stream (A). Please see Supplementary Table 2 and Supplementary Table 3 for waste stream A (item dependent)

Item		Number of items used<br>Waste stream A used for product, followed by packaging where relevant									
		C1	C2	C3	C4	C5	C6	C7	C8	C9	C10
Minor op set		1	1	1	1	1	1	1	1	1	1
Reusable scrubs		5	5	5	6	7	5	8	5	5	6
Reusable tourniquet pressure cuff		1	1	1	1	0	0	0	1	1	1
Hand pack	Patient drape (fenestrated hand drape)	1	1	1	1	1	1	1	1	1	1
	Table drape (instruments)	1	1	1	1	1	1	1	1	1	1
	'Hand pack' packaging (drape component)	1	1	1	1	1	1	1	1	1	1
	Bowl	2	2	2	2	2	2	2	2	2	2
	Foam cube	2	2	2	2	2	2	2	2	2	2
	Gauze swab	1	1	1	1	1	1	1	1	1	1
	Kidney dish	1	1	1	1	1	1	1	1	1	1
	Light cover	1	1	1	1	1	1	1	1	1	1
	Needle counter	1	1	1	1	1	1	1	1	1	1
	Surgical blade	2	2	2	2	2	2	2	2	2	2
	Syringe (20 ml)	1	1	1	1	1	1	1	1	1	1
	'Hand pack' packaging (single-use equipment component)	1	1	1	1	1	1	1	1	1	1
Gloves (non-sterile, pair)		2	2	2	4	3	3	4	3	3	4
Sterile gloves (pair)		3	3	3	2	2	3	3	2	1	2
Sterile gloves (two pairs)		1	1	1	0	0	0	0	1	0	1
Sterile gloves (two pair, latex free)		0	0	0	1	1	0	0	0	1	0
Surgical face mask		6	6	6	6	1	1	6	3	3	5
Surgical face mask with eye protection		0	0	0	1	2	3	2	2	2	1
Surgical gown (including hand towels)		3	3	3	3	3	3	3	4	2	4
Surgical hat		5	5	5	6	7	5	8	5	5	6
Visor		0	0	0	0	0	0	0	0	1	0

Crepe bandage (7.5 cm wide)	1	1	1	1	1	1	1	1	1	1
Elasticated fabric dressing strip	3	3	3	2	2	2	2	2	0	2
Gauze (individual piece)	0	0	0	0	1	1	1	0	0	1
Gauze (sterile pack)	1	2	3	4	1	0	0	0	0	0
Incontinence pad	1	1	1	1	1	1	1	1	1	1
Nail scrubbing brush	3	1	0	1	3	1	0	2	2	0
Needle (blue)	0	0	0	0	0	1	1	0	1	1
Needle (green)	1	1	1	1	1	1	1	1	0	0
Needle (red)	1	1	1	1	1	0	0	0	0	0
Non-woven dressing	1	1	1	1	1	1	1	1	1	1
Skin marker	1	1	1	0	1	1	1	0	0	0
Stockinette tubular bandage	1	1	1	1	0	0	0	0	1	1
Surgical blade	1	1	0	0	1	0	0	0	0	0
Suture (monofilament, non-absorbable, 4-0)	1	0	0	1	0	0	0	1	0	1
Suture (monofilament, non-absorbable 5-0)	0	1	1	0	1	1	1	0	1	0
Syringe (10 ml)	1	1	1	1	0	0	0	1	0	1
Syringe (20 ml)	0	0	0	0	1	1	1	0	1	0
Tape (clear)	0	0	0	0	0	0	0	0	1	0
Undercast padding	1	1	1	1	1	1	1	1	1	1
Bupivacaine hydrochloride 0.5% (10 ml)	1	1	1	1	0	0	0	0	1	0
Chlorhexidine 2% from 500 ml container (100 ml)	1	1	1	1	1	1	1	1	1	1
Hyaluronidase 1500 I.U. (10 ml)	0	0	0	1	0	0	0	0	1	0
Levobupivacaine 5mg/10 ml (10 ml)	0	0	0	0	0	0	0	1	0	1
Lidocaine 1% (10 ml)	1	1	1	1	0	0	0	0	1	0
Lidocaine 1% with adrenaline 1:200,000 (20 ml)	0	0	0	0	1	1	1	0	0	0
Sodium Chloride 0.9% from 1 litre bottle (100 ml)	1	1	1	1	1	1	1	1	1	1
Clear bin bag (linen laundering)	1	1	1	1	1	1	1	1	1	1
Disinfectant sachet	1	1	1	1	1	1	1	1	1	1
Disinfectant wipe	8	7	5	5	4	5	3	12	5	6

Orange waste bag (infectious waste) with cable tie from theatre	1	1	1	1	1	1	1	1	1	1
Orange waste bag (infectious waste) with cable tie from scrub room	1	1	1	1	1	1	1	1	1	1
Mop head	1	1	1	1	1	1	1	1	1	1
Red bag (linen laundering) with cable tie	1	1	1	1	1	1	1	1	1	1

Supplementary Table 13: Items and waste streams used for inguinal hernia repair operation 1-6

Operation number H1=inguinal hernia operation one etc. All waste of given item disposed of using consistent waste stream (A). Please see Supplementary Table 4 and Supplementary Table 5 for waste stream A and B (item dependent)

Item	Number of items used<br>Waste stream A or B used for product, followed by packaging where relevant					
	H1	H2	H3	H4	H5	H6
General basic set (A)	1<br>A,A	1<br>A,A	0	0	0	0
General basic set (B)	0	0	1<br>A,A	1<br>A,A	1<br>A,A	1<br>A,A
Roberts artery forceps	1<br>A,A	0	0	0	0	0
Collingwood Stewart hernia forceps	0	0	0	1<br>A,A	0	0
High fluid drape	0	0	1<br>A,A	1<br>A,A	1<br>A,A	1<br>A,A
Huck towel	0	0	0	1<br>A,A	1<br>A,A	1<br>A,A
Low fluid drape	1<br>A,A	1<br>A,A	0	0	0	0
Reusable surgical gown (including hand towels)	1<br>B,B	1<br>B,B	0	2<br>A,A	0	2<br>A,A
Reusable surgical hat	0	0	2<br>A	0	0	0
Reusable scrubs	5<br>A	4<br>A	6<br>A	5<br>A	5<br>A	5<br>A
Diathermy pad lead	1<br>A	1<br>A	1<br>A	1<br>A	1<br>A	1<br>A
Shaver base	1<br>B	1<br>B	1<br>A	1<br>A	1<br>A	
Gloves (non-sterile, pair)	7<br>B	5<br>B	4<br>A	4<br>A	7<br>A	8<br>A
Sterile gloves (pair)	2<br>B,B	2<br>B,B	3<br>A,A	1<br>A,A	1<br>A,A	1<br>A,A
Sterile gloves (two pairs)	0	0	1<br>A,A	1<br>A,A	2<br>A,A	2<br>A,A
Sterile gloves (one pair, latex free)	1<br>B,B	1<br>B,B	0	2<br>A,A	0	0
Sterile under-gloves (pair)	1<br>B,B	1<br>B,B	0	0	0	0
Surgical face mask	1<br>B	1<br>B	3<br>A	5<br>A	2<br>A	2<br>A
Surgical face mask with eye protection	0	0	0	0	1<br>A	1<br>A

Surgical gown (including hand towels)	1<br>B,B	1<br>B,B	3<br>A,A	1<br>A,A	3<br>A,A	1<br>A,A
Surgical hat	5<br>B	4<br>B	4<br>A	5<br>A	6<br>A	3<br>A
Patient drape (incise drape, with iodine)	0	0	0	1<br>A,A	0	0
Table drape (instruments)	0	0	1<br>A,A	2<br>A,A	1<br>A,A	1<br>A,A
Absorbent towel pack	0	0	1<br>A,A	0	0	0
Diathermy pad	3<br>B,B	1<br>B,B	1<br>A,A	1<br>A,A	1<br>A,A	1<br>A,A
Diathermy tip	0	0	1<br>A,A	0	0	0
Gauze (sterile pack, 10x7.5 cm)	4<br>B,B	3<br>B,B	2<br>A,A	1<br>A,A	2<br>A,A	2<br>A,A
Gauze (sterile pack, 30x30 cm)	0	0	1<br>A,A	0	0	0
Gauze (sterile pack, 10x10 cm)	2<br>B,B	2<br>B,B	0	0	0	0
Incontinence pad	3<br>B	3<br>B	2<br>A	1<br>A	1<br>A	1<br>A
Kidney dish	1<br>B,B	1<br>B,B	0	0	0	0
Light handle	1<br>B,B	1<br>B,B	2<br>A,A	2<br>A,A	1<br>A,A	0
Mesh	1<br>B	1<br>B	1<br>A	1<br>A	1<br>A	1<br>A
Monopolar diathermy with smoke evacuation system	1<br>B,B	1<br>B,B	0	2<br>A,A	1<br>A,A	1<br>A,A
Nail scrubbing brush	2<br>B,B	1<br>B,B	1<br>A,A	0	1<br>A,A	0
Needle (green)	1<br>A,B	1<br>A,B	1<br>A,A	1<br>A,A	0	1<br>A,A
Needle counter	1<br>A,B	1<br>A,B	1<br>A,A	1<br>A,A	1<br>A,A	1<br>A,A
Nonwoven dressing (10x20 cm)	1<br>B,A	1<br>B,A	1<br>A,A	1<br>A,A	1<br>A,A	1<br>A,A
Nonwoven dressing (10x30 cm)	1<br>A,A	1<br>A,A	0	0	0	0
Pre-operative adhesive glove	3<br>B,B	3<br>B,B	1<br>A,A	1<br>A,A	1<br>A,A	0
Reinforced skin closure strip	1<br>A,A	1<br>A,A	0	0	0	0
Shaver head	1<br>A,B	1<br>A,B	1<br>A,A	1<br>A,A	1<br>A,A	0
Specimen pot (40 ml 4% formaldehyde)	1<br>A	1<br>A	0	0	0	0
Surgical blade (10)	1<br>A,B	1<br>A,B	1<br>A,A	1<br>A,A	1<br>A,A	1<br>A,A
Surgical suspensory bandage	1<br>A,A	1<br>A,A	0	0	0	0
Suture (braided, absorbable, 0)	1<br>A,A	0	0	0	0	0
Suture (braided, absorbable, 2-0)	1<br>A,B	1<br>A,B	4<br>A,A	2<br>A,A	1<br>A,A	1<br>A,A
Suture (monofilament, absorbable, 3-0)	1<br>A,B	1<br>A,B	1<br>A,A	1<br>A,A	1<br>A,A	1<br>A,A
Suture (monofilament, nonabsorbable, 1)	1	1	0	0	1	1

	A,B	A,B			A,A	A,A
Suture (monofilament, nonabsorbable, 2-0)	3<br>AB	3<br>A,B	2<br>A,A	0	0	0
Syringe (20 ml)	3<br>B,B	3<br>B,B	1<br>A,A	1<br>A,A	0	1<br>A,A
Chlorhexidine 1% from 500 ml container (100 ml)	0	0	1	1	1	1
Levobupivacaine 5mg/10 ml (10 ml)	6<br>A	6<br>A	0	0	0	3
Povidone iodine 10% from 500 ml container (60 ml)	1<br>A,A	1<br>A,A	0	0	0	0
Sodium Chloride 0.9% from 1 litre bottle (50 ml)	1	1	1	1	1	1
Topical skin adhesive (0.8 g)	0	0	0	1	0	0
Black waste bag (domestic waste) with cable tie	1<br>A	1<br>A	0	0	0	0
Chlorine tablet	2<br>A	2<br>A	0	0	0	0
Clear bin bag (for swab count)	1<br>A	1<br>A	0	0	0	0
Clear bin bag (recycling) with cable tie	0	0	1<br>A	1<br>A	1<br>A	1<br>A
Disinfectant sachet	0	0	1<br>A	1<br>A	1<br>A	1<br>A
Disinfectant wipe	8<br>B	5<br>B	7<br>A	6<br>A	7<br>A	7<br>A
Green bag (linen laundering) with cable tie	1<br>A	1<br>A	1<br>A	1<br>A	1<br>A	1<br>A
Mop head	1<br>B	1<br>B	1<br>A	1<br>A	1<br>A	1<br>A
Orange waste bag (infectious waste, large) with cable tie	0	0	1<br>A	1<br>A	1<br>A	1<br>A
Orange waste bag (infectious waste, small) with cable tie	0	0	1<br>A	1<br>A	1<br>A	1<br>A
Red bag (linen laundering) with cable tie	1<br>A	1<br>A	1<br>A	1<br>A	1<br>A	1<br>A
Yellow waste bag (clinical waste) with cable tie	1<br>A	1<br>A	0	0	0	0

Supplementary Table 14: Items and waste streams used for knee arthroplasty 1-10

Operation number K1=carpal tunnel decompression one etc. All waste of given item disposed of using consistent waste stream (A), aside from B for packaging of surgical gowns, orthopaedic hood for K2 and K3. Please see Supplementary Table 6 and Supplementary Table 7 for waste stream A and B (item dependent)

**Item**	**Number of items used**									
	K1	K2	K3	K4	K5	K6	K7	K8	K9	K10
Basic major orthopaedic set	1	1	1	1	1	1	1	1	1	1
Bipolar diathermy	0	0	0	0	0	0	1	0	0	0
Blunt Hohman bone elevator	0	0	0	0	0	0	0	1	1	0

Cruciate retaining femoral and tibial preparation and trialing set (size 1,2,7,8)		0	0	0	0	0	1	1	0	0	0
Cruciate retaining femoral and tibial trialing set (size 3-6)		0	1	1	1	1	1	1	1	1	1
Diathermy extras		0	0	0	0	0	1	0	0	0	0
Diathermy lead		0	0	0	0	0	0	1	0	0	0
Femoral and tibial preparation set (size 3-6)		1	1	1	1	1	1	1	1	1	1
Knee navigation set		0	0	0	0	0	0	0	1	0	0
Lanes tissue forceps		1	0	1	0	0	0	0	1	1	1
Light cover		1	1	1	1	1	1	1	1	1	1
Light handle		1	1	1	2	1	1	1	1	1	1
Miscellaneous knee system set		1	1	1	1	1	1	1	1	1	1
Non-toothed lamina spreader (large)		0	1	1	0	0	0	0	0	0	0
Non-toothed lamina spreader (small)		0	0	0	1	0	0	0	0	0	0
Orthopaedic surgical drill set		1	1	1	1	1	1	1	1	1	1
Patella preparation and trialing set		1	0	1	1	0	1	1	1	1	0
Posterior stabilised femoral and tibial trialing set (size 3-6)		1	0	0	0	1	0	0	1	0	0
Semb bone holding forceps		1	1	1	0	0	1	1	1	1	1
Reusable shaver base		0	0	0	0	1 A	0	1 A	0	0	0
Reusable scrubs		6	7	7	5	6	5	5	5	5	5
Knee pack	Surgical gown	1	1	1	1	1	1	1	1	1	1
	Knee pack packaging (gown component)	1	1	1	1	1	1	1	1	1	1
	Patient drape (240x150 cm)	1	1	1	1	1	1	1	1	1	1
	Patient drape (90 x75 cm)	1	1	1	1	1	1	1	1	1	1
	Patient drape (extremity, 230x325 cm)	1	1	1	1	1	1	1	1	1	1
	Patient drape (impervious, split, 152x177 cm)	1	1	1	1	1	1	1	1	1	1
	Patient drape (Mayo cover, 75x144 cm) x2	1	1	1	1	1	1	1	1	1	1
	Patient drape (pouch fluid collection, 40x35 cm)	1	1	1	1	1	1	1	1	1	1
	Patient drape (stockinette, impervious, 30x120 cm)	1	1	1	1	1	1	1	1	1	1
	Table drape (140x90 cm)	1	1	1	1	1	1	1	1	1	1
	Table drape (fan folded, 140x190 cm)	1	1	1	1	1	1	1	1	1	1

	Knee pack packaging (patient and instrument table drape component)	1	1	1	1	1	1	1	1	1	1
	Bowl (250 ml) x2	1	1	1	1	1	1	1	1	1	1
	Bowl (500 ml)	1	1	1	1	1	1	1	1	1	1
	Cast padding bandage x2	1	1	1	1	1	1	1	1	1	1
	Crepe bandage x2	1	1	1	1	1	1	1	1	1	1
	Diathermy bag	1	1	1	1	1	1	1	1	1	1
	Diathermy tip cleaner	1	1	1	1	1	1	1	1	1	1
	Kidney dish x3	1	1	1	1	1	1	1	1	1	1
	Light cover	1	1	1	1	1	1	1	1	1	1
	Monopolar diathermy	1	1	1	1	1	1	1	1	1	1
	Needle counter	1	1	1	1	1	1	1	1	1	1
	Skin marker pen	1	1	1	1	1	1	1	1	1	1
	Suction tubing	1	1	1	1	1	1	1	1	1	1
	Surgical blade (10) x3	1	1	1	1	1	1	1	1	1	1
	Swab gauze (10x7.5 cm) x5	1	1	1	1	1	1	1	1	1	1
	Swab gauze (30x30 cm) x10	1	1	1	1	1	1	1	1	1	1
	Towel dressing (2 in pack)	1	1	1	1	1	1	1	1	1	1
	Tray (small)	1	1	1	1	1	1	1	1	1	1
	Yankauer sucker	1	1	1	1	1	1	1	1	1	1
	Knee pack packaging (equipment component)	1	1	1	1	1	1	1	1	1	1
Gloves (non-sterile, pair)		7	7	8	7	7	5	6	10	7	7
Orthopaedic hood		3	2	2	2	3	0	0	2	3	3
Sterile gloves (pair)		0	5	5	7	6	6	8	5	5	7
Sterile gloves (two pairs)		8	2	2	3	1	1	2	2	3	3
Sterile gloves (two pair, latex free)		0	0	1	0	1	1	0	0	0	0
Surgical face mask		3	4	2	3	2	3	1	5	4	2
Surgical face mask with eye protection		1	1	3	1	1	3	2	0	0	0
Surgical gown (including hand towels)		3	2	2	3	3	2	2	3	4	3
Surgical hat		6	7	7	5	6	2	2	5	5	5

Sweat bands for hood x3	3	2	2	2	3	0	0	2	3	3
Patient drape (adhesive split sheet)	0	0	0	0	0	0	0	1	1	0
Patient drape (clear U drape)	0	1	1	1	1	0	0	0	0	1
Patient drape (incise drape)	0	0	0	0	0	1	1	0	0	0
Patient drape (incise drape, with iodine)	1	1	1	1	1	0	0	0	0	1
Table drape (instruments)	2	2	2	2	2	2	2	2	2	2
Adhesive operative towel	0	0	0	1	0	0	0	0	0	0
Batteries for knee navigation set (x3)	0	0	0	0	0	0	0	3	0	0
Border dressing (10x30 cm)	1	1	1	1	2	1	2	1	1	2
Border dressing (6x8 cm)	0	0	0	0	0	0	0	1	1	0
Catheter tip syringe	0	1	0	0	0	0	0	0	0	0
Cement mixing and delivery system	1	1	1	1	1	1	1	1	1	1
Cement mixing bowl	0	0	0	0	0	0	1	0	0	0
Crepe bandage (15 cm wide)	0	0	0	0	1	0	0	0	0	0
Cruciate retaining femoral implant	0	1	1	0	0	1	0		1	1
Diathermy bag	0	0	1	1	0	0	0	1	1	0
Diathermy tip	0	0	0	0	0	0	0	0	0	1
Diathermy tip cleaner	1	1	1	1	1	1	1	1	1	1
Elasticated fabric dressing strip	2	2	2	2	2	2	2	2	2	2
Gauze (individual piece)	1	1	1	1	0	0	0	0	0	0
Gauze (sterile pack)	0	0	0	0	0	0	0	0	0	0
High-vacuum wound drainage	0	0	0	0	0	1	1	0	0	0
Incontinence pad	2	3	1	1	1	1	1	1	1	1
Intravenous infusion giving set	1	1	1	1	1	1	1	0	0	1
Marker pen and ruler	0	0	0	0	0	0	0	1	0	0
Monopolar diathermy	0	0	0	0	0	0	0	0	1	0
Nail scrubbing brush	0	0	0	3	3	3	3	3	3	3
Needle (green)	1	1	1	1	0	0	0	0	0	0
Needle (red)	1	1	1	1	1	1	1	0	0	1
Needle (white)	2	2	2	2	2	1	1	2	2	2

Posterior stabilised femoral implant	1	0	0	1	1	0	0	1	0	0
Pre-operative adhesive glove	0	0	0	0	1	0	1	0	0	0
Primary tibial baseplate implant	1	1	1	1	1	1	1	1	1	1
Pulsed lavage system	1	1	1	1	1	1	1	1	1	1
Saw blade	1	1	1	2	1	1	1	1	1	1
Self-adherent bandage	1	0	0	0	0	0	0	0	0	0
Shaver head	0	0	0	0	1	0	1	0	0	0
Skin stapler	0	0	0	1	1	0	0	1	1	1
Sticky label	1	1	1	1	1	0	0	0	0	0
Suction receptacle	1	1	1	1	1	1	1	1	1	1
Suction tip	0	0	0	0	0	0	1	0	0	0
Suction tubing	0	0	1	0	0	1	0	0	0	0
Surgical blade (10)	0	1	1	0	0	0	1	0	0	0
Suture (braided, absorbable, 2-0)	1	1	1	1	0	0	0	1	1	1
Suture (monofilament, absorbable, 3-0, 643)	0	0	0	0	0	1	1	0	0	0
Suture (monofilament, absorbable 3-0, 696)	2	2	2	0	0	0	0	0	0	0
Suture (braided, absorbable 1-0, 803)	1	1	1	3	0	3	3	2	2	3
Suture (braided, absorbable 1-0, 932)	0	0	0	0	3	0	0	0	0	0
Swab tray (large)	1	1	1	1	2	2	1	1	1	1
Swab tray (small)	1	1	1	1	1	1	1	1	1	1
Symmetric patella implant	1	0	1	1	0	1	1	1	1	0
Syringe (20 ml)	1	1	1	1	1	1	1	0	0	1
Syringe (50 ml)	2	2	2	1	1	2	2	4	4	2
Tibial bearing insert cruciate retaining implant	1	1	1	1	0	0	1		1	1
Tibial bearing insert posterior stabilised implant	0	0	0	0	1	0	2	1	0	0
Tourniquet pressure cuff (leg)	1	1	1	1	1	1	1	1	1	1
Transparent film adhesive dressing	1	0	0	0	0	0	1	0	0	0
Tubular support bandage	0	0	0	0	1	1	1	1	1	1
Wound closure strips	2	2	2	0	0	0	0	0	2	0

Adrenaline 1mg in 1 ml (1 ml)	1	1	1	1	1	1	1	0	0	1
Bone cement mix (with gentamicin)	0	0	0	1	0	1	2	0	0	0
Bone cement mix (with tobramycin)	2	2	2	0	2	0	0	2	2	2
Chlorhexidine 2% from 500 ml bottle (150 ml)	1	1	1	1	1	0	0	0	0	1
Chlorhexidine gluconate in 70% denatured ethanol with 4 ml red stain solution from 200 ml bottle (150 ml)	1	0	0	1	1	0	0	0	0	1
Iodinated povidone 10% w/w alcoholic tincture from 500 ml bottle (50 ml)	0	0	0	1	0	1	1	1	1	0
Ketorolac tromethamine 30mg in 1 ml (1 ml)	1	1	1	1	1	1	1	0	0	1
Levobupivacaine 2.5mg/ml (10 ml)	0	0	0	0	0	0	0	4	4	0
Ropivacaine 75mg in 10 ml (10 ml)	2	2	2	2	2	2	2	0	0	2
Sodium chloride 0.9% (100 ml)	1	2	1	1	1	1	1	1	1	1
Sodium chloride 0.9% for irrigation (3 L bag)	1	1	1	1	1	1	1	1	1	1
Topical skin adhesive (0.8 g)	1	0	0	0	0	0	0	0	0	0
Anatomical waste bin	1	1	1	1	1	1	1	1	1	1
Clear bin bag (linen laundering)	1	1	1	1	1	1	1	1	1	1
Clear waste bag (recycling) with cable tie	1	1	1	1	1	1	1	1	1	1
Disinfectant sachet	1	1	1	1	1	1	1	1	1	1
Disinfectant wipe	50	45	50	16	62	22	25	20	25	30
Orange waste bag (infectious waste) with cable tie from theatre	2	2	2	2	2	2	2	2	2	2
Orange waste bag (infectious waste) with cable tie from scrub room	1	0	0	1	1	1	1	1	1	1
Mop head	1	1	1	1	1	1	1	1	1	1
Red bag (linen laundering) with cable tie	1	1	1	1	1	1	1	1	1	1
Yellow/ black waste bag (non-infectious offensive waste) with cable tie from scrub room	0	1	1	0	0	0	0	0	0	0

Supplementary Table 15: Items and waste streams used for laparoscopic cholecystectomy operation 1-6

Operation number L1=laparoscopic cholecystectomy operation one etc. Please see Supplementary Table 8 and Supplementary Table 9 for waste stream A and B (item dependent)

**Item**	**Number of items used or volume where specified<br>Waste stream A or B used for product, followed by packaging where relevant**					
	L1	L2	L3	L4	L5	L6
General basic set	1<br>A,A	1<br>A,A	1<br>A,A	1<br>A,A	1<br>A,A	1<br>A,A
General laparoscopic set	1<br>A,A	1<br>A,A	1<br>A,A	1<br>A,A	1<br>A,A	1<br>A,A
Laparoscope set (10 mm, 0 degree)	1<br>A,A	1<br>A,A	1<br>A,A	1<br>A,A	1<br>A,A	1<br>A,A
Diathermy lead	0	0	1<br>A,A	0	0	0
Laparoscopic grasping forceps	0	0	0	1<br>A,A	0	0
Quiver and clip	1<br>A,A	1<br>A,A	1<br>A,A	1<br>A,A	1<br>A,A	1<br>A,A
High fluid drape	1<br>A,N	1<br>A,A	1<br>A,A	1<br>A,A	1<br>A,A	1<br>A,A
Huck towel	0	1<br>A,A	0	1<br>A,B	1<br>A,B	1<br>A,B
Reusable surgical gown (including hand towels)	1<br>A,A	1<br>A,A	3<br>A,A	2<br>A,B	0	1<br>A,B
Reusable surgical gown (including hand towels) double pack	0	1<br>A,A	0	1<br>A,A	1<br>A,A	1<br>A,A
Reusable surgical hat	0	0	0	1<br>A	1<br>A	1<br>A
Scrubs	7<br>A	7<br>A	6<br>A	6<br>A	6<br>A	6<br>A
Diathermy pad lead	1<br>A	1<br>A	1<br>A	1<br>A	1<br>A	1<br>A
Gloves (non-sterile, pair)	8<br>A	7<br>A	6<br>A	5<br>B	6<br>B	3<br>B
Sterile gloves (pair)	0	1<br>A,A	3<br>A,A	2<br>B,A	2<br>B,A	2<br>B,A
Sterile gloves (two pairs)	3<br>A,A	3<br>A,A	0	1<br>B,A	1<br>B,A	2<br>B,A
Sterile gloves (one pair, latex free)	0	0	1<br>A,A	0	0	0
Surgical face mask	0	2<br>A	3<br>A	3<br>B	2<br>B	4<br>B
Surgical face mask with eye protection	1<br>A	1<br>A	0	0	1<br>B	0
Surgical gown (including hand towels)	2<br>A,A	0	0	0	1<br>B,A	1<br>B,A
Surgical hat	7<br>A	7<br>A	6<br>A	5<br>B	5<br>B	5<br>B
Table drape (instrument)	1<br>A,A	1<br>A,A	1<br>A,A	1<br>B,B	1<br>B,B	1<br>B,B
Absorbent towel pack	1<br>A,A	0	0	0	0	0
Anti-fog endoscopic demister	0	1<br>A,A	1<br>A,A	1<br>B,B	1<br>B,B	1<br>B,B

Diathermy pad	1<br>A,A	1<br>A,A	1<br>A,A	1<br>B,B	1<br>B,B	1<br>B,B
Endoscopic clip applier	1<br>A,A	1<br>A,A	1<br>A,A	1<br>A,B	1<br>A,B	1<br>A,B
Gauze (sterile pack)	2<br>A,A	1<br>A,A	1<br>A,A	2<br>B,B	2<br>B,B	2<br>B,B
Incontinence pad	1<br>A	1<br>A	1<br>A	1<br>B	2<br>B	1<br>B
Insufflating tubing	1<br>A,A	1<br>A,A	1<br>A,A	1<br>B,B	1<br>B,B	1<br>B,B
Laparoscope cover	1<br>A,A	1<br>A,A	1<br>A,A	1<br>B,B	1<br>B,B	1<br>B,B
Laparoscopic scissors	1<br>A,A	1<br>A,A	1<br>A,A	1<br>A,B	0	1<br>A,B
Laparoscopic tissue retrieval system	1<br>A,A	1<br>A,A	1<br>A,A	1<br>B,B	1<br>B,B	1<br>B,B
Light handle	1<br>A,A	0	0	0	0	0
Nail scrubbing brush	0	1<br>A,A	1<br>A,A	1<br>A,A	1<br>A,A	1<br>A,A
Needle (green)	1<br>A,A	1<br>A,A	1<br>A,A	1<br>A,B	1<br>A,B	1<br>A,B
Needle counter	1<br>A,A	1<br>A,A	1<br>A,A	1<br>A,B	1<br>A,B	1<br>A,B
Nonwoven dressing (6x7 cm)	4<br>A,A	4<br>A,A	4<br>A,A	4<br>B,B	3<br>B,B	4<br>B,B
Port (12 mm)	1<br>A,A	2<br>A,A	1<br>A,A	2<br>A,B	2<br>A,B	2<br>A,B
Port (5 mm, dual pack)	1<br>A,A	1<br>A,A	1<br>A,A	1<br>A,B	1<br>A,B	1<br>A,B
Pressure saline infusion bag	0	0	1<br>A,A	0	0	0
Reinforced skin closure strip	0	1<br>A,A	1<br>A,A	0	1<br>A,B	0
Specimen pot (40 ml 4% formaldehyde)	0	1<br>A,A	1<br>A,A	0	0	0
Specimen pot (not pre-filled)	1<br>A	0	0	1<br>B	1<br>B	1<br>B
Suction irrigation	0	0	1<br>A,A	1<br>B,B	0	1<br>B,B
Suction receptacle	0	0	1<br>A	1<br>B	0	0
Surgical blade (11)	1<br>A,A	0	0	0	0	0
Surgical blade (15)	0	1<br>A,A	0	1<br>A,B	1<br>A,B	1<br>A,B
Suture (braided, absorbable, 3-0)	0	1<br>A,A	1<br>A,A	2<br>A,B	2<br>A,B	1<br>A,B
Suture (monofilament, absorbable, 3-0)	1<br>A,A	0	0	0	0	0
Suture (monofilament, absorbable, 1)	1<br>A,A	2<br>A,A	2<br>A,A	1<br>A,B	1<br>A,B	1<br>A,A
Syringe (10 ml)	1<br>A,A	1<br>A,A	1<br>A,A	1<br>B,B	1<br>B,B	1<br>B,B
Syringe (20 ml)	2<br>A,A	1<br>A,A	1<br>A,A	2<br>B,B	1<br>B,B	1<br>B,B
Tonsil swab pack	0	1<br>A,A	0	1<br>B,B	1<br>B,B	1<br>B,B

Carbon dioxide (from 3kg cylinder containing 450 L)	28.5 litres	51.3 litres	102.7 litres	144 litres	74.5 litres	22.3 litres
Chlorhexidine 1% from 500 ml container (50 ml)	1	1	1	1	1	1
Levobupivacaine 2.5mg/10 ml (10 ml)	4<br>A	3<br>A	3<br>A	3<br>B	3<br>B	3<br>B
Sodium Chloride 0.9% for irrigation (1 L bag)	0	0	1<br>A	2<br>B	0	0
Sodium Chloride 0.9% from 1 litre bottle (50 ml)	1	1	1	1	1	1
Black waste bag (domestic waste) with cable tie	0	0	0	2	2	2
Clear bin bag (quiver diathermy)	1<br>A	1<br>A	1<br>A	1<br>A	1<br>A	1<br>A
Clear bin bag (recycling) with cable tie	1<br>A	1<br>A	1<br>A	0	0	0
Disinfectant sachet	1<br>A	1<br>A	1<br>A	1<br>B	1<br>B	1<br>B
Disinfectant wipe	8<br>A	7<br>A	5<br>A	4<br>B	15<br>B	6<br>B
Green bag (linen laundering) with cable tie	1<br>A	1<br>A	1<br>A	1<br>A	1<br>A	1<br>A
Mop head	1<br>A	1<br>A	1<br>A	1<br>B	1<br>B	1<br>B
Orange waste bag (infectious waste, large) with cable tie	1<br>A	1<br>A	1<br>A	0	0	0
Orange waste bag (infectious waste, small) with cable tie	1<br>A	1<br>A	1<br>A	0	0	0
Red bag (linen laundering) with cable tie	1<br>A	1<br>A	1<br>A	1<br>A	1<br>A	1<br>A
Yellow/ black waste bag (non-infectious offensive waste, large) with cable tie	0	0	0	1<br>A	1<br>A	1<br>A
Yellow/ black waste bag (non-infectious offensive waste, small) with cable tie	0	0	0	1<br>A	1<br>A	1<br>A

Supplementary Table 16: Items and waste streams used for tonsillectomy operation 1-10
Operation number T1=tonsillectomy one etc. Please see Supplementary Table 10 and Supplementary Table 11 for waste stream A and B (item dependent)

**Item**	**Number of items used**<br>**Waste stream A or B used for product, followed by packaging where relevant**									
	T1	T2	T3	T4	T5	T6	T7	T8	T9	T10
Tonsillectomy set<br>(set A for T1, T4-T10; set B for T2, T3)	1<br>A,A	1<br>A,A	1<br>A,A	1<br>A,A	1<br>A,A	1<br>A,A	1<br>A,A	1<br>A,A	1<br>A,A	1<br>A,A
Reusable ENT split head drape (two 42"x42")	0	1<br>A,B	1<br>A,A	0	0	0	0	0	0	0
Reusable surgical gown (including hand towels)	2<br>A,A	2<br>A,A	2<br>A,A	2<br>A,A	2<br>A,A	2<br>A,A	2<br>A,A	2<br>A,A	2<br>A,A	2<br>A,A
Reusable surgical hat	3<br>A	0	0	2<br>A	2<br>A	3<br>A	2<br>A	3<br>A	2<br>A	3<br>A
Reusable scrubs	4<br>A	4<br>A	4<br>A	4<br>A	4<br>A	6<br>A	5<br>A	7<br>A	6<br>A	6<br>A
Gloves (non-sterile, one pair)	1<br>A	2<br>B	2<br>B	2<br>A	2<br>A	1<br>A	1<br>A	1<br>A	3<br>A	3<br>A
Sterile gloves (one pair)	3<br>A,A	3<br>B,A	3<br>B,A	2<br>A,A	2<br>A,A	2<br>A,A	2<br>A,A	2<br>A,A	2<br>A,A	2<br>A,A
Surgical face mask	2<br>A	0	2<br>B	0	0	1<br>A	1<br>A	1<br>A	1<br>A	0
Surgical face mask with eye protection	0	2<br>B	0	0	0	1<br>A	1<br>A	1<br>A	0	0
Surgical hat	1<br>A	4<br>B	4<br>B	2<br>A	2<br>A	3<br>A	3<br>A	4<br>A	4<br>A	3<br>A
Patient drape (fenestrated ENT drape)	1<br>A,A	0	0	1<br>A,A	1<br>A,A	1<br>A,A	1<br>A,A	1<br>A,A	1<br>A,A	1<br>A,A
Table drape (instruments)	1<br>A,A	1<br>B,B	1<br>B,B	1<br>A,A	1<br>A,A	1<br>A,A	1<br>A,A	1<br>A,A	1<br>A,A	1<br>A,A
Coblation™ wand	0	0	0	0	0	1<br>A,A	1<br>A,A	1<br>A,A	1<br>A,A	1<br>A,A
Kidney dish	1<br>A,A	0	0	0	0	0	0	0	0	0
Filter needle	1<br>A,A	0	0	0	0	0	0	0	0	0
Gallipot	1<br>A,A	0	0	0	0	2<br>A,A	2<br>A,A	2<br>A,A	2<br>A,A	2<br>A,A
Incontinence pad	0	0	1<br>A	0	0	0	0	0	0	0
Braided silk tonsil ties	1<br>A,A	2<br>B,A	1<br>B,A	0	0	0	0	0	0	0
Gauze (non-sterile)	2<br>A	0	0	0	0	0	0	0	0	0
Nail scrubbing brush	1<br>A,A	1<br>B,A	0	0	0	1<br>A,A	0	0	0	0
Suction tip	0	1<br>BB	0	1<br>AA	1<br>AA	1<br>AA	1<br>AA	1<br>AA	1<br>AA	1<br>AA
Suction receptacle	1<br>A	1<br>B	1<br>B	1<br>A	1<br>A	1<br>A	1<br>A	1<br>A	1<br>A	1<br>A
Suction tubing	1<br>A	1<br>B	1<br>B	1<br>A	1<br>A	1<br>A	1<br>A	1<br>A	1<br>A	1<br>A
Syringe (10 ml)	1<br>A,A	0	0	0	0	0	0	0	0	0

Tonsil swab pack	1<br>A,A	3<br>B,B	4<br>B,A	2<br>A,A	1<br>A,A	2<br>A,A	2<br>A,A	2<br>A,A	1<br>A,A	1<br>A,A
Yankauer sucker	1<br>A,A	1<br>B,B	1<br>B,B	1<br>A,A	1<br>A,A	1<br>A,A	1<br>A,A	1<br>A,A	1<br>A,A	1<br>A,A
Specimen pot (40 ml 4% formaldehyde)	0	1<br>A	0	0	0	0	0	0	0	0
Bupivacaine 0.5% with 1:200,000 adrenaline (10 ml)	1<br>A,A	1<br>A,A	1<br>A,A	0	0	0	0	0	0	0
Chirocaine 2.5mg/ml (10 ml)	0	0	0	0	0	1<br>A,A	1<br>A,A	1<br>A,A	1<br>A,A	1<br>A,A
Sodium chloride 0.9% from 1 litre bottle (150 ml)	1	1	1	1	1	1	1	1	1	1
Sodium chloride 0.9%, intravenous infusion bag (500 ml)	0	0	0	0	0	1<br>A,A	1<br>A,A	1<br>A,A	1<br>A,A	1<br>A,A
Yellow soft paraffin BP 100% from 15g tube	0	1	1	1	1	1	1	1	1	1
Black waste bag (domestic waste) with cable tie	1<br>A	0	0	1<br>A	1<br>A	1<br>A	1<br>A	1<br>A	1<br>A	1<br>A
Clear waste bag (for swab count)	1<br>A	1<br>B	1<br>B	1<br>A	1<br>A	1<br>A	1<br>A	1<br>A	1<br>A	1<br>A
Clear waste bag (recycling) with cable tie	1<br>A	2<br>A	2<br>A	1<br>A	1<br>A	1<br>A	1<br>A	1<br>A	1<br>A	1<br>A
Disinfectant sachet	1<br>A	1<br>A	1<br>A	1<br>A	1<br>A	1<br>A	1<br>A	1<br>A	1<br>A	1<br>A
Disinfectant wipe	5<br>A	6<br>B	6<br>B	3<br>A	3<br>A	6<br>A	4<br>A	4<br>A	8<br>A	5<br>A
Green bag (linen laundering) with cable tie	1<br>A	1<br>A	1<br>A	1<br>A	1<br>A	1<br>A	1<br>A	1<br>A	1<br>A	1<br>A
Mop head	1<br>A	1<br>B	1<br>B	1<br>A	1<br>A	1<br>A	1<br>A	1<br>A	1<br>A	1<br>A
Orange waste bag (infectious waste) with cable tie	0	1<br>A	1<br>A	0	0	0	0	0	0	0
Yellow waste bag (clinical waste) with cable tie	1<br>A	0	0	1<br>A	1<br>A	1<br>A	1<br>A	1<br>A	1<br>A	1<br>A

Supplementary Table 17: Carbon footprint of carpal tunnel decompression operations impact assessment
C1= carpal tunnel decompression operation one etc. CO_2e=carbon dioxide equivalents

**Item**	**Carbon footprint (g CO_2e)**										
	**C1**	**C2**	**C3**	**C4**	**C5**	**C6**	**C7**	**C8**	**C9**	**C10**	**Mean average**
Minor op set	1922.18	1922.18	1922.18	1922.18	1922.18	1922.18	1922.18	1922.18	1922.18	1922.18	**1922.18**
Carbon footprint reusable minor op set (g CO_2e)	1922.18	1922.18	1922.18	1922.18	1922.18	1922.18	1922.18	1922.18	1922.18	1922.18	**1922.18**
Reusable scrubs	232.16	232.16	232.16	278.59	325.02	232.16	371.45	232.16	232.16	278.59	**264.66**
Carbon footprint reusable personal protective equipment (g CO_2e)	232.16	232.16	232.16	278.59	325.02	232.16	371.45	232.16	232.16	278.59	**264.66**
Reusable tourniquet pressure cuff	79.45	79.45	79.45	79.45	0	0	0	79.45	79.45	79.45	**55.61**
Carbon footprint reusable non-set equipment (g CO_2e)	79.45	79.45	79.45	79.45	0	0	0	79.45	79.45	79.45	**55.61**
Gloves (non-sterile, pair)	44.11	44.11	44.11	88.22	66.16	66.16	88.22	66.16	66.16	88.22	**66.16**
Sterile gloves (pair)	372.44	372.44	372.44	248.29	248.29	372.44	372.44	248.29	124.15	248.29	**297.95**
Sterile gloves (two pairs)	185.44	185.44	185.44	0	0	0	0	185.44	0	185.44	**92.72**
Sterile gloves (two pair, latex free)	0	0	0	180.69	180.69	0	0	0	180.69	0	**54.21**
Surgical face mask	104.62	104.62	104.62	104.62	17.44	17.44	104.62	52.31	52.31	87.19	**74.98**
Surgical face mask with eye protection	0	0	0	37.72	75.44	113.16	75.44	75.44	75.44	37.72	**49.04**
Surgical gown (including hand towels)	1996.07	1996.07	1996.07	1996.07	1996.07	1996.07	1996.07	2661.43	1330.71	2661.43	**2062.60**
Surgical hat	17.70	17.70	17.70	21.24	24.78	17.70	28.32	17.70	17.70	21.24	**20.17**
Visor	0	0	0	0	0	0	0	0	43.25	0	**4.32**
Carbon footprint single-use personal protective equipment (g CO_2e)	2,720.38	2,720.38	2,720.38	2,676.85	2,608.87	2,582.96	2,665.10	3,306.77	1,890.41	3,329.52	**2,722.16**

Hand pack	Patient drape (fenestrated hand drape)	2,739.93	2,739.93	2,739.93	2,739.93	2,739.93	2,739.93	2,739.93	2,739.93	2,739.93	2,739.93	**2,739.93**
	Table drape (instruments)	1,049.85	1,049.85	1,049.85	1,049.85	1,049.85	1,049.85	1,049.85	1,049.85	1,049.85	1,049.85	**1,049.85**
	Hand pack packaging (drape component)	91.28	91.28	91.28	91.28	91.28	91.28	91.28	91.28	91.28	91.28	**91.28**
Carbon footprint single-use patient and instrument table drapes (g CO_2e)		3,881.05	3,881.05	3,881.05	3,881.05	3,881.05	3,881.05	3,881.05	3,881.05	3,881.05	3,881.05	**3,881.05**
Hand pack	Bowl	127.24	127.24	127.24	127.24	127.24	127.24	127.24	127.24	127.24	127.24	**127.24**
	Foam cube	18.50	18.50	18.50	18.50	18.50	18.50	18.50	18.50	18.50	18.50	**18.50**
	Gauze swab	188.36	188.36	188.36	188.36	188.36	188.36	188.36	188.36	188.36	188.36	**188.36**
	Kidney dish	147.07	147.07	147.07	147.07	147.07	147.07	147.07	147.07	147.07	147.07	**147.07**
	Light cover	15.15	15.15	15.15	15.15	15.15	15.15	15.15	15.15	15.15	15.15	**15.15**
	Needle counter	236.83	236.83	236.83	236.83	236.83	236.83	236.83	236.83	236.83	236.83	**236.83**
	Surgical blade	14.52	14.52	14.52	14.52	14.52	14.52	14.52	14.52	14.52	14.52	**14.52**
	Syringe (20 ml)	26.64	26.64	26.64	26.64	26.64	26.64	26.64	26.64	26.64	26.64	**26.64**
	Hand packaging (single-use equipment component)	14.03	14.03	14.03	14.03	14.03	14.03	14.03	14.03	14.03	14.03	**14.03**
Crepe bandage (7.5 cm wide)		308.90	308.90	308.90	308.90	308.90	308.90	308.90	308.90	308.90	308.90	**308.90**
Elasticated fabric dressing strip		6.67	6.67	6.67	4.45	4.45	4.45	4.45	4.45	0	4.45	**4.67**
Gauze (individual piece)		0	0	0	0	37.19	37.19	37.19	0	0	37.19	**14.88**
Gauze (sterile pack)		203.26	406.53	609.79	813.06	203.26	0	0	0	0	0	**223.59**
Incontinence pad		95.65	95.65	95.65	95.65	95.65	95.65	95.65	95.65	95.65	95.65	**95.65**
Nail scrubbing brush		134.85	44.95	0	44.95	134.85	44.95	0	89.90	89.90	0	**58.44**
Needle (blue)		0	0	0	0	0	3.18	3.18	0	3.18	3.18	**1.27**

Needle (green)	3.63	3.63	3.63	3.63	3.63	3.63	3.63	3.63	0	0	**2.90**
Needle (red)	5.62	5.62	5.62	5.62	5.62	0	0	0	0	0	**2.81**
Non-woven dressing	10.17	10.17	10.17	10.17	10.17	10.17	10.17	10.17	10.17	10.17	**10.17**
Skin marker	54.56	54.56	54.56	0	54.56	54.56	54.56	0	0	0	**32.74**
Stockinette tubular bandage	83.34	83.34	83.34	83.34	0	0	0	0	83.34	83.34	**50**
Surgical blade	7.00	7.00	0	0	7.00	0	0	0	0	0	**2.10**
Suture (monofilament, non-absorbable, 4-0)	7.43	0	0	7.43	0	0	0	7.43	0	7.43	**2.97**
Suture (monofilament, non-absorbable 5-0)	0	7.43	7.43	0	7.43	7.43	7.43	0	7.43	0	**4.46**
Syringe (10 ml)	27.67	27.67	27.67	27.67	0	0	0	27.67	0	27.67	**16.60**
Syringe (20 ml)	0	0	0	0	65.05	65.05	65.05	0	65.05	0	**26.02**
Tape (clear)	0	0	0	0	0	0	0	0	4.95	0	**0.50**
Undercast padding	52.02	52.02	52.02	52.02	52.02	52.02	52.02	52.02	52.02	52.02	**52.02**
Carbon footprint single-use equipment and medical devices (g CO_2e)	1,789.11	1,902.48	2,053.79	2,245.23	1,778.12	1,475.52	1,430.57	1,388.16	1,508.94	1,418.34	**1,699.03**
Bupivacaine hydrochloride 0.5% (10 ml)	249.94	249.94	249.94	249.94	0	0	0	0	249.94	0	**124.97**
Chlorhexidine 2% from 500 ml container (100 ml)	107.30	107.30	107.30	107.30	107.30	107.30	107.30	107.30	107.30	107.30	**107.30**
Hyaluronidase 1500 I.U. (10 ml)	0	0	0	387.85	0	0	0	0	387.85	0	**77.57**
Levobupivacaine 5mg/10 ml (10 ml)	0	0	0	0	0	0	0	239.30	0	239.30	**47.86**
Lidocaine 1% (10 ml)	301.68	301.68	301.68	301.68	0	0	0	0	301.68	0	**150.84**
Lidocaine 1% with adrenaline 1:200,000 (20 ml)	0	0	0	0	702.05	702.05	702.05	0	0	0	**210.62**
Sodium Chloride 0.9% from 1 litre bottle (100 ml)	75.87	75.87	75.87	75.87	75.87	75.87	75.87	75.87	75.87	75.87	**75.87**

Carbon footprint pharmaceuticals (g CO_2e)	734.79	734.79	734.79	1,122.64	885.22	885.22	885.22	422.47	1,122.64	422.47	**795.03**
Clear bin bag (linen laundering)	12.52	12.52	12.52	12.52	12.52	12.52	12.52	12.52	12.52	12.52	**12.52**
Disinfectant sachet	27.49	27.49	27.49	27.49	27.49	27.49	27.49	27.49	27.49	27.49	**27.49**
Disinfectant wipe	112.78	98.68	70.49	70.49	56.39	70.49	42.29	169.17	70.49	84.58	**84.58**
Orange waste bag (infectious waste) with cable tie from theatre	307.44	307.44	307.44	307.44	307.44	307.44	307.44	307.44	307.44	307.44	**307.44**
Orange waste bag (infectious waste) with cable tie from scrub room	61.49	61.49	61.49	61.49	61.49	61.49	61.49	61.49	61.49	61.49	**61.49**
Mop head	151.71	151.71	151.71	151.71	151.71	151.71	151.71	151.71	151.71	151.71	**151.71**
Red bag (linen laundering) with cable tie	25.85	25.85	25.85	25.85	25.85	25.85	25.85	25.85	25.85	25.85	**25.85**
Carbon footprint cleaning products, waste (g CO_2e)	699.27	685.17	656.98	656.98	642.88	656.98	628.78	755.66	656.98	671.07	**671.07**
**Total Carbon footprint (g CO_2e)**	**12,058.38**	**12,157.65**	**12,280.77**	**12,862.96**	**12,043.34**	**11,636.07**	**11,784.36**	**11,987.90**	**11,293.81**	**12,002.67**	**12,010.79**

Supplementary Table 18: Carbon footprint of inguinal hernia repair operations impact assessment

H1= inguinal hernia operation one etc. CO_2e=carbon dioxide equivalents

Item	Carbon footprint (g CO2e)						
	**H1**	**H2**	**H3**	**H4**	**H5**	**H6**	**Mean average**
General basic set (set A for H1, H2; set B for H3-6)	3,017.22	3,017.22	2,395.86	2,395.86	2,395.86	2,395.86	**2,602.98**
Roberts artery forceps	197.07	0	0	0	0	0	**32.84**
Collingwood Stewart hernia forceps	0	0	0	197.10	0	0	**32.85**
Carbon footprint reusable sets and individually wrapped instruments (g CO_2e)	3,214.29	3,017.22	2,395.86	2,592.96	2,395.86	2,395.86	**2,668.68**
High fluid drape	0.00	0.00	1098.42	1098.42	1098.42	1098.42	**732.28**
Huck towel	0.00	0.00	0.00	123.51	123.51	123.51	**61.76**
Low fluid drape	744.54	744.54	0.00	0.00	0.00	0.00	**248.18**
Carbon footprint reusable patient drapes (g CO_2e)	744.54	744.54	1,098.42	1,221.93	1,221.93	1,221.93	**1,042.21**
Reusable surgical gown (including hand towels)	300.69	300.69	0.00	505.34	0.00	505.34	**268.68**
Reusable surgical hat	0.00	0.00	7.33	0.00	0.00	0.00	**1.22**
Reusable scrubs	290.19	232.16	348.23	290.19	290.19	290.19	**290.19**
Carbon footprint reusable personal protective equipment (g CO_2e)	590.88	532.84	355.56	795.54	290.19	795.54	**560.09**
Diathermy pad lead	9.24	9.24	9.24	9.24	9.24	9.24	**9.24**
Shaver base	0.47	0.47	0.44	0.44	0.44	0.00	**0.38**
Carbon footprint non-set reusable equipment (g CO_2e)	9.71	9.71	9.68	9.68	9.68	9.24	**9.62**
Gloves (non-sterile, pair)	177.17	126.55	88.22	88.22	154.38	176.44	**135.16**
Sterile gloves (pair)	287.87	287.87	358.26	119.42	119.42	119.42	**215.38**

Sterile gloves (two pairs)	0.00	0.00	178.60	178.60	357.19	357.19	**178.60**
Sterile gloves (one pair, latex free)	125.61	125.61	0.00	204.95	0.00	0.00	**76.03**
Sterile under-gloves (pair)	108.14	108.14	0.00	0.00	0.00	0.00	**36.05**
Surgical face mask	19.64	19.64	52.31	87.19	34.87	34.87	**41.42**
Surgical face mask with eye protection	0.00	0.00	0.00	0.00	37.72	37.72	**12.57**
Surgical gown (including hand towels)	774.01	774.01	1980.58	660.19	1980.58	660.19	**1138.26**
Surgical hat	19.93	15.94	14.16	17.70	21.24	10.62	**16.60**
Carbon footprint single-use patient drape and instrument table drape (g CO_2e)	1,512.37	1,457.76	2,672.12	1,356.25	2,705.40	1,396.45	**1,850.06**
Patient drape (incise drape, with iodine)	0.00	0.00	0.00	116.09	0.00	0.00	**19.35**
Table drape (instruments)	0.00	0.00	775.39	1550.78	775.39	775.39	**646.16**
Carbon footprint single-use personal protective equipment (g CO_2e)	0	0	775.39	1,666.87	775.39	775.39	**665.51**
Absorbent towel pack	0.00	0.00	75.77	0.00	0.00	0.00	**12.63**
Diathermy pad	228.43	76.14	69.67	69.67	69.67	69.67	**97.21**
Diathermy tip	0.00	0.00	17.08	0.00	0.00	0.00	**2.85**
Gauze (sterile pack, 10x7.5 cm)	666.16	499.62	318.22	159.11	318.22	318.22	**379.92**
Gauze (sterile pack, 30x30 cm)	0.00	0.00	713.28	0.00	0.00	0.00	**118.88**
Gauze (sterile pack, 10x10 cm)	426.93	426.93	0.00	0.00	0.00	0.00	**142.31**
Incontinence pad	272.90	272.90	151.46	75.73	75.73	75.73	**154.08**
Kidney dish	73.04	73.04	0.00	0.00	0.00	0.00	**24.35**
Light handle	30.14	30.14	59.67	59.67	29.84	0.00	**34.91**

Mesh	279.26	279.26	229.30	229.30	229.30	229.30	**245.95**
Monopolar diathermy with smoke evacuation system	741.95	741.95	0.00	1282.93	641.46	641.46	**674.96**
Nail scrubbing brush	103.92	51.96	39.43	0.00	39.43	0.00	**39.12**
Needle (green)	3.33	3.33	3.63	3.63	0.00	3.63	**2.92**
Needle counter	252.74	252.74	254.94	254.94	254.94	254.94	**254.20**
Nonwoven dressing (10x20 cm)	24.66	24.66	23.48	23.48	23.48	23.48	**23.87**
Nonwoven dressing (10x30 cm)	49.99	49.99	0.00	0.00	0.00	0.00	**16.66**
Pre-operative adhesive glove	41.97	41.97	11.80	11.80	11.80	0.00	**19.89**
Reinforced skin closure strip	8.92	8.92	0.00	0.00	0.00	0.00	**2.97**
Shaver head	47.46	47.46	46.96	46.96	46.96	0.00	**39.30**
Specimen pot (40 ml 4% formaldehyde)	132.52	132.52	0.00	0.00	0.00	0.00	**44.17**
Surgical blade (10)	7.92	7.92	8.10	8.10	8.10	8.10	**8.04**
Surgical suspensory bandage	201.21	201.21	0.00	0.00	0.00	0.00	**67.07**
Suture (braided, absorbable, 0)	16.89	0.00	0.00	0.00	0.00	0.00	**2.82**
Suture (braided, absorbable, 2-0)	16.89	16.89	72.57	36.28	18.14	18.14	**29.82**
Suture (monofilament, absorbable, 3-0)	16.89	16.89	18.14	18.14	18.14	18.14	**17.73**
Suture (monofilament, nonabsorbable, 1)	16.89	16.89	0.00	0.00	18.14	18.14	**11.68**
Suture (monofilament, nonabsorbable, 2-0)	50.67	50.67	36.28	0.00	0.00	0.00	**22.94**
Syringe (20 ml)	215.40	215.40	65.05	65.05	0.00	65.05	**104.33**
Carbon footprint single-use equipment and medical devices (g CO_2e)	3,927.07	3,539.39	2,214.85	2,344.80	1,803.36	1,744.01	**2,595.58**

Chlorhexidine 1% from 500 ml container (100 ml)	0.00	0.00	107.30	107.30	107.30	107.30	**71.53**
Levobupivacaine 5mg/10 ml (10 ml)	1446.45	1446.45	0.00	0.00	0.00	717.91	**601.80**
Povidone iodine 10% from 500 ml container (60 ml)	183.24	183.24	0.00	0.00	0.00	0.00	**61.08**
Sodium Chloride 0.9% from 1 litre bottle (50 ml)	37.94	37.94	37.94	37.94	37.94	37.94	**37.94**
Topical skin adhesive (0.8 g)	0.00	0.00	0.00	3924.51	0.00	0.00	**654.09**
Carbon footprint pharmaceuticals (g CO_2e)	1,667.63	1,667.63	145.23	4,069.74	145.23	863.14	**1,426.43**
Black waste bag (domestic waste) with cable tie	104.60	104.60	0.00	0.00	0.00	0.00	**34.87**
Chlorine tablet	14.03	14.03	0.00	0.00	0.00	0.00	**4.68**
Clear bin bag (for swab count)	72.21	72.21	0.00	0.00	0.00	0.00	**24.07**
Clear bin bag (recycling) with cable tie	0.00	0.00	65.63	65.63	65.63	65.63	**43.75**
Disinfectant sachet	0.00	0.00	34.36	34.36	34.36	34.36	**22.90**
Disinfectant wipe	177.26	110.79	143.30	122.83	143.30	143.30	**140.13**
Green bag (linen laundering) with cable tie	17.42	17.42	17.42	17.42	17.42	17.42	**17.42**
Mop head	202.32	202.32	189.63	189.63	189.63	189.63	**193.86**
Orange waste bag (infectious waste, large) with cable tie	0.00	0.00	307.44	307.44	307.44	307.44	**204.96**
Orange waste bag (infectious waste, small) with cable tie	0.00	0.00	136.03	136.03	136.03	136.03	**90.69**
Red bag (linen laundering) with cable tie	32.32	32.32	32.32	32.32	32.32	32.32	**32.32**
Yellow waste bag (clinical waste) with cable tie	153.14	153.14	0.00	0.00	0.00	0.00	**51.05**
Carbon footprint cleaning products, waste (g CO_2e)	773.29	706.81	926.13	905.66	926.13	926.13	**860.69**
**Total Carbon footprint (g CO_2e)**	**12,439.78**	**11,675.91**	**10,593.23**	**14,963.44**	**10,273.18**	**10,127.69**	**11,678.87**

Supplementary Table 19: Carbon footprint of knee arthroplasty operations impact assessment
K1= knee arthroplasty operation one etc. CO_2e=carbon dioxide equivalents

**Item**	**Carbon footprint (g CO_2e)**										
	**K1**	**K2**	**K3**	**K4**	**K5**	**K6**	**K7**	**K8**	**K9**	**K10**	**Mean average**
Basic major orthopaedic set	2,288.88	2,288.88	2,288.88	2,288.88	2,288.88	2,288.88	2,288.88	2,288.88	2,288.88	2,288.88	**2,288.88**
Bipolar diathermy	0	0	0	0	0	0	223.04	0	0	0	**22.30**
Blunt Hohman bone elevator	0	0	0	0	0	0	0	199.17	199.17	0	**39.83**
Cruciate retaining femoral and tibial preparation and trialing set (size 1,2,7,8)	0	0	0	0	0	2,365.63	2,365.63	0	0	0	**473.13**
Cruciate retaining femoral and tibial trialing set (size 3-6)	0	2,229.12	2,229.12	2,229.12	2,229.12	2,229.12	2,229.12	2,229.12	2,229.12	2,229.12	**2,006.21**
Diathermy extras	0	0	0	0	0	277.33	0	0	0	0	**27.73**
Diathermy lead	0	0	0	0	0	0	220.93	0	0	0	**22.09**
Femoral and tibial preparation set (size 3-6)	2,235.42	2,235.42	2,235.42	2,235.42	2,235.42	2,235.42	2,235.42	2,235.42	2,235.42	2,235.42	**2,235.42**
Knee navigation set	0	0	0	0	0	0	0	2,245.46	0	0	**224.55**
Lanes tissue forceps	198.68	0	198.68	0	0	0	0	198.68	198.68	198.68	**99.34**
Light cover	198.18	198.18	198.18	198.18	198.18	198.18	198.18	198.18	198.18	198.18	**198.18**
Light handle	198.17	198.17	198.17	396.33	198.17	198.17	198.17	198.17	198.17	198.17	**217.98**
Miscellaneous knee system set	2,239.23	2,239.23	2,239.23	2,239.23	2,239.23	2,239.23	2,239.23	2,239.23	2,239.23	2,239.23	**2,239.23**
Non-toothed lamina spreader (large)	0	223.88	223.88	0	0	0	0	0	0	0	**44.78**
Non-toothed lamina spreader (small)	0	0	0	200.58	0	0	0	0	0	0	**20.06**
Orthopaedic surgical drill set	2,255.92	2,255.92	2,255.92	2,255.92	2,255.92	2,255.92	2,255.92	2,255.92	2,255.92	2,255.92	**2,255.92**

Patella preparation and trialing set		2,239.47	0	2,239.47	2,239.47	0	2,239.47	2,239.47	2,239.47	2,239.47	0	**1,567.63**
Posterior stabilised femoral and tibial trialing set (size 3-6)		2,249.72	0	0	0	2,249.72	0	0	2,249.72	0	0	**674.92**
Semb bone holding forceps		187.57	187.57	187.57	0	0	187.57	187.57	187.57	187.57	187.57	**150.06**
Carbon footprint reusable sets and individually wrapped instruments (g CO_2e)		14,291.24	12,056.37	14,494.52	14,283.14	13,894.63	16,714.91	16,881.56	18,964.54	14,469.81	12,031.17	**14,808.19**
Reusable scrubs		464.31	541.70	541.70	386.93	464.31	386.93	386.93	386.93	386.93	386.93	**433.36**
Carbon footprint reusable personal protective equipment (g CO_2e)		464.31	541.70	541.70	386.93	464.31	386.93	386.93	386.93	386.93	386.93	**433.36**
Reusable shaver base		0	0	0	0	0.44	0	0.44	0	0	0	**0.09**
Carbon footprint reusable non-set equipment (g CO_2e)		0	0	0	0	0.44	0	0.44	0	0	0	**0.09**
Knee pack	Surgical gown	1,089.14	1,089.14	1,089.14	1,089.14	1,089.14	1,089.14	1,089.14	1,089.14	1,089.14	1,089.14	**1,089.14**
	Knee pack packaging (gown component)	55.62	55.62	55.62	55.62	55.62	55.62	55.62	55.62	55.62	55.62	**55.62**
Gloves (non-sterile, pair)		154.38	154.38	176.44	154.38	154.38	110.27	132.33	220.55	154.38	154.38	**156.59**
Orthopaedic hood		2,023.32	1,331.30	1,331.30	1,348.88	2,023.32	0.00	0.00	1,348.88	2,023.32	2,023.32	**1,345.37**
Sterile gloves (pair)		0.00	620.73	620.73	869.02	744.87	744.87	993.17	620.73	620.73	869.02	**670.39**
Sterile gloves (two pairs)		1,483.53	370.88	370.88	556.33	185.44	185.44	370.88	370.88	556.33	556.33	**500.69**
Sterile gloves (two pair, latex free)		0.00	0.00	180.69	0.00	180.69	180.69	0.00	0.00	0.00	0.00	**54.21**
Surgical face mask		52.31	69.75	34.87	52.31	34.87	52.31	17.44	87.19	69.75	34.87	**50.57**
Surgical face mask with eye protection		37.72	37.72	113.16	37.72	37.72	113.16	75.44	0.00	0.00	0.00	**45.26**
Surgical gown (including hand towels)		2,033.38	1,336.08	1,336.08	2,033.38	2,033.38	1,336.08	1,336.08	2,033.38	2,711.18	2,033.38	**1,826.14**
Surgical hat		84.95	99.10	99.10	70.79	84.95	28.32	28.32	70.79	70.79	70.79	**70.79**
Sweat bands for hood x3		58.50	39.00	39.00	39.00	58.50	0.00	0.00	39.00	58.50	58.50	**39.00**

Carbon footprint single-use personal protective equipment (g CO_2e)		7,072.85	5,203.69	5,447.00	6,306.56	6,682.88	3,915.40	4,117.91	5,936.15	7,409.72	6,945.35	**5,903.75**
Knee pack	Patient drape (240x150 cm)	1,163.39	1,163.39	1,163.39	1,163.39	1,163.39	1,163.39	1,163.39	1,163.39	1,163.39	1,163.39	**1,163.39**
	Patient drape (90 x75 cm)	228.66	228.66	228.66	228.66	228.66	228.66	228.66	228.66	228.66	228.66	**228.66**
	Patient drape (extremity, 230x325 cm)	1,635.81	1,635.81	1,635.81	1,635.81	1,635.81	1,635.81	1,635.81	1,635.81	1,635.81	1,635.81	**1,635.81**
	Patient drape (impervious, split, 152x177 cm)	644.18	644.18	644.18	644.18	644.18	644.18	644.18	644.18	644.18	644.18	**644.18**
	Patient drape (Mayo cover, 75x144 cm) x2	842.38	842.38	842.38	842.38	842.38	842.38	842.38	842.38	842.38	842.38	**842.38**
	Patient drape (pouch fluid collection, 40x35 cm)	1,287.00	1,287.00	1,287.00	1,287.00	1,287.00	1,287.00	1,287.00	1,287.00	1,287.00	1,287.00	**1,287.00**
	Patient drape (stockinette, impervious, 30x120 cm)	583.69	583.69	583.69	583.69	583.69	583.69	583.69	583.69	583.69	583.69	**583.69**
	Table drape (140x90 cm)	1,405.53	1,405.53	1,405.53	1,405.53	1,405.53	1,405.53	1,405.53	1,405.53	1,405.53	1,405.53	**1,405.53**
	Table drape (fan folded, 140x190 cm)	1,038.07	1,038.07	1,038.07	1,038.07	1,038.07	1,038.07	1,038.07	1,038.07	1,038.07	1,038.07	**1,038.07**
	Knee pack packaging (patient and instrument table drape component)	342.79	342.79	342.79	342.79	342.79	342.79	342.79	342.79	342.79	342.79	**342.79**
Patient drape (adhesive split sheet)		0.00	0.00	0.00	0.00	0.00	0.00	0.00	990.16	990.16	0.00	**198.03**
Patient drape (clear U drape)		0.00	440.77	440.77	440.77	440.77	0.00	0.00	0.00	0.00	440.77	**220.38**
Patient drape (incise drape)		0.00	0.00	0.00	0.00	0.00	290.25	290.25	0.00	0.00	0.00	**58.05**
Patient drape (incise drape, with iodine)		240.07	240.07	240.07	240.07	240.07	0.00	0.00	0.00	0.00	240.07	**144.04**
Table drape (instruments)		3,008.99	3,008.99	3,008.99	3,008.99	3,008.99	3,008.99	3,008.99	3,008.99	3,008.99	3,008.99	**3,008.99**
Carbon footprint single-use patient and instrument table drapes (g CO_2e)		12,420.56	12,861.33	12,861.33	12,861.33	12,861.33	12,470.74	12,470.74	13,170.64	13,170.64	12,861.33	**12,800.99**
Knee pack	Bowl (250 ml) x2	66.99	66.99	66.99	66.99	66.99	66.99	66.99	66.99	66.99	66.99	**66.99**
	Bowl (500 ml)	91.73	91.73	91.73	91.73	91.73	91.73	91.73	91.73	91.73	91.73	**91.73**

	Cast padding bandage x2	187.95	187.95	187.95	187.95	187.95	187.95	187.95	187.95	187.95	187.95	**187.95**
	Crepe bandage x2	550.02	550.02	550.02	550.02	550.02	550.02	550.02	550.02	550.02	550.02	**550.02**
	Diathermy bag	62.49	62.49	62.49	62.49	62.49	62.49	62.49	62.49	62.49	62.49	**62.49**
	Diathermy tip cleaner	10.97	10.97	10.97	10.97	10.97	10.97	10.97	10.97	10.97	10.97	**10.97**
	Kidney dish x3	114.82	114.82	114.82	114.82	114.82	114.82	114.82	114.82	114.82	114.82	**114.82**
	Light cover	14.14	14.14	14.14	14.14	14.14	14.14	14.14	14.14	14.14	14.14	**14.14**
	Monopolar diathermy	240.15	240.15	240.15	240.15	240.15	240.15	240.15	240.15	240.15	240.15	**240.15**
	Needle counter	214.29	214.29	214.29	214.29	214.29	214.29	214.29	214.29	214.29	214.29	**214.29**
	Skin marker pen	22.05	22.05	22.05	22.05	22.05	22.05	22.05	22.05	22.05	22.05	**22.05**
	Suction tubing	436.63	436.63	436.63	436.63	436.63	436.63	436.63	436.63	436.63	436.63	**436.63**
	Surgical blade (10) x3	8.10	8.10	8.10	8.10	8.10	8.10	8.10	8.10	8.10	8.10	**8.10**
	Swab gauze (10x7.5 cm) x5	32.48	32.48	32.48	32.48	32.48	32.48	32.48	32.48	32.48	32.48	**32.48**
	Swab gauze (30x30 cm) x10	135.15	135.15	135.15	135.15	135.15	135.15	135.15	135.15	135.15	135.15	**135.15**
	Towel dressing (2 in pack)	43.62	43.62	43.62	43.62	43.62	43.62	43.62	43.62	43.62	43.62	**43.62**
	Tray (small)	589.06	589.06	589.06	589.06	589.06	589.06	589.06	589.06	589.06	589.06	**589.06**
	Yankauer sucker	65.70	65.70	65.70	65.70	65.70	65.70	65.70	65.70	65.70	65.70	**65.70**
	Knee pack packaging (equipment component)	145.07	145.07	145.07	145.07	145.07	145.07	145.07	145.07	145.07	145.07	**145.07**
Adhesive operative towel		0	0	0	309.29	0	0	0	0	0	0	**30.93**
Batteries for knee navigation set (x3)		0	0	0	0	0	0	0	286.07	0	0	**28.61**
Border dressing (10x30 cm)		47.76	47.76	47.76	47.76	95.51	47.76	95.51	47.76	47.76	95.51	**62.08**

Border dressing (6x8 cm)	0	0	0	0	0	0	0	11.24	11.24	0	**2.25**
Catheter tip syringe	0.00	79.10	0	0	0	0	0	0	0	0	**7.91**
Cement mixing and delivery system	1,314.98	1,314.98	1,314.98	1,314.98	1,314.98	1,314.98	1,314.98	1,314.98	1,314.98	1,314.98	**1,314.98**
Cement mixing bowl	0	0	0	0	0	0	2035.94	0	0	0	**203.59**
Crepe bandage (15 cm wide)	0	0	0	0	605.98	0	0	0	0	0	**60.60**
Cruciate retaining femoral implant	0.00	5,518.27	5,518.27	0	0	5,518.27	0	0	5,518.27	5,518.27	**2,759.13**
Diathermy bag	0	0	75.26	75.26	0	0	0	75.26	75.26	0	**30.11**
Diathermy tip	0	0	0	0	0	0	0	0	0	17.08	**1.71**
Diathermy tip cleaner	69.67	69.67	69.67	69.67	69.67	69.67	69.67	69.67	69.67	69.67	**69.67**
Elasticated fabric dressing strip	4.45	4.45	4.45	4.45	4.45	4.45	4.45	4.45	4.45	4.45	**4.45**
Gauze (individual piece)	37.19	37.19	37.19	37.19	0	0	0	0	0	0	**14.88**
Gauze (sterile pack)	201.49	0	0	0	0	0	0	0	0	0	**20.15**
High-vacuum wound drainage	0	0	0	0	0	745.53	745.53	0	0	0	**149.11**
Incontinence pad	401.59	602.39	200.80	200.80	200.80	200.80	200.80	200.80	200.80	200.80	**261.03**
Intravenous infusion giving set	116.82	116.82	116.82	116.82	116.82	116.82	116.82	0.00	0.00	116.82	**93.45**
Marker pen and ruler	0	0	0	0	0	0	0	23.71	0	0	**2.37**
Monopolar diathermy	0	0	0	0	0	0	0	0	147.64	0	**14.76**
Nail scrubbing brush	0	0	0	134.85	134.85	134.85	134.85	134.85	134.85	134.85	**94.40**
Needle (green)	3.63	3.63	3.63	3.63	0	0	0	0	0	0	**1.45**
Needle (red)	5.62	5.62	5.62	5.62	5.62	5.62	5.62	0.00	0.00	5.62	**4.50**

Needle (white)	9.32	9.32	9.32	9.32	9.32	4.66	4.66	9.32	9.32	9.32	**8.39**
Posterior stabilised femoral implant	8821.07	0	0	8821.07	8821.07	0	0	8821.07	0	0	**3528.43**
Pre-operative adhesive glove	0	0	0	0	11.80	0	11.80	0	0	0	**2.36**
Primary tibial baseplate implant	2,996.83	2,996.83	2,996.83	2,996.83	2,996.83	2,996.83	2,996.83	2,996.83	2,996.83	2,996.83	**2,996.83**
Pulsed lavage system	3,726.63	3,726.63	3,726.63	3,726.63	3,726.63	3,726.63	3,726.63	3,726.63	3,726.63	3,726.63	**3,726.63**
Saw blade	186.52	186.52	186.52	373.05	186.52	186.52	186.52	186.52	186.52	186.52	**205.18**
Self-adherent bandage	342.20	0	0	0	0	0	0	0	0	0	**34.22**
Shaver head	0	0	0	0	46.96	0.00	46.96	0	0	0	**9.39**
Skin stapler	0	0	0	368.11	368.11	0	0	368.11	368.11	368.11	**184.06**
Sticky label	1.34	1.34	1.34	1.34	1.34	0	0	0	0	0	**0.67**
Suction receptacle	192.38	192.38	192.38	192.38	192.38	192.38	192.38	192.38	192.38	192.38	**192.38**
Suction tip	0	0	0	0	0	0	58.07	0	0	0	**5.81**
Suction tubing	0	0	492.93	0	0	492.93	0	0	0	0	**98.59**
Surgical blade (10)	0.00	8.10	8.10	0	0	0	8.10	0	0	0	**2.43**
Suture (braided, absorbable, 2-0)	25.83	25.83	25.83	25.83	0	0	0	25.83	25.83	25.83	**18.08**
Suture (monofilament, absorbable, 3-0, 643)	0	0	0	0	0	17.00	17.00	0	0	0	**3.40**
Suture (monofilament, absorbable 3-0, 696)	33.99	33.99	33.99	0	0	0	0	0	0	0	**10.20**
Suture (braided, absorbable 1-0, 803)	25.83	25.83	25.83	77.48	0	77.48	77.48	51.65	51.65	77.48	**49.07**
Suture (braided, absorbable 1-0, 932)	0	0	0	0	71.13	0	0	0	0	0	**7.11**
Swab tray (large)	268.37	268.37	268.37	268.37	536.74	536.74	268.37	268.37	268.37	268.37	**322.05**

Swab tray (small)	128.39	128.39	128.39	128.39	128.39	128.39	128.39	128.39	128.39	128.39	**128.39**
Symmetric patella implant	512.49	0.00	512.49	512.49	0	512.49	512.49	512.49	512.49	0	**358.74**
Syringe (20 ml)	65.05	65.05	65.05	65.05	65.05	65.05	65.05	0	0	65.05	**52.04**
Syringe (50 ml)	273.84	273.84	273.84	136.92	136.92	273.84	273.84	547.68	547.68	273.84	**301.23**
Tibial bearing insert cruciate retaining implant	1014.01	1014.01	1014.01	1014.01	0	0	1014.01	0	1014.01	1014.01	**709.80**
Tibial bearing insert posterior stabilised implant	0	0	0	0	1109.59	0.00	2219.18	1109.59	0	0	**443.84**
Tourniquet pressure cuff (leg)	980.50	980.50	980.50	980.50	980.50	980.50	980.50	980.50	980.50	980.50	**980.50**
Transparent film adhesive dressing	15.02	0	0	0	0	0	15.02	0	0	0	**3.00**
Tubular support bandage	0	0	0	0	214.75	214.75	214.75	214.75	214.75	214.75	**128.85**
Wound closure strips	14.93	14.93	14.93	0	0	0	0	0	14.93	0	**5.97**
Carbon footprint single-use equipment and medical devices (g CO_2e)	24,869.11	20,783.12	21,383.10	25,049.46	25,184.11	21,596.31	20,773.59	25,340.27	21,794.69	21,037.44	**22,781.12**
Adrenaline 1mg in 1 ml (1 ml)	36.45	36.45	36.45	36.45	36.45	36.45	36.45	0.00	0.00	36.45	**29.16**
Bone cement mix (with gentamicin)	0.00	0.00	0.00	13,202.78	0.00	13,202.78	26,405.56	0.00	0.00	0.00	**5,281.11**
Bone cement mix (with tobramycin)	26,426.55	26,426.55	26,426.55	0.00	26,426.55	0.00	0.00	26,426.55	26,426.55	26,426.55	**18,498.59**
Chlorhexidine 2% from 500 ml bottle (150 ml)	160.94	160.94	160.94	160.94	160.94	0.00	0.00	0.00	0.00	160.94	**96.57**
Chlorhexidine gluconate in 70% denatured ethanol with 4 ml red stain solution from 200 ml bottle (150 ml)	982.90	0.00	0.00	982.90	982.90	0.00	0.00	0.00	0.00	982.90	**393.16**
Iodinated povidone 10% w/w alcoholic tincture from 500 ml bottle (50 ml)	0.00	0.00	0.00	92.16	0.00	92.16	92.16	92.16	92.16	0.00	**46.08**
Ketorolac tromethamine 30mg in 1 ml (1 ml)	262.16	262.16	262.16	262.16	262.16	262.16	262.16	0.00	0.00	262.16	**209.73**

Levobupivacaine 2.5mg/ml (10 ml)	0.00	0.00	0.00	0.00	0.00	0.00	0.00	957.21	957.21	0.00	**191.44**
Ropivacaine 75mg in 10 ml (10 ml)	779.33	779.33	779.33	779.33	779.33	779.33	779.33	0.00	0.00	779.33	**623.47**
Sodium chloride 0.9% (100 ml)	115.98	231.96	115.98	115.98	115.98	115.98	115.98	115.98	115.98	115.98	**127.58**
Sodium chloride 0.9% for irrigation (3 L bag)	917.91	917.91	917.91	917.91	917.91	917.91	917.91	917.91	917.91	917.91	**917.91**
Topical skin adhesive (0.8 g)	3,924.51	0.00	0.00	0.00	0.00	0.00	0.00	0.00	0.00	0.00	**392.45**
Carbon footprint pharmaceuticals (g CO_2e)	33,606.74	28,815.31	28,699.33	16,550.62	29,682.23	15,406.78	28,609.56	28,509.81	28,509.81	29,682.23	**26,807.24**
Anatomical waste bin	208.26	208.26	208.26	208.26	208.26	208.26	208.26	208.26	208.26	208.26	**208.26**
Clear bin bag (linen laundering)	20.86	20.86	20.86	20.86	20.86	20.86	20.86	20.86	20.86	20.86	**20.86**
Clear waste bag (recycling) with cable tie	65.63	65.63	65.63	65.63	65.63	65.63	65.63	65.63	65.63	65.63	**65.63**
Disinfectant sachet	137.43	137.43	137.43	137.43	137.43	137.43	137.43	137.43	137.43	137.43	**137.43**
Disinfectant wipe	704.87	634.38	704.87	225.56	874.04	310.14	352.44	281.95	352.44	422.92	**486.36**
Orange waste bag (infectious waste) with cable tie from theatre	614.88	614.88	614.88	614.88	614.88	614.88	614.88	614.88	614.88	614.88	**614.88**
Orange waste bag (infectious waste) with cable tie from scrub room	102.48	0.00	0.00	102.48	102.48	102.48	102.48	102.48	102.48	102.48	**81.98**
Mop head	252.84	252.84	252.84	252.84	252.84	252.84	252.84	252.84	252.84	252.84	**252.84**
Red bag (linen laundering) with cable tie	43.09	43.09	43.09	43.09	43.09	43.09	43.09	43.09	43.09	43.09	**43.09**
Yellow/ black waste bag (non-infectious offensive waste) with cable tie from scrub room	0.00	38.65	38.65	0.00	0.00	0.00	0.00	0.00	0.00	0.00	**7.73**
Carbon footprint cleaning products, waste (g CO_2e)	2,150.34	2,016.02	2,086.51	1,671.03	2,319.51	1,755.61	1,797.90	1,727.42	1,797.90	1,868.39	**1,919.06**
**Total Carbon footprint (g CO_2e)**	**94,875.15**	**82,277.53**	**85,513.48**	**77,109.05**	**91,089.44**	**72,246.68**	**85,038.63**	**94,035.76**	**87,539.51**	**84,812.83**	**85,453.81**

Supplementary Table 20: Carbon footprint of laparoscopic cholecystectomy operations impact assessment

L1= laparoscopic cholecystectomy operation one etc. CO_2e=carbon dioxide equivalents

**Item**	**Carbon footprint (g CO_2e)**						
	**L1**	**L2**	**L3**	**L4**	**L5**	**L6**	**Mean average**
General basic set	2,395.86	2,395.86	2,395.86	2,395.86	2,395.86	2,395.86	**2,395.86**
General laparoscopic set	2,231.95	2,231.95	2,231.95	2,231.95	2,231.95	2,231.95	**2,231.95**
Laparoscope set (10 mm, 0 degree)	1,778.07	1,778.07	1,778.07	1,778.07	1,778.07	1,778.07	**1,778.07**
Diathermy lead	0	0	204.22	0	0	0	**34.04**
Laparoscopic grasping forceps	0	0	0	216.35	0	0	**36.06**
Quiver and clip	198.08	198.08	198.08	198.08	198.08	198.08	**198.08**
Carbon footprint reusable sets and individually wrapped instruments (g CO_2e)	6,603.97	6,603.97	6,808.19	6,820.32	6,603.97	6,603.97	**6,674.06**
High fluid drape reusable components	1098.42	1098.42	1098.42	1076.84	1076.84	1076.84	**1087.63**
Huck towel reusable components	0	123.51	0	113.48	113.48	113.48	**77.33**
Carbon footprint reusable patient drapes (g CO_2e)	1,098.42	1,221.93	1,098.42	1,190.32	1,190.32	1,190.32	**1,164.96**
Surgical gown (including hand towels) reusable components	252.67	252.67	758.02	505.34	0	252.67	**336.90**
Surgical gown (including hand towels) double pack reusable components	0	405.54	0	405.54	405.54	405.54	**270.36**
Surgical hat	0	0	0	3.66	3.66	3.66	**1.83**
Scrubs	406.27	406.27	348.23	348.23	348.23	348.23	**367.58**
Carbon footprint reusable personal protective equipment (g CO_2e)	658.94	1,064.49	1,106.25	1,262.78	757.44	1,010.11	**976.67**
Diathermy pad lead	9.24	9.24	9.24	9.24	9.24	9.24	**9.24**
Carbon footprint non-set reusable equipment (g CO_2e)	9.24	9.24	9.24	9.24	9.24	9.24	**9.24**

Table drape (instrument)	775.39	775.39	775.39	705.47	705.47	705.47	**740.43**
Carbon footprint single-use patient drape and instrument table drape (g CO_2e	775.39	775.39	775.39	705.47	705.47	705.47	**740.43**
Gloves (non-sterile, pair)	176.44	154.38	132.33	99.94	119.93	59.96	**123.83**
Sterile gloves (pair)	0	119.42	358.26	221.34	221.34	221.34	**190.28**
Sterile gloves (two pairs)	535.79	535.79	0	165.10	165.10	330.19	**288.66**
Sterile gloves (one pair, latex free)	0	0	102.47	0	0	0	**17.08**
Surgical face mask	0	34.87	52.31	48.12	32.08	64.16	**38.59**
Surgical face mask with eye protection	37.72	37.72	0	0	34.43	0	**18.31**
Surgical gown (including hand towels)	1320.38	0	0	0	613.31	613.31	**424.50**
Surgical hat	24.78	24.78	21.24	16.28	16.28	16.28	**19.94**
Carbon footprint single-use personal protective equipment (g CO_2e	2,095.10	906.96	666.60	550.77	1,202.47	1,305.25	**1,121.19**
Absorbent towel pack	75.77	0	0	0	0	0	**12.63**
Anti-fog endoscopic demister	0	682.36	682.36	674.57	674.57	674.57	**564.74**
Diathermy pad	69.67	69.67	69.67	65.42	65.42	65.42	**67.55**
Endoscopic clip applier	1646.34	1646.34	1646.34	1532.96	1532.96	1532.96	**1589.65**
Gauze (sterile pack)	318.22	159.11	159.11	300.64	300.64	300.64	**256.39**
Incontinence pad	75.73	75.73	75.73	66.06	132.12	66.06	**81.91**
Insufflating tubing	627.27	627.27	627.27	571.93	571.93	571.93	**599.60**
Laparoscope cover	146.42	146.42	146.42	131.68	131.68	131.68	**139.05**
Laparoscopic scissors	668.09	668.09	668.09	651.91	0.00	651.91	**551.35**

Laparoscopic tissue retrieval system	40.48	40.48	40.48	37.11	37.11	37.11	**38.80**
Light handle	29.84	0	0	0	0	0	**4.97**
Nail scrubbing brush	0	39.43	39.43	39.43	39.43	39.43	**32.86**
Needle (green)	3.63	3.63	3.63	3.33	3.33	3.33	**3.48**
Needle counter	254.94	254.94	254.94	252.74	252.74	252.74	**253.84**
Nonwoven dressing (6x7 cm)	35.45	35.45	35.45	31.38	23.53	31.38	**32.11**
Port (12 mm)	649.56	1299.11	649.56	1288.11	1288.11	1288.11	**1077.09**
Port (5 mm, dual pack)	657.66	657.66	657.66	652.17	652.17	652.17	**654.92**
Pressure saline infusion bag	0	0	679.18	0	0	0	**113.20**
Reinforced skin closure strip	0	7.80	7.80	0	7.08	0	**3.78**
Specimen pot (40 ml 4% formaldehyde)	0	126.60	126.60	0	0	0	**42.20**
Specimen pot (not pre-filled)	145.56	0	0	136.34	136.34	136.34	**92.43**
Suction irrigation	0	0	1576.63	1440.58	0	1440.58	**742.96**
Suction receptacle	0	0	373.87	327.34	0	0	**116.87**
Surgical blade (11)	7.42	0	0	0	0	0	**1.24**
Surgical blade (15)	0	7.00	0	6.79	6.79	6.79	**4.56**
Suture (braided, absorbable, 3-0)	0	6.09	6.09	11.53	11.53	5.76	**6.83**
Suture (monofilament, absorbable, 3-0)	18.14	0	0	0	0	0	**3.02**
Suture (monofilament, absorbable, 1)	18.14	36.28	36.28	16.89	16.89	16.89	**23.56**
Syringe (10 ml)	27.67	27.67	27.67	25.33	25.33	25.33	**26.50**

Syringe (20 ml)	130.11	65.05	65.05	119.17	59.59	59.59	**83.09**
Tonsil swab pack	0.00	49.70	0	46.69	46.69	46.69	**31.63**
Carbon footprint single-use equipment and medical devices (g CO_2e)	5,646.10	6,731.89	8,655.33	8,430.09	6,015.98	8,037.40	**7,252.80**
Carbon dioxide (from 3kg cylinder containing 450 L)	171.00	307.80	616.20	864.00	447.00	133.80	**423.30**
Chlorhexidine 1% from 500 ml container (50 ml)	53.65	53.65	53.65	53.65	53.65	53.65	**53.65**
Levobupivacaine 2.5mg/10 ml (10 ml)	957.21	717.91	717.91	713.72	713.72	713.72	**755.70**
Sodium Chloride 0.9% for irrigation (1 L bag)	0	0	304.49	585.11	0.00	0.00	**148.27**
Sodium Chloride 0.9% from 1 litre bottle (50 ml)	37.94	37.94	37.94	37.94	37.94	37.94	**37.94**
Carbon footprint pharmaceuticals (g CO_2e)	1,219.79	1,117.29	1,730.18	2,254.42	1,252.30	939.10	**1,418.85**
Black waste bag (domestic waste) with cable tie	0	0	0	209.19	209.19	209.19	**104.60**
Clear bin bag (quiver diathermy)	59.78	59.78	59.78	59.78	59.78	59.78	**59.78**
Clear bin bag (recycling) with cable tie	65.63	65.63	65.63	0	0	0	**32.81**
Disinfectant sachet	34.36	34.36	34.36	33.78	33.78	33.78	**34.07**
Disinfectant wipe	163.78	143.30	102.36	77.61	291.03	116.41	**149.08**
Green bag (linen laundering) with cable tie	17.42	17.42	17.42	17.42	17.42	17.42	**17.42**
Mop head	189.63	189.63	189.63	181.58	181.58	181.58	**185.61**
Orange waste bag (infectious waste, large) with cable tie	307.44	307.44	307.44	0	0	0	**153.72**
Orange waste bag (infectious waste, small) with cable tie	136.03	136.03	136.03	0	0	0	**68.02**
Red bag (linen laundering) with cable tie	32.32	32.32	32.32	32.32	32.32	32.32	**32.32**
Yellow/ black waste bag (non-infectious offensive waste, large) with cable tie	0	0	0	115.94	115.94	115.94	**57.97**

Yellow/ black waste bag (non-infectious offensive waste, small) with cable tie	0	0	0	73.11	73.11	73.11	**36.56**
Carbon footprint cleaning products, waste (g CO_2e)	1,006.38	985.91	944.96	800.72	1,014.14	839.53	**931.94**
**Total Carbon footprint (g CO_2e)**	19,113.34	19,417.07	21,794.56	22,024.14	18,751.33	20,640.39	20,290.14

Supplementary Table 21: Carbon footprint of tonsillectomy operations impact assessment

T1= tonsillectomy operation one etc. CO_2e=carbon dioxide equivalents

**Item**	**Carbon footprint (g CO_2e)**										
	**T1**	**T2**	**T3**	**T4**	**T5**	**T6**	**T7**	**T8**	**T9**	**T10**	**Mean average**
Tonsillectomy set (set A for T1, T4-T10; set B for T2, T3)	2,279.47	2,364.29	2,364.29	2,279.47	2,279.47	2,279.47	2,279.47	2,279.47	2,279.47	2,279.47	**2,296.43**
Carbon footprint reusable set (g CO_2e)	2,279.47	2,364.29	2,364.29	2,279.47	2,279.47	2,279.47	2,279.47	2,279.47	2,279.47	2,279.47	**2,296.43**
Reusable ENT split head drape (two 42"x42")	0	475.15	460.08	0	0	0	0	0	0	0	**93.52**
Carbon footprint reusable patient drapes (g CO_2e)	0	475.15	460.08	0	0	0	0	0	0	0	**93.52**
Reusable surgical gown (including hand towels)	474.03	474.03	474.03	474.03	474.03	474.03	474.03	474.03	474.03	474.03	**474.03**
Reusable surgical hat	8.79	0	0	5.86	5.86	8.79	5.86	8.79	5.86	8.79	**5.86**
Reusable scrubs	185.72	185.72	185.72	185.72	185.72	278.59	232.16	325.02	278.59	278.59	**232.16**
Carbon footprint reusable personal protective equipment (g CO_2e)	668.54	659.75	659.75	665.61	665.61	761.40	712.04	807.83	758.47	761.40	**712.04**
Gloves (non-sterile, one pair)	25.31	44.11	44.11	50.62	50.62	25.31	25.31	25.31	75.93	75.93	**44.26**
Sterile gloves (one pair)	399.62	358.26	358.26	266.41	266.41	266.41	266.41	266.41	266.41	266.41	**298.10**
Surgical face mask	39.28	0	34.87	0	0	19.64	19.64	19.64	19.64	0	**15.27**
Surgical face mask with eye protection	0	75.44	0	0	0	42.90	42.90	42.90	0	0	**20.42**
Surgical hat	3.19	11.33	11.33	6.38	6.38	9.57	9.57	12.76	12.76	9.57	**9.28**
Carbon footprint single-use personal protective equipment (g CO_2e)	467.39	489.13	448.57	323.41	323.41	363.83	363.83	367.02	374.74	351.91	**387.32**
Patient drape (fenestrated ENT drape)	274.74	0	0	274.74	274.74	274.74	274.74	274.74	274.74	274.74	**219.79**

Table drape (instruments)	856.70	775.39	775.39	856.70	856.70	856.70	856.70	856.70	856.70	856.70	**840.44**
Carbon footprint single-use patient and instrument table drapes (g CO_2e)	1,131.44	775.39	775.39	1,131.44	1,131.44	1,131.44	1,131.44	1,131.44	1,131.44	1,131.44	**1,060.23**
Coblation™ wand	0	0	0	0	0	932.68	932.68	932.68	932.68	932.68	**466.34**
Kidney dish	73.04	0	0	0	0	0	0	0	0	0	**7.30**
Filter needle	5.50	0	0	0	0	0	0	0	0	0	**0.55**
Gallipot	42.29	0	0	0	0	84.58	84.58	84.58	84.58	84.58	**46.52**
Incontinence pad	0	0	75.73	0	0	0	0	0	0	0	**7.57**
Braided silk tonsil ties	47.75	94.33	47.16	0	0	0	0	0	0	0	**18.92**
Gauze (non-sterile)	31.26	0	0	0	0	0	0	0	0	0	**3.13**
Nail scrubbing brush	39.43	43.83	0	0	0	39.43	0	0	0	0	**12.27**
Suction tip	0	18.30	0	18.68	18.68	18.68	18.68	18.68	18.68	18.68	**14.90**
Suction receptacle	447.21	373.89	373.89	447.21	447.21	447.21	447.21	447.21	447.21	447.21	**432.55**
Suction tubing	550.66	492.93	489.47	550.66	550.66	550.66	550.66	550.66	550.66	550.66	**538.77**
Syringe (10 ml)	30.50	0	0	0	0	0	0	0	0	0	**3.05**
Tonsil swab pack	51.48	149.09	194.20	102.97	51.48	102.97	102.97	102.97	51.48	51.48	**96.11**
Yankauer sucker	91.36	83.67	83.67	91.36	91.36	91.36	91.36	91.36	91.36	91.36	**89.82**
Specimen pot (40 ml 4% formaldehyde)	0	157.83	0	0	0	0	0	0	0	0	**15.78**
Carbon footprint single-use equipment and medical devices (g CO_2e)	1,410.49	1,413.87	1,264.13	1,210.88	1,159.39	2,267.57	2,228.14	2,228.14	2,176.65	2,176.65	**1,753.59**

Bupivacaine 0.5% with 1:200,000 adrenaline (10 ml)	246.04	246.04	246.04	0	0	0	0	0	0	0	**73.81**
Chirocaine 2.5mg/ml (10 ml)	0	0	0	0	0	251.71	251.71	251.71	251.71	251.71	**125.85**
Sodium chloride 0.9% from 1 litre bottle (150 ml)	113.81	113.81	113.81	113.81	113.81	113.81	113.81	113.81	113.81	113.81	**113.81**
Sodium chloride 0.9%, intravenous infusion bag (500 ml)	0	0	0	0	0	291.44	291.44	291.44	291.44	291.44	**145.72**
Yellow soft paraffin BP 100% from 15g tube	0	0.85	0.85	0.85	0.85	0.85	0.85	0.85	0.85	0.85	**0.77**
Carbon footprint single-use pharmaceuticals (g CO_2e)	359.85	360.70	360.70	114.66	114.66	657.81	657.81	657.81	657.81	657.81	**459.96**
Black waste bag (domestic waste) with cable tie	104.60	0	0	104.60	104.60	104.60	104.60	104.60	104.60	104.60	**83.68**
Clear waste bag (for swab count)	83.70	72.20	72.20	83.70	83.70	83.70	83.70	83.70	83.70	83.70	**81.40**
Clear waste bag (recycling) with cable tie	65.63	131.26	131.26	65.63	65.63	65.63	65.63	65.63	65.63	65.63	**78.75**
Disinfectant sachet	27.02	27.02	27.02	27.02	27.02	27.02	27.02	27.02	27.02	27.02	**27.02**
Disinfectant wipe	76.29	84.58	84.58	45.78	45.78	91.55	61.03	61.03	122.07	76.29	**74.90**
Green bag (linen laundering) with cable tie	21.00	21.00	21.00	21.00	21.00	21.00	21.00	21.00	21.00	21.00	**21.00**
Mop head	161.85	151.71	151.71	161.85	161.85	161.85	161.85	161.85	161.85	161.85	**159.82**
Orange waste bag (infectious waste) with cable tie	0	307.44	307.44	0	0	0	0	0	0	0	**61.49**
Yellow waste bag (clinical waste) with cable tie	153.14	0	0	153.14	153.14	153.14	153.14	153.14	153.14	153.14	**122.52**
Carbon footprint cleaning products, waste (g CO_2e)	693.24	795.21	795.21	662.72	662.72	708.50	677.98	677.98	739.01	693.24	**710.58**
**Total Carbon footprint (g CO_2e)**	**7,010.41**	**7,333.49**	**7,128.11**	**6,388.19**	**6,336.71**	**8,170.02**	**8,050.70**	**8,149.69**	**8,117.59**	**8,051.92**	**7,473.68**

Supplementary Table 22: Carbon footprint of products used in operations by product category.
C1=carpal tunnel decompression operation one etc. 'Instrument set and individually wrapped instruments' carbon footprint includes single-use sterile barrier system and single-use items within the set where relevant. CO_2e= carbon dioxide equivalents

**Operation**		**Carbon footprint (g CO_2e)**											
		**Reusable items**					**Single-use items**						
		Instrument sets and individually wrapped instruments	Personal protective equipment	Patient and/or instrument table drapes	Non-set equipment	**Total reusable items**	Personal protective equipment	Patient and/or instrument table drapes	Equipment and medical devices	Pharma -ceuticals	Cleaning products, waste	**Total single-use items**	**Total for operation**
Carpal tunnel decompression	C1	1,922	232	N/A	79	**2,234**	2,720	3,881	1,789	735	699	**9,825**	**12,058**
	C2	1,922	232	N/A	79	**2,234**	2,720	3,881	1,902	735	685	**9,924**	**12,158**
	C3	1,922	232	N/A	79	**2,234**	2,720	3,881	2,054	735	657	**10,047**	**12,281**
	C4	1,922	279	N/A	79	**2,280**	2,677	3,881	2,245	1,123	657	**10,583**	**12,863**
	C5	1,922	325	N/A	N/A	**2,247**	2,609	3,881	1,778	885	643	**9,796**	**12,043**
	C6	1,922	232	N/A	N/A	**2,154**	2,583	3,881	1,476	885	657	**9,482**	**11,636**
	C7	1,922	371	N/A	N/A	**2,294**	2,665	3,881	1,431	885	629	**9,491**	**11,784**
	C8	1,922	232	N/A	79	**2,234**	3,307	3,881	1,388	422	756	**9,754**	**11,988**
	C9	1,922	232	N/A	79	**2,234**	1,890	3,881	1,509	1,123	657	**9,060**	**11,294**
	C10	1,922	279	N/A	79	**2,280**	3,330	3,881	1,418	422	671	**9,722**	**12,003**
	Mean C1-C10	1,922	265	N/A	56	**2,242**	2,722	3,881	1,699	795	671	**9,768**	**12,011**
	Percentage of total	16 %	2 %	N/A	0.46 %	**19 %**	23%	32 %	14 %	7 %	6 %	**81 %**	**100%**
Inguinal hernia repair	H1	3,214	591	745	10	**4,559**	1,512	N/A	3,927	1,668	773	**7,880**	**12,440**
	H2	3,017	533	745	10	**4,304**	1,458	N/A	3,539	1,668	707	**7,372**	**11,676**
	H3	2,396	356	1,098	10	**3,860**	2,672	775	2,215	145	926	**6,734**	**10,593**
	H4	2,593	796	1,222	10	**4,620**	1,356	1,667	2,345	4,070	906	**10,343**	**14,963**
	H5	2,396	290	1,222	10	**3,918**	2,705	775	1,803	145	926	**6,356**	**10,273**
	H6	2,396	796	1,222	9	**4,423**	1,396	775	1,744	863	926	**5,705**	**10,128**
	MeanH1-H6	2,669	560	1,042	10	**4,281**	1,850	666	2,596	1,426	861	**7,398**	**11,679**
	Percentage of total	23 %	5 %	9 %	0.08 %	**37**	16%	6 %	22 %	12 %	7 %	**63 %**	**100 %**
Kne	K1	14,291	464	N/A	N/A	**14,756**	7,073	12,421	24,869	33,607	2,150	**80,120**	**94,875**

	K2	12,056	542	N/A	N/A	**12,598**	5,204	12,861	20,783	28,815	2,016	**69,679**	**82,278**
	K3	14,495	542	N/A	N/A	**15,036**	5,447	12,861	21,383	28,699	2,087	**70,477**	**85,513**
	K4	14,283	387	N/A	N/A	**14,670**	6,307	12,861	25,049	16,551	1,671	**62,439**	**77,109**
	K5	13,895	464	N/A	0.44	**14,359**	6,683	12,861	25,184	29,682	2,320	**76,730**	**91,089**
	K6	16,715	387	N/A	N/A	**17,102**	3,915	12,471	21,596	15,407	1,756	**55,145**	**72,247**
	K7	16,882	387	N/A	0.44	**17,269**	4,118	12,471	20,774	28,610	1,798	**67,770**	**85,039**
	K8	18,965	387	N/A	N/A	**19,351**	5,936	13,171	25,340	28,510	1,727	**74,684**	**94,036**
	K9	14,470	387	N/A	N/A	**14,857**	7,410	13,171	21,795	28,510	1,798	**72,683**	**87,540**
	K10	12,031	387	N/A	N/A	**12,418**	6,945	12,861	21,037	29,682	1,868	**72,395**	**84,813**
	Mean K1-K6	14,808	433	N/A	0.09	**15,242**	5,904	12,801	22,781	26,807	1,919	**70,212**	**85,454**
	Percentage of total	17 %	1 %	N/A	0.0001 %	**18 %**	7%	15 %	27 %	31 %	2 %	**82 %**	**100 %**
Laparoscopic cholecystectomy	L1	6,604	659	1,098	9	**8,371**	2,095	775	5,646	1,220	1,006	**10,743**	**19,113**
	L2	6,604	1,064	1,222	9	**8,900**	907	775	6,732	1,117	986	**10,517**	**19,417**
	L3	6,808	1,106	1,098	9	**9,022**	667	775	8,655	1,730	945	**12,772**	**21,795**
	L4	6,820	1,263	1,190	9	**9,283**	551	705	8,430	2,254	801	**12,741**	**22,024**
	L5	6,604	757	1,190	9	**8,561**	1,202	705	6,016	1,252	1,014	**10,190**	**18,751**
	L6	6,604	1,010	1,190	9	**8,814**	1,305	705	8,037	939	840	**11,827**	**20,640**
	Mean L1-L6	6,674	977	1,165	9	**8,825**	1,121	740	7,253	1,419	932	**11,465**	**20,290**
	Percentage of total	33 %	5 %	6 %	0.05 %	**43 %**	6%	4 %	36 %	7 %	5 %	**57 %**	**100 %**
Tonsillectomy	T1	2,279	669	N/A	N/A	**2,948**	467	1,131	1,410	360	693	**4,062**	**7,010**
	T2	2,364	660	475	N/A	**3,499**	489	775	1,414	361	795	**3,834**	**7,333**
	T3	2,364	660	460	N/A	**3,484**	449	775	1,264	361	795	**3,644**	**7,128**
	T4	2,279	666	N/A	N/A	**2,945**	323	1,131	1,211	115	663	**3,443**	**6,388**
	T5	2,279	666	N/A	N/A	**2,945**	323	1,131	1,159	115	663	**3,392**	**6,337**
	T6	2,279	761	N/A	N/A	**3,041**	364	1,131	2,268	658	708	**5,129**	**8,170**
	T7	2,279	712	N/A	N/A	**2,992**	364	1,131	2,228	658	678	**5,059**	**8,051**
	T8	2,279	808	N/A	N/A	**3,087**	367	1,131	2,228	658	678	**5,062**	**8,150**
	T9	2,279	758	N/A	N/A	**3,038**	375	1,131	2,177	658	739	**5,080**	**8,118**
	T10	2,279	761	N/A	N/A	**3,041**	352	1,131	2,177	658	693	**5,011**	**8,052**
	Mean T1-T10	2,296	712	94	N/A	**3,102**	387	1,060	1,754	460	711	**4,372**	**7,474**
	Percentage of total	31 %	10 %	1 %	N/A	**42 %**	5%	14 %	23 %	6 %	10 %	**58 %**	**100 %**

Supplementary Table 23: Mean average carbon footprint of products used for carpal tunnel decompression

Mean average carbon footprint of items used across ten carpal tunnel decompression operations (C1-C10), in order of contribution. Mean is dependent upon number of items used, and number of operations using the item. CO_2e= carbon dioxide equivalents.

**Product category**	**Product**	**Mean average carbon footprint (g CO_2e)**	**Percentage of mean average total (%)**	**Cumulative percentage (%)**
Single-use patient or instrument table drape	Patient drape (fenestrated hand drape)	2,740	22.81	22.81
Single-use personal protective equipment	Surgical gown (including hand towels)	2,063	17.17	39.99
Single-use patient or instrument table drape	Table drape (instruments)	1,050	8.74	48.73
Single-use equipment and medical devices	Crepe bandage (7.5 cm wide)	309	2.57	51.30
Cleaning products, waste bags	Orange waste bag (infectious waste) with cable tie from theatre	307	2.56	53.86
Single-use personal protective equipment	Sterile gloves (pair)	298	2.48	56.34
Reusable components from reusable set (minor op set)	Bipolar lead	268	2.23	58.57
Reusable personal protective equipment	Reusable scrubs	265	2.20	60.77
Single-use components from reusable set (minor op set)	Tray wrap (outer, 90x90 cm)	244	2.03	62.80
Single-use equipment and medical devices	Needle counter	237	1.97	64.78
Single-use equipment and medical devices	Gauze (sterile pack)	224	1.86	66.64
Reusable components from reusable set (minor op set)	Sponge holder forceps	222	1.85	68.48
Pharmaceuticals	Lidocaine 1% with adrenaline 1:200,000 (20 ml)	211	1.75	70.24
Single-use equipment and medical devices	Gauze swab	188	1.57	71.81
Reusable components from reusable set (minor op set)	Weitlaner retractor (3x4 teeth)	181	1.51	73.32
Cleaning products, waste bags	Mop head	152	1.26	74.58
Pharmaceuticals	Lidocaine 1% (10 ml)	151	1.26	75.83
Single-use equipment and medical devices	Kidney dish	147	1.22	77.06
Single-use equipment and medical devices	Bowl	127	1.06	78.12
Pharmaceuticals	Bupivacaine hydrochloride 0.5% (10 ml)	125	1.04	79.16
Single-use components from reusable set (minor op set)	Tray wrap (inner, 90x90 cm)	123	1.02	80.18
Reusable components from reusable set (minor op set)	Weitlaner retractor (2x3 teeth)	121	1.01	81.19
Pharmaceuticals	Chlorhexidine 2% from 500 ml container (100 ml)	107	0.89	82.08
Reusable components from reusable set (minor op set)	Suture scissor	103	0.86	82.94
Reusable components from reusable set (minor op set)	Crile wood needle holder	97	0.81	83.74
Single-use equipment and medical devices	Incontinence pad	96	0.80	84.54
Single-use personal protective equipment	Sterile gloves (two pairs)	93	0.77	85.31
Single-use patient or instrument table drape	Hand pack packaging (drape component)	91	0.76	86.07
Reusable components from reusable set (minor op set)	Towel clip	89	0.74	86.82
Cleaning products, waste bags	Disinfectant wipe	85	0.70	87.52
Pharmaceuticals	Hyaluronidase 1500 I.U. (10 ml)	78	0.65	88.17
Pharmaceuticals	Sodium Chloride 0.9% from 1 litre bottle (100 ml)	76	0.63	88.80
Single-use personal protective equipment	Surgical face mask	75	0.62	89.42
Reusable components from reusable set (minor op set)	BP scalpel handle	71	0.59	90.02
Reusable components from reusable set (minor op set)	McDonald dissector	69	0.57	90.59
Single-use personal protective equipment	Gloves (non-sterile, pair)	66	0.55	91.14
Reusable components from reusable set (minor op set)	Stevens tenotomy scissor	62	0.51	91.65

Cleaning products, waste bags	Orange waste bag (infectious waste) with cable tie from scrub room	61	0.51	92.16
Reusable components from reusable set (minor op set)	Adson dissecting toothed forceps	59	0.49	92.65
Single-use equipment and medical devices	Nail scrubbing brush	58	0.49	93.14
Non-set reusable equipment	Reusable tourniquet pressure cuff	56	0.46	93.60
Reusable components from reusable set (minor op set)	Mosquito artery forceps (curved)	54	0.45	94.06
Single-use personal protective equipment	Sterile gloves (two pair, latex free)	54	0.45	94.51
Reusable components from reusable set (minor op set)	Kilner cats paw retractor	53	0.44	94.95
Reusable components from reusable set (minor op set)	Bipolar diathermy forceps (small)	53	0.44	95.39
Single-use equipment and medical devices	Undercast padding	52	0.43	95.82
Single-use equipment and medical devices	Stockinette tubular bandage	50	0.42	96.24
Single-use personal protective equipment	Surgical face mask with eye protection	49	0.41	96.65
Pharmaceuticals	Levobupivacaine 5mg/10 ml (10 ml)	48	0.40	97.04
Reusable components from reusable set (minor op set)	Gillies skin hook	41	0.34	97.39
Single-use equipment and medical devices	Skin marker	33	0.27	97.66
Cleaning products, waste bags	Disinfectant sachet	27	0.23	97.89
Single-use equipment and medical devices	Syringe (20 ml)	27	0.22	98.11
Single-use equipment and medical devices	Syringe (20 ml)	26	0.22	98.33
Cleaning products, waste bags	Red bag (linen laundering) with cable tie	26	0.22	98.54
Single-use personal protective equipment	Surgical hat	20	0.17	98.71
Single-use equipment and medical devices	Foam cube	18	0.15	98.86
Single-use equipment and medical devices	Syringe (10 ml)	17	0.14	99.00
Single-use equipment and medical devices	Light cover	15	0.13	99.13
Single-use equipment and medical devices	Gauze (individual piece)	15	0.12	99.25
Single-use equipment and medical devices	Surgical blade	15	0.12	99.37
Single-use equipment and medical devices	Hand packaging (single-use equipment component)	14	0.12	99.49
Cleaning products, waste bags	Clear bin bag (linen laundering)	13	0.10	99.59
Single-use equipment and medical devices	Non-woven dressing	10	0.08	99.68
Single-use components from reusable set (minor op set)	Kit list	10	0.08	99.76
Single-use equipment and medical devices	Elasticated fabric dressing strip	5	0.04	99.80
Single-use equipment and medical devices	Suture (monofilament, non-absorbable 5-0)	4	0.04	99.84
Single-use personal protective equipment	Visor	4	0.04	99.87
Single-use equipment and medical devices	Suture (monofilament, non-absorbable, 4-0)	3	0.02	99.90
Single-use equipment and medical devices	Needle (green)	3	0.02	99.92
Single-use equipment and medical devices	Needle (red)	3	0.02	99.95
Single-use equipment and medical devices	Surgical blade	2	0.02	99.96
Reusable components from reusable set (minor op set)	Metal tray (small)	1	0.01	99.98
Single-use equipment and medical devices	Needle (blue)	1	0.01	99.99
Reusable components from reusable set (minor op set)	Pin mat	1	0.005	99.99
Reusable components from reusable set (minor op set)	Identification tag	1	0.005	100.00
Single-use equipment and medical devices	Tape (clear)	0.50	0.004	100.00

Supplementary Table 24: Mean average carbon footprint of products used for inguinal hernia repair

Mean average carbon footprint of items used across six inguinal hernia repair operations (H1-H6), in order of contribution. Mean is dependent upon number of items used, and number of operations using the item. CO_2e= carbon dioxide equivalents.

Product category	Product	Mean average carbon footprint (g CO_2e)	Percentage of mean average total (%)	Cumulative percentage (%)
Single-use personal protective equipment	Surgical gown (including hand towels)	1,138	9.75	9.75
Reusable patient or instrument table drapes	High fluid drape	732	6.27	16.02
Single-use equipment and medical devices	Monopolar diathermy with smoke evacuation system	675	5.78	21.80
Pharmaceuticals	Topical skin adhesive (0.8 g)	654	5.60	27.40
Single-use patient or instrument table drape	Table drape (instruments)	646	5.53	32.93
Pharmaceuticals	Levobupivacaine 5mg/10 ml (10 ml)	602	5.15	38.08
Reusable components from reusable set (general basic set B)	Container	429	3.67	41.75
Single-use equipment and medical devices	Gauze (sterile pack, 10x7.5 cm)	380	3.25	45.00
Reusable personal protective equipment	Reusable scrubs	290	2.48	47.49
Reusable personal protective equipment	Reusable surgical gown (including hand towels)	269	2.30	49.79
Single-use equipment and medical devices	Needle counter	254	2.18	51.97
Reusable patient or instrument table drapes	Low fluid drape	248	2.13	54.09
Single-use equipment and medical devices	Mesh	246	2.11	56.20
Single-use personal protective equipment	Sterile gloves (pair)	215	1.84	58.04
Single-use components from reusable set (general basic set A)	Tray wrap (outer, 150x180 cm)	209	1.79	59.83
Single-use components from reusable set (general basic set A)	Tray wrap (inner, 150x180 cm)	205	1.76	61.59
Cleaning products, waste bags	Orange waste bag (infectious waste, large) with cable tie	205	1.75	63.34
Cleaning products, waste bags	Mop head	194	1.66	65.00
Single-use personal protective equipment	Sterile gloves (two pairs)	179	1.53	66.53
Single-use equipment and medical devices	Incontinence pad	154	1.32	67.85
Single-use equipment and medical devices	Gauze (sterile pack, 10x10 cm)	142	1.22	69.07
Cleaning products, waste bags	Disinfectant wipe	140	1.20	70.27
Single-use personal protective equipment	Gloves (non-sterile, pair)	135	1.16	71.43
Reusable components from reusable set (general basic set B)	Kidney dish (25 cm) x4	131	1.12	72.55
Single-use equipment and medical devices	Gauze (sterile pack, 30x30 cm)	119	1.02	73.56
Single-use equipment and medical devices	Syringe (20 ml)	104	0.89	74.46
Single-use equipment and medical devices	Diathermy pad	97	0.83	75.29
Cleaning products, waste bags	Orange waste bag (infectious waste, small) with cable tie	91	0.78	76.07
Reusable components from reusable set (general basic set B)	Rampley sponge holder forceps x4	85	0.72	76.79
Reusable components from reusable set (general basic set B)	Schnidt artery forceps x6	78	0.67	77.46
Single-use personal protective equipment	Sterile gloves (one pair, latex free)	76	0.65	78.11
Pharmaceuticals	Chlorhexidine 1% from 500 ml container (100 ml)	72	0.61	78.72
Reusable components from reusable set (general basic set B)	Towel clip x6	70	0.60	79.32
Reusable components from reusable set (general basic set B)	Spencer Wells curved artery forceps x4	69	0.59	79.91
Single-use equipment and medical devices	Surgical suspensory bandage	67	0.57	80.48
Reusable components from reusable set (general basic set A)	Sponge holder forceps x4	63	0.54	81.02
Reusable patient or instrument table drapes	Huck towel	62	0.53	81.55
Pharmaceuticals	Povidone iodine 10% from 500 ml container (60 ml)	61	0.52	82.07

Cleaning products, waste bags	Yellow waste bag (clinical waste) with cable tie	51	0.44	82.51
Reusable components from reusable set (general basic set B)	Spencer Wells straight artery forceps x6	48	0.41	82.92
Single-use components from reusable set (general basic set B)	Gallipot x3	48	0.41	83.33
Reusable components from reusable set (general basic set B)	Treves toothed dissecting forceps x6	46	0.40	83.72
Single-use equipment and medical devices	Specimen pot (40 ml 4% formaldehyde)	44	0.38	84.10
Cleaning products, waste bags	Clear bin bag (recycling) with cable tie	44	0.37	84.48
Reusable components from reusable set (general basic set B)	Basket	41	0.36	84.83
Single-use personal protective equipment	Surgical face mask	41	0.35	85.19
Reusable components from reusable set (general basic set B)	Czerny retractor x2	41	0.35	85.53
Reusable components from reusable set (general basic set B)	Halstead mosquito curved artery forceps x6	40	0.35	85.88
Single-use equipment and medical devices	Shaver head	39	0.34	86.22
Single-use equipment and medical devices	Nail scrubbing brush	39	0.33	86.55
Pharmaceuticals	Sodium Chloride 0.9% from 1 litre bottle (50 ml)	38	0.32	86.88
Single-use personal protective equipment	Sterile under-gloves (pair)	36	0.31	87.18
Reusable components from reusable set (general basic set B)	Diathermy lead	36	0.31	87.49
Reusable components from reusable set (general basic set B)	Travers self retaining retractor	36	0.31	87.80
Single-use equipment and medical devices	Light handle	35	0.30	88.10
Cleaning products, waste bags	Black waste bag (domestic waste) with cable tie	35	0.30	88.39
Reusable components from reusable set (general basic set A)	Towel clip x5	35	0.30	88.69
Reusable components from reusable set (general basic set B)	Langenbeck retractor (medium) x2	34	0.29	88.99
Reusable components from reusable set (general basic set A)	Langenbeck medium retractor x2	34	0.29	89.28
Single-use components from reusable set (general basic set A)	Paper (90x90 cm)	34	0.29	89.56
Cleaning products, waste bags	Red bag (linen laundering) with cable tie	32	0.28	89.84
Reusable components from reusable set (general basic set B)	Diathermy quiver	32	0.27	90.11
Single-use equipment and medical devices	Suture (braided, absorbable, 2-0)	30	0.26	90.37
Reusable components from reusable set (general basic set B)	Lanes tissue forceps x2	28	0.24	90.60
Reusable components from reusable set (general basic set B)	Bipolar diathermy	26	0.23	90.83
Reusable components from reusable set (general basic set A)	Travers self-retaining retractor	26	0.22	91.05
Reusable components from reusable set (general basic set A)	Langenbeck small retractor x2	25	0.22	91.27
Single-use equipment and medical devices	Kidney dish	24	0.21	91.48
Reusable components of individually wrapped instruments (Collingwood Stewart hernia forceps)	Collingwood Stewart Hernia forceps	24	0.21	91.69
Reusable components of individually wrapped instruments (Roberts artery forceps)	Roberts artery forceps	24	0.21	91.89
Cleaning products, waste bags	Clear bin bag (for swab count)	24	0.21	92.10
Single-use equipment and medical devices	Nonwoven dressing (10x20 cm)	24	0.20	92.30
Reusable components from reusable set (general basic set A)	Dunhill artery forceps x5	24	0.20	92.51
Reusable components from reusable set (general basic set A)	Spencer Wells curved artery forceps x2	23	0.20	92.71
Reusable components from reusable set (general basic set B)	Mayo curved scissors	23	0.20	92.90
Single-use equipment and medical devices	Suture (monofilament, nonabsorbable, 2-0)	23	0.20	93.10
Cleaning products, waste bags	Disinfectant sachet	23	0.20	93.30
Reusable components from reusable set (general basic set A)	Diathermy lead	23	0.19	93.49
Reusable components from reusable set (general basic set A)	Czerny retractor x2	22	0.19	93.68
Reusable components from reusable set (general basic set A)	Mosquito fine artery forceps x5	22	0.19	93.87
Reusable components from reusable set (general basic set A)	Morris retractor	22	0.19	94.05
Reusable components from reusable set (general basic set A)	Diathermy quiver	20	0.17	94.23
Reusable components from reusable set (general basic set B)	Bonney toothed dissecting forceps	20	0.17	94.40
Single-use equipment and medical devices	Pre-operative adhesive glove	20	0.17	94.57
Reusable components from reusable set (general basic set A)	Lanes tissue forceps x2	20	0.17	94.74
Single-use patient or instrument table drape	Patient drape (incise drape, with iodine)	19	0.17	94.90

Reusable components from reusable set (general basic set B)	Mayo Hegar needle holder x2	19	0.16	95.07
Reusable components from reusable set (general basic set B)	BP scalpel handle (no. 4) x2	19	0.16	95.23
Single-use components from reusable set (general basic set B)	Bag plain closure (H)	19	0.16	95.39
Reusable components from reusable set (general basic set B)	Allis tissue forceps x2	19	0.16	95.55
Single-use equipment and medical devices	Suture (monofilament, absorbable, 3-0)	18	0.15	95.70
Cleaning products, waste bags	Green bag (linen laundering) with cable tie	17	0.15	95.85
Single-use equipment and medical devices	Nonwoven dressing (10x30 cm)	17	0.14	95.99
Single-use personal protective equipment	Surgical hat	17	0.14	96.14
Reusable components from reusable set (general basic set B)	Bulldog clip	16	0.14	96.28
Reusable components from reusable set (general basic set B)	BP scalpel handle (no. 3) x2	16	0.14	96.42
Reusable components from reusable set (general basic set B)	McIndoe plain diathermy forceps	16	0.14	96.56
Reusable components from reusable set (general basic set B)	Babcock tissue forceps x2	16	0.14	96.69
Reusable components from reusable set (general basic set A)	Mayo curved scissor	15	0.13	96.82
Reusable components from reusable set (general basic set B)	Mayo straight scissors	15	0.13	96.95
Single-use components from reusable set (general basic set A)	Tray lining (60x60 cm)	14	0.12	97.07
Reusable components from reusable set (general basic set A)	Mayo straight scissor	14	0.12	97.19
Reusable components from reusable set (general basic set A)	Allis tissue forceps x2	13	0.11	97.30
Single-use components from reusable set (general basic set A)	Gallipot x2	13	0.11	97.41
Single-use equipment and medical devices	Absorbent towel pack	13	0.11	97.52
Single-use personal protective equipment	Surgical face mask with eye protection	13	0.11	97.63
Reusable components from reusable set (general basic set B)	Plain dissecting forceps (7")	12	0.10	97.73
Reusable components from reusable set (general basic set A)	McIndoe plain diathermy forceps	12	0.10	97.83
Single-use equipment and medical devices	Suture (monofilament, nonabsorbable, 1)	12	0.10	97.93
Reusable components from reusable set (general basic set B)	McIndoe scissors	11	0.10	98.03
Reusable components from reusable set (general basic set A)	BP scalpel handle (no. 3) x2	11	0.09	98.12
Reusable components from reusable set (general basic set A)	Kidney dish (20 cm)	11	0.09	98.22
Reusable components from reusable set (general basic set B)	Stitch scissor	11	0.09	98.31
Reusable components from reusable set (general basic set A)	Debakey plan dissecting forceps x2	11	0.09	98.40
Reusable components from reusable set (general basic set A)	Babcock forceps x2	10	0.09	98.49
Reusable components from reusable set (general basic set A)	Gilles toothed dissecting forceps x2	10	0.09	98.57
Reusable components from reusable set (general basic set B)	Plain dissecting forceps (5")	10	0.08	98.66
Reusable components from reusable set (general basic set A)	Lahey cholecystectomy forceps	10	0.08	98.74
Reusable non-set equipment	Diathermy pad lead	9	0.08	98.82
Single-use components of individually wrapped instruments (Roberts artery forceps)	Flexible pouch	9	0.07	98.89
Single-use components of individually wrapped instruments (Collingwood Stewart hernia forceps)	Flexible pouch	9	0.07	98.97
Single-use components from reusable set (general basic set A)	Pulp tray (no.3)	8	0.07	99.04
Reusable components from reusable set (general basic set A)	Mayo Hegar needle holder	8	0.07	99.11
Single-use equipment and medical devices	Surgical blade (10)	8	0.07	99.18
Reusable components from reusable set (general basic set B)	Gillies toothed dissecting forceps	8	0.07	99.25
Reusable components from reusable set (general basic set A)	Hernia ring	8	0.07	99.31
Reusable components from reusable set (general basic set A)	Crile baby needle holder	8	0.07	99.38
Reusable components from reusable set (general basic set A)	Mcindoe curved scissor	8	0.07	99.45
Reusable components from reusable set (general basic set B)	Debakey dissecting forceps	7	0.06	99.51
Reusable components from reusable set (general basic set B)	McIndoe dissecting forceps	7	0.06	99.56
Single-use components from reusable set (general basic set B)	Kit list	7	0.06	99.62
Reusable components from reusable set (general basic set A)	BP scalpel handle (no. 4)	6	0.05	99.67
Reusable components from reusable set (general basic set A)	Baby Mixter forceps	5	0.05	99.72
Single-use components from reusable set (general basic set B)	Filter paper	5	0.04	99.76

Cleaning products, waste bags	Chlorine tablet	5	0.04	99.80
Single-use components from reusable set (general basic set B)	Tamper proof tags	5	0.04	99.84
Single-use components from reusable set (general basic set A)	Kit list	3	0.03	99.87
Single-use equipment and medical devices	Reinforced skin closure strip	3	0.03	99.89
Single-use equipment and medical devices	Needle (green)	3	0.03	99.92
Single-use equipment and medical devices	Diathermy tip	3	0.02	99.94
Single-use equipment and medical devices	Suture (braided, absorbable, 0)	3	0.02	99.97
Reusable components from reusable set (general basic set A)	Metal tray (medium)	2	0.01	99.98
Reusable personal protective equipment	Reusable surgical hat	1	0.01	99.99
Reusable non-set equipment	Shaver base	0.4	0.003	100.00
Reusable components from reusable set (general basic set B)	Identification tag	0.3	0.002	100.00
Reusable components from reusable set (general basic set A)	Identification tag	0.2	0.002	100.00
Reusable components of individually wrapped instruments (Roberts artery forceps)	Identification tag	0.1	0.001	100.00
Reusable components of individually wrapped instruments (Collingwood Stewart hernia forceps)	Identification tag	0.1	0.001	100.00

Supplementary Table 25: Mean average carbon footprint of products used for knee arthroplasty
Mean average carbon footprint of items used across ten knee arthroplasty operations (K1-K10), in order of contribution. Mean is dependent upon number of items used, and number of operations using the item. CO_2e= carbon dioxide equivalents.

Product category	Product	Mean average carbon footprint (g CO_2e)	Percentage of mean average total (%)	Cumulative percentage (%)
Pharmaceuticals	Bone cement mix (with tobramycin)	18,499	21.65	21.65
Pharmaceuticals	Bone cement mix (with gentamicin)	5,281	6.18	27.83
Single-use equipment and medical devices	Pulsed lavage system	3,727	4.36	32.19
Single-use equipment and medical devices	Posterior stabilised femoral implant	3,528	4.13	36.32
Single-use patient or instrument table drape	Table drape (instruments)	3,009	3.52	39.84
Single-use equipment and medical devices	Primary tibial baseplate implant	2,997	3.51	43.35
Single-use equipment and medical devices	Cruciate retaining femoral implant	2,759	3.23	46.57
Single-use personal protective equipment	Surgical gown (including hand towels)	1,826	2.14	48.71
Single-use patient or instrument table drape	Patient drape (extremity, 230x325 cm)	1,636	1.91	50.63
Single-use patient or instrument table drape	Table drape (140x90 cm)	1,406	1.64	52.27
Single-use personal protective equipment	Orthopaedic hood	1,345	1.57	53.84
Single-use equipment and medical devices	Cement mixing and delivery system	1,315	1.54	55.38
Single-use patient or instrument table drape	Patient drape (pouch fluid collection, 40x35 cm)	1,287	1.51	56.89
Single-use patient or instrument table drape	Patient drape (240x150 cm)	1,163	1.36	58.25
Single-use personal protective equipment	Gown	1,089	1.27	59.53
Single-use patient or instrument table drape	Table drape (fan folded, 140x190 cm)	1,038	1.21	60.74
Single-use equipment and medical devices	Tourniquet pressure cuff (leg)	980	1.15	61.89
Pharmaceuticals	Sodium chloride 0.9% for irrigation (3 L bag)	918	1.07	62.96
Single-use patient or instrument table drape	Patient drape (Mayo cover, 75x144 cm) x2	842	0.99	63.95
Single-use equipment and medical devices	Tibial bearing insert cruciate retaining implant	710	0.83	64.78
Single-use personal protective equipment	Sterile gloves (pair)	670	0.78	65.56
Reusable components from reusable set (basic major orthopaedic set)	Container	644	0.75	66.32
Single-use patient or instrument table drape	Patient drape (impervious, split, 152x177 cm)	644	0.75	67.07
Reusable components from reusable set (miscellaneous knee system set)	Container	644	0.75	67.82
Reusable components from reusable set (femoral and tibial preparation set (size 3-6))	Container	644	0.75	68.58
Reusable components from reusable set (orthopaedic surgical drill)	Container	644	0.75	69.33
Pharmaceuticals	Ropivacaine 75mg in 10 ml (10 ml)	623	0.73	70.06
Cleaning products, waste bags	Orange waste bag (infectious waste) with cable tie from theatre	615	0.72	70.78
Single-use equipment and medical devices	Tray (small)	589	0.69	71.47
Single-use patient or instrument table drape	Patient drape (stockinette, impervious, 30x120 cm)	584	0.68	72.15
Reusable components from reusable set (cruciate retaining femoral and tibial trialing set (size 3-6))	Container	579	0.68	72.83
Single-use equipment and medical devices	Crepe bandage x2	550	0.64	73.47
Single-use personal protective equipment	Sterile gloves (two pairs)	501	0.59	74.06
Cleaning products, waste bags	Disinfectant wipe	486	0.57	74.63
Reusable components from reusable set (patella preparation and trialing set)	Container	451	0.53	75.16
Single-use equipment and medical devices	Tibial bearing insert posterior stabilised implant	444	0.52	75.67
Single-use equipment and medical devices	Suction tubing	437	0.51	76.19
Reusable personal protective equipment	Reusable scrubs	433	0.51	76.69

Pharmaceuticals	Chlorhexidine gluconate in 70% denatured ethanol with 4 ml red stain solution from 200 ml bottle (150 ml)	393	0.46	77.15
Pharmaceuticals	Topical skin adhesive (0.8 g)	392	0.46	77.61
Single-use equipment and medical devices	Symmetric patella implant	359	0.42	78.03
Single-use patient or instrument table drape	Knee pack packaging (patient and instrument table drape component)	343	0.40	78.43
Single-use equipment and medical devices	Swab tray (large)	322	0.38	78.81
Single-use equipment and medical devices	Syringe (50 ml)	301	0.35	79.16
Reusable components from reusable set (basic major orthopaedic set)	Heath mallet	283	0.33	79.49
Single-use equipment and medical devices	Incontinence pad	261	0.31	79.80
Cleaning products, waste bags	Mop head	253	0.30	80.10
Single-use equipment and medical devices	Monopolar diathermy	240	0.28	80.38
Single-use patient or instrument table drape	Patient drape (90 x75 cm)	229	0.27	80.64
Reusable components from reusable set (basic major orthopaedic set)	Cement gun	225	0.26	80.91
Single-use patient or instrument table drape	Patient drape (clear U drape)	220	0.26	81.17
Single-use equipment and medical devices	Needle counter	214	0.25	81.42
Reusable components from reusable set (patella preparation and trialing set)	Instruments (top tray, 5 items) item 1	211	0.25	81.66
Reusable components from reusable set (patella preparation and trialing set)	Instruments (top tray, 5 items) item 2	211	0.25	81.91
Reusable components from reusable set (patella preparation and trialing set)	Instruments (top tray, 5 items) item 3	211	0.25	82.16
Reusable components from reusable set (patella preparation and trialing set)	Instruments (top tray, 5 items) item 4	211	0.25	82.40
Reusable components from reusable set (patella preparation and trialing set)	Instruments (top tray, 5 items) item 5	211	0.25	82.65
Pharmaceuticals	Ketorolac tromethamine 30mg in 1 ml (1 ml)	210	0.25	82.90
Cleaning products, waste bags	Anatomical waste bin	208	0.24	83.14
Single-use equipment and medical devices	Saw blade	205	0.24	83.38
Single-use equipment and medical devices	Cement mixing bowl	204	0.24	83.62
Single-use patient or instrument table drape	Patient drape (adhesive split sheet)	198	0.23	83.85
Reusable components from reusable set (posterior stabilised femoral and tibial trialing set (size 3-6))	Container	193	0.23	84.08
Single-use equipment and medical devices	Suction receptacle	192	0.23	84.30
Pharmaceuticals	Levobupivacaine 2.5mg/ml (10 ml)	191	0.22	84.53
Single-use equipment and medical devices	Cast padding bandage x2	188	0.22	84.75
Single-use equipment and medical devices	Skin stapler	184	0.22	84.96
Reusable components of individually wrapped instruments (light handle)	Light cover	161	0.19	85.15
Single-use personal protective equipment	Gloves (non-sterile, pair)	157	0.18	85.33
Single-use equipment and medical devices	High-vacuum wound drainage	149	0.17	85.51
Reusable components of individually wrapped instruments (light cover)	Light cover	147	0.17	85.68
Single-use equipment and medical devices	Knee pack packaging (equipment component)	145	0.17	85.85
Single-use patient or instrument table drape	Patient drape (incise drape, with iodine)	144	0.17	86.02
Cleaning products, waste bags	Disinfectant sachet	137	0.16	86.18
Single-use equipment and medical devices	Swab gauze (30x30 cm) x10	135	0.16	86.34
Single-use equipment and medical devices	Tubular support bandage	129	0.15	86.49
Reusable components from reusable set (cruciate retaining femoral and tibial preparation and trialing set (size 1,2,7,8))	Container	129	0.15	86.64
Single-use equipment and medical devices	Swab tray (small)	128	0.15	86.79
Pharmaceuticals	Sodium chloride 0.9% (100 ml)	128	0.15	86.94
Reusable components from reusable set (orthopaedic surgical drill)	Instruments (13 items) item 1	122	0.14	87.08
Reusable components from reusable set (orthopaedic surgical drill)	Instruments (13 items) item 2	122	0.14	87.22
Reusable components from reusable set (orthopaedic surgical drill)	Instruments (13 items) item 3	122	0.14	87.36
Reusable components from reusable set (orthopaedic surgical drill)	Instruments (13 items) item 4	122	0.14	87.51
Reusable components from reusable set (orthopaedic surgical drill)	Instruments (13 items) item 5	122	0.14	87.65
Reusable components from reusable set (orthopaedic surgical drill)	Instruments (13 items) item 6	122	0.14	87.79

Reusable components from reusable set (orthopaedic surgical drill)	Instruments (13 items) item 7	122	0.14	87.93
Reusable components from reusable set (orthopaedic surgical drill)	Instruments (13 items) item 8	122	0.14	88.08
Reusable components from reusable set (orthopaedic surgical drill)	Instruments (13 items) item 9	122	0.14	88.22
Reusable components from reusable set (orthopaedic surgical drill)	Instruments (13 items) item 10	122	0.14	88.36
Reusable components from reusable set (orthopaedic surgical drill)	Instruments (13 items) item 11	122	0.14	88.50
Reusable components from reusable set (orthopaedic surgical drill)	Instruments (13 items) item 12	122	0.14	88.64
Reusable components from reusable set (orthopaedic surgical drill)	Instruments (13 items) item 13	122	0.14	88.79
Reusable components from reusable set (basic major orthopaedic set)	Bone nibbler x2	121	0.14	88.93
Reusable components of individually wrapped instruments (Semb bone holding forceps)	Semb bone holding forceps	121	0.14	89.07
Single-use equipment and medical devices	Kidney dish x3	115	0.13	89.20
Single-use equipment and medical devices	Suction tubing	99	0.12	89.32
Pharmaceuticals	Chlorhexidine 2% from 500 ml bottle (150 ml)	97	0.11	89.43
Reusable components from reusable set (basic major orthopaedic set)	Norfolk and Norwich retractor x2	94	0.11	89.54
Single-use equipment and medical devices	Nail scrubbing brush	94	0.11	89.65
Single-use equipment and medical devices	Intravenous infusion giving set	93	0.11	89.76
Single-use equipment and medical devices	Bowl (500 ml)	92	0.11	89.87
Reusable components from reusable set (femoral and tibial preparation set (size 3-6))	Instruments (bottom tray, 13 items) item 1	86	0.10	89.97
Reusable components from reusable set (femoral and tibial preparation set (size 3-6))	Instruments (bottom tray, 13 items) item 2	86	0.10	90.07
Reusable components from reusable set (femoral and tibial preparation set (size 3-6))	Instruments (bottom tray, 13 items) item 3	86	0.10	90.17
Reusable components from reusable set (femoral and tibial preparation set (size 3-6))	Instruments (bottom tray, 13 items) item 4	86	0.10	90.27
Reusable components from reusable set (femoral and tibial preparation set (size 3-6))	Instruments (bottom tray, 13 items) item 5	86	0.10	90.37
Reusable components from reusable set (femoral and tibial preparation set (size 3-6))	Instruments (bottom tray, 13 items) item 6	86	0.10	90.47
Reusable components from reusable set (femoral and tibial preparation set (size 3-6))	Instruments (bottom tray, 13 items) item 7	86	0.10	90.57
Reusable components from reusable set (femoral and tibial preparation set (size 3-6))	Instruments (bottom tray, 13 items) item 8	86	0.10	90.67
Reusable components from reusable set (femoral and tibial preparation set (size 3-6))	Instruments (bottom tray, 13 items) item 9	86	0.10	90.77
Reusable components from reusable set (femoral and tibial preparation set (size 3-6))	Instruments (bottom tray, 13 items) item 10	86	0.10	90.87
Reusable components from reusable set (femoral and tibial preparation set (size 3-6))	Instruments (bottom tray, 13 items) item 11	86	0.10	90.97
Reusable components from reusable set (femoral and tibial preparation set (size 3-6))	Instruments (bottom tray, 13 items) item 12	86	0.10	91.08
Reusable components from reusable set (femoral and tibial preparation set (size 3-6))	Instruments (bottom tray, 13 items) item 13	86	0.10	91.18
Cleaning products, waste bags	Orange waste bag (infectious waste) with cable tie from scrub room	82	0.10	91.27
Reusable components from reusable set (cruciate retaining femoral and tibial trialing set (size 3-6))	Instruments (top tray, 16 items) item 1	74	0.086	91.36
Reusable components from reusable set (cruciate retaining femoral and tibial trialing set (size 3-6))	Instruments (top tray, 16 items) item 2	74	0.086	91.44
Reusable components from reusable set (cruciate retaining femoral and tibial trialing set (size 3-6))	Instruments (top tray, 16 items) item 3	74	0.086	91.53
Reusable components from reusable set (cruciate retaining femoral and tibial trialing set (size 3-6))	Instruments (top tray, 16 items) item 4	74	0.086	91.62
Reusable components from reusable set (cruciate retaining femoral and tibial trialing set (size 3-6))	Instruments (top tray, 16 items) item 5	74	0.086	91.70
Reusable components from reusable set (cruciate retaining femoral and tibial trialing set (size 3-6))	Instruments (top tray, 16 items) item 6	74	0.086	91.79
Reusable components from reusable set (cruciate retaining femoral and tibial trialing set (size 3-6))	Instruments (top tray, 16 items) item 7	74	0.086	91.87
Reusable components from reusable set (cruciate retaining femoral and tibial trialing set (size 3-6))	Instruments (top tray, 16 items) item 8	74	0.086	91.96
Reusable components from reusable set (cruciate retaining femoral and tibial trialing set (size 3-6))	Instruments (top tray, 16 items) item 9	74	0.086	92.05
Reusable components from reusable set (cruciate retaining femoral and tibial trialing set (size 3-6))	Instruments (top tray, 16 items) item 10	74	0.086	92.13
Reusable components from reusable set (cruciate retaining femoral and tibial trialing set (size 3-6))	Instruments (top tray, 16 items) item 11	74	0.086	92.22
Reusable components from reusable set (cruciate retaining femoral and tibial trialing set (size 3-6))	Instruments (top tray, 16 items) item 12	74	0.086	92.31
Reusable components from reusable set (cruciate retaining femoral and tibial trialing set (size 3-6))	Instruments (top tray, 16 items) item 13	74	0.086	92.39
Reusable components from reusable set (cruciate retaining femoral and tibial trialing set (size 3-6))	Instruments (top tray, 16 items) item 14	74	0.086	92.48
Reusable components from reusable set (cruciate retaining femoral and tibial trialing set (size 3-6))	Instruments (top tray, 16 items) item 15	74	0.086	92.56
Reusable components from reusable set (cruciate retaining femoral and tibial trialing set (size 3-6))	Instruments (top tray, 16 items) item 16	74	0.086	92.65
Reusable components of individually wrapped instruments (Lanes tissue forceps)	Lanes tissue forceps	74	0.086	92.74

Single-use personal protective equipment	Surgical hat	71	0.083	92.82
Single-use equipment and medical devices	Diathermy tip cleaner	70	0.082	92.90
Single-use equipment and medical devices	Bowl (250 ml) x2	67	0.078	92.98
Reusable components from reusable set (basic major orthopaedic set)	McGoey punch	67	0.078	93.06
Single-use equipment and medical devices	Yankauer sucker	66	0.077	93.13
Cleaning products, waste bags	Clear waste bag (recycling) with cable tie	66	0.077	93.21
Reusable components from reusable set (miscellaneous knee system set)	Instruments (top tray, 13 items) item 1	65	0.076	93.29
Reusable components from reusable set (miscellaneous knee system set)	Instruments (top tray, 13 items) item 2	65	0.076	93.36
Reusable components from reusable set (miscellaneous knee system set)	Instruments (top tray, 13 items) item 3	65	0.076	93.44
Reusable components from reusable set (miscellaneous knee system set)	Instruments (top tray, 13 items) item 4	65	0.076	93.51
Reusable components from reusable set (miscellaneous knee system set)	Instruments (top tray, 13 items) item 5	65	0.076	93.59
Reusable components from reusable set (miscellaneous knee system set)	Instruments (top tray, 13 items) item 6	65	0.076	93.66
Reusable components from reusable set (miscellaneous knee system set)	Instruments (top tray, 13 items) item 7	65	0.076	93.74
Reusable components from reusable set (miscellaneous knee system set)	Instruments (top tray, 13 items) item 8	65	0.076	93.81
Reusable components from reusable set (miscellaneous knee system set)	Instruments (top tray, 13 items) item 9	65	0.076	93.89
Reusable components from reusable set (miscellaneous knee system set)	Instruments (top tray, 13 items) item 10	65	0.076	93.97
Reusable components from reusable set (miscellaneous knee system set)	Instruments (top tray, 13 items) item 11	65	0.076	94.04
Reusable components from reusable set (miscellaneous knee system set)	Instruments (top tray, 13 items) item 12	65	0.076	94.12
Reusable components from reusable set (miscellaneous knee system set)	Instruments (top tray, 13 items) item 13	65	0.076	94.19
Reusable components from reusable set (knee navigation set)	Container	64	0.075	94.27
Single-use equipment and medical devices	Diathermy bag	62	0.073	94.34
Single-use equipment and medical devices	Border dressing (10x30 cm)	62	0.073	94.41
Single-use equipment and medical devices	Crepe bandage (15 cm wide)	61	0.071	94.48
Reusable components from reusable set (miscellaneous knee system set)	Instruments (bottom tray, 12 items) item 1	58	0.068	94.55
Reusable components from reusable set (miscellaneous knee system set)	Instruments (bottom tray, 12 items) item 2	58	0.068	94.62
Reusable components from reusable set (miscellaneous knee system set)	Instruments (bottom tray, 12 items) item 3	58	0.068	94.69
Reusable components from reusable set (miscellaneous knee system set)	Instruments (bottom tray, 12 items) item 4	58	0.068	94.76
Reusable components from reusable set (miscellaneous knee system set)	Instruments (bottom tray, 12 items) item 5	58	0.068	94.83
Reusable components from reusable set (miscellaneous knee system set)	Instruments (bottom tray, 12 items) item 6	58	0.068	94.89
Reusable components from reusable set (miscellaneous knee system set)	Instruments (bottom tray, 12 items) item 7	58	0.068	94.96
Reusable components from reusable set (miscellaneous knee system set)	Instruments (bottom tray, 12 items) item 8	58	0.068	95.03
Reusable components from reusable set (miscellaneous knee system set)	Instruments (bottom tray, 12 items) item 9	58	0.068	95.10
Reusable components from reusable set (miscellaneous knee system set)	Instruments (bottom tray, 12 items) item 10	58	0.068	95.17
Reusable components from reusable set (miscellaneous knee system set)	Instruments (bottom tray, 12 items) item 11	58	0.068	95.24
Reusable components from reusable set (miscellaneous knee system set)	Instruments (bottom tray, 12 items) item 12	58	0.068	95.30
Single-use patient or instrument table drape	Patient drape (incise drape)	58	0.068	95.37
Reusable components from reusable set (basic major orthopaedic set)	Langenbeck retractor (large) x2	57	0.066	95.44
Reusable components from reusable set (basic major orthopaedic set)	Sponge holder x3	56	0.066	95.50
Single-use components of individually wrapped instruments (light handle)	Flexible pouch	56	0.066	95.57
Single-use personal protective equipment	Knee pack packaging (gown component)	56	0.065	95.64
Single-use personal protective equipment	Sterile gloves (two pair, latex free)	54	0.063	95.70
Single-use equipment and medical devices	Syringe (20 ml)	52	0.061	95.76
Single-use components of individually wrapped instruments (light cover)	Flexible pouch	51	0.060	95.82
Reusable components from reusable set (basic major orthopaedic set)	Basket	51	0.059	95.88
Single-use personal protective equipment	Surgical face mask	51	0.059	95.94
Single-use equipment and medical devices	Suture (braided, absorbable 1-0, 803)	49	0.057	96.00
Reusable components from reusable set (basic major orthopaedic set)	Hohmann lever bone x2	49	0.057	96.05
Reusable components from reusable set (basic major orthopaedic set)	Kocher artery forceps (7") x4	47	0.054	96.11

Pharmaceuticals	Iodinated povidone 10% w/w alcoholic tincture from 500 ml bottle (50 ml)	46	0.054	96.16
Single-use personal protective equipment	Surgical face mask with eye protection	45	0.053	96.21
Single-use equipment and medical devices	Towel dressing (2 in pack)	44	0.051	96.27
Cleaning products, waste bags	Red bag (linen laundering) with cable tie	43	0.050	96.32
Reusable components from reusable set (basic major orthopaedic set)	Curette	42	0.049	96.37
Reusable components from reusable set (basic major orthopaedic set)	Langenbeck retractor (medium) x2	40	0.047	96.41
Single-use personal protective equipment	Sweat bands for hood x3	39	0.046	96.46
Reusable components from reusable set (basic major orthopaedic set)	Capener gouge (large)	38	0.045	96.50
Reusable components from reusable set (basic major orthopaedic set)	Capener gouge (small)	38	0.045	96.55
Single-use equipment and medical devices	Self-adherent bandage	34	0.040	96.59
Reusable components from reusable set (basic major orthopaedic set)	Osteotome (8 mm)	33	0.039	96.63
Single-use equipment and medical devices	Swab gauze (10x7.5 cm) x5	32	0.038	96.66
Reusable components from reusable set (basic major orthopaedic set)	Osteotome (25 mm)	31	0.037	96.70
Single-use equipment and medical devices	Adhesive operative towel	31	0.036	96.74
Reusable components of individually wrapped instruments (non-toothed lamina spreader (large))	Non-toothed lamina spreader (large)	31	0.036	96.77
Single-use equipment and medical devices	Diathermy bag	30	0.035	96.81
Reusable components of individually wrapped instruments (blunt Hohman bone elevator)	Blunt Hohman bone elevator	30	0.035	96.84
Single-use components of individually wrapped instruments (Semb bone holding forceps)	Flexible pouch	29	0.034	96.88
Pharmaceuticals	Adrenaline 1mg in 1 ml (1 ml)	29	0.034	96.91
Single-use equipment and medical devices	Batteries for knee navigation set (x3)	29	0.033	96.94
Reusable components from reusable set (basic major orthopaedic set)	Bristow elevator	27	0.031	96.98
Reusable components from reusable set (posterior stabilised femoral and tibial trialing set (size 3-6))	Instruments (top tray, 16 items) item 1	26	0.030	97.01
Reusable components from reusable set (posterior stabilised femoral and tibial trialing set (size 3-6))	Instruments (top tray, 16 items) item 2	26	0.030	97.04
Reusable components from reusable set (posterior stabilised femoral and tibial trialing set (size 3-6))	Instruments (top tray, 16 items) item 3	26	0.030	97.07
Reusable components from reusable set (posterior stabilised femoral and tibial trialing set (size 3-6))	Instruments (top tray, 16 items) item 4	26	0.030	97.10
Reusable components from reusable set (posterior stabilised femoral and tibial trialing set (size 3-6))	Instruments (top tray, 16 items) item 5	26	0.030	97.13
Reusable components from reusable set (posterior stabilised femoral and tibial trialing set (size 3-6))	Instruments (top tray, 16 items) item 6	26	0.030	97.16
Reusable components from reusable set (posterior stabilised femoral and tibial trialing set (size 3-6))	Instruments (top tray, 16 items) item 7	26	0.030	97.19
Reusable components from reusable set (posterior stabilised femoral and tibial trialing set (size 3-6))	Instruments (top tray, 16 items) item 8	26	0.030	97.22
Reusable components from reusable set (posterior stabilised femoral and tibial trialing set (size 3-6))	Instruments (top tray, 16 items) item 9	26	0.030	97.25
Reusable components from reusable set (posterior stabilised femoral and tibial trialing set (size 3-6))	Instruments (top tray, 16 items) item 10	26	0.030	97.28
Reusable components from reusable set (posterior stabilised femoral and tibial trialing set (size 3-6))	Instruments (top tray, 16 items) item 11	26	0.030	97.31
Reusable components from reusable set (posterior stabilised femoral and tibial trialing set (size 3-6))	Instruments (top tray, 16 items) item 12	26	0.030	97.34
Reusable components from reusable set (posterior stabilised femoral and tibial trialing set (size 3-6))	Instruments (top tray, 16 items) item 13	26	0.030	97.37
Reusable components from reusable set (posterior stabilised femoral and tibial trialing set (size 3-6))	Instruments (top tray, 16 items) item 14	26	0.030	97.40
Reusable components from reusable set (posterior stabilised femoral and tibial trialing set (size 3-6))	Instruments (top tray, 16 items) item 15	26	0.030	97.43
Reusable components from reusable set (posterior stabilised femoral and tibial trialing set (size 3-6))	Instruments (top tray, 16 items) item 16	26	0.030	97.46
Single-use components of individually wrapped instruments (Lanes tissue forceps)	Flexible pouch	26	0.030	97.49
Reusable components from reusable set (femoral and tibial preparation set (size 3-6))	Instruments (top tray, 17 items) item 1	25	0.030	97.52
Reusable components from reusable set (femoral and tibial preparation set (size 3-6))	Instruments (top tray, 17 items) item 2	25	0.030	97.55
Reusable components from reusable set (femoral and tibial preparation set (size 3-6))	Instruments (top tray, 17 items) item 3	25	0.030	97.58
Reusable components from reusable set (femoral and tibial preparation set (size 3-6))	Instruments (top tray, 17 items) item 4	25	0.030	97.61
Reusable components from reusable set (femoral and tibial preparation set (size 3-6))	Instruments (top tray, 17 items) item 5	25	0.030	97.64
Reusable components from reusable set (femoral and tibial preparation set (size 3-6))	Instruments (top tray, 17 items) item 6	25	0.030	97.67
Reusable components from reusable set (femoral and tibial preparation set (size 3-6))	Instruments (top tray, 17 items) item 7	25	0.030	97.70
Reusable components from reusable set (femoral and tibial preparation set (size 3-6))	Instruments (top tray, 17 items) item 8	25	0.030	97.72
Reusable components from reusable set (femoral and tibial preparation set (size 3-6))	Instruments (top tray, 17 items) item 9	25	0.030	97.75

Reusable components from reusable set (femoral and tibial preparation set (size 3-6))	Instruments (top tray, 17 items) item 10	25	0.030	97.78
Reusable components from reusable set (femoral and tibial preparation set (size 3-6))	Instruments (top tray, 17 items) item 11	25	0.030	97.81
Reusable components from reusable set (femoral and tibial preparation set (size 3-6))	Instruments (top tray, 17 items) item 12	25	0.030	97.84
Reusable components from reusable set (femoral and tibial preparation set (size 3-6))	Instruments (top tray, 17 items) item 13	25	0.030	97.87
Reusable components from reusable set (femoral and tibial preparation set (size 3-6))	Instruments (top tray, 17 items) item 14	25	0.030	97.90
Reusable components from reusable set (femoral and tibial preparation set (size 3-6))	Instruments (top tray, 17 items) item 15	25	0.030	97.93
Reusable components from reusable set (femoral and tibial preparation set (size 3-6))	Instruments (top tray, 17 items) item 16	25	0.030	97.96
Reusable components from reusable set (femoral and tibial preparation set (size 3-6))	Instruments (top tray, 17 items) item 17	25	0.030	97.99
Reusable components from reusable set (basic major orthopaedic set)	Spencer Wells artery forceps (curved, 7") x2	25	0.029	98.02
Reusable components from reusable set (basic major orthopaedic set)	Mayo needle holder x 2	24	0.028	98.05
Reusable components from reusable set (basic major orthopaedic set)	Universal orange scissor	24	0.028	98.08
Reusable components from reusable set (basic major orthopaedic set)	Quiver	24	0.028	98.10
Single-use equipment and medical devices	Skin marker pen	22	0.026	98.13
Reusable components from reusable set (basic major orthopaedic set)	Towel clip x3	22	0.025	98.16
Cleaning products, waste bags	Clear bin bag (linen laundering)	21	0.024	98.18
Single-use equipment and medical devices	Gauze (sterile pack)	20	0.024	98.20
Reusable components from reusable set (basic major orthopaedic set)	Trethowan bone lever x2	20	0.023	98.23
Reusable components from reusable set (femoral and tibial preparation set (size 3-6))	Tray	20	0.023	98.25
Single-use equipment and medical devices	Suture (braided, absorbable, 2-0)	18	0.021	98.27
Reusable components from reusable set (cruciate retaining femoral and tibial trialing set (size 3-6))	Instruments (bottom tray, 12 items) item 1	18	0.021	98.29
Reusable components from reusable set (cruciate retaining femoral and tibial trialing set (size 3-6))	Instruments (bottom tray, 12 items) item 2	18	0.021	98.31
Reusable components from reusable set (cruciate retaining femoral and tibial trialing set (size 3-6))	Instruments (bottom tray, 12 items) item 3	18	0.021	98.33
Reusable components from reusable set (cruciate retaining femoral and tibial trialing set (size 3-6))	Instruments (bottom tray, 12 items) item 4	18	0.021	98.35
Reusable components from reusable set (cruciate retaining femoral and tibial trialing set (size 3-6))	Instruments (bottom tray, 12 items) item 5	18	0.021	98.37
Reusable components from reusable set (cruciate retaining femoral and tibial trialing set (size 3-6))	Instruments (bottom tray, 12 items) item 6	18	0.021	98.40
Reusable components from reusable set (cruciate retaining femoral and tibial trialing set (size 3-6))	Instruments (bottom tray, 12 items) item 7	18	0.021	98.42
Reusable components from reusable set (cruciate retaining femoral and tibial trialing set (size 3-6))	Instruments (bottom tray, 12 items) item 8	18	0.021	98.44
Reusable components from reusable set (cruciate retaining femoral and tibial trialing set (size 3-6))	Instruments (bottom tray, 12 items) item 9	18	0.021	98.46
Reusable components from reusable set (cruciate retaining femoral and tibial trialing set (size 3-6))	Instruments (bottom tray, 12 items) item 10	18	0.021	98.48
Reusable components from reusable set (cruciate retaining femoral and tibial trialing set (size 3-6))	Instruments (bottom tray, 12 items) item 11	18	0.021	98.50
Reusable components from reusable set (cruciate retaining femoral and tibial trialing set (size 3-6))	Instruments (bottom tray, 12 items) item 12	18	0.021	98.52
Reusable components from reusable set (basic major orthopaedic set)	Bone hook	16	0.019	98.54
Reusable components from reusable set (basic major orthopaedic set)	Mayo scissor (curved, 6")	16	0.018	98.56
Reusable components of individually wrapped instruments (bipolar diathermy)	Bipolar diathermy forceps	15	0.018	98.57
Reusable components from reusable set (basic major orthopaedic set)	Spencer Wells artery forceps(straight, 5") x2	15	0.018	98.59
Reusable components of individually wrapped instruments (diathermy lead)	Diathermy lead	15	0.018	98.61
Reusable components from reusable set (miscellaneous knee system set)	Tray	15	0.018	98.63
Reusable components of individually wrapped instruments (non-toothed lamina spreader (small))	Non-toothed lamina spreader (small)	15	0.017	98.65
Single-use equipment and medical devices	Gauze (individual piece)	15	0.017	98.66
Single-use equipment and medical devices	Monopolar diathermy	15	0.017	98.68
Reusable components from reusable set (basic major orthopaedic set)	Scalpel handle (no. 4) x2	15	0.017	98.70
Reusable components from reusable set (cruciate retaining femoral and tibial trialing set (size 3-6))	Tray	15	0.017	98.71
Reusable components from reusable set (basic major orthopaedic set)	Scalpel handle (no. 4L)	14	0.017	98.73
Single-use equipment and medical devices	Light cover	14	0.017	98.75
Single-use components of individually wrapped instruments (non-toothed lamina spreader (large))	Flexible pouch	14	0.016	98.76
Reusable components from reusable set (patella preparation and trialing set)	Tray	14	0.016	98.78
Reusable components from reusable set (basic major orthopaedic set)	BP scalpel handle (no. 3) x2	13	0.015	98.80
Reusable components from reusable set (basic major orthopaedic set)	Mayo scissor (straight, 6")	12	0.014	98.81

Reusable components from reusable set (basic major orthopaedic set)	Curette (double ended)	12	0.014	98.82
Reusable components from reusable set (basic major orthopaedic set)	Bonney toothed dissecting forceps	12	0.013	98.84
Reusable components from reusable set (basic major orthopaedic set)	BP scalpel handle (no. 3L)	11	0.013	98.85
Single-use equipment and medical devices	Diathermy tip cleaner	11	0.013	98.86
Reusable components from reusable set (basic major orthopaedic set)	McIndoe scissor	10	0.012	98.87
Single-use components of individually wrapped instruments (blunt Hohman bone elevator)	Flexible pouch	10	0.012	98.89
Single-use equipment and medical devices	Suture (monofilament, absorbable 3-0, 696)	10	0.012	98.90
Single-use components from reusable set (basic major orthopaedic set)	Kit list	10	0.012	98.91
Single-use components from reusable set (miscellaneous knee system set)	Kit list	10	0.012	98.92
Single-use components from reusable set (femoral and tibial preparation set (size 3-6))	Kit list	10	0.012	98.93
Single-use components from reusable set (orthopaedic surgical drill)	Kit list	10	0.012	98.94
Reusable components from reusable set (knee navigation set)	Instruments (bottom tray, 16 items) item 1	10	0.011	98.96
Reusable components from reusable set (knee navigation set)	Instruments (bottom tray, 16 items) item 2	10	0.011	98.97
Reusable components from reusable set (knee navigation set)	Instruments (bottom tray, 16 items) item 3	10	0.011	98.98
Reusable components from reusable set (knee navigation set)	Instruments (bottom tray, 16 items) item 4	10	0.011	98.99
Reusable components from reusable set (knee navigation set)	Instruments (bottom tray, 16 items) item 5	10	0.011	99.00
Reusable components from reusable set (knee navigation set)	Instruments (bottom tray, 16 items) item 6	10	0.011	99.01
Reusable components from reusable set (knee navigation set)	Instruments (bottom tray, 16 items) item 7	10	0.011	99.02
Reusable components from reusable set (knee navigation set)	Instruments (bottom tray, 16 items) item 8	10	0.011	99.04
Reusable components from reusable set (knee navigation set)	Instruments (bottom tray, 16 items) item 9	10	0.011	99.05
Reusable components from reusable set (knee navigation set)	Instruments (bottom tray, 16 items) item 10	10	0.011	99.06
Reusable components from reusable set (knee navigation set)	Instruments (bottom tray, 16 items) item 11	10	0.011	99.07
Reusable components from reusable set (knee navigation set)	Instruments (bottom tray, 16 items) item 12	10	0.011	99.08
Reusable components from reusable set (knee navigation set)	Instruments (bottom tray, 16 items) item 13	10	0.011	99.09
Reusable components from reusable set (knee navigation set)	Instruments (bottom tray, 16 items) item 14	10	0.011	99.10
Reusable components from reusable set (knee navigation set)	Instruments (bottom tray, 16 items) item 15	10	0.011	99.12
Reusable components from reusable set (knee navigation set)	Instruments (bottom tray, 16 items) item 16	10	0.011	99.13
Single-use equipment and medical devices	Shaver head	9	0.011	99.14
Reusable components from reusable set (cruciate retaining femoral and tibial preparation and trialing set (size 1,2,7,8))	Instruments (top tray, 19 items) item 1	9	0.011	99.15
Reusable components from reusable set (cruciate retaining femoral and tibial preparation and trialing set (size 1,2,7,8))	Instruments (top tray, 19 items) item 2	9	0.011	99.16
Reusable components from reusable set (cruciate retaining femoral and tibial preparation and trialing set (size 1,2,7,8))	Instruments (top tray, 19 items) item 3	9	0.011	99.17
Reusable components from reusable set (cruciate retaining femoral and tibial preparation and trialing set (size 1,2,7,8))	Instruments (top tray, 19 items) item 4	9	0.011	99.18
Reusable components from reusable set (cruciate retaining femoral and tibial preparation and trialing set (size 1,2,7,8))	Instruments (top tray, 19 items) item 5	9	0.011	99.19
Reusable components from reusable set (cruciate retaining femoral and tibial preparation and trialing set (size 1,2,7,8))	Instruments (top tray, 19 items) item 6	9	0.011	99.20
Reusable components from reusable set (cruciate retaining femoral and tibial preparation and trialing set (size 1,2,7,8))	Instruments (top tray, 19 items) item 7	9	0.011	99.21
Reusable components from reusable set (cruciate retaining femoral and tibial preparation and trialing set (size 1,2,7,8))	Instruments (top tray, 19 items) item 8	9	0.011	99.22
Reusable components from reusable set (cruciate retaining femoral and tibial preparation and trialing set (size 1,2,7,8))	Instruments (top tray, 19 items) item 9	9	0.011	99.23
Reusable components from reusable set (cruciate retaining femoral and tibial preparation and trialing set (size 1,2,7,8))	Instruments (top tray, 19 items) item 10	9	0.011	99.24
Reusable components from reusable set (cruciate retaining femoral and tibial preparation and trialing set (size 1,2,7,8))	Instruments (top tray, 19 items) item 11	9	0.011	99.25
Reusable components from reusable set (cruciate retaining femoral and tibial preparation and trialing set (size 1,2,7,8))	Instruments (top tray, 19 items) item 12	9	0.011	99.27
Reusable components from reusable set (cruciate retaining femoral and tibial preparation and trialing set (size 1,2,7,8))	Instruments (top tray, 19 items) item 13	9	0.011	99.28
Reusable components from reusable set (cruciate retaining femoral and tibial preparation and trialing set (size 1,2,7,8))	Instruments (top tray, 19 items) item 14	9	0.011	99.29
Reusable components from reusable set (cruciate retaining femoral and tibial preparation and trialing set (size 1,2,7,8))	Instruments (top tray, 19 items) item 15	9	0.011	99.30
Reusable components from reusable set (cruciate retaining femoral and tibial preparation and trialing set (size 1,2,7,8))	Instruments (top tray, 19 items) item 16	9	0.011	99.31
Reusable components from reusable set (cruciate retaining femoral and tibial preparation and trialing set (size 1,2,7,8))	Instruments (top tray, 19 items) item 17	9	0.011	99.32
Reusable components from reusable set (cruciate retaining femoral and tibial preparation and trialing set (size 1,2,7,8))	Instruments (top tray, 19 items) item 18	9	0.011	99.33
Reusable components from reusable set (cruciate retaining femoral and tibial preparation and trialing set (size 1,2,7,8))	Instruments (top tray, 19 items) item 19	9	0.011	99.34
Reusable components from reusable set (cruciate retaining femoral and tibial preparation and trialing set (size 1,2,7,8))	Instruments (bottom tray, 18 items) item 1	9	0.011	99.35

Reusable components from reusable set (cruciate retaining femoral and tibial preparation and trialing set (size 1,2,7,8))	Instruments (bottom tray, 18 items) item 2	9	0.011	99.36
Reusable components from reusable set (cruciate retaining femoral and tibial preparation and trialing set (size 1,2,7,8))	Instruments (bottom tray, 18 items) item 3	9	0.011	99.37
Reusable components from reusable set (cruciate retaining femoral and tibial preparation and trialing set (size 1,2,7,8))	Instruments (bottom tray, 18 items) item 4	9	0.011	99.38
Reusable components from reusable set (cruciate retaining femoral and tibial preparation and trialing set (size 1,2,7,8))	Instruments (bottom tray, 18 items) item 5	9	0.011	99.39
Reusable components from reusable set (cruciate retaining femoral and tibial preparation and trialing set (size 1,2,7,8))	Instruments (bottom tray, 18 items) item 6	9	0.011	99.40
Reusable components from reusable set (cruciate retaining femoral and tibial preparation and trialing set (size 1,2,7,8))	Instruments (bottom tray, 18 items) item 7	9	0.011	99.41
Reusable components from reusable set (cruciate retaining femoral and tibial preparation and trialing set (size 1,2,7,8))	Instruments (bottom tray, 18 items) item 8	9	0.011	99.42
Reusable components from reusable set (cruciate retaining femoral and tibial preparation and trialing set (size 1,2,7,8))	Instruments (bottom tray, 18 items) item 9	9	0.011	99.44
Reusable components from reusable set (cruciate retaining femoral and tibial preparation and trialing set (size 1,2,7,8))	Instruments (bottom tray, 18 items) item 10	9	0.011	99.45
Reusable components from reusable set (cruciate retaining femoral and tibial preparation and trialing set (size 1,2,7,8))	Instruments (bottom tray, 18 items) item 11	9	0.011	99.46
Reusable components from reusable set (cruciate retaining femoral and tibial preparation and trialing set (size 1,2,7,8))	Instruments (bottom tray, 18 items) item 12	9	0.011	99.47
Reusable components from reusable set (cruciate retaining femoral and tibial preparation and trialing set (size 1,2,7,8))	Instruments (bottom tray, 18 items) item 13	9	0.011	99.48
Reusable components from reusable set (cruciate retaining femoral and tibial preparation and trialing set (size 1,2,7,8))	Instruments (bottom tray, 18 items) item 14	9	0.011	99.49
Reusable components from reusable set (cruciate retaining femoral and tibial preparation and trialing set (size 1,2,7,8))	Instruments (bottom tray, 18 items) item 15	9	0.011	99.50
Reusable components from reusable set (cruciate retaining femoral and tibial preparation and trialing set (size 1,2,7,8))	Instruments (bottom tray, 18 items) item 16	9	0.011	99.51
Reusable components from reusable set (cruciate retaining femoral and tibial preparation and trialing set (size 1,2,7,8))	Instruments (bottom tray, 18 items) item 17	9	0.011	99.52
Reusable components from reusable set (cruciate retaining femoral and tibial preparation and trialing set (size 1,2,7,8))	Instruments (bottom tray, 18 items) item 18	9	0.011	99.53
Single-use components from reusable set (cruciate retaining femoral and tibial trialing set (size 3-6))	Kit list	9	0.011	99.54
Single-use equipment and medical devices	Needle (white)	8	0.010	99.55
Reusable components from reusable set (orthopaedic surgical drill)	Tray	8	0.010	99.56
Single-use equipment and medical devices	Surgical blade (10) x3	8	0.009	99.57
Single-use equipment and medical devices	Catheter tip syringe	8	0.009	99.58
Cleaning products, waste bags	Yellow/ black waste bag (non-infectious offensive waste) with cable tie from scrub room	8	0.009	99.59
Reusable components from reusable set (basic major orthopaedic set)	Stitch scissor	8	0.009	99.60
Reusable components from reusable set (basic major orthopaedic set)	Gillies toothed dissecting forceps	7	0.009	99.61
Single-use components from reusable set (basic major orthopaedic set)	Filter paper	7	0.009	99.61
Single-use components from reusable set (miscellaneous knee system set)	Filter paper	7	0.009	99.62
Single-use components from reusable set (femoral and tibial preparation set (size 3-6))	Filter paper	7	0.009	99.63
Single-use components from reusable set (orthopaedic surgical drill)	Filter paper	7	0.009	99.64
Single-use equipment and medical devices	Suture (braided, absorbable 1-0, 932)	7	0.008	99.65
Reusable components from reusable set (basic major orthopaedic set)	Treves dissecting forceps	7	0.008	99.66
Single-use components from reusable set (patella preparation and trialing set)	Kit list	7	0.008	99.67
Single-use components of individually wrapped instruments (diathermy extras)	Flexible pouch	7	0.008	99.67
Single-use components of individually wrapped instruments (bipolar diathermy)	Flexible pouch	7	0.008	99.68
Single-use components of individually wrapped instruments (diathermy lead)	Flexible pouch	7	0.008	99.69
Single-use components from reusable set (basic major orthopaedic set)	Tamper proof tags	7	0.008	99.70
Single-use components from reusable set (miscellaneous knee system set)	Tamper proof tags	7	0.008	99.71
Single-use components from reusable set (femoral and tibial preparation set (size 3-6))	Tamper proof tags	7	0.008	99.71
Single-use components from reusable set (orthopaedic surgical drill)	Tamper proof tags	7	0.008	99.72
Reusable components from reusable set (basic major orthopaedic set)	Galabin ligature carrier	7	0.008	99.73
Single-use components from reusable set (cruciate retaining femoral and tibial trialing set (size 3-6))	Filter paper	7	0.008	99.74
Reusable components of individually wrapped instruments (diathermy extras)	Diathermy lead	6	0.008	99.75
Single-use components from reusable set (cruciate retaining femoral and tibial trialing set (size 3-6))	Tamper proof tags	6	0.007	99.75
Single-use equipment and medical devices	Wound closure strips	6	0.007	99.76
Reusable components from reusable set (basic major orthopaedic set)	McDonald dissector	6	0.007	99.77
Single-use equipment and medical devices	Suction tip	6	0.007	99.77
Reusable components of individually wrapped instruments (diathermy extras)	Bipolar diathermy forceps	5	0.006	99.78

Reusable components from reusable set (posterior stabilised femoral and tibial trialing set (size 3-6))	Tray	5	0.006	99.79
Single-use components of individually wrapped instruments (non-toothed lamina spreader (small))	Flexible pouch	5	0.006	99.79
Single-use components from reusable set (patella preparation and trialing set)	Filter paper	5	0.006	99.80
Reusable components of individually wrapped instruments (diathermy extras)	Monopolar diathermy	5	0.006	99.80
Single-use components from reusable set (patella preparation and trialing set)	Tamper proof tags	5	0.006	99.81
Single-use equipment and medical devices	Needle (red)	4	0.005	99.81
Single-use equipment and medical devices	Elasticated fabric dressing strip	4	0.005	99.82
Reusable components from reusable set (posterior stabilised femoral and tibial trialing set (size 3-6))	Instruments (bottom tray, 16 items) item 1	4	0.004	99.82
Reusable components from reusable set (posterior stabilised femoral and tibial trialing set (size 3-6))	Instruments (bottom tray, 16 items) item 2	4	0.004	99.83
Reusable components from reusable set (posterior stabilised femoral and tibial trialing set (size 3-6))	Instruments (bottom tray, 16 items) item 3	4	0.004	99.83
Reusable components from reusable set (posterior stabilised femoral and tibial trialing set (size 3-6))	Instruments (bottom tray, 16 items) item 4	4	0.004	99.84
Reusable components from reusable set (posterior stabilised femoral and tibial trialing set (size 3-6))	Instruments (bottom tray, 16 items) item 5	4	0.004	99.84
Reusable components from reusable set (posterior stabilised femoral and tibial trialing set (size 3-6))	Instruments (bottom tray, 16 items) item 6	4	0.004	99.84
Reusable components from reusable set (posterior stabilised femoral and tibial trialing set (size 3-6))	Instruments (bottom tray, 16 items) item 7	4	0.004	99.85
Reusable components from reusable set (posterior stabilised femoral and tibial trialing set (size 3-6))	Instruments (bottom tray, 16 items) item 8	4	0.004	99.85
Reusable components from reusable set (posterior stabilised femoral and tibial trialing set (size 3-6))	Instruments (bottom tray, 16 items) item 9	4	0.004	99.86
Reusable components from reusable set (posterior stabilised femoral and tibial trialing set (size 3-6))	Instruments (bottom tray, 16 items) item 10	4	0.004	99.86
Reusable components from reusable set (posterior stabilised femoral and tibial trialing set (size 3-6))	Instruments (bottom tray, 16 items) item 11	4	0.004	99.86
Reusable components from reusable set (posterior stabilised femoral and tibial trialing set (size 3-6))	Instruments (bottom tray, 16 items) item 12	4	0.004	99.87
Reusable components from reusable set (posterior stabilised femoral and tibial trialing set (size 3-6))	Instruments (bottom tray, 16 items) item 13	4	0.004	99.87
Reusable components from reusable set (posterior stabilised femoral and tibial trialing set (size 3-6))	Instruments (bottom tray, 16 items) item 14	4	0.004	99.88
Reusable components from reusable set (posterior stabilised femoral and tibial trialing set (size 3-6))	Instruments (bottom tray, 16 items) item 15	4	0.004	99.88
Reusable components from reusable set (posterior stabilised femoral and tibial trialing set (size 3-6))	Instruments (bottom tray, 16 items) item 16	4	0.004	99.89
Reusable components from reusable set (cruciate retaining femoral and tibial preparation and trialing set (size 1,2,7,8))	Tray	4	0.004	99.89
Single-use equipment and medical devices	Suture (monofilament, absorbable, 3-0, 643)	3	0.004	99.89
Reusable components from reusable set (basic major orthopaedic set)	Pin for instruments	3	0.004	99.90
Reusable components of individually wrapped instruments (diathermy extras)	Tray	3	0.004	99.90
Single-use equipment and medical devices	Transparent film adhesive dressing	3	0.004	99.90
Single-use components from reusable set (posterior stabilised femoral and tibial trialing set (size 3-6))	Kit list	3	0.004	99.91
Single-use equipment and medical devices	Surgical blade (10)	2	0.003	99.91
Single-use equipment and medical devices	Marker pen and ruler	2	0.003	99.91
Single-use equipment and medical devices	Pre-operative adhesive glove	2	0.003	99.92
Reusable components from reusable set (patella preparation and trialing set)	Instruments (bottom tray, 13 items) item 1	2	0.003	99.92
Reusable components from reusable set (patella preparation and trialing set)	Instruments (bottom tray, 13 items) item 2	2	0.003	99.92
Reusable components from reusable set (patella preparation and trialing set)	Instruments (bottom tray, 13 items) item 3	2	0.003	99.92
Reusable components from reusable set (patella preparation and trialing set)	Instruments (bottom tray, 13 items) item 4	2	0.003	99.93
Reusable components from reusable set (patella preparation and trialing set)	Instruments (bottom tray, 13 items) item 5	2	0.003	99.93
Reusable components from reusable set (patella preparation and trialing set)	Instruments (bottom tray, 13 items) item 6	2	0.003	99.93
Reusable components from reusable set (patella preparation and trialing set)	Instruments (bottom tray, 13 items) item 7	2	0.003	99.94
Reusable components from reusable set (patella preparation and trialing set)	Instruments (bottom tray, 13 items) item 8	2	0.003	99.94
Reusable components from reusable set (patella preparation and trialing set)	Instruments (bottom tray, 13 items) item 9	2	0.003	99.94
Reusable components from reusable set (patella preparation and trialing set)	Instruments (bottom tray, 13 items) item 10	2	0.003	99.94
Reusable components from reusable set (patella preparation and trialing set)	Instruments (bottom tray, 13 items) item 11	2	0.003	99.95
Reusable components from reusable set (patella preparation and trialing set)	Instruments (bottom tray, 13 items) item 12	2	0.003	99.95
Reusable components from reusable set (patella preparation and trialing set)	Instruments (bottom tray, 13 items) item 13	2	0.003	99.95
Single-use equipment and medical devices	Border dressing (6x8 cm)	2	0.003	99.95
Single-use components from reusable set (posterior stabilised femoral and tibial trialing set (size 3-6))	Filter paper	2	0.003	99.96
Single-use components from reusable set (posterior stabilised femoral and tibial trialing set (size 3-6))	Tamper proof tags	2	0.002	99.96

Single-use components from reusable set (cruciate retaining femoral and tibial preparation and trialing set (size 1,2,7,8))	Kit list	2	0.002	99.96
Reusable components from reusable set (knee navigation set)	Tray	2	0.002	99.96
Reusable components from reusable set (miscellaneous knee system set)	Pins (long, headless) x4 item 1	2	0.002	99.97
Reusable components from reusable set (miscellaneous knee system set)	Pins (long, headless) x4 item 2	2	0.002	99.97
Reusable components from reusable set (miscellaneous knee system set)	Pins (long, headless) x4 item 3	2	0.002	99.97
Reusable components from reusable set (miscellaneous knee system set)	Pins (long, headless) x4 item 4	2	0.002	99.97
Single-use equipment and medical devices	Diathermy tip	2	0.002	99.97
Single-use components from reusable set (cruciate retaining femoral and tibial preparation and trialing set (size 1,2,7,8))	Filter paper	1	0.002	99.98
Single-use equipment and medical devices	Needle (green)	1	0.002	99.98
Single-use components from reusable set (cruciate retaining femoral and tibial preparation and trialing set (size 1,2,7,8))	Tamper proof tags	1	0.002	99.98
Reusable components from reusable set (miscellaneous knee system set)	Drill bit/ pins x4 item 1	1	0.002	99.98
Reusable components from reusable set (miscellaneous knee system set)	Drill bit/ pins x4 item 2	1	0.002	99.98
Reusable components from reusable set (miscellaneous knee system set)	Drill bit/ pins x4 item 3	1	0.002	99.98
Reusable components from reusable set (miscellaneous knee system set)	Drill bit/ pins x4 item 4	1	0.002	99.99
Single-use components from reusable set (knee navigation set)	Kit list	1	0.001	99.99
Single-use components of individually wrapped instruments (diathermy extras)	Kit list	1	0.001	99.99
Reusable components from reusable set (miscellaneous knee system set)	Nails (short, headed) x4 item 1	1	0.001	99.99
Reusable components from reusable set (miscellaneous knee system set)	Nails (short, headed) x4 item 2	1	0.001	99.99
Reusable components from reusable set (miscellaneous knee system set)	Nails (short, headed) x4 item 3	1	0.001	99.99
Reusable components from reusable set (miscellaneous knee system set)	Nails (short, headed) x4 item 4	1	0.001	99.99
Single-use components from reusable set (knee navigation set)	Filter paper	1	0.001	99.99
Single-use components from reusable set (knee navigation set)	Tamper proof tags	1	0.001	99.99
Single-use equipment and medical devices	Sticky label	1	0.001	99.99
Reusable components of individually wrapped instruments (light handle)	Identification tag	0.43	0.001	100.00
Reusable components from reusable set (basic major orthopaedic set)	Identification tag	0.39	0.00046	100.00
Reusable components from reusable set (miscellaneous knee system set)	Identification tag	0.39	0.00046	100.00
Reusable components from reusable set (femoral and tibial preparation set (size 3-6))	Identification tag	0.39	0.00046	100.00
Reusable components from reusable set (orthopaedic surgical drill)	Identification tag	0.39	0.00046	100.00
Reusable components of individually wrapped instruments (light cover)	Identification tag	0.39	0.00046	100.00
Reusable components from reusable set (cruciate retaining femoral and tibial trialing set (size 3-6))	Identification tag	0.35	0.00041	100.00
Reusable components of individually wrapped instruments (Semb bone holding forceps)	Identification tag	0.31	0.00036	100.00
Reusable components from reusable set (patella preparation and trialing set)	Identification tag	0.27	0.00032	100.00
Reusable components from reusable set (basic major orthopaedic set)	Pin mat	0.21	0.00024	100.00
Reusable components of individually wrapped instruments (Lanes tissue forceps)	Identification tag	0.19	0.00023	100.00
Reusable components from reusable set (posterior stabilised femoral and tibial trialing set (size 3-6))	Identification tag	0.12	0.00014	100.00
Non-set reusable equipment	Shaver base	0.09	0.00010	100.00
Reusable components from reusable set (cruciate retaining femoral and tibial preparation and trialing set (size 1,2,7,8))	Identification tag	0.08	0.00009	100.00
Reusable components of individually wrapped instruments (blunt Hohman bone elevator)	Identification tag	0.08	0.00009	100.00
Reusable components of individually wrapped instruments (non-toothed lamina spreader (large))	Identification tag	0.08	0.00009	100.00
Reusable components from reusable set (knee navigation set)	Pin mat	0.04	0.00005	100.00
Reusable components from reusable set (knee navigation set)	Identification tag	0.04	0.00005	100.00
Reusable components of individually wrapped instruments (diathermy extras)	Identification tag	0.04	0.00005	100.00
Reusable components of individually wrapped instruments (diathermy lead)	Identification tag	0.04	0.00005	100.00
Reusable components of individually wrapped instruments (non-toothed lamina spreader (small))	Identification tag	0.04	0.00005	100.00

Supplementary Table 26: Mean average carbon footprint of products used for laparoscopic cholecystectomy

Mean average carbon footprint of items used across six operations (L1-L10), in order of contribution. CO_2e= carbon dioxide equivalents. Mean average across six operations (L1-L6)

Product category	Product	Mean average carbon footprint (g CO_2e)	Percentage of mean average total (%)	Cumulative percentage (%)
Single-use equipment and medical devices	Endoscopic clip applier	1,590	7.83	7.83
Reusable patient or instrument table drape	High fluid drape	1,088	5.36	13.19
Single-use equipment and medical devices	Port (12 mm)	1,077	5.31	18.50
Pharmaceuticals	Levobupivacaine 2.5 mg/10 ml (10 ml)	756	3.72	22.23
Single-use equipment and medical devices	Suction irrigation	743	3.66	25.89
Single-use patient or instrument table drape	Table drape (instrument)	740	3.65	29.54
Single-use equipment and medical devices	Port (5 mm, dual pack)	655	3.23	32.77
Reusable components from reusable set (general basic set)	Container	643	3.17	35.94
Single-use equipment and medical devices	Insufflating tubing	600	2.96	38.89
Single-use equipment and medical devices	Anti-fog endoscopic demister	565	2.78	41.67
Single-use equipment and medical devices	Laparoscopic scissors	551	2.72	44.39
Reusable components from reusable set (Laparoscope set (10 mm, 0 degree))	Light lead	533	2.63	47.02
Single-use components from reusable set (Laparoscope set (10 mm, 0 degree))	Tray wrap (inner, 100x100 cm)	444	2.19	49.21
Single-use personal protective equipment	Surgical gown (including hand towels)	425	2.09	51.30
Pharmaceuticals	Carbon dioxide (from 3 kg cylinder containing 450 L)	423	2.09	53.39
Reusable components from reusable set (general laparoscopic set)	Container base (deep)	399	1.97	55.36
Reusable personal protective equipment	Reusable scrubs	368	1.81	57.17
Reusable personal protective equipment	Reusable surgical gown (including hand towels)	337	1.66	58.83
Reusable components from reusable set (Laparoscope set (10 mm, 0 degree))	Wire cage with lid	322	1.59	60.42
Single-use personal protective equipment	Sterile gloves (two pairs)	289	1.42	61.84
Reusable personal protective equipment	Reusable surgical gown (including hand towels) double pack	270	1.33	63.17
Single-use equipment and medical devices	Gauze (sterile pack)	256	1.26	64.44
Single-use equipment and medical devices	Needle counter	254	1.25	65.69
Reusable components from reusable set (general laparoscopic set)	Container lid (deep)	247	1.22	66.90
Reusable components from reusable set (Laparoscope set (10 mm, 0 degree))	Hopkins laparoscope (10 mm, 0 degree)	239	1.18	68.08
Reusable components from reusable set (general laparoscopic set)	Kelly crocodile grasping forceps x2	229	1.13	69.21
Single-use components from reusable set (Laparoscope set (10 mm, 0 degree))	Tray wrap (outer, 100x100 cm)	228	1.13	70.34
Reusable components from reusable set (general basic set)	Kidney dish (25 cm) x4	196	0.97	71.30
Single-use personal protective equipment	Sterile gloves (pair)	190	0.94	72.24
Cleaning products, waste bags	Mop head	186	0.91	73.16
Reusable components from reusable set (general laparoscopic set)	Langenbeck retractor (small) x2	172	0.85	74.00
Cleaning products, waste bags	Orange waste bag (infectious waste, large) with cable tie	154	0.76	74.76
Reusable components from reusable set (general laparoscopic set)	Raptor toothed grasping forceps	152	0.75	75.51
Cleaning products, waste bags	Disinfectant wipe	149	0.73	76.24
Pharmaceuticals	Sodium Chloride 0.9% for irrigation (1 L bag)	148	0.73	76.98
Reusable components from reusable set (general laparoscopic set)	Diathermy lead	143	0.71	77.68
Single-use equipment and medical devices	Laparoscope cover	139	0.69	78.37
Reusable components from reusable set (quiver and clip)	Quiver	137	0.68	79.04

Reusable components from reusable set (general basic set)	Rampley sponge holder forceps x4	127	0.63	79.67
Single-use personal protective equipment	Gloves (non-sterile, pair)	124	0.61	80.28
Reusable components from reusable set (general laparoscopic set)	Kocher artery forceps x2	121	0.59	80.87
Reusable components from reusable set (general basic set)	Schnidt artery forceps x6	117	0.58	81.45
Single-use equipment and medical devices	Suction receptacle	117	0.58	82.03
Reusable components from reusable set (general laparoscopic set)	Manhes grasping forceps	116	0.57	82.60
Single-use equipment and medical devices	Pressure saline infusion bag	113	0.56	83.16
Reusable components from reusable set (general laparoscopic set)	Pietlyn dissecting forceps	113	0.56	83.71
Reusable components from reusable set (general laparoscopic set)	Johann grasping forceps	110	0.54	84.26
Reusable components from reusable set (general basic set)	Towel clip x6	105	0.52	84.77
Reusable components from reusable set (general laparoscopic set)	Littlewoods tissue forceps x2	105	0.52	85.29
Cleaning products, waste bags	Black waste bag (domestic waste) with cable tie	105	0.52	85.80
Reusable components from reusable set (general basic set)	Spencer Wells curved artery forceps x4	103	0.51	86.31
Reusable components from reusable set (general laparoscopic set)	Lahey right angle dissecting forceps	102	0.50	86.82
Reusable components from reusable set (general laparoscopic set)	Maryland dissecting forceps	102	0.50	87.32
Single-use equipment and medical devices	Specimen pot (not pre-filled)	92	0.46	87.77
Single-use equipment and medical devices	Syringe (20 ml)	83	0.41	88.18
Single-use equipment and medical devices	Incontinence pad	82	0.40	88.59
Reusable patient or instrument table drape	Huck towel	77	0.38	88.97
Reusable components from reusable set (general basic set)	Spencer Wells straight artery forceps x6	72	0.35	89.32
Single-use components from reusable set (general basic set)	Gallipot x3	71	0.35	89.67
Reusable components from reusable set (general basic set)	Treves toothed dissecting forceps x6	70	0.34	90.02
Cleaning products, waste bags	Orange waste bag (infectious waste, small) with cable tie	68	0.34	90.35
Single-use equipment and medical devices	Diathermy pad	68	0.33	90.68
Reusable components from reusable set (general basic set)	Basket	62	0.31	90.99
Reusable components from reusable set (general basic set)	Czerny retractor x2	61	0.30	91.29
Reusable components from reusable set (general basic set)	Halstead mosquito curved artery forceps x6	60	0.30	91.59
Cleaning products, waste bags	Clear bin bag (quiver diathermy)	60	0.29	91.88
Cleaning products, waste bags	Yellow/ black waste bag (non-infectious offensive waste, large) with cable tie	58	0.29	92.17
Reusable components from reusable set (general basic set)	Diathermy lead	54	0.26	92.43
Pharmaceuticals	Chlorhexidine 1% from 500 ml container (50 ml)	54	0.26	92.70
Reusable components from reusable set (general basic set)	Travers self retaining retractor	53	0.26	92.96
Reusable components from reusable set (general basic set)	Langenbeck retractor (medium) x2	52	0.25	93.22
Single-use components from reusable set (quiver and clip)	Flexible pouch	51	0.25	93.47
Reusable components from reusable set (general basic set)	Diathermy quiver	47	0.23	93.70
Reusable components from reusable set (general laparoscopic set)	Desjardin forceps	43	0.21	93.92
Single-use equipment and medical devices	Specimen pot (40 ml 4% formaldehyde)	42	0.21	94.12
Reusable components from reusable set (general basic set)	Lanes tissue forceps x2	41	0.20	94.33
Reusable components from reusable set (general laparoscopic set)	Diathermy hook	40	0.20	94.53
Reusable components from reusable set (general basic set)	Bipolar diathermy	40	0.19	94.72
Single-use equipment and medical devices	Laparoscopic tissue retrieval system	39	0.19	94.91
Single-use personal protective equipment	Surgical face mask	39	0.19	95.10
Pharmaceuticals	Sodium Chloride 0.9% from 1 litre bottle (50 ml)	38	0.19	95.29
Cleaning products, waste bags	Yellow/ black waste bag (non-infectious offensive waste, small) with cable tie	37	0.18	95.47
Reusable components from reusable set (general basic set)	Mayo curved scissors	35	0.17	95.64
Cleaning products, waste bags	Disinfectant sachet	34	0.17	95.81
Single-use equipment and medical devices	Nail scrubbing brush	33	0.16	95.97

Cleaning products, waste bags	Clear bin bag (recycling) with cable tie	33	0.16	96.13
Cleaning products, waste bags	Red bag (linen laundering) with cable tie	32	0.16	96.29
Single-use equipment and medical devices	Nonwoven dressing (6x7 cm)	32	0.16	96.45
Single-use equipment and medical devices	Tonsil swab pack	32	0.16	96.61
Reusable components from reusable set (general basic set)	Bonney toothed dissecting forceps	30	0.15	96.75
Reusable components from reusable set (general basic set)	Mayo Hegar needle holder x2	29	0.14	96.90
Reusable components from reusable set (general basic set)	BP scalpel handle (no. 4) x2	29	0.14	97.04
Single-use components from reusable set (general basic set)	Bag plain closure (H)	28	0.14	97.17
Reusable components from reusable set (general basic set)	Allis tissue forceps x2	28	0.14	97.31
Single-use equipment and medical devices	Syringe (10 ml)	27	0.13	97.44
Reusable components from reusable set (diathermy lead)	Diathermy lead	25	0.13	97.57
Reusable components from reusable set (general basic set)	Bulldog clip	25	0.12	97.69
Reusable components from reusable set (general basic set)	BP scalpel handle (no. 3) x2	25	0.12	97.81
Reusable components from reusable set (general basic set)	McIndoe plain diathermy forceps	24	0.12	97.93
Reusable components from reusable set (laparoscopic grasping forceps)	Laparoscopic grasping forceps	24	0.12	98.05
Reusable components from reusable set (general basic set)	Babcock tissue forceps x2	24	0.12	98.17
Single-use equipment and medical devices	Suture (monofilament, absorbable, 1)	24	0.12	98.29
Reusable components from reusable set (general basic set)	Mayo straight scissors	22	0.11	98.39
Single-use personal protective equipment	Surgical hat	20	0.10	98.49
Reusable components from reusable set (general basic set)	Plain dissecting forceps (7")	18	0.09	98.58
Single-use personal protective equipment	Surgical face mask with eye protection	18	0.09	98.67
Cleaning products, waste bags	Green bag (linen laundering) with cable tie	17	0.09	98.76
Single-use personal protective equipment	Sterile gloves (one pair, latex free)	17	0.08	98.84
Reusable components from reusable set (general basic set)	McIndoe scissors	17	0.08	98.93
Reusable components from reusable set (general basic set)	Stitch scissor	16	0.08	99.01
Reusable components from reusable set (general basic set)	Plain dissecting forceps (5")	15	0.07	99.08
Single-use equipment and medical devices	Absorbent towel pack	13	0.06	99.14
Reusable components from reusable set (general laparoscopic set)	Laparoscopic instrument rack	13	0.06	99.20
Reusable components from reusable set (general basic set)	Gillies toothed dissecting forceps	12	0.06	99.26
Single-use components from reusable set (laparoscopic grasping forceps)	Flexible pouch	12	0.06	99.32
Reusable components from reusable set (general basic set)	Debakey dissecting forceps	11	0.05	99.37
Reusable components from reusable set (general basic set)	McIndoe dissecting forceps	10	0.05	99.42
Single-use components from reusable set (general basic set)	Kit list	10	0.05	99.47
Single-use components from reusable set (general laparoscopic set)	Kit list	10	0.05	99.52
Single-use components from reusable set (Laparoscope set (10 mm, 0 degree))	Kit list	10	0.05	99.57
Reusable components from reusable set (quiver and clip)	Clip	9	0.05	99.62
Non-set reusable equipment	Diathermy pad lead	9	0.05	99.66
Single-use components from reusable set (diathermy lead)	Flexible pouch	9	0.04	99.70
Single-use components from reusable set (general basic set)	Filter paper	7	0.04	99.74
Single-use components from reusable set (general laparoscopic set)	Filter paper	7	0.04	99.77
Single-use components from reusable set (general basic set)	Tamper proof tags	7	0.03	99.81
Single-use components from reusable set (general laparoscopic set)	Tamper proof tags	7	0.03	99.84
Single-use equipment and medical devices	Suture (braided, absorbable, 3-0)	7	0.03	99.88
Single-use equipment and medical devices	Light handle	5	0.02	99.90
Single-use equipment and medical devices	Surgical blade (15)	5	0.02	99.92
Single-use equipment and medical devices	Reinforced skin closure strip	4	0.02	99.94
Single-use equipment and medical devices	Needle (green)	3	0.02	99.96
Single-use equipment and medical devices	Suture (monofilament, absorbable, 3-0)	3	0.01	99.97

Reusable personal protective equipment	Reusable surgical hat	2	0.01	99.98
Single-use equipment and medical devices	Surgical blade (11)	1	0.01	99.99
Reusable components from reusable set (Laparoscope set (10 mm, 0 degree))	Identification tag	1	0.0032	99.99
Reusable components from reusable set (general laparoscopic set)	Identification tag	1	0.0028	100.00
Reusable components from reusable set (general basic set)	Identification tag	0.4	0.0019	100.00
Reusable components from reusable set (quiver and clip)	Identification tag	0.4	0.0019	100.00
Reusable components from reusable set (diathermy lead)	Identification tag	0.1	0.0003	100.00
Reusable components from reusable set (laparoscopic grasping forceps)	Identification tag	0.1	0.0003	100.00

Supplementary Table 27: Mean average carbon footprint of products used for tonsillectomy

Mean average carbon footprint of items used across ten operations (T1-T10), in order of contribution. Mean is dependent upon number of items used, and number of operations using the item. CO_2e= carbon dioxide equivalents.

Product category	Product	Mean average carbon footprint (g CO_2e)	Percentage of mean average total (%)	Cumulative percentage (%)
Single-use patient or instrument table drape	Table drape (instruments)	840	11.25	11.25
Single-use equipment and medical devices	Suction tubing	539	7.21	18.45
Reusable components from reusable set (tonsillectomy set A)	Container	514	6.88	25.34
Reusable personal protective equipment	Reusable surgical gown (including hand towels)	474	6.34	31.68
Single-use equipment and medical devices	Coblation™ wand	466	6.24	37.92
Single-use equipment and medical devices	Suction receptacle	433	5.79	43.71
Single-use personal protective equipment	Sterile gloves (one pair)	298	3.99	47.69
Reusable personal protective equipment	Reusable scrubs	232	3.11	50.80
Single-use patient or instrument table drape	Patient drape (fenestrated ENT drape)	220	2.94	53.74
Reusable components from reusable set (tonsillectomy set A)	Draffin bipod stand (long) x2	161	2.16	55.90
Cleaning products, waste bags	Mop head	160	2.14	58.04
Pharmaceuticals	Sodium chloride 0.9%, intravenous infusion bag (500 ml)	146	1.95	59.99
Reusable components from reusable set (tonsillectomy set A)	Draffin bipod stand (short) x2	139	1.86	61.85
Reusable components from reusable set (tonsillectomy set B)	Container	129	1.72	63.57
Pharmaceuticals	Chirocaine 2.5mg/ml (10 ml)	126	1.68	65.25
Cleaning products, waste bags	Yellow waste bag (clinical waste) with cable tie	123	1.64	66.89
Pharmaceuticals	Sodium chloride 0.9% from 1 litre bottle (150 ml)	114	1.52	68.41
Single-use equipment and medical devices	Tonsil swab pack	96	1.29	69.70
Reusable patient or instrument table drape	Reusable ENT split head drape (two 42"x42")	94	1.25	70.95
Single-use equipment and medical devices	Yankauer sucker	90	1.20	72.15
Cleaning products, waste bags	Black waste bag (domestic waste) with cable tie	84	1.12	73.27
Cleaning products, waste bags	Clear waste bag (for swab count)	81	1.09	74.36
Cleaning products, waste bags	Clear waste bag (recycling) with cable tie	79	1.05	75.41
Reusable components from reusable set (tonsillectomy set A)	Bipolar diathermy lead	78	1.05	76.46
Cleaning products, waste bags	Disinfectant wipe	75	1.00	77.46
Pharmaceuticals	Bupivacaine 0.5% with 1:200,000 adrenaline (10 ml)	74	0.99	78.45
Reusable components from reusable set (tonsillectomy set A)	Boyle Davis gag	73	0.98	79.43
Reusable components from reusable set (tonsillectomy set A)	Adenoid St Clair Thompson curette	69	0.93	80.36
Cleaning products, waste bags	Orange waste bag (infectious waste) with cable tie	61	0.82	81.18
Reusable components from reusable set (tonsillectomy set A)	Dennis Browne forceps	61	0.82	82.00
Reusable components from reusable set (tonsillectomy set A)	Boyle Davis tongue plate (99 mm)	61	0.81	82.82
Reusable components from reusable set (tonsillectomy set A)	Boyle Davis tongue plate (89 mm)	59	0.79	83.60
Reusable components from reusable set (tonsillectomy set A)	Boyle Davis tongue plate (74 mm)	52	0.70	84.30
Single-use equipment and medical devices	Gallipot	47	0.62	84.92
Reusable components from reusable set (tonsillectomy set A)	Beckman curette	46	0.62	85.54
Reusable components from reusable set (tonsillectomy set A)	Luc forceps (large)	46	0.61	86.16
Reusable components from reusable set (tonsillectomy set A)	Basket	45	0.60	86.76
Single-use personal protective equipment	Gloves (non-sterile, one pair)	44	0.59	87.35

Reusable components from reusable set (tonsillectomy set A)	Hurd dissector/ pillar retractor	43	0.58	87.93
Reusable components from reusable set (tonsillectomy set A)	Kidney dish (20 cm)	40	0.53	88.46
Reusable components from reusable set (tonsillectomy set A)	Bulldog clip	38	0.51	88.97
Reusable components from reusable set (tonsillectomy set A)	Negus knot pusher	36	0.48	89.45
Reusable components from reusable set (tonsillectomy set B)	Draffin bipod stand x 2	35	0.47	89.92
Reusable components from reusable set (tonsillectomy set A)	Negus curved artery forceps (large)	34	0.46	90.38
Reusable components from reusable set (tonsillectomy set A)	Birkett straight tonsil forceps	33	0.44	90.81
Reusable components from reusable set (tonsillectomy set A)	Metzenbaum curved scissors	32	0.43	91.25
Reusable components from reusable set (tonsillectomy set A)	Boyle Davis tongue plate (64 mm)	32	0.43	91.68
Reusable components from reusable set (tonsillectomy set A)	Bipolar diathermy forceps (8")	31	0.42	92.09
Cleaning products, waste bags	Disinfectant sachet	27	0.36	92.45
Reusable components from reusable set (tonsillectomy set A)	Wilson tonsil artery forceps	26	0.35	92.81
Reusable components from reusable set (tonsillectomy set A)	Gwyne Evans tonsil dissector	24	0.33	93.13
Reusable components from reusable set (tonsillectomy set A)	Dissecting forceps (long, toothed)	23	0.31	93.45
Cleaning products, waste bags	Green bag (linen laundering) with cable tie	21	0.28	93.73
Single-use personal protective equipment	Surgical face mask with eye protection	20	0.27	94.00
Reusable components from reusable set (tonsillectomy set B)	Boyle Davis gag (adult)	20	0.26	94.26
Reusable components from reusable set (tonsillectomy set B)	Small receiver x2	19	0.25	94.52
Single-use equipment and medical devices	Braided silk tonsil ties	19	0.25	94.77
Reusable components from reusable set (tonsillectomy set B)	Bipolar diathermy lead	19	0.25	95.02
Reusable components from reusable set (tonsillectomy set B)	Kidney dish (25 cm) x2	18	0.24	95.27
Reusable components from reusable set (tonsillectomy set B)	Diathermy quiver	18	0.24	95.50
Reusable components from reusable set (tonsillectomy set B)	Boyle Davis gag (paediatric)	17	0.23	95.73
Single-use equipment and medical devices	Specimen pot (40 ml 4% formaldehyde)	16	0.21	95.94
Single-use personal protective equipment	Surgical face mask	15	0.20	96.15
Reusable components from reusable set (tonsillectomy set B)	Doughty tongue plate (4")	15	0.20	96.35
Single-use equipment and medical devices	Suction tip	15	0.20	96.55
Reusable components from reusable set (tonsillectomy set B)	Masson needle holder (10")	14	0.19	96.75
Reusable components from reusable set (tonsillectomy set B)	Basket	13	0.18	96.92
Reusable components from reusable set (tonsillectomy set B)	Doughty tongue plate (3.5")	13	0.17	97.09
Single-use equipment and medical devices	Nail scrubbing brush	12	0.16	97.26
Reusable components from reusable set (tonsillectomy set B)	Yankauer sucker	12	0.16	97.41
Reusable components from reusable set (tonsillectomy set B)	Towel clip (ball/socket)	11	0.15	97.56
Reusable components from reusable set (tonsillectomy set B)	Luc forceps (large)	11	0.15	97.71
Reusable components from reusable set (tonsillectomy set B)	Woods ENT scissors	10	0.13	97.84
Single-use personal protective equipment	Surgical hat	9	0.12	97.97
Reusable components from reusable set (tonsillectomy set B)	Bulldog clip	9	0.12	98.09
Single-use components from reusable set (tonsillectomy set B)	Gallipot (60 ml) x2	8	0.11	98.20
Reusable components from reusable set (tonsillectomy set B)	Negus knot pusher	8	0.11	98.32
Reusable components from reusable set (tonsillectomy set B)	Negus curved artery forceps(large)	8	0.11	98.43
Single-use components from reusable set (tonsillectomy set A)	Kit list	8	0.11	98.53
Reusable components from reusable set (tonsillectomy set B)	Birketts straight tonsil forceps	8	0.10	98.64
Single-use equipment and medical devices	Incontinence pad	8	0.10	98.74
Reusable components from reusable set (tonsillectomy set B)	Bipolar forceps (8")	7	0.10	98.84
Single-use equipment and medical devices	Kidney dish	7	0.10	98.93
Reusable components from reusable set (tonsillectomy set B)	Mcindoe curved scissor (7")	7	0.10	99.03
Reusable components from reusable set (tonsillectomy set B)	Waugh toothed dissecting forceps (8")	7	0.10	99.13
Reusable components from reusable set (tonsillectomy set B)	Mollison pillar retractor	7	0.09	99.22

Reusable components from reusable set (tonsillectomy set B)	Wilson tonsil artery forceps	6	0.08	99.30
Reusable personal protective equipment	Reusable surgical hat	6	0.08	99.38
Single-use components from reusable set (tonsillectomy set A)	Filter paper	6	0.08	99.46
Reusable components from reusable set (tonsillectomy set B)	Gwyne Evans tonsil dissector	6	0.08	99.54
Single-use components from reusable set (tonsillectomy set B)	Bag plain closure (H)	6	0.08	99.61
Single-use components from reusable set (tonsillectomy set A)	Tamper proof tags	6	0.07	99.69
Single-use components from reusable set (tonsillectomy set A)	Gag guard	5	0.07	99.75
Reusable components from reusable set (tonsillectomy set B)	Treves plain dissecting forceps (5")	4	0.06	99.81
Single-use equipment and medical devices	Gauze (non-sterile)	3	0.04	99.85
Single-use equipment and medical devices	Syringe (10 ml)	3	0.04	99.89
Single-use components from reusable set (tonsillectomy set B)	Kit list	2	0.03	99.92
Single-use components from reusable set (tonsillectomy set B)	Gag guard	2	0.02	99.94
Single-use components from reusable set (tonsillectomy set B)	Filter paper	1	0.02	99.96
Single-use components from reusable set (tonsillectomy set B)	Tamper proof tags	1	0.02	99.98
Pharmaceuticals	Yellow soft paraffin BP 100% from 15g tube	1	0.01	99.99
Single-use equipment and medical devices	Filter needle	1	0.01	99.99
Reusable components from reusable set (tonsillectomy set A)	Identification tag	0.3	0.004	100.00
Reusable components from reusable set (tonsillectomy set B)	Identification tag	0.1	0.001	100.00

Supplementary table 28 Product types responsible for majority (≥80%) of carbon footprint of each operation.
Products listed are those responsible cumulatively for mean average ≥80% carbon footprint of each operation type. Mean average contribution of each product type across operations is specified in parenthesis (carbon footprint; percentage contribution), and will depend on the carbon footprint of the item, number of items used, and number of operations in which item was used. Individual products contributing >10% carbon footprint indicated in dark red, 5-10% in red, 3-5% orange, <1% green. Items are clustered in yellow or blue (alternating) where items are used across multiple operation types, and are ordered by magnitude of carbon footprint within clusters.

Item name (mean average carbon footprint g CO_2e for operation type; mean average % contribution to total for operation type)

		Carpal tunnel decompression (21/77 product types)	Inguinal hernia repair (35/148 product types)	Knee arthroplasty (47/463 product types)	Laparoscopic cholecystectomy (40/141 product types)	Tonsillectomy (28/104 product types)
**Reusable instruments**	**Instrument sets and individually wrapped instruments**		General basic set B container (429; 4%)	Basic major ortho set container (644; 1%)<br>Femoral and tibial preparation set size 3-6 container (644; 1%)<br>Miscellaneous knee system set container (644; 1%)<br>Orthopedic surgical drill container (644; 1%)<br>Cruciate retaining femoral and tibial trialing set (size 3-6) container (579; 1%)<br>Patella preparation and trialing set container (451; 1%)	General basic set container (643; 3%)<br>Laparoscopic set container base (399; 2%)<br>Laparoscopic set wire cage (322; 2%)<br>Laparoscopic set container lid (247; 1%)	Tonsillectomy set A container (514; 7%)<br>Tonsillectomy set B container (129; 2%)
		Bipolar lead (268; 2%)<br>Sponge holder forceps (222;2%)<br>Weitlaner retractor (181; 2%)	Kidney dish (131; 1%)<br>Rampley sponge holder forceps (85; 1%)<br>Schnidt artery forceps (78; 1%)<br>Towel clips (70; 1%)<br>Spencer Wells curved artery forceps (69; 1%)	Heath mallet (283; 0.3%)	Laparoscope light lead (533; 3%)<br>Hopkins laparoscope (239; 1%)<br>Kelly crocodile grasping forceps (229; 1%)<br>Kidney dish (196; 1%)<br>Langenbeck retractor small (172; 1%)<br>Raptor toothed grasping forceps (152; 1%)<br>Diathermy lead (143; 1%)<br>Quiver (137; 1%)<br>Rampley sponge holder forceps (127; 1%)	Long Draffin bipod stand (161; 2%)<br>Short Draffin bipod stand (139; 2%)<br>Bipolar diathermy lead (78; 1%)<br>Boyle Davis gag (73; 1%)<br>St Clair Thompson Adenoid curette (69; 1%)
	**Personal protective equipment**	Scrubs (265; 2%)	Scrubs (290; 2%)	Scrubs (433; 1%)	Scrubs (368; 2%)	Scrubs (232; 3%)
			Surgical gown (269; 2%)		Surgical gown 1/pack (337; 2%)<br>Surgical gown 2/pack (270; 1%)	Surgical gown (474; 6%)
	**Patient and/or instrument table drapes**		High fluid drape (732; 6%)<br>Low fluid drape (248; 2%)		High fluid drape (1,088; 5%)	ENT split head drape (94; 1%)
**Single-use items**	**Single-use components reusable instrument sets**	Outer tray wrap (244; 2%)<br>Inner tray wrap (123; 1%)	Outer tray wrap (209; 2%)<br>Inner tray wrap (205; 2%)		Inner tray wrap (444; 2%)<br>Outer tray wrap (228; 2%)	
	**Personal protective equipment**	Surgical gown (2,063; 17%)	Surgical gown (1,138; 10%)	Surgical gown (1,826; 2%)<br>Surgical gown from knee pack (1,089; 1%)	Surgical gown (270; 2%)	
		Sterile gloves (298; 2%)	Sterile gloves 1 pair/pack (215; 2%)<br>Sterile gloves 2 pairs/pack (179; 2%)<br>Non-sterile gloves (135; 1%)<br>Latex free sterile gloves (76; 1%)	Sterile gloves 1 pair/ pack (670; 1%)<br>Sterile gloves 2 pairs/ pack (501; 1%)	Sterile gloves 2 pairs/ pack (289; 1%)<br>Sterile gloves 1 pair/ pack (190; 1%)<br>Non-sterile gloves (124; 1%)	Sterile gloves (298; 4%)
				Orthopedic hood (1,345; 2%)		
	**Patient and/or instrument table drapes**	Instrument table drape (1,050; 9%)	Instrument table drape (646; 6%)	Instrument table drape 160x240 cm (3,009; 4%)<br>Instrument table drape 140x90 cm (1,406; 2%)<br>Instrument table drape 140x190 cm (1,038; 2%)	Instrument table drape (740; 4%)	Instrument table drape (840; 11%)

Single -use items		Fenestrated hand drape (2,740; 23%)		Patient extremity drape (1,636; 2%)<br>Patient drape fluid collection (1,287; 2%)<br>Patient drape 240x150 cm (1,163; 2%)<br>Mayo patient drape (842; 1%)<br>Impervious split patient drape (644; 1%)<br>Stockinette impervious patient drape (584; 1%)		Fenestrated ENT drape (220; 3%)
	**Equipment and medical devices**	Gauze (224; 2%)<br>Gauze from hand pack (188; 2%)	Gauze 10x7.5 cm (380; 3%)<br>Gauze 10x10 cm (142; 1%)<br>Gauze 30x30 cm (119; 1%)		Gauze (256; 1%)	Tonsil swab pack (96; 1%)
		Needle counter (237; 2%)	Needle counter (254; 2%)		Needle counter (254; 1%)	
				Pulsed lavage system (3,727; 4%)<br>Suction tubing (437; 1%)	Suction irrigation (743; 4%)	Suction tubing (539; 7%)<br>Suction receptacle (433; 6%)<br>Yankauer sucker (90; 1%)
		Crepe bandage (309; 3%)		Crepe bandage (550; 1%)		
			20 ml syringe (104; 1%)	50 ml syringe (301; 0.4%)		
		Kidney dish (147; 1%)<br>Bowl (127; 1%)	Monopolar diathermy with smoke evacuation (675; 6%)<br>Mesh (246; 2%)<br>Incontinence pad (154; 1%)<br>Diathermy pad (97; 1%)<br>Surgical suspensory bandage (67; 1%)	Posterior stabilized femoral implant (3,528; 4%)<br>Primary tibial baseplate implant (2,997; 4%)<br>Cruciate retaining femoral implant (2,759; 3%)<br>Tibial bearing insert cruciate retaining implant (710; 1%)<br>Tibial bearing insert posterior stabilised implant (444; 1%)<br>Symmetric patella implant (359; 0.4%)<br>Cement mixing & delivering system (1,315; 2%)<br>Tourniquet pressure cuff (980; 1%)<br>Small tray (589; 1%)<br>Swab tray (322; 0.4%)<br>Incontinence pad (261; 0.3%)	Endoscopic clip applier (1,590; 8%)<br>12 mm port (1,077; 5%)<br>5 mm port (655; 3%)<br>Laparoscopic scissors (551; 3%)<br>Insufflation tubing (600; 3%)<br>Anti-fog endoscopic demister (565; 3%)<br>Laparoscope cover (139; 1%)	Coblator (466; 6%)
	**Pharmaceuticals**	Lidocaine with adrenaline (211; 2%)<br>Lidocaine (151; 1%)<br>Bupivacaine hydrochloride (125; 1%)	Levobupivacaine (602; 5%)	Ropivacaine (623; 1%)	Levobupivacaine (756; 4%)	Chirocaine (126; 2%)<br>Bupivacaine with adrenaline (74; 1%)
				Sodium chloride for irrigation (918; 1%)	Sodium chloride for irrigation (148; 1%)	Sodium chloride infusion bag (146; 2%)<br>Sodium chloride (114; 2%)
			Topical skin adhesive (654; 6%)	Topical skin adhesive (392; 0.5%)		
			Chlorhexidine (72; 1%)	Chlorhexidine gluconate in denatured ethanol with red stain solution (393; 0.5%)		
				Bone cement with tobramycin (18,499; 22%)<br>Bone cement with gentamycin (5,281; 6%)	Carbon dioxide (423; 2%)	
	**Cleaning products, waste**	Orange waste bag (307; 3%)	Orange waste bag, large (205; 2%)<br>Orange waste bag, small (91; 1%)	Orange waste bag (615; 1%)	Orange waste bag (154; 1%)	Yellow waste bag (123; 2%)<br>Black waste bag (84; 1%)<br>Clear waste bag- swab count (81;1%)<br>Clear waste bag- recycling (79; 1%)
		Mop head (152; 1%)	Mop head (194; 2%)	Mop head (253; 0.3%)	Mop head (186; 1%)	Mop head (160; 2%)
			Disinfectant wipe (140; 1%)	Disinfectant wipe (486; 1%)	Disinfectant wipe (149; 1%)	Disinfectant wipe (75; 1%)

Supplementary Table 29: Carbon footprint of processes across life cycle of products used in all five operations
C1=carpal tunnel decompression operation one etc. CO_2e= carbon dioxide equivalents

Operation		Carbon footprint (g CO_2e)								
		**Reusable items**					**Single-use items**			**Total for operation**
		Production all reusables	Production single-use packaging for reusables	Decontamin -ation	Linen laundering	Waste disposal all reusables	Production single-use items	Production packaging for single-use items	Waste disposal all single-use items	
Carpal tunnel decompression	C1	112	310	1,531	194	87	8,071	475	1,278	**12,058**
	C2	112	310	1,531	194	87	8,166	479	1,279	**12,158**
	C3	112	310	1,531	194	87	8,278	482	1,287	**12,281**
	C4	119	310	1,531	233	87	8,805	483	1,295	**12,863**
	C5	67	305	1,531	272	73	7,994	515	1,287	**12,043**
	C6	51	305	1,531	194	73	7,755	482	1,245	**11,636**
	C7	74	305	1,531	310	73	7,767	478	1,246	**11,784**
	C8	112	310	1,531	194	87	8,007	423	1,324	**11,988**
	C9	112	310	1,531	194	87	7,567	385	1,108	**11,294**
	C10	119	310	1,531	233	87	7,995	414	1,313	**12,003**
	Mean C1-C10	99	309	1,531	221	82	8,041	462	1,266	**12,011**
	Percentage of total	1%	3%	13%	2%	1%	67%	4%	11%	**100%**
Inguinal hernia repair	H1	181	1,293	1,677	929	312	5,859	747	1,442	**12,440**
	H2	171	1,293	1,531	881	301	5,445	704	1,351	**11,676**
	H3	283	120	2,154	1,161	58	5,168	737	912	**10,593**
	H4	357	282	2,299	1,457	101	8,709	767	991	**14,963**
	H5	283	163	2,154	1,162	72	4,862	665	912	**10,273**
	H6	356	282	2,154	1,457	90	4,492	569	728	**10,128**
	Mean H1-H6	272	572	1,995	1,174	156	5,756	698	1,056	**11,679**
	Percentage of total	2%	5%	17%	10%	1%	49%	6%	9%	**100%**

Knee arthroplasty	K1	519	260	13,505	388	83	68,193	6,875	5,051	**94,875**
	K2	458	256	11,351	453	80	58,858	6,064	4,758	**82,278**
	K3	520	315	13,651	453	98	59,290	6,366	4,821	**85,513**
	K4	483	272	13,505	323	87	52,205	5,234	5,000	**77,109**
	K5	501	191	13,215	388	65	65,302	6,304	5,124	**91,089**
	K6	687	331	15,659	323	102	45,874	4,608	4,663	**72,247**
	K7	682	348	15,805	323	112	56,009	6,759	5,002	**85,039**
	K8	624	337	17,959	323	108	63,401	6,334	4,949	**94,036**
	K9	488	301	13,651	323	94	61,035	6,446	5,202	**87,540**
	K10	425	242	11,351	323	76	60,982	6,353	5,059	**84,813**
	Mean K1-K6	539	285	13,965	362	90	59,115	6,134	4,963	**85,454**
	Percentage of total	1%	0.33%	16%	0.42%	0.11%	69%	7%	6%	**100%**
Laparoscopic cholecystectomy	L1	398	792	5,530	1,351	216	7,947	1,493	1,387	**19,113**
	L2	476	893	5,530	1,676	240	7,777	1,514	1,311	**19,417**
	L3	469	951	5,676	1,597	246	9,680	1,579	1,597	**21,795**
	L4	506	1,007	5,676	1,777	233	10,259	1,645	922	**22,024**
	L5	431	834	5,530	1,483	199	8,172	1,338	764	**18,751**
	L6	468	893	5,530	1,630	209	9,319	1,664	928	**20,640**
	Mean L1-L6	458	895	5,579	1,586	224	8,859	1,539	1,152	**20,290**
	Percentage of total	2%	4%	27%	8%	1%	44%	8%	6%	**100%**
Tonsillectomy	T1	199	110	2,154	457	21	3,024	222	823	**7,010**
	T2	249	184	2,154	790	51	3,152	211	542	**7,333**
	T3	249	184	2,154	790	36	3,032	202	482	**7,128**
	T4	199	110	2,154	455	21	2,548	159	743	**6,388**
	T5	199	110	2,154	455	21	2,508	154	736	**6,337**
	T6	214	110	2,154	535	22	3,686	463	986	**8,170**
	T7	206	110	2,154	493	21	3,628	459	979	**8,051**
	T8	222	110	2,154	573	22	3,630	459	979	**8,150**
	T9	214	110	2,154	532	22	3,647	454	985	**8,118**
	T10	214	110	2,154	535	22	3,591	454	973	**8,052**
	Mean T1-T10	217	125	2,154	561	26	3,245	324	823	**7,474**
	Percentage of total	3%	2%	29%	8%	0.35%	43%	4%	11%	**100**

Supplementary table 30: Carbon footprint of bone cement using process-based approach

The material composition of products were determined based upon product manufacturer data sheets,(13, 14) matching listed materials to available emission factors (Supplementary table 4). A number of materials were omitted due to lack of emission factors; for both types of bone cement N,n-dimethyl-para- toluidine and hydroquinone were omitted; for bone cement mix with gentamicin, gentamicin sulphate and dibenzoyl peroxide were also omitted; for bone cement with tobramycin, tobramycin sulphate was also omitted. Omitted materials were an estimated 4% - 7% of products by weight (excluding packaging), and omitted material weights were allocated across other product materials. The material composition of packaging was based on expert assessment, taking into account available emission factors. Carbon footprint per use equates to a single use of a product in a single operation. CO_2e= carbon dioxide equivalents, ops.= operations

Product	Component	Material(s)	Weight (g)	Number of uses	Number of ops per use	Waste stream	Carbon footprint per use (g CO_2e)		
							Production	Waste stream	Total
Bone cement mix (with gentamicin)	Product	Methyl methacrylate	36.40	1	1	N/A	261.53	N/A	261.53
		Polymethyl methacrylate	16.40				125.67		125.67
		Zirconium oxide	8.20				29.65		29.65
	Packaging	Paper	154.82			Infectious waste	230.68	88.14	318.82
		Polyethylene terephthalate	13.06				52.66	7.44	60.10
		Glass general	11.61			Medicinal contaminated sharps waste	16.72	12.47	29.19
		Polypropylene oriented film	11.42			Infectious waste	39.17	6.50	45.67
		Aluminium foil	9.17				68.50	5.22	73.72
		Low density polyethylene resin	1.18				2.45	0.67	3.13
Bone cement mix (with tobramycin)	Product	Methyl methacrylate	49.93	1	1	N/A	358.74	N/A	358.74
		Polymethyl methacrylate	6.56				50.27		50.27

		Barium Sulfide	4.51				5.34		5.34
	Packaging	Paper	83.65			Infectious waste	124.64	47.62	172.26
		Low density polyethylene film	11.02				28.66	6.28	34.94
		Glass general	9.30			Medicinal contaminated waste	13.39	9.99	23.38
		Polyethylene terephthalate	5.53			Infectious waste	22.30	3.15	25.45
		High density polyethylene resin	2.76				5.32	1.57	6.89
		Low density polyethylene resin	1.18				2.45	0.67	3.13
		Polypropylene oriented film	0.61				2.09	0.35	2.44

References

1. Vozzola E, Overcash M, Griffing E. Environmental considerations in the selection of isolation gowns: A life cycle assessment of reusable and disposable alternatives. Am J Infect Control 2018;46(8):881-6.
2. Overcash M. A comparison of reusable and disposable perioperative textiles: sustainability state-of-the-art 2012. Anesth Analg 2012;114(5):1055-66.
3. Carre A. Life cycle assessment comparing laundered surgical gowns with polypropylene based disposable gowns. Melbourne, Australia: RMIT University, 2008.
4. Department for Environment, Food and Rural Affairs/ Department for Business, Energy & Industrial Strategy. UK Government GHG Conversion Factors for Company Reporting [database]. London, UK: Department for Environment, Food and Rural Affairs/ Department for Business, Energy & Industrial Strategy, 2021.
5. European Textile Services Association. Assessment of global warming potential of two textile services. Brussels, Belgium: European Textile Services Association, 2015.
6. Inventory of Carbon and Energy v3.0 [database]. Jones C, Hammond G: University of Bath; 2019.
7. Parvatker AG, Tunceroglu H, Sherman JD, Coish P, Anastas P, Zimmerman JB, et al. Cradle-to-Gate Greenhouse Gas Emissions for Twenty Anesthetic Active Pharmaceutical Ingredients Based on Process Scale-Up and Process Design Calculations. ACS Sustainable Chemistry & Engineering. 2019;7(7):6580-91.
8. Carbon Factors Dataset version 5.3 [database]. Small World Consulting: Lancaster University; 2020.
9. UK Government GHG Conversion Factors for Company Reporting [database]. Department for Environment, Food and Rural Affairs/ Department for Business, Energy & Industrial Strategy; 2021.
10. SimaPro Version 9.10, Ecoinvent (version 3.6) [database]. PRé Sustainability, Amersfoort, Netherlands, 2019.
11. Rizan C, Lillywhite R, Reed M, Bhutta M. Minimising carbon footprint and financial costs of decontamination and packaging reusable surgical instruments. British Journal of Surgery 2022;109:200-10.
12. Rizan C, Bhutta MF, Reed M, Lillywhite R. The carbon footprint of waste streams in a UK hospital. Journal of Cleaner Production 2021;286:125446.
13. Stryker. Material safety data sheet- product name: Simplex® HV cement with Gentamicin. [Internet]. 2014; [cited 2022 Jan 10]. Available from: https://www.strykermeded.com/media/2027/simplex-hv-cement-with-gentamicin.pdf
14. Stryker. Material safety data sheet- antibiotic simplex with Tobramycin. [Internet]. 2017; [cited 2022 Jan 10]. Available from: https://www.strykermeded.com/media/2792/antibiotic-simplex-p-with-tobramycin_sds_nov_2019.pdf
